# Supplementary material for: The impacts of antipsychotic medications on eating-related outcomes: A mixed methods systematic review
Source: PLoS One. 2025 Feb 3;20(2):e0308037. doi: 10.1371/journal.pone.0308037 (PMC11790239; doi:10.1371/journal.pone.0308037)
Supplement: S2 File — (DOCX) [file pone.0308037.s002.docx]

**S2 File. Information Sources and Search Strategy for the Systematic Review.**

A comprehensive literature search was initially run on 9 November 2021, then rerun on 15 March 2023 and 9 May 2024. The search included the following 3 electronic databases:

- Psychological Information Database (PsycInfo; OVID interface, from inception 1872),
- Medical Literature Analysis and Retrieval System Online (MEDLINE; OVID interface, from inception 1946),
- Web of Science Core Collection.

The electronic databases were selected based on their scope and were searched from inception. Databases available through the same interface were searched separately. To supplement the electronic databases search, reference lists of included study reports and relevant systematic reviews [1, 2] were searched to identify further eligible study reports.

- 1. **Search strategy in the electronic databases:**
     1. ***Web of Science Core Collection from inception to 15 March 2023:***

(TS=((appetite OR eating OR hunger OR satiation OR satiety OR polyphagia OR hyperphagia OR craving) )) AND TS=((Antipsychotic* OR anti-psychotic*))

- - 1. ***APA PsycInfo Ovid 1806 to March Week 1 2023:***

1. Antipsychotic Agents/

2. anti-psychotic*.mp.

3. antipsychotic*.mp.

4. 1 or 2 or 3

5. Appetite/

6. appetite.mp.

7. "Feeding and Eating Disorders"/

8. "Feeding and Eating Disorders of Childhood"/

9. Binge-Eating Disorder/

10. Night Eating Syndrome/

11. Eating/

12. eating.mp.

13. Feeding Behavior/

14. Hunger/

15. hunger.mp.

16. Satiation/

17. satiation.mp.

18. satiety.mp.

19. polyphagia.mp.

20. Hyperphagia/

21. hyperphagia.mp.

22. Craving/

23. craving.mp.

24. 5 or 6 or 7 or 8 or 9 or 10 or 11 or 12 or 13 or 14 or 15 or 16 or 17 or 18 or 19 or 20 or 21 or 22 or 23

25. 4 and 24

- - 1. ***Ovid MEDLINE(R) 1946 to February Week 5 2023***

1. Antipsychotic Agents/

2. anti-psychotic*.mp.

3. antipsychotic*.mp.

4. 1 or 2 or 3

5. Appetite/

6. appetite.mp.

7. "Feeding and Eating Disorders"/

8. "Feeding and Eating Disorders of Childhood"/

9. Binge-Eating Disorder/

10. Night Eating Syndrome/

11. Eating/

12. eating.mp.

13. Feeding Behavior/

14. Hunger/

15. hunger.mp.

16. Satiation/

17. satiation.mp.

18. satiety.mp.

19. polyphagia.mp.

20. Hyperphagia/

21. hyperphagia.mp.

22. Craving/

23. craving.mp.

24. 5 or 6 or 7 or 8 or 9 or 10 or 11 or 12 or 13 or 14 or 15 or 16 or 17 or 18 or 19 or 20 or 21 or 22 or 23

25. 4 and 24

**References**

1. Stogios N, Smith E, Asgariroozbehani R, Hamel L, Gdanski A, Selby P, et al. Exploring patterns of disturbed eating in psychosis: A scoping review. Nutrients. 2020;12(12). doi: 10.3390/nu12123883.

2. Mutwalli H, Keeler JL, Bektas S, Dhopatkar N, Treasure J, Himmerich H. Eating cognitions, emotions and behaviour under treatment with second generation antipsychotics: A systematic review and meta-analysis. J Psychiatr Res. 2023;160:137-62. doi: 10.1016/j.jpsychires.2023.02.006.
