# Supplementary material for: The impacts of antipsychotic medications on eating-related outcomes: A mixed methods systematic review
Source: PLoS One. 2025 Feb 3;20(2):e0308037. doi: 10.1371/journal.pone.0308037 (PMC11790239; doi:10.1371/journal.pone.0308037)
Supplement: S4 File — (DOCX) [file pone.0308037.s004.docx]

**S4 File. Methods used to prepare the quantitative data for synthesis.**

- 1. **Effect measures in quantitative studies**
     1. ***Unit of analysis issues within primary studies***

All included primary studies assessed eating-related outcomes at an individual level.

- - 1. ***Dichotomous outcome data***

Dichotomous outcome data were analysed by calculating unadjusted odds ratio (OR) of developing the outcome of interest in the treatment group compared to the reference group (i.e., control or unexposed group).

- - 1. ***Continuous outcome data***

Continuous outcome data were analysed by calculating the absolute difference between the mean values in the two groups of interest (in between-group analyses; treatment group mean value at endpoint – reference group mean value at endpoint) or between pre- and post-treatment values (in within-group analyses; post-treatment mean value - baseline mean value). If not already reported, a statistical significance test for mean difference between two independent samples was calculated using an unpaired t-test. If the standard error of the mean of one group was two times or more than that of the other group, then a t-test of unequal variance was used instead. These calculations were performed in STATA 15.1. If not already reported, a statistical significance test for mean difference between two dependent samples could not be calculated unless the number of pairs, mean difference and the standard deviation of the mean difference were provided. Even though p-values were reported for completion of data presentation, they were not incorporated in the synthesis.

When synthesising findings from non-randomised studies, adjusted effect estimates are preferred over unadjusted estimates due to the high potential of bias caused by confounding in these studies. However, most of the non-randomised studies included in this review did not adjust for potentially important confounders. Studies which did take account of confounding, either in the design or analysis phases, adjusted for different variables. We decided to extract both unadjusted effect estimates and adjusted estimates derived from the final, fully adjusted models in these studies. Unadjusted and adjusted effect estimates were compared to determine the magnitude of the confounding effect. However, the effect direction binary statistic was based on the adjusted effect estimate only.

Given the wide variation in the operationalisation of each eating-related outcome, it was inappropriate to calculate standardised mean difference (SMD).

- 1. **Handling missing data**

Six records [1-6] presented some or all of the results of intertest for this review in graphical format, rather than numerical format. Lead authors of these records were contacted via email and requested to provide the missing data. In the absence of a response, the graphical representation of results was used to determine the direction of effect. When this approach was employed, it was clearly indicated in the detailed ‘Summary of effect measures of antipsychotic medications on eating-related outcomes’ tables and ‘GRADE’ tables provided in the Supplementary Files.

One record [7] presented the prevalence of change in eating-related outcomes as proportions (%) without providing absolute numbers. The lead author was contacted to provide further details. In the absence of a response, approximate numbers were calculated based on the available data.

Ten randomised controlled trials did not provide complete outcome data [4, 6, 8-15]. Lead authors were contacted to provide further details. The analytical sample was unclear in two studies [8, 15]. However, results of the study conducted by Bitter, Treuer (8) were included in the narrative synthesis as the data represented a head-to-head comparison between different forms of olanzapine. It was unclear whether the study conducted by Fountaine, Taylor (15) used a per protocol or intention-to-treat analysis. Data of interest were not reported in one study [11]. This was clearly indicated in the Study and Participant Characteristics table (S5 File). One study [14] presented the prevalence of eating disorders as percentages without providing absolute numbers. The outcomes of this study were included in the narrative synthesis as comparative data were not provided. One study [6] presented one of the outcomes of interest as a line graph (no numerical data were provided). One study [13] had considerable loss to follow-up: 33.53% of participants in the olanzapine arm and 53.18% of participants in the haloperidol arm were lost to follow-up. However, this study used an intention-to-treat analysis and provided prevalence of decreased appetite in each arm. The outcomes of this study were included in the narrative synthesis as comparative data were not provided. One study [12] used last available observations for participants who were lost to follow-up before five weeks (post-treatment assessment time). One study, reported in two records [10, 16], presented the Three Factor Eating Questionnaire scores for 17/50 participants and the Food Craving Inventory scores for 50/50 participants. The difference in analytical samples was clearly reported in the corresponding summary of effect measures of antipsychotics on eating-related outcomes tables and GRADE tables. One study, reported in two records [9, 16], presented a different overall sample size across 2 tables (Table 2 and 3). We used the he sample size reported in Table 2 as it included the outcome of interest.

**References**

1. Smith RC, Rachakonda S, Dwivedi S, Davis JM. Olanzapine and risperidone effects on appetite and ghrelin in chronic schizophrenic patients. Psychiatry Res. 2012;199(3):159-63. doi: 10.1016/j.psychres.2012.03.011.

2. Teff KL, Rickels MR, Grudziak J, Fuller C, Nguyen HL, Rickels K. Antipsychotic-induced insulin resistance and postprandial hormonal dysregulation independent of weight gain or psychiatric disease. Diabetes. 2013;62(9):3232-40. doi: 10.2337/db13-0430.

3. Teff KL, Rickels K, Alshehabi E, Rickels MR. Metabolic Impairments Precede Changes in Hunger and Food Intake Following Short-Term Administration of Second-Generation Antipsychotics. J Clin Psychopharmacol. 2015;35(5):579-82. doi: 10.1097/JCP.0000000000000393.

4. Ballon JS, Pajvani UB, Mayer LE, Freyberg Z, Freyberg R, Contreras I, et al. Pathophysiology of drug induced weight and metabolic effects: findings from an RCT in healthy volunteers treated with olanzapine, iloperidone, or placebo. J Psychopharmacol. 2018;32(5):533-40. doi: 10.1177/0269881118754708.

5. Blouin M, Tremblay A, Jalbert ME, Venables H, Bouchard RH, Roy MA, et al. Adiposity and eating behaviors in patients under second generation antipsychotics. Obesity (Silver Spring). 2008;16(8):1780-7. doi: 10.1038/oby.2008.277.

6. Roerig JL, Mitchell JE, de Zwaan M, Crosby RD, Gosnell BA, Steffen KJ, et al. A comparison of the effects of olanzapine and risperidone versus placebo on eating behaviors. J Clin Psychopharmacol. 2005;25(5):413-8. doi: 10.1097/01.jcp.0000177549.36585.29.

7. Treuer T, Hoffmann VP, Chen AK, Irimia V, Ocampo M, Wang G, et al. Factors associated with weight gain during olanzapine treatment in patients with schizophrenia or bipolar disorder: results from a six-month prospective, multinational, observational study. World J Biol Psychiatry. 2009;10(4 Pt 3):729-40. doi: 10.1080/15622970903079507.

8. Bitter I, Treuer T, Dilbaz N, Oyffe I, Ciorabai EM, Gonzalez SL, et al. Patients' preference for olanzapine orodispersible tablet compared with conventional oral tablet in a multinational, randomized, crossover study. World J Biol Psychiatry. 2010;11(7):894-903. doi: 10.3109/15622975.2010.505663.

9. Hardy T, Henry RR, Forrester TD, Kryzhanovskaya LA, Watson SB, Marks DM, et al. Insulin Sensitivity in Patients with Schizophrenia or Schizoaffective Disorder Treated with Olanzapine or Risperidone. 162nd Annual Meeting Shaping our Future: Science and Service; San Francisco: American Psychiatric Association; 2009. p. 14-5.

10. Hoffman VP, Case M, Jacobson JG. Algorithms including amantadine, metformin and zonisamide for mitigation of weight gain during olanzapine treatment in outpatients with schizophrenia. APA San Francisco 2009.

11. Daurignac E, Leonard KE, Dubovsky SL. Increased lean body mass as an early indicator of olanzapine-induced weight gain in healthy men. Int Clin Psychopharmacol. 2015;30(1):23-8. doi: 10.1097/YIC.0000000000000052.

12. Kane JM, Marder SR, Schooler NR, Wirshing WC, Umbricht D, Baker RW, et al. Clozapine and haloperidol in moderately refractory schizophrenia: a 6-month randomized and double-blind comparison. Arch Gen Psychiatry. 2001;58(10):965-72. doi: 10.1001/archpsyc.58.10.965.

13. Tollefson GD, Beasley CM, Jr., Tran PV, Street JS, Krueger JA, Tamura RN, et al. Olanzapine versus haloperidol in the treatment of schizophrenia and schizoaffective and schizophreniform disorders: results of an international collaborative trial. Am J Psychiatry. 1997;154(4):457-65. doi: 10.1176/ajp.154.4.457.

14. Khazaal Y, Fresard E, Rabia S, Chatton A, Rothen S, Pomini V, et al. Cognitive behavioural therapy for weight gain associated with antipsychotic drugs. Schizophr Res. 2007;91(1-3):169-77. doi: 10.1016/j.schres.2006.12.025.

15. Fountaine RJ, Taylor AE, Mancuso JP, Greenway FL, Byerley LO, Smith SR, et al. Increased food intake and energy expenditure following administration of olanzapine to healthy men. Obesity (Silver Spring). 2010;18(8):1646-51. doi: 10.1038/oby.2010.6.

16. Case M, Treuer T, Karagianis J, Hoffmann VP. The potential role of appetite in predicting weight changes during treatment with olanzapine. BMC Psychiatry. 2010;10:72. doi: 10.1186/1471-244X-10-72.
