# Supplementary material for: The impacts of antipsychotic medications on eating-related outcomes: A mixed methods systematic review
Source: PLoS One. 2025 Feb 3;20(2):e0308037. doi: 10.1371/journal.pone.0308037 (PMC11790239; doi:10.1371/journal.pone.0308037)
Supplement: S5 File — (DOCX) [file pone.0308037.s005.docx]

**S5 File. Study and participant characteristics of the 55 included quantitative studies.**

| **Citation** | **Country** | **Study characteristics** | | | | | | | **Outcome (measurement)** | **Funding** | **Additional data** |
| --- | --- | --- | --- | --- | --- | --- | --- | --- | --- | --- | --- |
|  |  | **Aim(s)** | **Study design** | **Study setting** | **Study population**  **Analytical sample size** | **Antipsychotics, n** | **Sampling strategy** | **Follow-up period/Drop-out** |  |  |  |
| **Quantitative randomised controlled trials** | | | | |  |  | | | | |  |
| (Ballon et al., 2018) | USA | to investigate metabolic effects of olanzapine and iloperidone versus placebo in healthy volunteers | 3-arm double-blind, parallel RCT | Lab-based | **Sample size:** **n= 24**  Olanzapine arm: n= 7;  iloperidone arm: n=7;  placebo arm: n=10  **Study population:**  Healthy volunteers  **Age, mean (SD)**:  Olanzapine arm: 26.70 (2.00);  Iloperidone arm: 27.30 (1.60)  Placebo arm: 25.80 (1.50)  **Sex, male n**:  Olanzapine arm: 5  Iloperidone arm: 4  Placebo arm: 9  **Ethnicity, n:**  Olanzapine arm: 5 Black, 1 White, 1 Other  Iloperidone arm: 5 Black, 1 White, 1 Other  Placebo arm: 4 Black, 4 White, 2 Other **Baseline BMI (kg/m^2^), mean (SD):**  Olanzapine arm: 22.70 (0.30)  Iloperidone arm: 23.60 (0.60)  Placebo arm: 22.70 (0.50) | Olanzapine 7;  Iloperidone 7; | **Randomisation:** standard block randomisation to the olanzapine, iloperidone, or placebo arms. **Sequence generation:** no information **Allocation concealment:** no information **Blinding**: double blinding with no further information | **Follow-up:** 28 days **Loss to follow-up:** Of 30 randomised participants, only 24 completed  the study and were included in the per-protocol analysis (6/30 dropped-out; 3 for lack of compliance and 3 for adverse effects). | 1. **Food intake** Gram weight, kcal macronutrient composition of all food consumed from a standardised laboratory meal.   **2.** **Hunger, fullness, desire to eat:** Measured using a 100-mm visual analogue scale (VAS) prior and subsequent to a standardised laboratory lunch meal. | Pharmaceutical funding  Conflicts of interest | Mean (SD) premeal hunger scores (mm) on day 28 in olanzapine arm: 89 (11);  Iloperidone arm: 86 (19). |
| (Bitter et al., 2010) | Multicentre  (Brazil, Israel, Mexico, Romania, Turkey) | to compare patients’ preference for olanzapine orodispersible tablet (ODT) with oral conventional tablet (OCT). | Randomised, open-label, two period crossover trial | Outpatient | **Sample size:** **n= 207**  (Analytical sample size of interest is unclear. It included all patients who received at least 1 dose of study medication during study)  **Study population:**  Schizophrenia  Diagnostic criteria: DSM-IV or DSM-IV-TR.  **Age, mean (SD)**: 35.3 (11.1)  **Sex, male n (%)**: 131 (63.3)  **Ethnicity, n (%):** Caucasian 131(63.3), Hispanic 54 (26.1), other 22 (10.6)  **Country of origin, n:** Brazil 52, Israel 20, Mexico 49, Romania 42, Turkey 102.  **Baseline BMI (kg/m^2^), mean (SD):** 26.9 (5.0) | Olanzapine 207: ODT mean dose: 12.3mg/d,  OCT mean dose: 12.4mg/d.  **Prior AP treatment, n:** no AP treatment for past 2 years: 44; AP-naïve: 1; AP monotherapy: 81; >1 AP: 81 | **Randomisation:** simple 1:1 ratio  **Sequence generation:** computer generated randomisation sequence.  **Allocation concealment:** no information | **Follow-up:** 12 weeks  **Loss to follow-up:** Of 265 randomised participants, only 207 were included in per protocol analysis (90/265 lost to follow-up; completers: 175). | 1. **Appetite:**   Measured using a 100-mm VAS | Pharmaceutical funding  Conflicts of interest | Comparison between ODT, OCT:  MD (SD) in appetite in ODT group: −0.1 (14.9); in OCT group 2.0 (14.7).  Analytical sample size is unclear. |
| (Case et al., 2010)  Study 1:  (Hardy et al., 2009) | USA | Secondary analysis of data collected from (Hardy et al., 2009) to investigate the association between changes in appetite and weight gain during olanzapine treatment. | 2-arm parallel, double-blind RCT  (Analysis of interest: data collected from participants in olanzapine arm) | Community/  outpatient | **Sample size:** **n= 68**  **Study population, n (%):**  Schizophrenia 41 (60.29), schizoaffective disorder 27 (39.71)  Diagnostic criteria: DSM-IV-TR criteria  **Age, mean (SD)**: 43.50 (9.50)  **Sex, male n (%):** 45 (66.20) **Ethnicity, n (%):**  White 27 (39.70), African American 34 (50.00), East/Southeast Asian 1 (1.50), Hispanic 4 (5.9), West Asian 0, Other 2 (2.90).  **Baseline BMI (kg/m^2^), mean (SD):** 28.96 (5.17) | Olanzapine 68  **Mean modal daily dose of olanzapine:** 12.9 mg  **Prior AP treatment:** Participants excluded if they had olanzapine, risperidone, or depot APs within 4 weeks of study entry, or clozapine within 2 years of entry.  Patients discontinued previous AP 3-10 days before enrolment.  **Use of other** **medications**: Other APs, mood stabilizers (not SSRIs) was not allowed during the study.  Participants received 4 dietary consultations and were encouraged to have regular physical activity. | **Randomisation**: no information **Sequence generation:** no information **Allocation concealment:** no information **Blinding**: double blind but no further information. | **Follow-up:** Secondary analysis of weight and appetite data collected at baseline and at 2 weeks. | **Appetite changes:** Measured appetite, hunger, craving, fullness and eating behaviour collectively using the Eating Behavior Assessment (EBA; non-validated). | Pharmaceutical company  Conflicts of interest |  |
| (Case et al., 2010)  Study 2 (Karagianis et al., 2009) | Multicentre (Canada, the Netherlands, USA, Mexico) | Secondary analysis of data collected from (Karagianis et al., 2009) to investigate the association between changes in appetite and weight gain during olanzapine treatment. | 2-arm parallel, double-blind RCT  (Analysis of interest: data collected from participants in SOT arm) | Community/  outpatient | **Sample size: n= 65**  **Study population, n (%):**  Schizophrenia 36 (55.40); bipolar disorder 14 (21.50); schizoaffective disorder 12 (18.50); schizophreniform 2 (3.10); other related psychiatric disorder 1 (1.50).  Diagnostic criteria: DSM-IV or DSM-IV-TR.  **Age (years), mean (SD)**: 38.70 (12.20) **Sex, male n (%):** 33 (50.80)  **Baseline BMI (kg/m^2^), mean (SD):** 28.30 (4.80)  **Ethnicity, n (%):** White 33 (50.80); African American 4 (6.20); East/Southeast Asian 1 (1.50); Hispanic 23 (35.40); Other 1 (1.50).  At enrolment, patients had been taking 5-20 mg standard olanzapine tablets/day between 4-52 weeks and experienced weight gain ≥5 kg or a change of ≥1 kg/m^2^ BMI.  BMI increase on prior olanzapine treatment, mean (SD): 2.6 (1.21) | Olanzapine 65  **Mead daily dose of olanzapine:** 13.23 mg  **Duration of prior olanzapine treatment** in weeks, mean (SD): 19.90 (13.80).  **Use of other medications** that could affect weight or participating in a weight loss program were exclusion criteria. | **Randomisation**: blocked (1:1), stratified randomisation by baseline BMI (BMI <30 and BMI ≥30 kg/m2) and investigation site. **Sequence generation:** no information **Allocation concealment:** no information **Blinding**: double blind but no further information | **Follow-up:** Secondary analysis of weight and appetite data collected at baseline and at 2 weeks. | **Appetite changes:** Measured using Platypus Appetite Rating Scale (PARS; non-validated measure), no details regarding constructs being tested. | Pharmaceutical company  Conflicts of interest |  |
| (Case et al., 2010)  Study 3  (Hoffman et al., 2009) | Multicentre (Brazil, Israel, Republic of Korea, Mexico, Puerto Rico, Russia, USA) | Secondary analysis of data collected from (Hoffman et al., 2009) to investigate the association between changes in appetite and weight gain during olanzapine treatment. | 3-arm parallel, open label RCT  (Reported analysis: olanzapine only arm) | Community/  outpatient | **Sample size: n= 50**  **Study population, n (%):**  Schizophrenia 42 (84); schizoaffective disorder 8 (16)  Diagnostic criteria: DSM-IV-TR  **Age (years), range**: 38.7 (8.9)  **Sex, male n (%):** 31 (62)  **Ethnicity, n (%):** African 2 (4.0); Caucasian 23 (46.0); East Asian 10 (20.0); Hispanic14 (28.0); West Asian 1 (2.0)  **Baseline BMI (kg/m^2^), mean (SD):**  27.0 (4.5)  (Sociodemographic data extracted from olanzapine only arm reported in (Hoffmann et al., 2012)). | Olanzapine 50  **Mean modal daily dose of olanzapine:** 13.33 mg  Brief weight management education was provided at baseline.  **Prior AP treatment:** NR | **Randomisation**: no information **Sequence generation:** no information **Allocation concealment:** no information | **Follow-up:** Secondary analysis of weight and appetite data collected at baseline and at 2 weeks. | **Appetite changes:** Measured using the Eating Inventory (EI; validated), Food Craving Inventory (FCI; validated) | Pharmaceutical company  Conflicts of interest |  |
| (Daurignac et al., 2015) | USA | To investigate the effect of olanzapine on early changes in weight, behaviour, and metabolism in healthy participants. | 2-arm parallel, double-blind RCT | Lab-based | **Sample size:** **n= 19**  Olanzapine arm: n= 13;  Placebo arm: n=6  **Study population:**  Healthy volunteers  **Age (years), mean (SD)**:  Olanzapine arm: 23.1 (5.1)  Placebo arm: 24.8 (2.5)  **Sex, male n**:  19 (100)  **Baseline BMI (kg/m^2^), mean (SD):**  Olanzapine arm: 25.3 (3.0)  Placebo arm: 24.2 (2.9) | Olanzapine 13  Mean (SD) daily olanzapine dose (mg) for 1^st^ week: 5 (0.0) mg/day, for 2^nd^ week 9.3 (1.4) | **Randomisation**: no information **Sequence generation:** no information **Allocation concealment:** no information **Blinding**: double blind but no further information | **Follow-up:** 2 weeks  **Loss to follow-up/dropouts:**  Of 24 participants, 19 completed the 2-week study period (loss to follow-up: 1 from olanzapine arm, 4 from placebo arm), were included in per protocol analysis. | 1. **Food intake:**   Measured using 24-hour dietary recall (at baseline, day 12), and gram weight of all food consumed at a lab-based breakfast assessment (at baseline, day 12). | Pharmaceutical funding  Conflicts of interest | 24-hour intake: data: NR; but did not change significantly  in both arms. Laboratory breakfast assessment data: NR; but did not change significantly in both arms. |
| (Fountaine et al., 2010) | USA | to investigate potential mechanisms of olanzapine-induced weight  gain and accompanying metabolic effects in healthy volunteers. | Randomised, double blind, placebo controlled two period crossover trial | Lab setting | **Sample size: n= 30 (21 completers)**  **Study population:**  Healthy volunteers  **Age (years), mean (range)**: 27 (18-49)  **Sex, male n (%):** 30 (100)  **Baseline BMI (kg/m^2^), mean (SD):** 22.60 (2.20) | Olanzapine and placebo treatments were  administered on study days 1-15 of each study period. | **Randomisation:** no information. **Sequence generation:** computer-  generated randomisation schedule. **Allocation concealment:** no information. **Blinding**: double blinding with no further information. | **Follow-up:**  Sequence 1: participants received placebo for 15 days (period 1),  then olanzapine (period 2: 5 mg/day for 3 days, then 10 mg/day for  12 days).  Sequence 2: order reversed. Washout period: 12 days.  **Loss to follow-up:** 9. | 1. **Food intake:**   Gram weight of all food consumed on day 14 of study periods 1 and 2. | Pharmaceutical company  Conflicts of interest |  |
| (Holt et al., 2018) | UK | To develop a lifestyle intervention for people with first episode psychosis or schizophrenia and to evaluate its clinical effectiveness, cost-effectiveness, delivery and acceptability' | 2-arm, analyst-blind, parallel-group RCT  (Analysis of interest: baseline data of whole sample) | Community/  outpatient | **Sample size: n= 412**  Intervention arm: n= 207  Control arm: n= 205  **Study population, n (%):**  Schizophrenia or schizoaffective disorder [n=63/412 (15.29) had FEP, defined as < 3 years since presentation to the mental health team].  Diagnostic criteria: ICD-10  **Age (years), mean (SD)**:  Intervention arm: 40.00 (11.30)  Control arm: 40.10 (11.50)  **Sex, male n (%):**  Intervention arm: 115 (55.60)  Control arm: 95 (46.30)  **Ethnicity, n (%):**  Intervention arm: White 179 (86.50), Asian 9 (4.30), Black 12 (5.80), Mixed 4 (1.90), Other 3 (1.40).  Control arm: White 170 (82.90), Asian 7 (3.40), Black 19 (9.30), Mixed 7 (3.40), Other 2 (1.00).  **Baseline BMI (kg/m^2^), mean (SD):**  Intervention arm (206/207): 36.10 (7.20)  Control arm (204/205): 35.30 (7.20)  **Proportion of sample receiving APs, n (%):**  Intervention arm: 207 (100.0)  Control arm: 204 (99.50) | Haloperidol 10, amisulpride 37, aripiprazole 74, clozapine 170, olanzapine 62, quetiapine 52, risperidone 31, flupentixol 19, zuclopenthixol 31, paliperidone 15, other APs 28  **Duration of AP treatment in years, median (IQR):**  Intervention arm 13 (5-20)  Control arm 12 (5-20)  Any antidepressant use (including lithium, mirtazapine, citalopram, others): Intervention arm 131 (63.30%)  Control arm 129 (62.90%) | **Randomisation:** permuted block randomisation using a 1:1 ratio, stratified by site and time since start of AP (< 3 months or ≥ 3 months).  **Sequence generation:** generated using the CTRU web-based system, **Allocation concealment:** an unblinded member of the site research team informed the participant and their GP of the treatment allocation **Blinding:** Outcome assessors were blinded to treatment allocation. | Analysis of interest: baseline data of whole sample (n= 412) | **1. Self-reported nutrition:** Measured using the adapted Dietary Instrument for Nutrition Education (DINE) questionnaire | Non-pharmaceutical company funding  Conflicts of interest | **Macronutrient intake in whole sample:**   - **Baseline fat intake (g):** Low: 46.60% (n= 192/412) *, medium: 31.07% (n= 128/412) *, high: 22.33% (n= 92/412) * - **Baseline unsaturated fat intake:** Low: 4.37% (n= 18/412), medium: 45.39% (n= 187/412), high: 50.00% (n= 206/412) - **Baseline daily sugar intake (g):** 80.78 - **Baseline fibre intake (g):** Low: 58.74% (n= 242/412) *, medium: 25.49% (n= 105/412) *, high: 15.78% (n= 65/412) * - **Baseline weekly alcohol (units):** 3.80 |
| (Kane et al., 2001) | USA | To compare efficacy of clozapine with moderate-dose haloperidol in partially responsive, community-based patients. | 2-arm parallel, double-blind RCT | Community | **Sample size, n= 71**  Clozapine arm: n = 37  Haloperidol arm: n = 34  **Study population, n (%):**  Schizophrenia or schizoaffective disorder with partial or poor response (i.e.: failure in 2 trials of conventional APs at dosages equivalent to ≥ 600 mg/d chlorpromazine hydrochloride for at least 6 weeks and 1 trial of conventional AP at dosages equivalent to 250-500 mg/d chlorpromazine hydrochloride, for same length of time).  Diagnostic criteria: DSM-III-R  **Age (years), mean (SD)**:  Clozapine arm: 41 (10)  Haloperidol arm: 40 (8)  **Sex, male n (%):**  Clozapine arm: 26 (70)  Haloperidol arm: 24 (71)  **Ethnicity, n (%):**  Clozapine arm: White 25 (68)  Haloperidol arm: White 24 (71)  **Baseline BMI (Kg/m2), mean (SD):** NR | Clozapine arm: 37  Haloperidol arm: 34  **Duration of prior AP treatment in months, mean (SD):**  Clozapine arm: 195 (119)  Haloperidol arm: 203 (96) | **Randomisation:** blocked by site  **Sequence generation:** Computer-generated randomisation schedules  **Allocation concealment:** no information  **Blinding:** double blind treatment administration. | **Follow-up:** 29 weeks  **Loss to follow-up/drop out:**  Of the 71 participants, 34 dropped out.  Clozapine arm: 13  Haloperidol arm: 22 (N.B.: outcome of interest was ascertained for all 71 participants at 5 weeks or earlier). | 1. **Anorexia/decreased appetite:**   Measured using a checklist of adverse effects completed at 5 weeks or last available observation if that occurred before 5 weeks. | Partial pharmaceutical funding  Conflicts of interest | Haloperidol-treated patients experienced a greater decrease in appetite than clozapine-treated patients  (Mean (SD) decreased appetite rating in clozapine-treated patient: 1.05 (2.03); in haloperidol-treated patients: 1.41 (0.61)). |
| (Kang et al., 2024) | China | To evaluate the effect of continuous theta burst stimulation on olanzapine-induced weight gain | 2-arm parallel, double-blind RCT  (Analysis of interest: data from the olanzapine and sham intervention arm) | Inpatient | **Sample size: n= 19**  **Study population, n (%):**  First episode (< 2 years) drug-naive individuals with schizophrenia.  Diagnostic criteria: DSM-5 criteria, clinician diagnosis  **Age (years), mean (SD)**: 24.55 (6.22)  **Sex, male n (%):** 2 (10.53)  **Baseline BMI (Kg/m2), mean (SD):**  21.70 (3.71) | Olanzapine 19  Participants received olanzapine and sham intervention for 5 days.  **Mean Daily Dose (SD) of olanzapine, mg/day:** 15.26 (4.57)  **No prior AP treatment.** | **Randomisation:** simple 1:1 ratio  **Sequence generation:** Computer-generated randomisation schedules  **Allocation concealment:** sealed randomisation envelopes used for participant assignment  **Blinding:** double blind treatment administration (including researchers) | Of 20 participants randomised to the sham arm, 1 dropped out. | **1. Eating cognitions and behaviours:**  Three-Factor Eating Questionnaire-Revised 21-item Version (TFEQ-R21) at baseline and day 6. | Non-pharmaceutical funding.  No conflicts. |  |
| (Khazaal et al., 2007) | Switzerland | To evaluate the effectiveness of CBT on eating and weight-related cognitions, binge eating symptomatology and weight loss in patients who reported weight gain during AP treatment. | 2-arm parallel open-label RCT  (Analysis of interest: baseline data of whole sample) | Community/  outpatient | **Sample size: n= 61**  **Study population, n (%):**  Schizophrenia and schizoaffective disorders 45 (73.8); bipolar disorder 5 (8.2), schizotypal disorder 4 (6.6), depression or personality disorders 7 (11.5).  Diagnostic criteria: DSM-IV criteria  **Age (years), mean (SD)**: 40.7 (10.3)  **Sex, male n (%):** 28 (45.9)  **Baseline BMI (Kg/m2), mean (SD):**  30 (6.3) | Olanzapine 29, risperidone 8, clozapine 11, quetiapine 2, amisulpride 5, FGA 6 | **Randomisation**: no information **Sequence generation**: no information **Allocation concealment, blinding:** NA | **Analysis of interest:** baseline data of the whole sample (n= 61) | **1. Eating-related cognitions:** Measured using MAC-R. **2. Binge eating symptomatology:** Assessed using DSM-IV criteria.  Participants were classified into 4 categories: no binge eating; binge episodes lasting less than 2 days a week and occurring more than once a month (Binge symptomatology: BS); BED; bulimia nervosa. | Funding: not stated  Conflicts: not stated | N (%) of:  BED in overall sample: n= 14/61 (23)  BED and BS in overall sample: n= 35/61 (56.6) |
| (Kluge et al., 2007) | Germany | To investigate abnormal eating behaviour (food craving, binge eating) and weight gain associated with clozapine and olanzapine treatment | 2-arm parallel, double-blind RCT | Inpatient | **Sample size: n= 30**  Clozapine arm: n= 15; Olanzapine arm: n= 15  **Study population, n (%):**  Schizophrenia 26 (86.67) , schizophreniform 3 (10.00), schizoaffective disorder 1 (3.30).  Diagnostic criteria: DSM-IV  **Age (years), mean (SD)**:  Clozapine arm: 36.70 (13.00)  Olanzapine rm: 32.80 (8.30) **Sex, male n (%):**  Clozapine arm: 7 (46.70%)  Olanzapine: 5 (33.30%)  **Baseline BMI (kg/m^2^), mean (SD):**  Clozapine arm: 25.40 (5.20)  Olanzapine arm: 24.40 (5.10) | Clozapine 15 Olanzapine 15 | **Randomisation**: no information **Sequence generation:** no information **Allocation concealment, blinding**: no information | **Follow-up:** 6 weeks **Loss to follow-up:** 4  (3 in the clozapine arm, 1 in the olanzapine arm) | **1. Abnormal eating behaviour:**  Measured weekly using a physician-rated binary scale, capturing the presence or absence of 4 symptoms during the preceding week (increased appetite, increased food intake, binge eating, and food craving for sweet and fatty food).  **2. Binge-eating episodes**: identified using DSM-IV core criteria. | Pharmaceutical company  Conflicts of interest | Likelihood to experience craving for sweet and/or fatty food higher in olanzapine arm (48.9%) than  clozapine arm (23.3%); likelihood to experience  binge eating in olanzapine group (16.7%) higher than clozapine group (8.9%). |
| (Park et al., 2013) | South Korea | to compare the effects of ziprasidone and olanzapine on appetite and other parameters in adults with schizophrenia or other psychotic disorders. | 2-arm parallel open-label RCT | Outpatient (baseline and week 12 assessments were conducted in an inpatient setting) | **Sample size: n= 20**  Olanzapine arm: n=10; Ziprasidone arm: n=10  **Study population, n (%):**  New onset psychotic episode (either first onset or a new onset discontinuation-related episode) in patients diagnosed with brief psychotic disorder, schizophreniform disorder, schizophrenia, or schizoaffective disorder.  Diagnostic criteria: DSM-IV, clinician diagnosis.  N (%): NR  Participants were excluded if they had received APs during the past 3 months or had received medications (other than lorazepam, clonazepam, zolpidem, benztropine, or propranolol) for any medical or other psychiatric condition.  **Median age in years, (IQR):**  Ziprasidone arm: 34.50 ( 26.3-40.3)  Olanzapine arm: 31.50 ( 26.5-41.3)  **Sex, male n (%):**  Ziprasidone arm: 5 (50%),  Olanzapine arm: 5 (50%)  **Baseline BMI (kg/m^2^), median (IQR):**  Ziprasidone arm: 21.5 (19.2-24.0)  Olanzapine arm: 21.9 (17.9-23.7) | Olanzapine 10; Ziprasidone 10  **Daily doses** (mg/day) during the 12-week study period, mean (IQR):  Ziprasidone 109 (65-140)  Olanzapine 11.6 (8.2-15.5). | **Randomisation**: stratified (by age and sex) balanced randomisation (1:1) to ziprasidone or olanzapine  **Sequence generation:** no information  **Allocation concealment:** no information | **Follow-up:** 12 weeks | **1. Appetite:** Measured subjective hunger, fullness, desire to eat and prospective food consumption using a 10 cm VAS (non-validated). | Pharmaceutical company  Conflicts of interest | Difference between ziprasidone and olanzapine median (range) appetite scores at end of study: ziprasidone arm: 5 ( 3.38, 5.31)  Olanzapine arm: 5 (4.69, 6.13), but  Ziprasidone arm showed a greater percent change in appetite than olanzapine arm (−20% vs. 0%, p = 0.059) |
| (Roerig et al., 2005) | USA | to compare the effect of a 2-week exposure to olanzapine, risperidone, or placebo on eating behaviours in healthy participants using a feeding laboratory paradigm. | 3-arm parallel, double-blind RCT | Feeding laboratory | **Sample size: n= 48**  Olanzapine arm: n=16; Risperidone arm: n=16; Placebo arm: n=16  **Study population:**  Healthy volunteers  **Age (years), mean**:  Placebo: 32.7  Risperidone: 36.2  Olanzapine: 33.6 **Sex, male n (%):**  Placebo: 5 (31.3)  Risperidone: 4 (25.0)  Olanzapine: 2 (12.5)  **Baseline BMI (kg/m^2^), mean (SD):**  Placebo arm: 24.1  Risperidone arm: 24.97  Olanzapine arm: 23.63 | Olanzapine 16; Risperidone 16 | **Randomisation**: no information **Sequence generation:** no information **Allocation concealment:** no information **Blinding**: double blinding, no further information. | **Follow-up:** 2 weeks **Loss to follow-up:** 7  Of the 55 participants randomised, 48 completed the study and were included in per protocol analysis (7 were lost to follow-up). | **1. Hunger/appetite:**  Measured using 100-mm VAS.  **2. Calories consumed at dinner session:**  Gram weight and kcal of food consumed during the lab-based dinner session were recorded. | Pharmaceutical company  Conflicts of interest | N (%) of participants who experienced an increase in appetite: olanzapine arm 6 (37.5), risperidone arm 7 (43.8) |
| (Smith et al., 2012) | USA | to compare the effect of a 5-month treatment with olanzapine or risperidone on appetite in schizophrenia patients | 2-arm, parallel-group, open-label RCT | Inpatient | **Sample size: n= 46**  Olanzapine arm: n=23; Risperidone arm: n=23  **Study population, n:**  Olanzapine arm, n: schizophrenia 17; schizoaffective disorder 6.  Risperidone arm, n: schizophrenia 15; schizoaffective disorder 8.  Diagnostic criteria: DSM-IV  **Age (years), mean (SD)**:  Olanzapine arm 41.20 (7.30)  Risperidone arm: 42.50 (9.10)  **Sex, male n (%):**  Olanzapine arm 23 (100)  Risperidone arm 22 (95.70%)  **Ethnicity, n:**  Olanzapine arm: White 1; Black non-Hispanic 17; Hispanic 5; Other 0.  Risperidone arm: Black non-Hispanic 17; Hispanic 6.  **Baseline BMI (kg/m^2^), mean (SD):**  Olanzapine arm 29.96 (6.50)  Risperidone arm 28.85 (5.70) | Olanzapine 23; Risperidone 23  **Dose, mean (SD) mg/day:**  Olanzapine arm  25.20 (10.10)  Risperidone arm 6.10 (1.80)  **APs at baseline in olanzapine arm, n:** olanzapine 13; risperidone 6; neither olanzapine nor risperidone 4.  Other medications received:  antidepressants n=3, lithium n=4, valproate n=11.  **APs at baseline in risperidone arm, n:**  olanzapine 8; risperidone 11; neither olanzapine nor risperidone 4.  Other medications:  antidepressants n= 4; lithium n= 4; valproate n= 9.  No AP washout period. Patients switched to new AP were cross-tapered onto new AP, cross-tapered off baseline AP. | **Randomisation**: participants were assigned to olanzapine or risperidone arm, based on a stratified 4-block random assignment procedure. Stratification was based on type of prior antipsychotic treatment, length of treatment, BMI **Sequence generation:** a random number table was generated for each stratification group.  **Allocation concealment:** no information. | **Follow-up:** 5 months **Loss to follow-up:** 49 patients were randomly assigned to olanzapine or risperidone, 3 participants (2 in the olanzapine arm, 1 in the risperidone arm), did not complete at least 2 months of study treatment and were not included in the per protocol analysis. | **1. Appetite, hunger, and food craving:** Measured using the VAS, EBA (non-validated). | Pharmaceutical company  Conflicts of interest |  |
| (Teff et al., 2013) | USA | to determine if olanzapine, aripiprazole have metabolic effects independent of weight gain or psychiatric disease, in an in-patient setting while maintaining  activity levels. | 3-arm parallel, double-blind RCT | Inpatient and required to maintain their activity levels in the hospital consistent with their free-living level. | **Sample size: n= 30**  Olanzapine arm: n=10; Aripiprazole arm: n=10; Placebo arm: n=10  **Study population:**  Healthy volunteers  **Age (years), mean (SD)**:  Olanzapine arm: 26.10 (3.50)  Aripiprazole arm: 25.90 (4.30)  Placebo arm: 29.90 (7.50) **Sex, male n (%):**  Olanzapine arm: 7 (70%)  Aripiprazole arm: 7 (70%)  Placebo arm: 7 (70%)  **Baseline BMI (kg/m^2^), mean (SD):**  Olanzapine arm: 22.10 (1.40); aripiprazole arm: 22.40 (1.30); placebo: 21.80 (1.90) | Olanzapine 10; aripiprazole 10; | **Randomisation**: no details **Sequence generation:** no details **Allocation concealment:** no details **Blinding:** participants and study personnel were blinded as to the assignment. | **Follow-up:** 12 nights **Loss to follow-up:** 0 | 1. **Hunger and satiety:** Cumulative daily scores, measured using VAS, completed daily before and after each meal. 2. **Drug side effects:**   Assessed using daily symptom questionnaires. | Non-pharmaceutical  No conflicts of interest |  |
| (Teff et al., 2015) |  | to evaluate the effects of olanzapine or aripiprazole on food intake and hunger in an inpatient setting where activity was controlled. |  |  |  |  |  |  | **1. Hunger and fullness/satiety:**  Cumulative daily scores, measured using VAS, completed daily before and after each meal. **2. Food intake:** Ad libitum food intake was assessed in a lab setting on days 2 (pre- intervention) and 11 (post-intervention). |  |  |
| (Tollefson et al., 1997) | Multicentre (USA, European countries) | to compare the  therapeutic profile of olanzapine with that of haloperidol. | 2-arm parallel, double-blind RCT | Inpatient/ outpatient | **Sample size: n= 1996**  Olanzapine arm: n= 1336  Haloperidol arm: n= 660  **Study population:**  Schizophrenia, schizophreniform disorder, or schizoaffective disorder (numbers: NR)  Diagnostic criteria: DSM-III-R criteria  **Age, mean (SD)**:  Olanzapine arm: 38.7 (11.6)  Haloperidol arm: 38.3 (11.1)  **Sex, male n (%):** NR  **Country:** North America 1073, Europe 923  **Baseline BMI (kg/m^2^), mean (SD):** NR | Olanzapine 1336  Haloperidol 660  **Mean (SD) daily dose:** olanzapine  13.2 mg (5.8), haloperidol 11.8 (5.6) | **Randomisation**: block (2:1) by olanzapine: haloperidol participant ratio **Sequence generation:** no details **Allocation concealment:** no details **Blinding:** double blind but no further information | **Follow-up:** 6 weeks  **Loss to follow-up/dropout:**  Of 1336 in olanzapine arm, 448 (33.53%) were lost to follow-up. Of 660 participants in haloperidol arm, 351(53.18%) were lost to follow-up.  Intent-to-treat analysis. | 1. **Decreased appetite** as an adverse event reported in clinical report form records. | Pharmaceutical company  Conflicts of interest | N (%) of participants who reported decreased appetite:  olanzapine arm: n=149/1306 (11.4); haloperidol arm: n=115/636 (18.1); standardised effect size= -0.19 |
| (Yang et al., 2021) | China | to determine whether Bifidobacterium intervention can improve olanzapine-induced weight gain. | 2-arm, parallel-group, open-label RCT  (Analysis of interest: olanzapine only arm) | Inpatient/ outpatient | **Sample size: n= 34**  **Study population:**  Schizophrenia, schizoaffective disorder (numbers: NR).  Diagnostic criteria: DSM-5 criteria, clinician diagnosis  **Age, mean (SD)**: 23.64 (4.99)  **Sex, male n (%):** 12 (54.55)  **Baseline BMI (kg/m^2^), mean (SD):** 21.43 (1.64) | Olanzapine 34  **Mean (SD) daily dose:** 18.75 (1.97)  **Prior AP treatment:** Most patients were treated with olanzapine at enrolment, mean (SD) months= 11.26 (3.43). Most patients had been treated with single or combined APs in the past, 1 patient was AP-naïve at enrolment. | **Randomisation**: no details **Sequence generation:** no details  **Allocation concealment:** no information. | **Follow-up: 12 weeks**  **Loss to follow-up/dropout:** Of 35 participants in the olanzapine only arm, 1 participant was lost to follow-up | 1. **Appetite:**   Measured using a 3-point Likert self-report  scale (1= increased appetite, 2= decreased appetite, 3= no change in appetite), conducted 30 min before lunch on the week 4, 8, 12 visits. Patients were asked to compare changes in the past week with changes in the week before. | Non-pharmaceutical funding.  Conflicts of interest | At week 4, appetite increased in 18 patients,  decreased in 2, did not  change in 14.  At weeks 8 and 12, appetite increased in  25 patients, did not change in 9. |
| **Quantitative non-randomised studies** | | | | | | | | | | |  |
| (Stip et al., 2012) | Canada | To examine changes in the brain activity associated with 16 weeks of olanzapine  treatment in schizophrenic patients. | Non-randomised controlled trial | Outpatient | **Sample size: n= 25**  Olanzapine arm: n= 15 completers  Control arm: n= 10  **Study population:**  Olanzapine arm: Schizophrenia  Diagnostic criteria: DSM-IV  Control arm: Healthy volunteers  **Age (years), mean**:  Olanzapine arm: 35.83 (10.12)  Control arm: 33.9 (10.76)  **Sex, male n (%):**  Olanzapine arm: 11 (73.3)  Control arm: 8 (80)  **Ethnicity, n (%):**  Olanzapine arm: 13 (86.7) Caucasians, 2 (13.3) Caribbean  Control arm: 10 (100) Caucasians  **Baseline body weight (Kg), mean (SD):**  Olanzapine arm: 82.0 (16.8)  Control arm: NR | Olanzapine 15  **Daily mean (SD)**  **dosage of olanzapine:** 16.18 mg (6.97)  **Prior AP treatment, n** (21/24 participants received prior AP treatment): 12 risperidone, 6 quetiapine, 2  haloperidol, 1 clozapine, 1 perphenazine, 3 no medication. There was no washout period. (N.B.: sum > 24) | Consecutive | **Follow-up:** 16 weeks  Loss to follow-up: Of 24 patients receiving olanzapine, 9 were lost to follow-up/drop-out. | 1. **Eating cognitions and behaviours:**   Three-Factor Eating Questionnaire (TFEQ) at weeks 1 and 16 (data for control arm NR). | Pharmaceutical company  No conflicts of interest |  |
| (Briffa and Meehan, 1998) | Australia | To assess the magnitude of clozapine-induced weight gain | 12-month pre-post study | Inpatient,  community | **Sample size: n=36**  **Study population:**  Patients entering a clozapine treatment program diagnosed with schizophrenia or schizoaffective disorder (numbers: NR).  Diagnostic criteria: DSM-III-R  **Age (years), mean**:  Males: 37.2  Females: 42.6  **Sex, male n (%):** 20 (55.6)  **Baseline BMI (kg/m^2^), mean (SD):** NR | Clozapine 36  Clozapine introduced at 25 mg/day, titrated until therapeutic levels achieved. | Consecutive | **Follow-up:** 12 months  **Loss to follow-up:** Of the 51 participants who had consented to participate,15 were lost to follow-up. | 1. **Appetite:**   Changes in appetite over the first 3 months.  Assessed through interviews with the patients and/or the nursing staff caring for them.  Changes in appetite were categorised as being either ‘less’, ‘unchanged’ or ‘more’. | Funding: not stated  Conflicts of interest: not stated | N (%) of participants who described a 'craving for food' or 'feeling hungry' in the weeks following clozapine treatment: 13/36 (36.11) |
| (Friedrich et al., 2023) | Poland | To investigate whether healthy eating habits could be introduced to and  maintained by patients diagnosed with schizophrenia who reside in a nursing home.  (Analysis of interest: baseline data) | Pre-post study | Inpatient (nursing home) | **Sample size: n= 30**  **Study population:**  Schizophrenia  Diagnostic criteria: not reported  **Age (years), range**: males: 52-80; females: 45-80.  **Sex, male n (%):** 18 (60)  **Baseline BMI (kg/m^2^), mean (SD):** NR | Clozapine 12, olanzapine 15,  haloperidol 3, risperidone 3,  quetiapine 2.  (some participants were treated with 2 APs). | Not explained | Analysis of interest: baseline data. | 1. **Food intake:**   Food intake during 30 days prior to the dietary intervention. Assessed using a questionnaire developed by the authors. Data was collected in the form of ‘menus’ and used to calculate daily and macronutrient intake.  N.B.: Informed consent was provided by 21 participants and 9 proxies. | No funding.  No conflicts of interest. | Energy (MJ) mean (SD) in females: 12.6 (1.14), males 12.6 (2.31).  Carbohydrates (g) mean (SD) in females: 462 (48.8), in males 449 (74.1).  Total protein (g) mean (SD) in females:  93.2 (5.84), in males: 93.5 (13.9).  Fat (g) mean (SD) in females: 104 (8.99), in males 107 (27.3).  Cholesterol (mg) mean (SD) in females:  350 (40.7), in males 354 (58.1).  Fiber (g) mean (SD) in females:  39.5 (8.42), in males 36.0 (7.97). |
| (Ntalkitsi et al., 2022) | Greece | To investigate the effects of a nutritional  intervention based on the Mediterranean diet on FEP patients receiving 2 categories of APs.  (Analysis of interest: baseline data of the whole sample) | 6-month pre-post study | Outpatient | **Sample size: n=21**  **Study population:**  FEP (psychotic episode for the first time, within 6 months) patients, n (%): schizophrenia 10 (47.62), chronic delusional disorder 1 (4.76), unspecified non-organic psychosis 9 (42.86), bipolar disorder with a recent manic episode with psychotic symptoms 1 (4.76)  Diagnostic criteria: ICD-10, clinician-diagnosed  **Age (years), mean**: 35.9 (10.2)  **Sex, male n (%):** 13 (61.90)  **Baseline BMI (kg/m^2^), mean (SD):** 26.6 (4.89) | 11 (52.38) receiving ‘APs with a lower risk of weight gain and metabolic complications (i.e., aripiprazole, amisulpride,  quetiapine, paliperidone, ziprasidone); 10 (47.62) receiving ‘APs with higher risk of weight gain and metabolic  complications (i.e., olanzapine, asenapine, clozapine, and risperidone).’ | Not explained | **Follow-up:** 6 months  (Analysis of interest: baseline data of the whole sample) | 1. **Food intake:**   Dietician-administered dietary habits questionnaire; Mediterranean diet score (MedDietScore) questionnaire (to assess adherence to Mediterranean diet); 24-hour dietary intake recall (to assess daily energy and macronutrient intake). | Non- pharmaceutical funding.  No conflicts of interest | Median (minimum, maximum) MetDietScore 32.0 (22.0, 37.0)  Energy (kcal), mean (SD): 1730 (504)  Carbohydrates (g), mean (SD): 193 (71.7)  Proteins (g), mean (SD): 55.6 (24.1)  Fats (g), mean (SD): 83.3 (31.7) |
| (Costa E Silva et al., 2001) | Multicentre (Latin American  Institutions: Argentina, Brazil, Chile, Colombia, Peru, Venezuela) | To assess the effects of switching schizophrenia patients with haloperidol-induced EPS from haloperidol  to olanzapine. | 7-week prospective study | Inpatient, outpatient | **Sample size: n= 94 (83 completers)**  **Study population:**  Patients with schizophrenia, schizophreniform disorder, or schizoaffective disorder (numbers: NR) who were taking haloperidol and had EPS  Diagnostic criteria: ICD-10.  **Age (years), mean**:  31.8 (8.3)  **Sex, male n (%):** 73 (77.7)  **Baseline BMI (kg/m^2^), mean (SD):** NR | Haloperidol lead-in phase: 5-9 days (mean (SD) dose: 12.7 (7.6) mg/day)  Olanzapine treatment: 6 weeks (mean (SD) dose: 11.6 (3.4) mg/day).  **Duration of prior haloperidol treatment in years, mean:**  2.82 | Not explained. | **Follow-up:** 7 weeks  **Loss to follow-up:** 11 | 1. **Appetite:**   spontaneously reported  treatment-emergent adverse event. Adverse events were defined by the authors as any event that worsened from  baseline or first appeared during treatment. | Pharmaceutical company  Conflicts of interest | N (%) of participants who reported an increase in appetite: 14/94 (14.89). |
| (Huang et al., 2020) | China | to evaluate the association between increase in appetite and olanzapine-induced weight changes and accompanying metabolic effects in drug-naïve first-episode patients with schizophrenia. | 12-week pre-post study | Inpatient | **Sample size: n= 31**  **Study population:**  AP naïve/first episode schizophrenia patients  Diagnostic criteria: DSM-5  Reported baseline data for 33 participants:  **Age (years), mean (range)**: 23.5 (18-36)  **Sex, male n (%):** 12 (36.4)  **Baseline BMI (kg/m^2^), mean (SD):** 21.3 (1.7) | Olanzapine 31 | Not explained | **Follow-up:** 12 weeks **Loss to follow-up:** 2 dropped out after 4 weeks because of increased appetite; not included in the analysis. | **1. Proportion of patients who had increased appetite and duration between olanzapine treatment and increased appetite.** Appetite was assessed daily, 30 minutes before lunch, using 4 standardised, physician-rated questions: Hungry, felt full, thinking about food, and overeating. | Non-pharmaceutical  Conflicts: not stated | N (%) of AP-treated participants who reported an increase in appetite from baseline after a 12-week olanzapine treatment: 24/31 (77.4) |
| (Mathews et al., 2012) | USA | ‘To test the hypothesis that olanzapine treatment disrupts the neural activity associated with the anticipation and receipt (consumption) of food rewards (chocolate milk and tomato juice).' | 7-day pre-post study | Lab-based | **Sample size: n= 19**  **Study population:**  Healthy volunteers  **Age (years), mean**:  27.5 (5.9) **Sex, male n (%):**10 (52.6)  **Ethnicity, n (%):** White 14 (73.7), African American 2 (10.5), Hispanic 2 (10.5), > 1 Race 1 (5.3)  **Baseline BMI (kg/m^2^), mean (SD):**  25.78 (4.82) | Olanzapine 19 | Convenience | **Follow-up:** 7 days  **Loss to follow-up:** 25 participants consented to take part, 6 dropped out of the study, and were not included in the analysis. | **1. Eating behaviour:** Measured using the TFEQ. | Non-pharmaceutical  Conflicts of interest |  |
| (Garriga et al., 2019) | Spain | to investigate specific food cravings and subsequent food consumption in patients starting clozapine, and their possible relation to weight and BMI. | 18-week prospective study | Community/  outpatient | **Sample size: n= 34**  Normal weight (NW): n= 13; Overweight/obese (OWO): n= 21  **Study population, n (%):**  Schizophrenia 27 (79.40), schizoaffective 5 (14.70), bipolar disorder 2 (5.90)  (N.B.: overall sample had clinical indication to start clozapine treatment)  Diagnostic criteria: DSMIV-TR  **Age (years), mean (SD):**  NW: 34.50 (11.40)  OWO: 38.20 (12.80)  **Sex, male n (%):**  NW: 7 (53.90)  OWO: 14 (66.70)  **Baseline BMI (kg/m^2^), mean (SD):**  OWO: 30.3 0 (3.70)  NW: 22.00 (2.70) | Clozapine 34  **Clozapine dose:** start dose 12.5-25 mg on 1^st^ day,  followed by weekly upward adjustments  of 25-50 mg, until satisfactory symptom control or major side effects.  **Duration of prior AP treatment in years, mean (SD):**  OWO: 10.9 (11.0); n=21/21 received SGA  NW: 5.2 (7.6); received FGA n=3 (23.1%); SGA n=7 (53.8%), none n=3 (23.1%). | Consecutive | **Follow-up:** 18 weeks  **Loss to follow-up:** 4 did not complete the 18-week visit. | **1. Total and specific food cravings**:  Measured using the Food Craving Inventory-Spanish version (FCI-SP), Cuestionario de Frecuencia de Consumo de Alimentos (CFCA). | Non-pharmaceutical  Conflicts of interest |  |
| (Gothelf et al., 2002) | Israel | to examine caloric intake and energy expenditure as possible contributors to olanzapine-induced weight gain in adolescents with schizophrenia. | 4-week prospective study  (Analysis of interest: analysis of data collected before and after treatment in the olanzapine-treated participants) | Inpatient | **Sample size: n= 10**  **Study population:**  Schizophrenia  Diagnostic criteria: DSM-IV, clinician-administered  **Age (years), mean (SD):**  17.0 (1.6)  **Sex, male n (%):** 10 (100)  **Baseline BMI (kg/m^2^), mean (SD):**  24.5 (5.9) | Olanzapine 10 **Mean (SD) daily dose of olanzapine dose:** 14.0 (4.1) mg/day  **Prior AP treatment, n:** drug naïve n=1, clomipramine n=1, AP medication other than olanzapine n=8.  Participants were excluded if they had received other medications that affect weight (lithium, antidepressant or valproate).  **Mean (SD) washout period:** 17.6 days (27.8) | Not explained | **Follow-up:** 4 weeks | **Food intake**:  A clinical dietician closely monitored food intake for 2 consecutive days. All food products and beverages consumed were weighed before and after meals.  Total daily caloric intake, carbohydrate, fat, and protein contents, were calculated using computerised food tables (Dietary Evaluation for Windows, Jerusalem). | Non-pharmaceutical  Conflicts of interest: not stated |  |
| (Saugo et al., 2020) | Italy | To investigate the dietary habits of a sample of FEP patients treated in community settings. | 9-month prospective study  (Analysis of interest:  cross-sectional analysis of food intake data collected from GET UP patients (at the follow-up visit) and compared with data collected from a nationally representative sample of the Italian population (INRAN-SCAI) 2005-2006). | Community/  outpatient | **Sample size: n=**  **2367**  Exposed group: n= 54; Unexposed group: n= 2313  **Exposed group:**  Subgroup of GET UP study (Ruggeri et al., 2015) participants (patients with FEP), >75% non-affective psychosis.  Diagnostic criteria: ICD-10, clinician diagnosis.  Sociodemographic characteristics of analytical sample (n=54): NR.  Age (years), range: 18-54  Sex, male n (%): 33 (61.1)  Baseline BMI (kg/m^2^) of AP-treated females: 21.40 (2.10); AP-treated males: 24.50 (4.40)  **Unexposed group:** Subsample of the Italian National Food Consumption Survey (INRAN-SCAI) 2005-06 sample aged 18-64.90 years (Leclercq et al., 2009). This is a nationally representative sample of the Italian general population (Leclercq et al., 2009).  Age (years), range: 18-64.90  Sex, male n (%): 1068/2313 (46.17)  Baseline BMI (kg/m^2^) of female unexposed group: 23.20 (3.80), male unexposed group: 25.40 (3.40). | **Exposed group:**  Females:  FGA: n=6; SGA: n=14; both: n=1  Males:  FGA: n=5; SGA: n=25; both: n= 3 | Exposed group: consecutive sample of eligible patients recruited within one participating unit in the GET UP study  Unexposed group: muti-stage probability sampling | None-response: Of 96 AP-treated participants, 42 did not complete the  EPIC due to refusal or unavailability of treating clinician. | **1. Food intake:**  Measured in exposed group using the European Prospective Investigation into Cancer and Nutrition (EPIC) Questionnaire at the follow-up visit; clinician-administered.  Measured in unexposed group using self-report of food consumption for 3 consecutive days (Leclercq et al., 2009). | Non-pharmaceutical funding.  Conflicts: not stated |  |
| (Treuer et al., 2009) | Multi-centre (China, Mexico, Romania, Taiwan) | to explore factors associated with weight gain in patients initiating or switching to olanzapine treatment for schizophrenia or bipolar mania. | 6-month prospective study | Community/  outpatient | **Sample size: n= 622**  (analytical sample size was unclear; 589 completers)  **Study population, n (%):**  Schizophrenia 529 (85.0), bipolar disorder (mania) 93 (15)  Diagnostic criteria: DSM-IV-TR or ICD-10  **Age (years), mean (SD):** 32.6 (12.2)  **Sex, male n (%):** 273 (44)  **Country, n (%):** China 330 (53.1), Romania 151 (24.3), Mexico 91 (14.60), Taiwan 50 (8.0).  **Baseline BMI (kg/m^2^), mean (SD):** 23.2 (3.9)  Of 622 participants initially enrolled in the original study, 463 (74.50%) patients had received prior AP therapy before study entry.  Participants who had been participating in a weight control programme (WCP) at study entry, n (%): 147/622 (23.6). Of the remaining 475 patients, 99 (20.8%) started a WCP after study entry visit and during olanzapine therapy.  During the study, 68 (11%) of the patients at each visit had taken other APs in addition to olanzapine since the previous visit (SGA, n=35 (5.70%) and FGA, n=33 (5.30%); and 105 (17.00%) of the patients had taken mood stabilizers (valproate, carbamazepine). | Olanzapine 622 | Not explained | **Follow-up:** 6 months **Loss to follow-up:**  71. | **1) Changes in appetite, hunger, fullness and eating behaviours:** Measured using the Eating Attitude Scale (EAS)**.**  **2) Frequency of consumption of specific food groups:** Physician-rated.  **3) Meal regularity:** average number of meals consumed each day, snack consumption at certain times of the day. | Pharmaceutical company  Conflicts of interest | N (%) of participants who reported: Stronger appetite than usual after 6-month olanzapine treatment: 288/589 (48.9);  felt they required a much larger amount of food to feel sufficiently full (relative to baseline) after 6-month olanzapine treatment: 204/589 (34.7) |
| (Case et al., 2010)  Study 4 (Treuer et al., 2009) |  |  |  |  |  |  |  | This secondary analysis included weight and appetite data collected at baseline and 4 weeks. | **1) Changes in appetite, hunger, fullness and eating behaviours:** Measured using the EAS**.** |  |  |
| (Abbas and Liddle, 2013) | UK | To compare food cravings in patients treated with olanzapine, patients treated with typical antipsychotics, and in healthy participants. | Cross-sectional | 2 exposed groups: inpatient, outpatient  Unexposed group: community | **Sample size: n= 60**  Olanzapine group, n=20;  FGA group, n= 20  unexposed group, n= 20  **Olanzapine group:**  Study population: schizophrenia  Diagnostic criteria: ICD-10 revision criteria, clinician diagnosis. Age (years), mean (SD): 39.4 (11.5)  Sex, male n (%):11 (55)  Baseline BMI (Kg/m2), mean (SD): 29.5 (5.4)  **FGA group:**  Study population: schizophrenia  Diagnostic criteria: ICD-10 revision criteria, clinician diagnosis. Age (years), mean (SD): 39.4 (12.0)  Sex, male n (%): 9 (45)  Baseline BMI (kg/m^2^), mean (SD): 27.3 (5.4)  **Unexposed group:** healthy participants  Age (years), mean (SD): 40.9 (11.0)  Sex, male n (%):9 (45).  Baseline BMI (kg/m^2^), mean (SD): 25.8 (5.0). | Olanzapine 20,  FGA 20,  **Olanzapine group:**  Duration of AP treatment in months, mean (SD): 15.1 (19).  Use of other medications (SSRI, TCA, mirtazapine, venlafaxine), n=8.  **FGA group:**  Duration of AP treatment in months, mean (SD): 19.7 (11).  Use of other medications (SSRI, TCA, mirtazapine, venlafaxine), n= 7 | Sampling strategy not explained; sample size calculation provided | NA | **Food craving:** Measured using the FCI. | Funding: not stated  Conflicts: none | Craving scores of those who were taking olanzapine for < 4 months (7 patients) were compared with those who were taking it for > 6 months (13 patients) and with controls with ‘no significant differences’. |
| (Archie et al., 2007) | Canada | To investigate the dietary patterns of patients with a psychotic disorder | Cross-sectional | Exposed group: Community/  Outpatient  Unexposed group: Community | **Sample size: n= 309**  Exposed group: n= 101 Unexposed group, n= 208  **Exposed group:**  Study population, n (%):  Schizophrenia 46 (45.54), schizoaffective disorder 25 (24.75), delusional disorder 4 (3.96), bipolar disorder 8 (7.92), depression with psychotic features 7 (6.93), FEP 11 (10.89).  Diagnostic criteria: DSM-IV  Age (years), mean (SD): 35.00 (10.50)  Sex, male n (%):64 (63.40)  Ethnicity, n (%): Aboriginal 1 (0.99), Black 4 (3.96), Filipino 1 (0.99), Japanese 1 (0.99), Latin American 3 (2.97), South Asian 4 (2.97), South East Asian 1 (0.99), West Asian 1 (0.99), White 85 (84.16)  Baseline BMI (kg/m^2^), mean (SD) [for 99/101 participants]: 28.88 (6.16)**  **Unexposed group:**  A sample of employees in a company in San Francisco Bay, USA (Block et al., 2000)  Age (years), range: 20 to 69 years  Sex, male n (%): 74 (35.60)  Ethnicity, n (%): Hispanic 15 (7.21); White, not Hispanic 135 (64.90); African American 7 (3.37); Asian, Pacific Islander 45 (21.63); Missing/not specified 6 (2.88).  Baseline BMI (kg/m^2^), mean (SD): NR | Risperidone: n= 29; olanzapine: n= 26; clozapine: n= 16; combination: n=15; FGA: n=15  **Duration of AP treatment:** NR.  (FEP: n=11/101) | Convenience | NA | **1. Food intake:** Measured using the Dietary Fat Screener and the Fruit and Vegetable and Fibre Screener. | Funding: not stated  Conflicts: none |  |
| (Blouin et al., 2008) | Canada | To investigate eating behaviours in patients treated with a SGA and compare them with those of a sample of healthy participants. | Cross-sectional | SGA-treated group: Outpatient  Unexposed group: community  (Assessments were conducted in a lab setting) | **Sample size: n= 38**  Exposed group: n=18 Unexposed group: n= 20  **Exposed group:**  Study population, n (%):  Schizophrenia or other related psychoses, treated with a single SGA for at least 3 months.  Schizophrenia 15 (83.33), schizoaffective disorders 2 (11.11), delusional disorder 1 (5.56).  Diagnostic criteria: DSM-IV, clinician diagnosis  Age (years), mean (SD): 30.5 (7.9)  Sex, male n (%): 18 (100)  Baseline BMI (kg/m^2^), mean (SD): 28.8 (5.0)  Patients had to be sedentary(<30 minutes of physical activity/week)  **Unexposed group:**  Study population: healthy men taking no regular medication (matched to the SGA-treated group for age, weekly physical activity levels)  Age (years), mean (SD): 29.5 (6.7)  Sex, male n (%): 20 (100)  Baseline BMI (kg/m2), mean (SD): 25.0 (3.3) | SGAs including clozapine: n = 2; olanzapine:  n = 9; risperidone: n = 2; quetiapine: n = 3; ziprasidone: n = 2  **Duration of current SGA treatment** in months, mean (SD): 24.60 (19.70).  **Duration of prior AP exposure (FGA or SGA) in SGA-treated group** months, mean (SD): 35.30 (22.00).  SGA-treated group had to be sedentary (practicing <30 minutes of continuous physical activity/week). | Not explained | NA | 1. **Hunger, satiety quotient (SQ):** Hunger and prospective food consumption were measured before and after a meal using VAS. This information was used to estimate the satiating capacity of food, expressed as SQ. 2. **Macronutrient preference and spontaneous intake**:   Lab setting; food was weighed before and after the buffet.   1. **Eating cognitions:**   Measured using the TFEQ. | Non-pharmaceutical funding  Conflicts: none | Macronutrient intake as % of meal (SD):  Fat intake in SGA-treated group: 33.30 (4.60); unexposed: 33.40 (8.00)  Duration of current SGA treatment was  associated with decreased dietary restraint (r = -0.5; p= 0.03); strategic dieting behaviour (r =  -0.52; p= 0.03); decreased emotional susceptibility to  disinhibition (r= -0.62; p= 0.006). |
| (de Beaurepaire, 2021) | France | To investigate the prevalence of eating disorders in patients with schizophrenia or schizoaffective disorder chronically treated with an AP monotherapy. | Cross-sectional | Community/  outpatient | **Sample size: n= 156**  **Study population:**  Schizophrenia or schizoaffective disorder treated with AP monotherapy for ≤8 years (numbers: NR)  **Age (years), mean:** 41.7  **Sex, male n (%):** 88 (56.41)  **Baseline BMI (kg/m^2^), mean (SD):** NR | Clozapine 15; olanzapine 33; risperidone 35; aripiprazole 24; haloperidol 27; amisulpride 7; zuclopenthixol 8; fluphenazine 3; pipotiazine 4. | Retrospective study: All eligible participants were included in the analysis (consecutive) | NA | **1. Eating disorders:**  Binge eating disorder was diagnosed as either syndromal (fulfilling all DSM-IV criteria) or subsyndromal (partially fulfilling DSM-IV criteria). Night eating was evaluated using the following question: “Do you get up at night to eat?” | Non-pharmaceutical  funding  Conflicts: none | N (%) of participants with:  Bulimia nervosa= 0; syndromal BED: 7/156 (4.4); subsyndromal BED: 9/156 (18.7); night eating: 47/156 (30.13).  OR of eating disorders in those receiving AP for 2-8 years versus those receiving AP for 2 years (reference group), OR [95% CI]:  BED: 0.86 [0.44-1.71]; night eating 0.86 [0.44-1.71] |
| (Henderson et al., 2006) | USA | To evaluate the dietary intake of patients with schizophrenia or schizoaffective disorder treated with SGAs, and compare them with a subsample of the National Health and Nutrition Examination Survey (NHANES) 1999-2000. | Cross-sectional | SGA-treated group: Community/  Outpatient  Unexposed group: community | **Sample size: n= 811**  SGA-treated group: n= 88  Unexposed group (NHANES 1999-2000): n= 723  **Exposed group:**  Study population, n (%):  Schizophrenia 65 (73.86), schizoaffective disorders 23 (26.14) Diagnostic criteria: NR  Age (years), mean (SD): 45 (10)  Sex, male n (%): 63 (71.6)  Ethnicity, n (%):Caucasian 62 (70.5), African descent 24 (27.3), Hispanic 1 (0.01), Other 1 (0.01)  Baseline BMI (kg/m^2^), mean (SD): 31.3 (12.7)  **Unexposed group:**  Study population: subsample of the (NHANES 1999-2000, a nationally representative sample of civilian, non-institutionalised adults in the USA (matched to the SGA-treated group for age, gender and ethnicity).  Age (years), mean (SD): 44 (19)  Sex, male n (%):503 (69.6%)  Ethnicity, n (%):Caucasian 507 (70.1), African descent 194 (26.8), Hispanic 17 (2.4), Other 5 (0.7)  Baseline BMI (kg/m^2^), mean (SD): 28.3 (6.6) | SGA-treated group: olanzapine 42, clozapine 25, risperidone 15, quetiapine 3, ziprasidone 1, med-free 2  **Duration of treatment in SGA-treated group:** NR. | SGA-treated group: not explained  Unexposed group (NHANES 1999-2000): stratified, multistage probability sample | NA | **1. Food intake:** SGA-treated group: measured using the self-rated four-day dietary record (on 3 week days and 1 weekend day) and the Block Food Frequency Questionnaire (FFQ; researcher-administered).   Unexposed group (NHANES, 1999-2000): measured using the 24-hour dietary recall method. | Non-pharmaceutical funding  Conflicts: not stated | Subgroup of SGA-treated group that completed FFQ (n =43): % of calories from sweets was 19.3 (SD = 12.6) ‘indicating a large consumption of cake, cookies, candy and sweetened beverages'. |
| (Henderson et al., 2010) | USA | To investigate the relationship between clozapine and risperidone with glucose and lipid metabolism and dietary fat intake in patients with schizophrenia. | Cross sectional | Community/  outpatient | **Sample size: n= 46**  Clozapine-treated group: 31; Risperidone-treated group: 15  **Clozapine group:**  Study population: schizophrenia  Diagnostic criteria: DSM-IV-TR, clinician diagnosis. Age (years), mean (SD): 41 (9)  Sex, male n (%): 24 (77)  Ethnicity, n (%):Caucasian 28 (90), African American 2 (7), Hispanic 1 (3)  Baseline BMI (kg/m^2^), mean (SD): 27.3 (4.6)  **Risperidone group:**  Study population: schizophrenia  Diagnostic criteria: DSM-  IV-TR, clinician diagnosis. Age (years), mean (SD): 44 (10)  Sex, male n (%): 10 (67)  Ethnicity, n (%): Caucasian 10 (67), African American 5 (33)  Baseline BMI (kg/m^2^), mean (SD): 25.7 (2.8) | Clozapine 31, risperidone 15 | Not explained | NA | **1. Food intake:**  Measured using a 4-day food record. | Non-pharmaceutical funding  Conflicts of interest | Comparison of food intake in clozapine- and risperidone-treated groups (after controlling for: age, race, gender, BMI, duration of illness, family history of diabetes):  Energy intake, mean (SD): clozapine: 2092 (1009), risperidone: 1923 (557), p = 0.469  Carbohydrate (% total energy), mean (SD): clozapine: 48.8 (7.5), risperidone: 55.7 (9.5) p= 0.009  Protein (% total energy), mean (SD):  clozapine: 16.2 (3.9), risperidone: 13.8 (3.4); p=0.036  Fat (% of total energy), mean (SD): clozapine: 36.0 (6.7), risperidone: 30.9 (5.7), p=0.013 |
| (Jakobsen et al., 2018b) | Denmark | To investigate the dietary habits of overweight people with schizophrenia spectrum disorders and to compare their dietary habits with those from the general Danish population. | Cross-sectional | Exposed group: community/  Outpatient  Unexposed group: community | **Sample size: n=** **3362**  Exposed group: n= 346 Unexposed group: n= 3016  **Exposed group:**  Baseline data of 346/428 CHANGE trial participants (schizophrenia spectrum disorders and increased waist circumference i.e., > 88 cm for women and 102 cm for men) used in analysis of interest.  Diagnostic criteria: ICD-10 (Speyer et al., 2016)  Age (years), mean (SD): NR  Sex, male n (%): NR  Baseline BMI (kg/m^2^), mean (SD): NR  **Unexposed group:**  Study population:  Danish National Survey of Dietary Habits and Physical Activity (DANSDA) 2011-2013, a nationally representative sample of individuals aged 4-75 years old. Age (years), mean (SD): NR  Sex, male n (%): NR  Baseline BMI (kg/m^2^), mean (SD): NR | SGAs (n= 346) including olanzapine, clozapine, quetiapine  **Duration of prior AP treatment** in SGA-treated group: NR.  **Use of other medications:** NR | Exposed group: baseline data of the CHANGE trial participants (a 3-arm superiority randomised trial)  Unexposed group (DANSDA): not stated | NA | **1. Food intake:**  Measured in cases using 24-hour recall and a FFQ.  Measured in controls using a food record for 7 consecutive days in controls. | Non-pharmaceutical funding  No conflicts of interest | Macronutrient intake as % of energy (SD): Fat intake in SGA-treated group: 33.9 (10.6); unexposed: 37.0 (5.4) |
| (Khazaal et al., 2006a) | Switzerland | To compare binge eating symptomatology in a group of individuals receiving treatment for schizophrenia with a group of non-psychiatric controls. | Cross-sectional | SGA-treated group:  Outpatient  Unexposed group: community | **Sample size: n= 80** (stratified according to BMI).  Exposed group: 40 (20 with BMI <28; 20 with BMI ≥28); Unexposed group: 40 (20 with BMI <28; 20 with BMI ≥28)  **Exposed group:**  Study population: schizophrenia  Diagnostic criteria: DSM-IV  Age (years), mean (SD): cases with BMI <28: 31.7 (9.2), cases with BMI ≥28: 36.1 (8.9)  Sex, male n (%): cases with BMI <28: 11/20 (55), cases with BMI ≥28: 10/20 (50)  Baseline BMI (kg/m^2^), mean (SD):  cases with BMI <28:23.6 (2.2)  cases with BMI ≥28: 32.9 (6.1)  **Unexposed group:**  Study population: clinic workers (stratified according to BMI)  Age (years), mean (SD): participants with BMI <28: 33.9 (13.0), participants with BMI ≥28: 37.2( 8.1)  Sex, male n (%): participants with BMI <28: 7/20 (35), participants with BMI ≥28: 12 (60)  Baseline BMI (kg/m^2^), mean (SD):  participants with BMI <28: 21.1 (2.5), participants with BMI ≥28: 33.8 (4.9) | SGAs including olanzapine, clozapine, quetiapine, risperidone.  All patients in the SGA-treated group (n=40):  **duration of treatment** with SGA in years, mean (SD): 8.30 (6.20)  Use of other treatments: most patients were taking ≥1  comedications (alprazolam, atorvastatine,  clorazepate, citalopram, esomeprazole, flurazepam,  lorazepam, macrogolum, propranolol). | Convenience sample.  Both SGA-treated and unexposed samples were stratified according to BMI into 2 subgroups: participants with BMI > 28 and those BMI < 28. | NA | **1. Binge eating status:** based on DSM-IV criteria.  Classified as having (1) no binge eating, (2) binge episodes < 2 days/week (BS), (3) BED or (4) bulimia nervosa (BN). | Funding: not stated  Conflict: not stated |  |
| (Khazaal et al., 2006b) |  | Assess cognitions associated with restrained eating in severely overweight SGA-treated patients with schizophrenia. |  |  |  |  |  |  | **1. Eating cognitions:**  Measured using the Revised version of the Mizes Anorectic Cognitions questionnaire (MAC-R) |  |  |
| (Khazaal et al., 2009) | Switzerland | To explore differences in hunger, negative alliesthesia and eating cognitions between patients treated with SGAs and in non-psychiatric participants. | Cross-sectional | Unclear | **Sample size: n= 37**  Exposed group: 22 (10 with weight gain; 12 without weight gain); Unexposed group: 15 (5 with weight gain; 10 without weight gain)  **Exposed group:**  Study population: male patients with schizophrenia, treated with SGA (stratified according to weight gain).  Diagnostic criteria: DSM-IV, clinician diagnosis  Age (years), mean (SD): cases with weight gain: 31.3 (8.3), cases without weight gain: 31.8 (10.2)  Sex, male n (%): 22 (100%)  Baseline BMI (kg/m^2^), mean (SD): cases with weight gain: 28.6 (4.2), cases without weight gain: 24 (5.6)  **Unexposed group:**  Study population: male health professionals and university students (stratified according to weight gain).  Age (years), mean (SD): participants with weight gain: 32.8 (8.9), participants without weight gain: 25.4 (3.7)  Sex, male n (%): 15 (100)  Baseline BMI (kg/m^2^), mean (SD): participants with weight gain: 29.2 (4.3), participants without weight gain: 22.5 (1.7) | SGAs including olanzapine 14, clozapine 2, risperidone 2, quetiapine 4 | Convenience  Both SGA-treated and unexposed samples were stratified according to weight gain in the last month into 2 subgroups: participants with clearly documented weight gain (minimum 2 kg in the last month), and participants with persistent stable weight (neither gained nor lost >1 kg in the last month). | NA | **1. Eating cognitions:** Measured using the TFEQ. | Funding: not stated  Conflicts: not stated |  |
| (Khosravi, 2020) | Iran | To investigate factors associated with disordered eating behaviours (DEB) among patients with schizophrenia | Cross-sectional | Not stated | **Sample size: n= 308**  Exposed group: 154,  Unexposed group: 154  **Exposed group:**  Study population: Schizophrenia.  Diagnostic criteria: DSM-5, clinician diagnosis  Age (years), range: 20-60  Sex, male n (%): 64 (41.56) Baseline BMI (kg/m^2^), mean (SD): NR  **Unexposed group:**  Study population: Healthy volunteers from same geographical area (1:1 matching)  Age (years), range: 20-60  Sex, male n (%): 84 (54.55)  Baseline BMI (kg/m^2^), mean (SD): NR | FGAs, SGAs (n= 154) (no further details provided)  **Duration of AP treatment:** NR  Use of antidepressants or mood stabilizers in previous 3 months was an exclusion criterion. | Convenience | NA | **1. Disordered eating behaviours (DEB):** Measured using the Persian version of the Eating Attitude Test (EAT-26). | Funding: none  Conflicts: none | Among patients with schizophrenia who had DEBs (n=64), SGAs were positively correlated with EAT-26 score compared to FGAs (adj. for psychosocial rehabilitation, duration of psychosis, anxiety, depression, severity of psychosis, tobacco smoking, T2DM).  Regression coefficient: 2.78, 95% CI (0.06-5.49), p-value <0.05 |
| (Kouidrat et al., 2018) | France | To investigate the eating behaviours, clinical and biological data of a sample of schizophrenia patients compared to values of healthy participants. | Cross-sectional | Outpatient | **Sample size: n= 147**  Exposed group: 66; Unexposed group: 81  **Exposed group:**  Study population: Schizophrenia or schizoaffective disorder (numbers: NR).  Diagnostic criteria: DSM-IV  Age (years), mean (SD): 44 (11)  Sex, male n (%): 43 (65.15)  Baseline BMI (kg/m^2^), mean (SD): 30.3 (8.2)  **Unexposed group:**  Study population: Participants recruited from the local university and local community.  Age (years), mean (SD): 32 (14)  Sex, male n (%): 24 (29.63)  Baseline BMI (kg/m^2^), mean (SD): 24 (3.3) | 62/66 (93.94%) of exposed group received APs.  APs, n (%): FGA 25 (37.88), SGA 20 (30.30), both 17 (25.76)  **Duration of prior AP treatment:** NR  Use of other medications, n (%): antidepressants 40 (60.61). | Exposed group: consecutive sample.  Unexposed group: convenience sample. | NA | **1. Eating cognitions:** Measured using the Three-Factor Eating Questionnaire-Revised 21-item Version (TFEQ-R21). | Funding: not stated  Conflicts: not stated |  |
| (Nunes et al., 2014) | Brazil | To investigate food consumption patterns patients with schizophrenia. | Cross-sectional | Outpatient | **Sample size: n= 50**  Exposed group: 25; Unexposed group: 25  **Exposed group:**  Study population: Schizophrenia  Diagnostic criteria: ICD-10  Age (years), mean (SD): 40.5 (9.2)  Sex, male n (%):15 (60)  Baseline BMI (kg/m^2^), mean (SD): 29.09 (6.30)  **Unexposed group:**  Study population: Healthy volunteers  Age (years), mean (SD): 37.2 (12.4)  Sex, male n (%): 13 (52)  Baseline BMI (kg/m^2^), mean (SD): 26.91 (4.39)  (Matched to cases by age, sex, BMI) | FGA, n= 7 (28), SGA, n= 17(68); both, n=1 (4).  **Duration of AP treatment:** NR.  Use of other medications: NR | Exposed group: consecutive sample.  Unexposed group: convenience sample. | NA | 1. **Food intake:**   Measured using a researcher-administered FFQ. | Non-pharmaceutical funding  No conflicts of interest |  |
| (Sentissi et al., 2009) | France | To investigate the association between different antipsychotic medications and food attitudes. | Cross-sectional | Inpatient, outpatient | **Sample size: n= 153**  FGA-treated group: 27  SGA-treated group: 93  Untreated group: 33  **Study population:**  Schizophrenia  Diagnostic criteria: DSM-IV  **Age (years), mean (SD):** 33.1 (8.7)  **Sex, male n (%):** 94 (61.44)  **Baseline BMI (kg/m^2^), mean (SD):** 25.6 (5.5) | 120/153 (78.43) of participants received APs, n: clozapine 20, olanzapine 23, amisulpride 14, risperidone 20, aripiprazole 16, FGAs 27 (mainly butyrophenon haloperidol or phenothiazines)  **Duration of AP treatment in months, mean (SD):**  FGA- and SGA-treated groups: 36.2 (49.7)  Untreated group: AP-naive (n=23) or without treatment for at least 3 months. | Consecutive | NA | **1. Eating cognitions:** Measured using the TFEQ and the Dutch Eating Behavior Questionnaire (DEBQ). | Non-pharmaceutical funding  Conflicts: none | **TFEQ restriction scores, mean (SD):**  untreated: 8.5 (4.53); SGA: 8.7 (4.7); FGA: 7.6 (4.9)  **TEFQ disinhibition scores, mean (SD):** untreated: 6.3 (3.9); SGA: 6.1 (3.6); FGA 4.5 (2.8)  **TFEQ hunger scores, mean (SD):** untreated: 6.0 (3.8), SGA: 5.7 (3.1), FGA: 4.8 (3.3)  **DEBQ restriction scores, mean (SD):** untreated 2.5 (0.8), SGA 2.5 (0.8), FGA 2.3 (0.6)  **DEBQ emotional scores, mean (SD):** untreated 2.6 (0.7), SGA 2.7 (0.6), FGA 2.5 (0.6)  **DEBQ external scores, mean (SD):** untreated 2.5 (0.7), SAGA 2.5 (0.6), FGA 2.2 (0.5) |
| (Stefanska et al., 2017) | Poland | To assess the nutritional value of food intake in female and male patients with schizophrenia (analysis of interest). | Cross-sectional | Outpatient | **Sample size: n= 158**  Exposed group: 60;  Unexposed group: 98  **Exposed group:**  Study population: Schizophrenia  Diagnostic criteria: ICD-10  Age (years), mean (SD): 37.94 (11.05)*  Sex, male n (%): 28 (46.67)  Baseline BMI (kg/m^2^), mean (SD) of females in exposed group: 27.2 (5.7), of males in exposed group: 27.6 (5.9)  **Unexposed group:**  Study population: Healthy volunteers  Age (years), mean (SD): 39.12 (13.33)*  Sex, male n (%): 38 (38.78)  Baseline BMI (kg/m^2^), mean (SD) of females in unexposed group: 25.8 (5.2), of males in unexposed group: 27.3 (5.7) | FGAs or SGAs: Most commonly prescribed (no numbers provided) were olanzapine,  risperidone, haloperidol, clozapine.  Females, n (%): one AP 15 (47), ≥2 APs 17 (53)  Males, n (%): one AP 10 (36),  ≥2 APs 18 (64)  **Duration of prior AP treatment:** ≥1 year | Not explained | NA | 1. **Food intake:**   Measured using 24-hour diet recall from 3 weekdays. | Non- pharmaceutical funding  Conflicts: none | Macronutrient intake as % of energy: see Supplementary Material |
| (Stefanska et al., 2018) | Poland | To assess the  nutritional habits of people with  schizophrenia. | Cross-sectional | Outpatient | **Sample size: n= 155**  Exposed group: 85;  Unexposed group: 70  **Exposed group:**  Study population: Schizophrenia  Diagnostic criteria: ICD-10  Age (years), mean (SD): 38.44 (9.29)*  Sex, male n (%):30 (42.86)  Baseline BMI (kg/m^2^), mean (SD) of females in exposed group: 25.1 (5.3), of males in exposed group: 25.0 (4.6).  **Unexposed group:**  Study population: Healthy volunteers  Age (years), mean (SD): 37.21 (10.99)*  Sex, male n (%):40 (47.06)  Baseline BMI (kg/m^2^), mean (SD) of females in unexposed group: 24.4 (5.0), of males in unexposed group: 25.9 (4.2). | FGAs or SGAs: Most commonly prescribed (no numbers provided) were olanzapine, risperidone, haloperidol, clozapine.  33/85 (39%) received 1 AP;  52/85 (61%) received 2 or 3 APs.  **Duration of prior AP treatment:** ≥1 year. | Not explained | NA | 1. **Food intake:**   Measured using 24-hour diet recall from 3 weekdays and 1 weekend day. | Non- pharmaceutical funding  Conflicts: none |  |
| **Quantitative descriptive studies** | | | | | | | | | | | |
| (Goluza et al., 2017) | Australia | To examine the prevalence of food addiction (FA), associations between FA and participant characteristics in a sample of outpatient schizophrenia patients. | Cross-sectional | Outpatient | **Sample size: n= 93**  **Study population:**  Schizophrenia  Diagnostic criteria: NR  **Age (years), range**: 18-55  **Sex, male n (%):** 61 (65.59)  **Baseline BMI (kg/m^2^), mean (SD):** NR | Quetiapine 75, olanzapine 6, clozapine 12  **Duration of prior AP treatment in years, mean (SD):** NR | Convenience | NA | 1. **Food addiction:** Measured using the Yale Food Addiction Scale (YFAS). | Funding: none  Conflicts of interest: NR | N (%) of participants who met diagnostic criteria  for FA= 25 (26.9).  Mean number (SD) of symptoms of FA= 3.74 (1.82).  Commonest FA symptoms: persistent desire/ repeated unsuccessful attempts to cut down intake (97.9%),  tolerance/continued overeating despite physical or psychological-  problems (63.8%), use continues despite knowledge of  adverse consequences (57.5%). |
| (Jakobsen et al., 2018a) | Denmark | to investigate associations between clinical and psychosocial factors and cardiovascular  risk factors in patients with schizophrenia and abdominal obesity enrolled in the  CHANGE trial. | Secondary analysis of baseline data from the CHANGE trial | Community/outpatient | **Sample size: n= 428**  Baseline data of CHANGE trial participants (schizophrenia spectrum disorders and increased waist circumference i.e., > 88 cm for women and 102 cm for men).  **Study population, n (%):**  Schizophrenia 378 (88.30), schizoaffective psychosis 45 (10.50), persistent delusional disorder 5  (1.20).  Diagnostic criteria: ICD-10  **Age (years), mean (SD):** 38.60 (12.40)  **Sex, male n (%):** 191 (44.6%)  **Baseline BMI (kg/m^2^), mean (SD):** 34.20 (6.00) | 408/428 (95.33%) were prescribed APs. 127/428 (29.67%) were on either olanzapine or clozapine and  166 (38.78%) received >1 AP (Jakobsen et al., 2017)  **Chlorpromazine equivalents, mg/day,**  **mean (SD):** 473.50  (397.90)  **Use of antidepressants, n (%):** 187 (43.70) | Baseline data of the CHANGE trial participants (a 3-arm superiority randomised trial) | NA | 1. **Food intake:**   Dietary quality score (DQS) calculated using a FFQ. | Non-pharmaceutical funding  No conflicts of interest | Defined Daily Doses (DDD) of olanzapine, clozapine, quetiapine were positively associated with DQS  (0.10  [95% CI:  -0.08 to  0.28], p=0.28).  Analysis adjusted for baseline age, sex. |
| (Kirkegaard et al., 1982) | Denmark | To investigate serious side effects associated with the long-term clozapine treatment | Cross-sectional | Not stated | **Sample size: n= 17**  **Study population, n (%):**  Patients diagnosed with schizophrenia who were on long-term clozapine treatment.  **Age (years), mean:** 38.2  **Sex, male n (%):** 13 (76.5)  **Baseline BMI (kg/m^2^), mean (SD):** NR | Clozapine 17  **Mean daily dose of clozapine:** 479.8 g  **Duration of prior AP treatment:** 35.9 months | Not explained | NA | **1. Hunger:**  No details provided on outcome assessment. | Funding: not stated  Conflicts: not stated | N (%) of participants who experienced increased hunger:  2/17 (11.8) |
| (Kurpad et al., 2010) | India | To investigate the prevalence of BED among patients on treatment for non-affective psychoses. | Cross-sectional | Community/ outpatient | **Sample size: n= 73**  **Study population, n (%):** Schizophrenia or psychoses not otherwise specified (NOS) (numbers: NR).  Diagnostic criteria: ICD-10  **Age (years):** 17-65  51/73 (69.86%) < 40 years  **Sex, male n (%):**  38 (52.1)  **Baseline BMI (kg/m^2^):**  N (%) of participants with BMI ≥23: 51 (69.9). | 63/73 (86.3%) received APs  Including risperidone: n=45, olanzapine: n=18  **Median duration of AP treatment:** 3 years.  **Use of other medications:** 25/73 received antidepressants | Not explained | NA | **1. Eating behaviours, binge eating, BED:**  Assessed using a questionnaire developed by the authors that checks the presence/absence of DSM-IV criteria for BED.  Binge eating not clearly identified.  **2. Food intake:**  Measured using the 24-hour diet recall and calculated using a commercially available software (Annapurna software, Bangalore, India, 1998). Data not reported. | Funding: not stated  No conflicts of interest | Prevalence of BED: n= 0 |
| (Lappin et al., 2018) | Australia | To assess cardio-metabolic risk factors and their management in a sample of  outpatients treated with clozapine. | Cross-sectional | Outpatient | **Sample size: n= 416**  **Study population, n (%):**  Schizophrenia 364 (87.50) ,  schizoaffective disorder 52 (12.50)  Diagnostic criteria: NR  **Age (years), mean (SD):**  43.70 (11.20)  **Sex, male n (%):**  268 (64.42)  **Ethnicity [n=412], n (%):** Europid/White Australian 273 (66.26), Indigenous Australian 22 (5.34), Asian 51 (12.38), Europid/White Other 62 (15.05), Black African/Black Other 4 (0.97)  **Baseline BMI (kg/m^2^), mean (SD):**  79.6% had BMI ≥25. | Clozapine only: 266/391 (68.03); >1 AP: 125/391 (31.97) [included FGAs: 35, SGAs: 85, both: 5].  **Duration of prior clozapine treatment in years, mean (SD):**  10.4 (4.9)  **Median daily clozapine dose:** 275 mg (range: 25-800) **Use of other medications:** antidepressant 110/391 (28.13), mood stabilizer 63 (16.11). | Consecutive | NA | **Food intake:**  Assessed using a food intake questionnaire that evaluates intake of 6 ‘healthy’ and 4 ‘unhealthy’ food categories. | Non-pharmaceutical funding  No conflicts of interest | Denominators not provided: >50% of participants did not consume fruit, vegetables, wholegrain foods, unsweetened dairy/alternatives, foods with healthy fats daily.  17% of participants reported consuming the following multiple times per day’: 17% fruit, 17% vegetables, 16%  wholegrain foods, 8% unsweetened dairy/alternatives, 18%  foods with healthy fats.  Unhealthy  food intake: 59%, 55%, 54%, 37%  of reported ≥1/day intake of alcohol,  savoury discretionary foods, sweet discretionary foods and sweet  drinks respectively. |
| (Llorca et al., 2017) | USA | To investigate treatment-emergent adverse events (TEAEs) associated with SGAs from both patient and physician perspectives. | Cross-sectional | Community | **Study population:**  **Patients with schizophrenia, n= 17**  **Psychiatrists, n= 4**  **Patients with schizophrenia:**  Study population: Patients diagnosed with schizophrenia, taking ≥ 1 SGAs within the past year, and reporting ≥ 1 TEAEs associated with SGA.  Diagnostic criteria: NR, clinician diagnosis  Age (years), mean (range):45.5 (25–59)  Sex, male n (%): 11 (65)  Ethnicity, n (%): White 9 (53), African American 8 (47), Hispanic/Latino 1 (6), Mixed race 1 (6) (N.B.: numbers do not add up because participants could report >1 ethnic background)  Baseline BMI (kg/m^2^): NR  **Psychiatrists n= 4** | SGAs, n (%): quetiapine 2 (12), aripiprazole 2 (12), risperidone 4 (24), olanzapine 4 (24), lurasidone 2 (12), clozapine 2 (12), ziprasidone 1 (6), paliperidone 1 (6), fluphenazine 1 (6), asenapine 1 (6)  (N.B.: patients received≥ 1 AP) | Convenience | NA | **1.** Frequency, bother, and most bothersome SGA adverse events reported by patients  2. SGA adverse events reported by physicians as clinically important and/or bothersome | Pharmaceutical funding  Conflicts of interest | **Patient results**:  Prevalence of increased appetite and/or weight gain, n (%): 16/17 (94.12)  Psychiatrist results: weight gain was not attributed to medication, but rather to ‘poor eating  habits that were exacerbated during hospitalization. |
| (Morell et al., 2019) | Australia | To assess  the prevalence of metabolic syndrome, other cardio-metabolic risk factors in a sample of participants with severe mental illness (SMI) prescribed long-acting injectable (LAI) APs. | Cross-sectional | Community/outpatient | **Sample size: n= 301**  **Study population, n (%):**  Participants with SMI prescribed LAI [n=300]: schizophrenia 214 (71.3), schizoaffective disorder 63 (21.0), bipolar disorder 23/300 (7.7)  Diagnostic criteria: NR  **Age (years), mean (SD):**  44.4 (12.3)  **Sex, male n (%):**  197 (65.4)  **Country of birth [n= 298], n (%):** Australia 185 (62.1); Asia, Middle East, North Africa, Southern Europe 44 (14.8); other 69 (23.2)  **Baseline BMI (kg/m^2^), mean (SD):**  30.3 (7.9) | **Class of LAI, n (%):**  FGA 121 (40.2),  SGA 180 (59.8)  **Duration of treatment on LAI (years), median (range) [n=181]:** 3.0 (0-36)  **AP polypharmacy [n=301], n (%):**  LAI only 192 (63.8),  ≥1 additional APs to LAI 109 (36.2)  **DDD LAI (mg), median (range) [n=301]:** 1.0 (0.2–2.9)  **Use of other medications, n (%):** antidepressant 29 (9.6), mood stabiliser 56 (18.6) | Consecutive | NA | 1. **Diet quality:**   Assessed using a 10-item,  picture-guided, food intake questionnaire developed to  evaluate food consumption patterns in people with mental illness. | Non-pharmaceutical funding  No conflicts of interest | N (%) of participants who did not consume the following on daily basis:  fruit 172 (61.9), vegetables 167 (60.0), wholegrains  158 (57.4)  N (%) of participants who reported at least daily intake of: sugary  drinks 117 (43),  sweet or savoury discretionary foods 92 (33.5),  alcohol 15 (<6)  (N.B. it is unclear what the denominators for these counts are). |
| (Qurashi et al., 2015) | UK | To assess the effects and overall satisfaction with clozapine in comparison to previously prescribed  APs. | Cross-sectional | Inpatient | **Sample size: n= 56**  **Study population:** patients with schizophrenia prescribed clozapine ≥3 months.  Diagnostic criteria: NR.  **Age (years), mean (SD):**  37.9 (10.6)  **Sex, male n (%):** 56 (100)  **Ethnicity, n (%):** White British 42 (75),  Other White 3 (5.36); White or Black  Caribbean 3 (5.36); Black Caribbean 2 (3.57); Black African 2 (3.57); Pakistani 2 (3.57); Black or White African 1 (1.79); Other Asian background 1 (1.79).  **Baseline BMI (kg/m^2^), mean (SD):** NR | Clozapine 56  **Mean (SD) daily dose of clozapine:** 349.3 mg (134.0)  **Mean duration of clozapine treatment:** 520.1 days (range: 93-2074 days). | Consecutive  All patients prescribed stable dose of clozapine for  ≥3 months approached.  Of 67 patients approached,  84% agreed to participate in survey. | NA | 1. Increased appetite as a side effect:   Ratings of **side effects** with clozapine in comparison to previously prescribed APs, and ratings of the effects experienced in terms of hedonic response (how participants felt about them).  Measured using a researcher-administered questionnaire. | Non-pharmaceutical funding  No conflicts of interest | N (%) of participants who reported a deterioration in appetite with clozapine in comparison to previously prescribed APs: 11 (19.64%)* |
| (Srour et al., 2023) | Qatar | To investigate the views of patients and their primary carers about clozapine. | Cross-sectional | Outpatient clinic in psychiatry hospital | **Sample size: n= 42**  **Study population:** patients with schizophrenia 37 (88.10), schizoaffective disorder 5 (11.90) prescribed clozapine ≥4 months.  Diagnostic criteria: NR  **Age (years), mean (SD):** 33.90 (10.31)  **Sex, male n (%):** 28 (66.70)  **Nationality, n (%):** Qatari 19 (45.20), non-Qatari 23 (54.80)  **Baseline BMI (kg/m^2^), mean (SD):** NR | Clozapine 42  **Duration of clozapine treatment:**  <1 year: n=4; 1 to < 2 years: n=10; 2 to < 5 years: n=17; 5 to < 10 years: n=10; ≥10 years: n=1.  **Prescribed another AP, n (%):** 14 (33.30), most commonly amisulpride.  **Use of other medications:**  mood stabilizer 14 (33.30);  antidepressant 12 (28.60). | Consecutive.  Of 70 patients approached, 42 (60%) agreed to participate. | NA | 1. Increased appetite:   Ratings of **side effects** with clozapine in comparison to previously prescribed APs.  Clinician-administered questionnaire.  Ratings >3 indicate that the variable was regarded as better during clozapine treatment. | Funding: not stated  No conflicts of interest | Increased appetite mean rating: 2.52 (i.e., clozapine was considered worse than participants’ last prescribed AP). |
| (Murashita et al., 2005) | Japan | To determine the mechanism of appetite and weight gain during olanzapine treatment by investigating circulating ghrelin levels. | Case series | Outpatient | **Sample size: n= 7**  **Study population:**  Schizophrenia  Diagnostic criteria: DSM-IV  **Age (years), mean (SD):** 46.3 (15.7)  **Sex, male n (%):** 4 (57.1)  **Baseline BMI (kg/m^2^), mean (SD):**  27.2 (3.3) | Olanzapine 7  **Mean (SD) daily dose of olanzapine:** 10.7 (1.9) mg/day  **Prior AP treatment, n:**  risperidone 2,  haloperidol 3,  zotepine and quetiapine 2. | Not explained | 6 months | **1. Appetite:**  Patients were regularly interviewed regarding appetite, and classified into ‘increased’, ‘unchanged’, and ‘decreased’.' | Funding: not stated  Conflicts: not stated | N (%) of participants who reported increase in appetite from baseline after 6 months of olanzapine: 6/7 (85.70) |
| (Horiguchi et al., 1999) | Japan | A case report of nocturnal eating/drinking syndrome (NEDS) caused by neuroleptic-induced restless leg syndrome. | Case report | Sleep research clinic | **Sample size: n= 1**  **Study population:**  Schizophrenia  Diagnostic criteria: DSM-IV  **Age**: 51  **Sex**: Female  **Baseline BMI (kg/m^2^), mean (SD):**  NR | Haloperidol 3mg/day for 2 years  **Prior AP treatment:** APs for past 30 years (no details provided). | NA | 1 month | **NEDS:**  Nocturnal recordings of eating-related awakenings due to restless leg syndrome (RLS). | Funding: not stated  Conflicts: not stated | A case report NEDS (of boiled rice, hot milk with sugar) caused by neuroleptic-induced restless leg syndrome while receiving haloperidol. |

AP: antipsychotic medication; adj.: adjusted; BED: binge eating disorder; BS: Binge symptomatology; BMI: body mass index; CBT: cognitive behvaioural therapy; CFCA: Cuestionario de Frecuencia de Consumo de Alimentos; CI: confidence interval; CTRU: Clinical Trials Research Unit; d: day; DANSDA: Danish National Survey of Dietary Habits and Physical Activity; DDD: Defined Daily Doses; DEB: disordered eating behaviours; DEBQ: Dutch Eating Behavior Questionnaire; DINE: Dietary Instrument for Nutrition Education questionnaire; DQS: dietary quality score; DSM-IV: Diagnostic and Statistical Manual of Mental Disorders, 4th Edition; DSM-IV-TR: Diagnostic and Statistical Manual of Mental Disorders, 4th Edition, Text Revision; DSM-5: Diagnostic and Statistical Manual of Mental Disorders, 5^th^ Edition; EAS: Eating Appetite Scale/Eating Attitude Scale; EAT-26: Eating Attitude Test; EBA: Eating Behavior Assessment; EI: Eating Inventory; EPIC: European Prospective Investigation into Cancer and Nutrition Questionnaire; FA: food addiction; FCI: Food Craving Inventory; FCI-SP: Food Craving Inventory-Spanish version; FEP: first episode of psychosis; FGA: first-generation antipsychotic; FFQ: food frequency questionnaire; g: grams; ICD-10: International Classification of Diseases 10th Revision; INRAN-SCAI: Italian National Food Consumption Survey; IQ: interquartile range; kcal: kilocalories; kg/m^2^: kilogram per square metre; LAI: long-acting injectable; MAC-R: Revised version of the Mizes Anorectic Cognitions questionnaire; MedDietScore: Mediterranean diet score questionnaire; mg: milligrams; mm: millimetres; NHANES: National Health and Nutrition Examination Survey; NR: not reported; n: number; NW: normal weight; OCT: oral conventional tablet; ODT: orodispersible tablet; OR: odds ratio; OWO: overweight/obese; p: p-value; PARS: Platypus Appetite Rating Scale; RCT: randomised controlled trial; RLS: restless leg syndrome; SD: standard deviation; SGA: second-generation antipsychotic; SMI: severe mental illness; SOT: standard olanzapine tablets; SQ: satiety quotient; SSRIs: selective serotonin reuptake inhibitors; T2DM: Type 2 diabetes mellitus; TCA: tricyclic antidepressants; TEAEs: treatment-emergent adverse events; TFEQ: Three-Factor Eating Questionnaire; TFEQ-R21: Three-Factor Eating Questionnaire-Revised 21-item Version; VAS: visual analogue scale; WCP: weight control programme; YFAS: Yale Food Addiction Scale.

# References

Abbas, M. J. & Liddle, P. F. (2013). ‘Olanzapine and food craving: A case control study’ *Hum Psychopharmacol*, 28 (1), pp. 97-101. DOI: 10.1002/hup.2278 Available at: <https://www.ncbi.nlm.nih.gov/pubmed/23169487>.

Archie, S. M., et al. (2007). ‘Psychotic disorders, eating habits, and physical activity: Who is ready for lifestyle changes?’ *Psychiatric Services*, 58 (2), pp. 233-239. DOI: 10.1176/ps.2007.58.2.233.

Ballon, J. S., et al. (2018). ‘Pathophysiology of drug induced weight and metabolic effects: Findings from an rct in healthy volunteers treated with olanzapine, iloperidone, or placebo’ *J Psychopharmacol*, 32 (5), pp. 533-540. DOI: 10.1177/0269881118754708 Available at: <https://www.ncbi.nlm.nih.gov/pubmed/29444618>.

Bitter, I., et al. (2010). ‘Patients' preference for olanzapine orodispersible tablet compared with conventional oral tablet in a multinational, randomized, crossover study’ *World J Biol Psychiatry*, 11 (7), pp. 894-903. DOI: 10.3109/15622975.2010.505663 Available at: <https://www.ncbi.nlm.nih.gov/pubmed/20653494>.

Block, G., Gillespie, C., Rosenbaum, E. H. & Jenson, C. (2000). ‘A rapid food screener to assess fat and fruit and vegetable intake’ *Am J Prev Med*, 18 (4), pp. 284-8. DOI: 10.1016/s0749-3797(00)00119-7 Available at: <https://www.ncbi.nlm.nih.gov/pubmed/10788730>.

Blouin, M., et al. (2008). ‘Adiposity and eating behaviors in patients under second generation antipsychotics’ *Obesity (Silver Spring)*, 16 (8), pp. 1780-7. DOI: 10.1038/oby.2008.277 Available at: <https://www.ncbi.nlm.nih.gov/pubmed/18535555>.

Briffa, D. & Meehan, T. (1998). ‘Weight changes during clozapine treatment’ *Aust N Z J Psychiatry*, 32 (5), pp. 718-21. DOI: 10.3109/00048679809113128 Available at: <https://www.ncbi.nlm.nih.gov/pubmed/9805596>.

Case, M., Treuer, T., Karagianis, J. & Hoffmann, V. P. (2010). ‘The potential role of appetite in predicting weight changes during treatment with olanzapine’ *BMC Psychiatry*, 10 p. 72. DOI: 10.1186/1471-244X-10-72 Available at: <https://www.ncbi.nlm.nih.gov/pubmed/20840778>.

Costa E Silva, J. A., et al. (2001). ‘Olanzapine as alternative therapy for patients with haloperidol-induced extrapyramidal symptoms: Results of a multicenter, collaborative trial in latin america’ *J Clin Psychopharmacol*, 21 (4), pp. 375-81. DOI: 10.1097/00004714-200108000-00004 Available at: <https://www.ncbi.nlm.nih.gov/pubmed/11476121>.

Daurignac, E., Leonard, K. E. & Dubovsky, S. L. (2015). ‘Increased lean body mass as an early indicator of olanzapine-induced weight gain in healthy men’ *Int Clin Psychopharmacol*, 30 (1), pp. 23-8. DOI: 10.1097/YIC.0000000000000052 Available at: <https://www.ncbi.nlm.nih.gov/pubmed/25350366>.

de Beaurepaire, R. (2021). ‘Binge eating disorders in antipsychotic-treated patients with schizophrenia: Prevalence, antipsychotic specificities, and changes over time’ *J Clin Psychopharmacol*, 41 (2), pp. 114-120. DOI: 10.1097/JCP.0000000000001357 Available at: <https://www.ncbi.nlm.nih.gov/pubmed/33587392>.

Fountaine, R. J., et al. (2010). ‘Increased food intake and energy expenditure following administration of olanzapine to healthy men’ *Obesity (Silver Spring)*, 18 (8), pp. 1646-51. DOI: 10.1038/oby.2010.6 Available at: <https://www.ncbi.nlm.nih.gov/pubmed/20134408> (Accessed: 2023/06/22).

Friedrich, M., Fugiel, J. & Sadowska, J. (2023). 'Assessing effects of diet alteration on carbohydrate–lipid metabolism of antipsychotic-treated schizophrenia patients in interventional study', *Nutrients*, 15(8) [Online]. DOI: 10.3390/nu15081871 Available at: <https://doi.org/10.3390/nu15081871>.

Garriga, M., et al. (2019). ‘Food craving and consumption evolution in patients starting treatment with clozapine’ *Psychopharmacology (Berl)*, 236 (11), pp. 3317-3327. DOI: 10.1007/s00213-019-05291-3 Available at: <https://www.ncbi.nlm.nih.gov/pubmed/31197435>.

Goluza, I., et al. (2017). ‘Exploration of food addiction in people living with schizophrenia’ *Asian J Psychiatr*, 27 pp. 81-84. DOI: 10.1016/j.ajp.2017.02.022 Available at: <https://www.ncbi.nlm.nih.gov/pubmed/28558903>.

Gothelf, D., et al. (2002). ‘Weight gain associated with increased food intake and low habitual activity levels in male adolescent schizophrenic inpatients treated with olanzapine’ *Am J Psychiatry*, 159 (6), pp. 1055-7. DOI: 10.1176/appi.ajp.159.6.1055 Available at: <https://www.ncbi.nlm.nih.gov/pubmed/12042200>.

Hardy, T., et al. 'Insulin sensitivity in patients with schizophrenia or schizoaffective disorder treated with olanzapine or risperidone', *162nd Annual Meeting Shaping our Future: Science and Service*, San Francisco: American Psychiatric Association, pp. 14-15.

Henderson, D. C., et al. (2006). ‘Dietary intake profile of patients with schizophrenia’ *Ann Clin Psychiatry*, 18 (2), pp. 99-105. DOI: 10.1080/10401230600614538 Available at: <https://www.ncbi.nlm.nih.gov/pubmed/16754415>.

Henderson, D. C., et al. (2010). ‘Dietary saturated fat intake and glucose metabolism impairments in nondiabetic, nonobese patients with schizophrenia on clozapine or risperidone’ *Ann Clin Psychiatry*, 22 (1), pp. 33-42. Available at: <https://www.ncbi.nlm.nih.gov/pubmed/20196981>.

Hoffman, V. P., Case, M. & Jacobson, J. G. 'Algorithms including amantadine, metformin and zonisamide for mitigation of weight gain during olanzapine treatment in outpatients with schizophrenia', *APA San Francisco*

Hoffmann, V. P., Case, M. & Jacobson, J. G. (2012). ‘Assessment of treatment algorithms including amantadine, metformin, and zonisamide for the prevention of weight gain with olanzapine: A randomized controlled open-label study’ *J Clin Psychiatry*, 73 (2), pp. 216-23. DOI: 10.4088/JCP.09m05580 Available at: <https://www.ncbi.nlm.nih.gov/pubmed/21672497>.

Holt, R. I., et al. (2018). ‘Structured lifestyle education to support weight loss for people with schizophrenia, schizoaffective disorder and first episode psychosis: The stepwise rct’ *Health Technol Assess*, 22 (65), pp. 1-160. DOI: 10.3310/hta22650 Available at: <https://www.ncbi.nlm.nih.gov/pubmed/30499443>.

Horiguchi, J., et al. (1999). ‘Nocturnal eating/drinking syndrome and neuroleptic-induced restless legs syndrome’ *Int Clin Psychopharmacol*, 14 (1), pp. 33-6. Available at: <https://www.ncbi.nlm.nih.gov/pubmed/10221640>.

Huang, J., et al. (2020). ‘Corrigendum: Increased appetite plays a key role in olanzapine-induced weight gain in first-episode schizophrenia patients’ *Front Pharmacol*, 11 p. 878. DOI: 10.3389/fphar.2020.00878 Available at: <https://www.ncbi.nlm.nih.gov/pubmed/32587520>.

Jakobsen, A. S., et al. (2018a). ‘Associations between clinical and psychosocial factors and metabolic and cardiovascular risk factors in overweight patients with schizophrenia spectrum disorders - baseline and two-years findings from the change trial’ *Schizophr Res*, 199 pp. 96-102. DOI: 10.1016/j.schres.2018.02.047 Available at: <https://doi.org/10.1016/j.schres.2018.02.047>.

Jakobsen, A. S., et al. (2017). ‘Effect of lifestyle coaching versus care coordination versus treatment as usual in people with severe mental illness and overweight: Two-years follow-up of the randomized change trial’ *PLoS One*, 12 (10), p. e0185881. DOI: 10.1371/journal.pone.0185881 Available at: <https://www.ncbi.nlm.nih.gov/pubmed/28985228>.

Jakobsen, A. S., et al. (2018b). ‘Dietary patterns and physical activity in people with schizophrenia and increased waist circumference’ *Schizophr Res*, 199 pp. 109-115. DOI: 10.1016/j.schres.2018.03.016 Available at: <https://www.ncbi.nlm.nih.gov/pubmed/29555213>.

Kane, J. M., et al. (2001). ‘Clozapine and haloperidol in moderately refractory schizophrenia: A 6-month randomized and double-blind comparison’ *Arch Gen Psychiatry*, 58 (10), pp. 965-72. DOI: 10.1001/archpsyc.58.10.965 Available at: <https://www.ncbi.nlm.nih.gov/pubmed/11576036> (Accessed: 6/23/2023).

Kang, D., et al. (2024). ‘The effect of continuous theta burst stimulation on antipsychotic-induced weight gain in first-episode drug-naive individuals with schizophrenia: A double-blind, randomized, sham-controlled feasibility trial’ *Transl Psychiatry*, 14 (1), p. 61. DOI: 10.1038/s41398-024-02770-w Available at: <https://www.ncbi.nlm.nih.gov/pubmed/38272892>.

Karagianis, J., et al. (2009). ‘A randomized controlled trial of the effect of sublingual orally disintegrating olanzapine versus oral olanzapine on body mass index: The platypus study’ *Schizophr Res*, 113 (1), pp. 41-8. DOI: 10.1016/j.schres.2009.05.024 Available at: <https://www.ncbi.nlm.nih.gov/pubmed/19535229>.

Khazaal, Y., et al. (2009). ‘Hunger and negative alliesthesia to aspartame and sucrose in patients treated with antipsychotic drugs and controls’ *Eat Weight Disord*, 14 (4), pp. e225-30. DOI: 10.1007/BF03325121 Available at: <https://www.ncbi.nlm.nih.gov/pubmed/20179410>.

Khazaal, Y., Fresard, E., Borgeat, F. & Zullino, D. (2006a). ‘Binge eating symptomatology in overweight and obese patients with schizophrenia: A case control study’ *Ann Gen Psychiatry*, 5 p. 15. DOI: 10.1186/1744-859X-5-15 Available at: <https://www.ncbi.nlm.nih.gov/pubmed/16968528>.

Khazaal, Y., et al. (2007). ‘Cognitive behavioural therapy for weight gain associated with antipsychotic drugs’ *Schizophr Res*, 91 (1-3), pp. 169-77. DOI: 10.1016/j.schres.2006.12.025 Available at: <https://www.ncbi.nlm.nih.gov/pubmed/17306507>.

Khazaal, Y., et al. (2006b). ‘Eating and weight related cognitions in people with schizophrenia : A case control study’ *Clin Pract Epidemiol Ment Health*, 2 p. 29. DOI: 10.1186/1745-0179-2-29 Available at: <https://www.ncbi.nlm.nih.gov/pubmed/17076886>.

Khosravi, M. (2020). ‘Biopsychosocial factors associated with disordered eating behaviors in schizophrenia’ *Ann Gen Psychiatry*, 19 (1), p. 67. DOI: 10.1186/s12991-020-00314-2 Available at: <https://www.ncbi.nlm.nih.gov/pubmed/33292324>.

Kirkegaard, A., Hammershoj, E. & Ostergard, P. (1982). ‘Evaluation of side effects due to clozapine in long-term treatment of psychosis’ *Arzneimittel-Forschung*, 32 (4), pp. 465-8. Available at: <http://ovidsp.ovid.com/ovidweb.cgi?T=JS&PAGE=reference&D=med2&NEWS=N&AN=7201818>.

Kluge, M., et al. (2007). ‘Clozapine and olanzapine are associated with food craving and binge eating: Results from a randomized double-blind study’ *J Clin Psychopharmacol*, 27 (6), pp. 662-6. DOI: 10.1097/jcp.0b013e31815a8872 Available at: <https://www.ncbi.nlm.nih.gov/pubmed/18004133>.

Kouidrat, Y., et al. (2018). ‘Disordered eating behaviors as a potential obesogenic factor in schizophrenia’ *Psychiatry Res*, 269 pp. 450-454. DOI: 10.1016/j.psychres.2018.08.083 Available at: <https://www.ncbi.nlm.nih.gov/pubmed/30195737>.

Kurpad, S. S., George, S. A. & Srinivasan, K. (2010). ‘Binge eating and other eating behaviors among patients on treatment for psychoses in india’ *Eat Weight Disord*, 15 (3), pp. e136-43. DOI: 10.1007/BF03325293 Available at: <https://www.ncbi.nlm.nih.gov/pubmed/21150249>.

Lappin, J. M., et al. (2018). ‘Cardio-metabolic risk and its management in a cohort of clozapine-treated outpatients’ *Schizophr Res*, 199 pp. 367-373. DOI: 10.1016/j.schres.2018.02.035 Available at: <https://www.ncbi.nlm.nih.gov/pubmed/29486959>.

Leclercq, C., et al. (2009). ‘The italian national food consumption survey inran-scai 2005-06: Main results in terms of food consumption’ *Public Health Nutr*, 12 (12), pp. 2504-32. DOI: 10.1017/S1368980009005035 Available at: <https://www.ncbi.nlm.nih.gov/pubmed/19278564>.

Llorca, P. M., et al. (2017). ‘Assessing the burden of treatment-emergent adverse events associated with atypical antipsychotic medications’ *BMC Psychiatry*, 17 (1), p. 67. DOI: 10.1186/s12888-017-1213-6 Available at: <https://www.ncbi.nlm.nih.gov/pubmed/28193195>.

Mathews, J., et al. (2012). ‘Neural correlates of weight gain with olanzapine’ *Arch Gen Psychiatry*, 69 (12), pp. 1226-37. DOI: 10.1001/archgenpsychiatry.2012.934 Available at: <https://www.ncbi.nlm.nih.gov/pubmed/22868896>.

Morell, R., et al. (2019). ‘Cardio-metabolic risk in individuals prescribed long-acting injectable antipsychotic medication’ *Psychiatry Res*, 281 p. 112606. DOI: 10.1016/j.psychres.2019.112606 Available at: <https://www.ncbi.nlm.nih.gov/pubmed/31629301>.

Murashita, M., et al. (2005). ‘Olanzapine increases plasma ghrelin level in patients with schizophrenia’ *Psychoneuroendocrinology*, 30 (1), pp. 106-10. DOI: 10.1016/j.psyneuen.2004.05.008 Available at: <https://www.ncbi.nlm.nih.gov/pubmed/15358448>.

Ntalkitsi, S., Efthymiou, D., Bozikas, V. & Vassilopoulou, E. (2022). 'Halting the metabolic complications of antipsychotic medication in patients with a first episode of psychosis: How far can we go with the mediterranean diet? A pilot study', *Nutrients*, 14(23) [Online]. DOI: 10.3390/nu14235012 Available at: <https://doi.org/10.3390/nu14235012>.

Nunes, D., et al. (2014). ‘Nutritional status, food intake and cardiovascular disease risk in individuals with schizophrenia in southern brazil: A case-control study’ *Rev Psiquiatr Salud Ment*, 7 (2), pp. 72-9. DOI: 10.1016/j.rpsm.2013.07.001 Available at: <https://www.ncbi.nlm.nih.gov/pubmed/24054065>.

Park, S., Yi, K. K., Kim, M. S. & Hong, J. P. (2013). ‘Effects of ziprasidone and olanzapine on body composition and metabolic parameters: An open-label comparative pilot study’ *Behav Brain Funct*, 9 p. 27. DOI: 10.1186/1744-9081-9-27 Available at: <https://www.ncbi.nlm.nih.gov/pubmed/23866300>.

Qurashi, I., et al. (2015). ‘An evaluation of subjective experiences, effects and overall satisfaction with clozapine treatment in a uk forensic service’ *Ther Adv Psychopharmacol*, 5 (3), pp. 146-50. DOI: 10.1177/2045125315581996 Available at: <https://www.ncbi.nlm.nih.gov/pubmed/26199716> (Accessed: 2023/06/30).

Roerig, J. L., et al. (2005). ‘A comparison of the effects of olanzapine and risperidone versus placebo on eating behaviors’ *J Clin Psychopharmacol*, 25 (5), pp. 413-8. DOI: 10.1097/01.jcp.0000177549.36585.29 Available at: <https://www.ncbi.nlm.nih.gov/pubmed/16160615>.

Ruggeri, M., et al. (2015). ‘Feasibility and effectiveness of a multi-element psychosocial intervention for first-episode psychosis: Results from the cluster-randomized controlled get up piano trial in a catchment area of 10 million inhabitants’ *Schizophr Bull*, 41 (5), pp. 1192-203. DOI: 10.1093/schbul/sbv058 Available at: <https://www.ncbi.nlm.nih.gov/pubmed/25995057> (Accessed: 2/14/2023).

Saugo, E., et al. (2020). ‘Dietary habits and physical activity in first-episode psychosis patients treated in community services. Effect on early anthropometric and cardio-metabolic alterations’ *Schizophr Res*, 216 pp. 374-381. DOI: 10.1016/j.schres.2019.11.010 Available at: <https://www.ncbi.nlm.nih.gov/pubmed/31806524>.

Sentissi, O., et al. (2009). ‘Impact of antipsychotic treatments on the motivation to eat: Preliminary results in 153 schizophrenic patients’ *Int Clin Psychopharmacol*, 24 (5), pp. 257-64. DOI: 10.1097/YIC.0b013e32832b6bf6 Available at: <https://www.ncbi.nlm.nih.gov/pubmed/19606055>.

Smith, R. C., Rachakonda, S., Dwivedi, S. & Davis, J. M. (2012). ‘Olanzapine and risperidone effects on appetite and ghrelin in chronic schizophrenic patients’ *Psychiatry Res*, 199 (3), pp. 159-63. DOI: 10.1016/j.psychres.2012.03.011 Available at: <https://www.ncbi.nlm.nih.gov/pubmed/22475524>.

Speyer, H., et al. (2016). ‘The change trial: No superiority of lifestyle coaching plus care coordination plus treatment as usual compared to treatment as usual alone in reducing risk of cardiovascular disease in adults with schizophrenia spectrum disorders and abdominal obesity’ *World Psychiatry*, 15 (2), pp. 155-65. DOI: 10.1002/wps.20318 Available at: <https://www.ncbi.nlm.nih.gov/pubmed/27265706> (Accessed: 2023/03/12).

Srour, A., et al. (2023). ‘Patients' and primary carers' views on clozapine treatment for schizophrenia: A cross-sectional study in qatar’ *Saudi Pharm J*, 31 (2), pp. 214-221. DOI: 10.1016/j.jsps.2022.12.005 Available at: <https://www.ncbi.nlm.nih.gov/pubmed/36942276>.

Stefanska, E., et al. (2017). ‘Eating habits and nutritional status of patients with affective disorders and schizophrenia’ *Psychiatr Pol*, 51 (6), pp. 1107-1120. DOI: 10.12740/PP/74558 Available at: <https://www.ncbi.nlm.nih.gov/pubmed/29432506>.

Stefanska, E., et al. (2018). ‘The assessment of the nutritional value of meals consumed by patients with recognized schizophrenia’ *Rocz Panstw Zakl Hig*, 69 (2), pp. 183-192. Available at: <https://www.ncbi.nlm.nih.gov/pubmed/29766697>.

Stip, E., et al. (2012). ‘Neural changes associated with appetite information processing in schizophrenic patients after 16 weeks of olanzapine treatment’ *Transl Psychiatry*, 2 (6), p. e128. DOI: 10.1038/tp.2012.53 Available at: <https://www.ncbi.nlm.nih.gov/pubmed/22714121>.

Teff, K. L., Rickels, K., Alshehabi, E. & Rickels, M. R. (2015). ‘Metabolic impairments precede changes in hunger and food intake following short-term administration of second-generation antipsychotics’ *J Clin Psychopharmacol*, 35 (5), pp. 579-82. DOI: 10.1097/JCP.0000000000000393 Available at: <https://www.ncbi.nlm.nih.gov/pubmed/26274045>.

Teff, K. L., et al. (2013). ‘Antipsychotic-induced insulin resistance and postprandial hormonal dysregulation independent of weight gain or psychiatric disease’ *Diabetes*, 62 (9), pp. 3232-40. DOI: 10.2337/db13-0430 Available at: <https://www.ncbi.nlm.nih.gov/pubmed/23835329> (Accessed: 5/1/2023).

Tollefson, G. D., et al. (1997). ‘Olanzapine versus haloperidol in the treatment of schizophrenia and schizoaffective and schizophreniform disorders: Results of an international collaborative trial’ *Am J Psychiatry*, 154 (4), pp. 457-65. DOI: 10.1176/ajp.154.4.457 Available at: <https://www.ncbi.nlm.nih.gov/pubmed/9090331> (Accessed: 2023/06/30).

Treuer, T., et al. (2009). ‘Factors associated with weight gain during olanzapine treatment in patients with schizophrenia or bipolar disorder: Results from a six-month prospective, multinational, observational study’ *World J Biol Psychiatry*, 10 (4 Pt 3), pp. 729-40. DOI: 10.1080/15622970903079507 Available at: <https://www.ncbi.nlm.nih.gov/pubmed/19606406>.

Yang, Y., et al. (2021). ‘Effect of bifidobacterium on olanzapine-induced body weight and appetite changes in patients with psychosis’ *Psychopharmacology (Berl)*, 238 (9), pp. 2449-2457. DOI: 10.1007/s00213-021-05866-z Available at: <https://www.ncbi.nlm.nih.gov/pubmed/34002246>.
