# Supplementary material for: The impacts of antipsychotic medications on eating-related outcomes: A mixed methods systematic review
Source: PLoS One. 2025 Feb 3;20(2):e0308037. doi: 10.1371/journal.pone.0308037 (PMC11790239; doi:10.1371/journal.pone.0308037)
Supplement: S6 File — (DOCX) [file pone.0308037.s006.docx]

**S6 File. Studies retrieved for full-text examination, including those that were excluded from the analyses and reasons for exclusion.**

|  | **Covidence ID** | **Citation** | **Title** | **DOI** | **Decision** | **Reason for exclusion** |
| --- | --- | --- | --- | --- | --- | --- |
|  | #2 | Abbas et al., 2013 | Olanzapine and food craving: a case control study | DOI: [10.1002/hup.2278](https://dx.doi.org/10.1002/hup.2278) | Included | Not applicable |
|  | #56 | Archie et al., 2007 | Psychotic disorders, eating habits, and physical activity: Who is ready for lifestyle changes? | DOI: [10.1176/appi.ps.58.2.233](https://dx.doi.org/10.1176/appi.ps.58.2.233) | Included | Not applicable |
|  | #92 | Ballon et al., 2018 | Pathophysiology of drug induced weight and metabolic effects: findings from an RCT in healthy volunteers treated with olanzapine, iloperidone, or placebo | DOI: [10.1177/0269881118754708](https://dx.doi.org/10.1177/0269881118754708) | Included | Not applicable |
|  | #4799 | Bitter et al., 2010 | Patients' preference for olanzapine orodispersible tablet compared with conventional oral tablet in a multinational, randomized, crossover study | DOI: [10.3109/15622975.2010.505663](https://dx.doi.org/10.3109/15622975.2010.505663) | Included | Not applicable |
|  | #172 | Blouin et al., 2008 | Adiposity and eating behaviors in patients under second generation antipsychotics | DOI: [10.1038/oby.2008.277](https://dx.doi.org/10.1038/oby.2008.277) | Included | Not applicable |
|  | #212 | Briffa and Meehan, 1998 | Weight changes during clozapine treatment | DOI: [10.3109/00048679809113128](https://dx.doi.org/10.3109/00048679809113128) | Included | Not applicable |
|  | #260 | Case et al., 2010 | The potential role of appetite in predicting weight changes during treatment with olanzapine | DOI: [10.1186/1471-244X-10-72](https://dx.doi.org/10.1186/1471-244X-10-72) | Included | Not applicable |
|  | #351 | Costa E Silva et al., 2001 | Olanzapine as alternative therapy for patients with haloperidol-induced extrapyramidal symptoms: Results of a multicenter, collaborative trial in Latin America | DOI: [10.1097/00004714-200108000-00004](https://dx.doi.org/10.1097/00004714-200108000-00004) | Included | Not applicable |
|  | #4795 | Daurignac et al., 2015 | Increased lean body mass as an early indicator of olanzapine-induced weight gain in healthy men | DOI: 10.1097/YIC.0000000000000052 | Included | Not applicable |
|  | #391 | De Beaurepaire, 2021 | Binge Eating Disorders in Antipsychotic-Treated Patients With Schizophrenia Prevalence, Antipsychotic Specificities, and Changes Over Time | DOI: [10.1097/JCP.0000000000001357](https://dx.doi.org/10.1097/JCP.0000000000001357) | Included | Not applicable |
|  | #4796 | Fountaine et al., 2010 | Increased Food Intake and Energy Expenditure Following Administration of Olanzapine to Healthy Men | DOI: [10.1038/oby.2010.6](https://dx.doi.org/10.1038/oby.2010.6) | Included | Not applicable |
|  | #5578 | Friedrich et al., 2023 | Assessing Effects of Diet Alteration on Carbohydrate-Lipid Metabolism of Antipsychotic-Treated Schizophrenia Patients in Interventional Study | DOI: [10.3390/nu15081871](https://dx.doi.org/10.3390/nu15081871) | Included | Not applicable |
|  | #556 | Garriga et al., 2019 | Food craving and consumption evolution in patients starting treatment with clozapine | DOI: [10.1007/s00213-019-05291-3](https://dx.doi.org/10.1007/s00213-019-05291-3) | Included | Not applicable |
|  | #4801 | Goluza et al., 2017 | Exploration of food addiction in people living with schizophrenia | DOI: [10.1016/j.ajp.2017.02.022](https://dx.doi.org/10.1016/j.ajp.2017.02.022) | Included | Not applicable |
|  | #590 | Gothelf et al., 2002 | Weight gain associated with increased food intake and low habitual activity levels in male adolescent schizophrenic inpatients treated with olanzapine | DOI: [10.1176/appi.ajp.159.6.1055](https://dx.doi.org/10.1176/appi.ajp.159.6.1055) | Included | Not applicable |
|  | #646 | Haracz et al., 2018 | The "Double Whammy": Women's Experiences of Weight Gain After Diagnosis and Treatment for Schizophrenia Spectrum Disorders | DOI: [10.1097/NMD.0000000000000803](https://dx.doi.org/10.1097/NMD.0000000000000803) | Included | Not applicable |
|  | #676 | Henderson et a., 2006 | Dietary intake profile of patients with schizophrenia | DOI: 10.1080/10401230600614538 | Included | Not applicable |
|  | #681 | Henderson et al., 2010 | Dietary saturated fat intake and glucose metabolism impairments in nondiabetic, nonobese patients with schizophrenia on clozapine or risperidone | Henderson DC, Sharma B, Fan X, Copeland PM, Borba CP, Freudenreich O, et al. Dietary saturated fat intake and glucose metabolism impairments in nondiabetic, nonobese patients with schizophrenia on clozapine or risperidone. Ann Clin Psychiatry. 2010;22(1):33-42. | Included | Not applicable |
|  | #708 | Holt et al., 2018 | Structured lifestyle education to support weight loss for people with schizophrenia, schizoaffective disorder and first episode psychosis: the STEPWISE RCT | DOI: [10.3310/hta22650](https://dx.doi.org/10.3310/hta22650) | Included | Not applicable |
|  | #715 | Horiguchi et al., 1999 | Nocturnal eating/drinking syndrome and neuroleptic-induced restless legs syndrome | Horiguchi J, Yamashita H, Mizuno S, Kuramoto Y, Kagaya A, Yamawaki S, et al. Nocturnal eating/drinking syndrome and neuroleptic-induced restless legs syndrome. Int Clin Psychopharmacol. 1999;14(1):33-6. | Included | Not applicable |
|  | #725 | Huang et al., 2020 | Increased Appetite Plays a Key Role in Olanzapine-Induced Weight Gain in First-Episode Schizophrenia Patients | DOI: [10.3389/fphar.2020.00878](https://dx.doi.org/10.3389/fphar.2020.00878) | Included | Not applicable |
|  | #756 | Jakobsen et al., 2018 | Associations between clinical and psychosocial factors and metabolic and cardiovascular risk factors in overweight patients with schizophrenia spectrum disorders - Baseline and two-years findings from the CHANGE trial | DOI: [10.1016/j.schres.2018.02.047](https://dx.doi.org/10.1016/j.schres.2018.02.047) | Included | Not applicable |
|  | #758 | Jakobsen et al., 2018 | Dietary patterns and physical activity in people with schizophrenia and increased waist circumference | DOI: [10.1016/j.schres.2018.03.016](https://dx.doi.org/10.1016/j.schres.2018.03.016) | Included | Not applicable |
|  | #4827 | Kaar et al., 2019 | Making decisions about antipsychotics: a qualitative study of patient experience and the development of a decision aid | DOI: [10.1186/s12888-019-2304-3](https://dx.doi.org/10.1186/s12888-019-2304-3) | Included | Not applicable |
|  | #791 | Kane et al., 2001 | Clozapine and haloperidol in moderately refractory schizophrenia - A 6-month randomized and double-blind comparison | DOI: [10.1001/archpsyc.58.10.965](https://dx.doi.org/10.1001/archpsyc.58.10.965) | Included | Not applicable |
|  | #5161 | Kang et al., 2024 | The effect of continuous theta burst stimulation on antipsychotic-induced weight gain in first-episode drug-naive individuals with schizophrenia: a double-blind, randomized, sham-controlled feasibility trial | DOI: [10.1038/s41398-024-02770-w](https://dx.doi.org/10.1038/s41398-024-02770-w) | Included | Not applicable |
|  | #831 | Khazaal et al., 2009 | Hunger and negative alliesthesia to aspartame and sucrose inpatients treated with antipsychotic drugs and controls | DOI: [10.1007/BF03325121](https://dx.doi.org/10.1007/BF03325121) | Included | Not applicable |
|  | #837 | Khazaal et al., 2007 | Cognitive behavioural therapy for weight gain associated with antipsychotic drugs | DOI: [10.1016/j.schres.2006.12.025](https://dx.doi.org/10.1016/j.schres.2006.12.025) | Included | Not applicable |
|  | #834 | Khazaal et al., 2006 | Binge eating symptomatology in overweight and obese patients with schizophrenia: A case control study | DOI: [10.1186/1744-859X-5-15](https://dx.doi.org/10.1186/1744-859X-5-15) | Included | Not applicable |
|  | #839 | Khazaal et al., 2006 | Eating and weight related cognitions in people with schizophrenia: A case control study | DOI: [10.1186/1745-0179-2-29](https://dx.doi.org/10.1186/1745-0179-2-29) | Included | Not applicable |
|  | #842 | Khosravi, 2020 | Biopsychosocial factors associated with disordered eating behaviors in schizophrenia | DOI: [10.1186/s12991-020-00314-2](https://dx.doi.org/10.1186/s12991-020-00314-2) | Included | Not applicable |
|  | #857 | Kirkegaard et al., 1982 | Evaluation of side effects due to clozapine in long-term treatment of psychosis | Kirkegaard A, Hammershoj E, Ostergard P. Evaluation of side effects due to clozapine in long-term treatment of psychosis. Arzneimittel-Forschung. 1982;32(4):465-8. | Included | Not applicable |
|  | #871 | Kluge et al., 2007 | Clozapine and olanzapine are associated with food craving and binge eating - Results from a randomized double-blind study | DOI: [10.1097/jcp.0b013e31815a8872](https://dx.doi.org/10.1097/jcp.0b013e31815a8872) | Included | Not applicable |
|  | #884 | Kouidrat et al., 2018 | Disordered eating behaviors as a potential obesogenic factor in schizophrenia | DOI: [10.1016/j.psychres.2018.08.083](https://dx.doi.org/10.1016/j.psychres.2018.08.083) | Included | Not applicable |
|  | #905 | Kurpad et al., 2010 | Binge eating and other eating behaviors among patients on treatment for psychoses in India | DOI: [10.1007/BF03325293](https://dx.doi.org/10.1007/BF03325293) | Included | Not applicable |
|  | #4802 | Lappin et al., 2018 | Cardio-metabolic risk and its management in a cohort of clozapine-treated outpatients | DOI: [10.1016/j.schres.2018.02.035](https://dx.doi.org/10.1016/j.schres.2018.02.035) | Included | Not applicable |
|  | #991 | Llorca et al., 2017 | Assessing the burden of treatment-emergent adverse events associated with atypical antipsychotic medications | DOI: [10.1186/s12888-017-1213-6](https://dx.doi.org/10.1186/s12888-017-1213-6) | Included | Not applicable |
|  | #1073 | Mathews et al., 2012 | Neural Correlates of Weight Gain With Olanzapine | DOI: [10.1001/archgenpsychiatry.2012.934](https://dx.doi.org/10.1001/archgenpsychiatry.2012.934) | Included | Not applicable |
|  | #4804 | Morell et al., 2019 | Cardio-metabolic risk in individuals prescribed long-acting injectable antipsychotic medication | DOI: [10.1016/j.psychres.2019.112606](https://dx.doi.org/10.1016/j.psychres.2019.112606) | Included | Not applicable |
|  | #1170 | Murashita et al., 2005 | Olanzapine increases plasma ghrelin level in patients with schizophrenia | DOI: [10.1016/j.psyneuen.2004.05.008](https://dx.doi.org/10.1016/j.psyneuen.2004.05.008) | Included | Not applicable |
|  | #2683 | Ntalkitsi et al., 2022 | Halting the Metabolic Complications of Antipsychotic Medication in Patients with a First Episode of Psychosis: How Far Can We Go with the Mediterranean Diet? A Pilot Study | DOI: [10.3390/nu14235012](https://dx.doi.org/10.3390/nu14235012) | Included | Not applicable |
|  | #4816 | Nunes et al., 2014 | Estado nutricional, ingesta alimentaria y riesgo de enfermedad cardiovascular en individuos con esquizofrenia en el sur de Brasil: estudio de casos-controles  Nutritional status, food intake and cardiovascular disease risk in individuals with schizophrenia in southern Brazil: A case---control study | DOI: [10.1016/j.rpsm.2013.07.001](https://dx.doi.org/10.1016/j.rpsm.2013.07.001) | Included | Not applicable |
|  | #1263 | Park et al., 2013 | Effects of ziprasidone and olanzapine on body composition and metabolic parameters: an open-label comparative pilot study | DOI: [10.1186/1744-9081-9-27](https://dx.doi.org/10.1186/1744-9081-9-27) | Included | Not applicable |
|  | #4806 | Qurashi et al., 2015 | An evaluation of subjective experiences, effects and overall satisfaction with clozapine treatment in a UK forensic service | DOI: [10.1177/2045125315581996](https://dx.doi.org/10.1177/2045125315581996) | Included | Not applicable |
|  | #1402 | Roerig et al., 2005 | A comparison of the effects of olanzapine and risperidone versus placebo on eating behaviors | DOI: [10.1097/01.jcp.0000177549.36585.29](https://dx.doi.org/10.1097/01.jcp.0000177549.36585.29) | Included | Not applicable |
|  | #1462 | Saugo et al., 2020 | Dietary habits and physical activity in first-episode psychosis patients treated in community services. Effect on early anthropometric and cardio-metabolic alterations | DOI: [10.1016/j.schres.2019.11.010](https://dx.doi.org/10.1016/j.schres.2019.11.010) | Included | Not applicable |
|  | #1495 | Sentissi et al., 2009 | Impact of antipsychotic treatments on the motivation to eat: preliminary results in 153 schizophrenic patients | DOI: [10.1097/YIC.0b013e32832b6bf6](https://dx.doi.org/10.1097/YIC.0b013e32832b6bf6) | Included | Not applicable |
|  | #1552 | Smith et al., 2012 | Olanzapine and risperidone effects on appetite and ghrelin in chronic schizophrenic patients | DOI: [10.1016/j.psychres.2012.03.011](https://dx.doi.org/10.1016/j.psychres.2012.03.011) | Included | Not applicable |
|  | #5688 | Srour et al., 2023 | Patients? and primary carers? views on clozapine treatment for schizophrenia: A cross-sectional study in Qatar | DOI: [10.1016/j.jsps.2022.12.005](https://dx.doi.org/10.1016/j.jsps.2022.12.005) | Included | Not applicable |
|  | #4819 | Stefańska et al., 2017 | Eating habits and nutritional status of patients with affective disorders and schizophrenia | DOI: [10.12740/PP/74558](https://dx.doi.org/10.12740/PP/74558) | Included | Not applicable |
|  | #1601 | Stip et al., 2012 | Neural changes associated with appetite information processing in schizophrenic patients after 16 weeks of olanzapine treatment | DOI: [10.1038/tp.2012.53](https://dx.doi.org/10.1038/tp.2012.53) | Included | Not applicable |
|  | #1639 | Teferra et al., 2013 | Perspectives on reasons for non-adherence to medication in persons with schizophrenia in Ethiopia: a qualitative study of patients, caregivers and health workers | DOI: [10.1186/1471-244X-13-168](https://dx.doi.org/10.1186/1471-244X-13-168) | Included | Not applicable |
|  | #1641 | Teff et al., 2015 | Metabolic Impairments Precede Changes in Hunger and Food Intake Following Short-Term Administration of Second-Generation Antipsychotics | DOI: [10.1097/JCP.0000000000000393](https://dx.doi.org/10.1097/JCP.0000000000000393) | Included | Not applicable |
|  | #1642 | Teff et al., 2013 | Antipsychotic-induced insulin resistance and postprandial hormonal dysregulation independent of weight gain or psychiatric disease | DOI: [10.2337/db13-0430](https://dx.doi.org/10.2337/db13-0430) | Included | Not applicable |
|  | #1673 | Treuer et al., 2009 | Factors associated with weight gain during olanzapine treatment in patients with schizophrenia or bipolar disorder: results from a six-month prospective, multinational, observational study | DOI: [10.1080/15622970903079507](https://dx.doi.org/10.1080/15622970903079507) | Included | Not applicable |
|  | #1695 | Usher et al., 2013 | The experience of weight gain as a result of taking second-generation antipsychotic medications: the mental health consumer perspective | DOI: [10.1111/jpm.12019](https://dx.doi.org/10.1111/jpm.12019) | Included | Not applicable |
|  | #4823 | Vandyk et al., 2012 | Qualitative descriptive study exploring schizophrenia and the everyday effect of medication-induced weight gain | DOI: [10.1111/j.1447-0349.2011.00790.x](https://dx.doi.org/10.1111/j.1447-0349.2011.00790.x) | Included | Not applicable |
|  | #4820 | Stefańska et al., 2018 | The assessment of the nutritional value of meals consumed by patients with recognized schizophrenia | Stefanska E, Wendolowicz A, Lech M, Wilczynska K, Konarzewska B, Zapolska J, et al. The assessment of the nutritional value of meals consumed by patients with recognized schizophrenia. Rocz Panstw Zakl Hig. 2018;69(2):183-92. | Included | Not applicable |
|  | #1822 | Xiao et al., 2012 | Psychosocial processes influencing weight management among persons newly prescribed atypical antipsychotic medications | DOI: [10.1111/j.1365-2850.2011.01773.x](https://dx.doi.org/10.1111/j.1365-2850.2011.01773.x) | Included | Not applicable |
|  | #4947 | Yang et al., 2021 | Effect of Bifidobacteriumon olanzapine-induced body weight and appetite changes in patients with psychosis | DOI: [10.1007/s00213-021-05866-z](https://dx.doi.org/10.1007/s00213-021-05866-z) | Included | Not applicable |
|  | #4798 | Tollefson et al., 1997 | Olanzapine versus haloperidol in the treatment of schizophrenia and schizoaffective and schizophreniform disorders: results of an international collaborative trial | DOI: [10.1176/ajp.154.4.457](https://dx.doi.org/10.1176/ajp.154.4.457) | Included | Not applicable |
|  | #10 | Adamowicz et al., 2020 | Metabolic Syndrome and Cognitive Functions in Schizophrenia-Implementation of Dietary Intervention | DOI: [10.3389/fpsyt.2020.00359](https://dx.doi.org/10.3389/fpsyt.2020.00359) | Excluded | No eating behaviour measures/outcomes |
|  | #80 | Bachmann et al., 2012 | Subjective and Biological Weight-Related Parameters in Adolescents and Young Adults with Schizophrenia Spectrum Disorder under Clozapine or Olanzapine Treatment | DOI: [10.1024/1422-4917/a000165](https://dx.doi.org/10.1024/1422-4917/a000165) | Excluded | Not in English |
|  | #95 | Baptista et al., 2002 | Mechanisms of weight gain induced by antipsychotic drugs | DOI: [10.4088/JCP.v63n0312a](https://dx.doi.org/10.4088/JCP.v63n0312a) | Excluded | Not primary research |
|  | #5256 | Batko et al., 2023 | Risk factors for weight gain in patients with first-episode psychosis | DOI: [10.12740/PP/146196](https://dx.doi.org/10.12740/PP/146196) | Excluded | <75% sample prescribed antipsychotics |
|  | #137 | Beebe et al., 2018 | Telephone Intervention-Problem Solving (TIPS) for Schizophrenia Spectrum Disorders: Responses of Stable Outpatients Over Nine Months | DOI: [10.1080/01612840.2018.1431824](https://dx.doi.org/10.1080/01612840.2018.1431824) | Excluded | Wrong topic |
|  | #155 | Bernstein and Jerrold, 1987 | Induction of obesity by psychotropic drugs | DOI: [10.1111/j.1749-6632.1987.tb36212.x](https://dx.doi.org/10.1111/j.1749-6632.1987.tb36212.x) | Excluded | Not primary research |
|  | #164 | Bimerew et al., 2007 | Substance abuse and the risk of readmission of people with schizophrenia at Amanuel Psychiatric Hospital, Ethiopia | DOI: [10.4102/curationis.v30i2.1078](https://doi.org/10.4102/curationis.v30i2.1078) | Excluded | No eating behaviour measures/outcomes |
|  | #170 | Blouin et a., 2004 | Satiety and binge eating tendencies among atypical antipsychotic treated-patients | No DOI | Excluded | Library unable to source |
|  | #174 | Blouin et al., 2007 | Adiposity and eating behaviours under atypical antipsychotics | No DOI | Excluded | Conference abstract |
|  | #175 | Blouin et al., 2003 | Diet composition, food preferences and cravings among patients treated with atypical antipsychotics | DOI: [10.1016/S0920-9964(03)80155-X](https://dx.doi.org/10.1016/S0920-9964(03)80155-X) | Excluded | Conference abstract |
|  | #171 | Blouin et al., 2008 | Adiposity and eating behaviors in patients under second generation antipsychotics | DOI: [10.1038/oby.2008.277](https://dx.doi.org/10.1038/oby.2008.277) | Excluded | duplicate |
|  | #179 | Bobo et al., 2011 | Effects of orally disintegrating vs regular olanzapine tablets on body weight, eating behavior, glycemic and lipid indices, and gastrointestinal hormones: A randomized, open comparison in outpatients with bipolar depression | No DOI | Excluded | >25% sample eating disorders, autism, dementia or severe or profound learning disability |
|  | #5584 | Brown et al., 2009 | Olanzapine/fluoxetine combination vs lamotrigine in the 6-month treatment of bipolar I depression | DOI: [10.1017/S1461145708009735](https://dx.doi.org/10.1017/S1461145708009735) | Excluded | >25% sample eating disorders, autism, dementia or severe or profound learning disability |
|  | #238 | Bussell et al., 2021 | Dietary Consumption Among Youth with Antipsychotic-Induced Weight Gain and Changes Following Healthy Lifestyle Education | DOI: [10.1089/cap.2020.0173](https://dx.doi.org/10.1089/cap.2020.0173) | Excluded | Not primary research |
|  | #237 | Bussell, 2019 | Examining dietary patterns and relationship to caloric intake in a sample of youth with antipsychotic induced weight gain | Dissertation Abstracts International: Section B: The Sciences and Engineering 2019;80(11-B(E)):No-Specified | Excluded | Not primary research |
|  | #259 | Cascade et al., 2010 | Real-world data on atypical antipsychotic medication side effects | No DOI | Excluded | Data of interest not reported |
|  | #2550 | Chandradasa et al., 2022 | Randomised, placebo-controlled trial on topiramate add-on therapy for weight reduction and symptomatology in overweight/obese persons with schizophrenia | [10.1016/j.ajp.2021.102963](https://dx.doi.org/10.1016/j.ajp.2021.102963) | Excluded | No eating behaviour measures/outcomes |
|  | #281 | Chee et al., 2019 | Parents' Perceptions of the Physical Health Outcomes of Young People Diagnosed with First Episode Psychosis | [10.1080/01612840.2018.1537322](https://dx.doi.org/10.1080/01612840.2018.1537322) | Excluded | No eating behaviour measures/outcomes |
|  | #2520 | Choong et al., 2012 | Psychotropic drug-induced weight gain and other metabolic complications in a Swiss psychiatric population | [10.1016/j.jpsychires.2012.01.014](https://dx.doi.org/10.1016/j.jpsychires.2012.01.014) | Excluded | >25% sample eating disorders, autism, dementia or severe or profound learning disability |
|  | #303 | Chopra, 2019 | Sleep Walking and Sleep Related Eating Disorder Associated with Atypical Antipsychotic Medications: Case Series and Review of the Literature | [10.1016/j.genhosppsych.2020.05.014](https://doi.org/10.1016/j.genhosppsych.2020.05.014) | Excluded | Data of interest not reported |
|  | #305 | Chopra 2020 | Sleepwalking and sleep-related eating associated with atypical antipsychotic medications: Case series and systematic review of literature | [10.1016/j.genhosppsych.2020.05.014](https://dx.doi.org/10.1016/j.genhosppsych.2020.05.014) | Excluded | Not primary research; duplicate |
|  | #315 | Coates et al., 2018 | Evaluation of a general practitioner-led cardiometabolic clinic: Physical health profile andtreatment outcomes for clients on clozapine | [10.1111/inm.12321](https://dx.doi.org/10.1111/inm.12321) | Excluded | No eating behaviour measures/outcomes |
|  | #348 | Correll 2006 | Effect of atypical antipsychotics on hypothalamic key regulatory peptides for appetite and energy balance in treatment-naive youth | No DOI | Excluded | Abstract only |
|  | #2917 | Das, 2016 | A case of sleepwalking with sleep-related eating associated with ziprasidone therapy in a patient with schizoaffective disorder. | [10.1097/JCP.0000000000000525https://dx.doi.org/10.1097/JCP.0000000000000525](https://dx.doi.org/10.1097/JCP.0000000000000525https:/dx.doi.org/10.1097/JCP.0000000000000525) | Excluded | Data of interest not reported |
|  | #387 | Davison, 2013 | The relationships among psychiatric medications, eating behaviors, and weight | [10.1016/j.eatbeh.2013.01.001](https://dx.doi.org/10.1016/j.eatbeh.2013.01.001) | Excluded | No eating behaviour measures/outcomes |
|  | #5252 | Eder et al., 2024 | Who is at risk for weight gain after weight-gain associated treatment with antipsychotics, antidepressants, and mood stabilizers: A machine learning approach | [10.1111/acps.13684](https://dx.doi.org/10.1111/acps.13684) | Excluded | >25% sample eating disorders, autism, dementia or severe or profound learning disability |
|  | #500 | Fenton et al., 2006 | Medication-induced weight gain and dyslipidemia in patients with schizophrenia | [10.1176/appi.ajp.163.10.1697](https://dx.doi.org/10.1176/appi.ajp.163.10.1697) | Excluded | Not primary research |
|  | #534 | Fresard et al., 2006 | Binge eating associated with antipsychotic treatment: A single case study | No DOI | Excluded | Not in English |
|  | #621 | Gury, 2004 | Schizophrenia, diabetes mellitus and antipsychotics | No DOI | Excluded | Not in English |
|  | #641 | Hampton, 2007 | Antipsychotics' link to weight gain found | <https://jamanetwork.com/journals/jama/issue/297/12> | Excluded | Not primary research |
|  | #678 | Henderson and Doraiswamy, 2008 | Prolactin-related and metabolic adverse effects of atypical antipsychotic agents | [10.4088/JCP.0208e04](https://dx.doi.org/10.4088/JCP.0208e04) | Excluded | Not primary research |
|  | #4809 | Hoffmann et al., 2011 | Assessment of Treatment Algorithms Including Amantadine, Metformin, and Zonisamide for the Prevention of Weight Gain With Olanzapine: A Randomized Controlled Open-Label Study | [10.4088/JCP.09m05580](https://dx.doi.org/10.4088/JCP.09m05580) | Excluded | No eating behaviour measures/outcomes |
|  | #710 | Holt and Peveler, 2009  2009 JUL | Obesity, serious mental illness and antipsychotic drugs | [10.1111/j.1463-1326.2009.01038.x](https://dx.doi.org/10.1111/j.1463-1326.2009.01038.x) | Excluded | Not primary research |
|  | #724 | Huang et al., 2020 | Increased Appetite Plays a Key Role in Olanzapine-Induced Weight Gain in | [10.3389/fphar.2020.00739](https://dx.doi.org/10.3389/fphar.2020.00739) | Excluded | duplicate |
|  | #733 | Huthwaite et al., 2017 | Obesity in a forensic and rehabilitation psychiatric service: a missed opportunity? | [10.1108/JFP-03-2017-0007](https://dx.doi.org/10.1108/JFP-03-2017-0007) | Excluded | Wrong topic |
|  | #767 | Jeong et al., 2007 | The functional MRI study underlying decreased food craving in schizophrenic patients taking second generation antipsychotics | No DOI | Excluded | Abstract only |
|  | #775 | Jones et a;., 2001 | Weight change and atypical antipsychotic treatment in patients with schizophrenia | No DOI | Excluded | No eating behaviour measures/outcomes |
|  | #5652 | Kar and Barreto, 2024 | Influence of Lifestyle Factors on Metabolic Syndrome in Psychiatric Patients Attending a Community Mental Health Setting: A Cross-sectional Study | [10.1177/02537176231219770](https://dx.doi.org/10.1177/02537176231219770) | Excluded | >25% sample eating disorders, autism, dementia or severe or profound learning disability |
|  | #830 | Khazaal et al., 2010 | A Measure of Dysfunctional Eating-Related Cognitions in People with Psychotic Disorders | [10.1007/s11126-009-9117-3](https://dx.doi.org/10.1007/s11126-009-9117-3) | Excluded | No eating behaviour measures/outcomes |
|  | #832 | Khazaal et al., 2008 | Antipsychotic drug and body weight set-point | [10.1016/j.physbeh.2008.05.013](https://dx.doi.org/10.1016/j.physbeh.2008.05.013) | Excluded | No eating behaviour measures/outcomes |
|  | #835 | Khazaal et al., 2008 | Cognitive behavioural therapy for obesity and binge eating associated to antipsychotic drugs | No DOI | Excluded | Wrong topic |
|  | #836 | Khazaal et al., 2005 | Antipsychotic induced weight gain: Place and treatment of restraint: A preliminary study | No DOI | Excluded | Not in English |
|  | #840 | Khazaal et al., 2007 | Dietary underreporting in women with schizophrenia requiring dietary intervention: A case control study | DOI: [10.1007/BF03327600](https://dx.doi.org/10.1007/BF03327600) | Excluded | No eating behaviour measures/outcomes |
|  | #838 | Khazaal et al., 2007 | Apple-pie group, conceptualization of a treatment module for antipsychotic induced weight gain  Apple-pie group, conceptualisation d'un module de traitement de la prise de poids associee aux traitements antipsychotiques. | No DOI | Excluded | Not in English |
|  | #843 | Khosravi et al., 2021 | Key factors involved in the feeding and eating disorders among schizophrenic patients and non-clinical controls | DOI: [10.12740/APP/127307](https://dx.doi.org/10.12740/APP/127307) | Excluded | No eating behaviour measures/outcomes |
|  | #851 | Kim et al., 2006 | A 12-week, randomized, open-label, parallel-group trial of topiramate in limiting weight gain during olanzapine treatment in patients with schizophrenia | DOI: [10.1016/j.schres.2005.10.001](https://dx.doi.org/10.1016/j.schres.2005.10.001) | Excluded | Not primary research |
|  | #854 | Kim et al., 2020 | The Psychology of Food Cravings in Patients With First-Episode Psychosis | DOI: [10.3389/fpsyt.2020.587486](https://dx.doi.org/10.3389/fpsyt.2020.587486) | Excluded | No eating behaviour measures/outcomes |
|  | #855 | Kinon et al., 2005 | Association between early and rapid weight gain and change in weight over one year of olanzapine therapy in patients with schizophrenia and related disorders | DOI: [10.1097/01.jcp.0000161501.65890.22](https://dx.doi.org/10.1097/01.jcp.0000161501.65890.22) | Excluded | No eating behaviour measures/outcomes |
|  | #870 | Kluge et al., 2013 | Substantial weight gain associated with severe carbohydrate craving in a patient receiving quetiapine | DOI: [10.1111/pcn.12033](https://dx.doi.org/10.1111/pcn.12033) | Excluded | >25% sample eating disorders, autism, dementia or severe or profound learning disability |
|  | #5696 | Krysta et al., 2023 | Biopsychosocial Variables in Male Schizophrenic Patients: A Comprehensive Comparison with Healthy Controls | DOI: [10.3390/ph16121633](https://dx.doi.org/10.3390/ph16121633) | Excluded | Data of interest not reported |
|  | #972 | Lin et al., 2005 | Management of atypical antipsychotic-induced weight gain in schizophrenic patients with topiramate | DOI: [10.1111/j.1440-1819.2005.01424.x](https://dx.doi.org/10.1111/j.1440-1819.2005.01424.x) | Excluded | Wrong topic |
|  | #981 | Litten et al., 2012 | A double-blind, placebo-controlled trial to assess the efficacy of quetiapine fumarate XR in very heavy-drinking alcohol-dependent patients | DOI: [10.1111/j.1530-0277.2011.01649.x](https://dx.doi.org/10.1111/j.1530-0277.2011.01649.x) | Excluded | Wrong topic |
|  | #1011 | Lungu et al., 2013 | Neuronal correlates of appetite regulation in patients with schizophrenia: is there a basis for future appetite dysfunction? | DOI: [10.1016/j.eurpsy.2012.02.001](https://dx.doi.org/10.1016/j.eurpsy.2012.02.001) | Excluded | No eating behaviour measures/outcomes |
|  | #1026 | Machielsen et al., 2018 | Comparing the effect of clozapine and risperidone on cue reactivity in male patients with schizophrenia and a cannabis use disorder: A randomized fMRI study | DOI: [10.1016/j.schres.2017.03.030](https://dx.doi.org/10.1016/j.schres.2017.03.030) | Excluded | No eating behaviour measures/outcomes |
|  | #1024 | Machielsen et al., 2014 | The effect of clozapine and risperidone on attentional bias in patients with schizophrenia and a cannabis use disorder: An fMRI study | DOI: [10.1177/0269881114527357](https://dx.doi.org/10.1177/0269881114527357) | Excluded | Wrong topic |
|  | #4825 | McDevitt et al., 2006 | Perceptions of Barriers and Benefits to Physical Activity Among Outpatients in Psychiatric Rehabilitation | DOI: [10.1111/j.1547-5069.2006.00077.x](https://dx.doi.org/10.1111/j.1547-5069.2006.00077.x) | Excluded | Data of interest not reported |
|  | #1108 | Meehan et al., 2011 | Consumer strategies for coping with antipsychotic medication side effects | DOI: [10.3109/10398562.2010.539612](https://dx.doi.org/10.3109/10398562.2010.539612) | Excluded | No eating behaviour measures/outcomes |
|  | #1197 | Natan et al., 2014 | [Nutritional assessment of inpatients with schizophrenia] | No DOI | Excluded | Not in English |
|  | #1200 | Neill, 2002 | Antipsychotic drug effects on appetite and satiety- mechanisms of action | No DOI | Excluded | Abstract only |
|  | #5246 | Nicol et al., 2022 | Use of an Interactive Obesity Treatment Approach in Individuals With Severe Mental Illness: Feasibility, Acceptability, and Proposed Engagement Criteria | DOI: [10.2196/38496](https://dx.doi.org/10.2196/38496) | Excluded | >25% sample eating disorders, autism, dementia or severe or profound learning disability |
|  | #1236 | Oriaku, 2020 | Antipsychotic medication and weight gain among inpatient psychiatric clients | Dissertation Abstracts International: Section B: The Sciences and Engineering 2020;81(12-B):No-Specified  US ProQuest Information & Learning US 2020 | Excluded | No eating behaviour measures/outcomes |
|  | #1246 | Ozenoglu et al., 2007 | Nutritional approach to metabolic changes arising out of schizophrenia therapy: Case report | DOI: [10.2169/internalmedicine.46.6323](https://dx.doi.org/10.2169/internalmedicine.46.6323) | Excluded | Wrong topic |
|  | #1267 | Park et al., 2011 | Description of a healthy lifestyle intervention for people with serious mental illness taking second-generation antipsychotics | DOI: [10.1111/j.1447-0349.2011.00747.x](https://dx.doi.org/10.1111/j.1447-0349.2011.00747.x) | Excluded | Not primary research |
|  | #1277 | Patri et al., 2000 | Weight gain and antipsychotic treatment  Prise de poids et traitement antipsychotique | No DOI | Excluded | Not in English |
|  | #1278 | Patri et al., 2000 | Weight gain and antipsychotic treatment  Prise de poids et traitement antipsychotique | No DOI | Excluded | Not in English, duplicate |
|  | #1281 | Pearsall et al., 2014 | Understanding the problems developing a healthy living programme in patients with serious mental illness: a qualitative study | DOI: [10.1186/1471-244X-14-38](https://dx.doi.org/10.1186/1471-244X-14-38) | Excluded | Wrong topic |
|  | #1283 | Peet, 2004 | Diet, diabetes and schizophrenia: review and hypothesis | DOI: [10.1192/bjp.184.47.s102](https://dx.doi.org/10.1192/bjp.184.47.s102) | Excluded | Not primary research |
|  | #1287 | Pendlebury et al., 2005 | Evaluation of a behavioural weight management programme for patients with severe mental illness: 3 year results | DOI: [10.1002/hup.707](https://dx.doi.org/10.1002/hup.707) | Excluded | Wrong topic |
|  | #4800 | Piparva et al., 2011 | Analysis of Adverse Drug Reactions of Atypical Antipsychotic Drugs in Psychiatry OPD | DOI: [10.4103/0253-7176.92067](https://dx.doi.org/10.4103/0253-7176.92067) | Excluded | Data of interest not reported |
|  | #1312 | Pooley et al., 2004 | A 5-HT2C receptor promoter polymorphism (HTR2C-759C/T) is associated with obesity in women, and with resistance to weight loss in heterozygotes | DOI: [10.1002/ajmg.b.20143](https://dx.doi.org/10.1002/ajmg.b.20143) | Excluded | No eating behaviour measures/outcomes |
|  | #1331 | Poyurovsky et al., 2007 | Attenuating effect of reboxetine on appetite and weight gain in olanzapine-treated schizophrenia patients: a double-blind placebo-controlled study | DOI: [10.1007/s00213-007-0731-1](https://dx.doi.org/10.1007/s00213-007-0731-1) | Excluded | Wrong topic |
|  | #1333 | Poyurovsky et al., 2003 | Attenuation of olanzapine-induced weight gain with reboxetine in patients with schizophrenia: A double-blind, placebo-controlled study | DOI: [10.1176/appi.ajp.160.2.297](https://dx.doi.org/10.1176/appi.ajp.160.2.297) | Excluded | No eating behaviour measures/outcomes |
|  | #1364 | Ramacciotti et al., 2004 | Schizophrenia and Binge-Eating Disorders | DOI: [10.4088/JCP.v65n0720a](https://dx.doi.org/10.4088/JCP.v65n0720a) | Excluded | Data of interest not reported |
|  | #1397 | Robinson, 2008 | Appetite regulation: Hormones and antipsychotics | No DOI | Excluded | Not primary research |
|  | #1410 | Roick et al., 2007 | Health habits of patients with schizophrenia | DOI: [10.1007/s00127-007-0164-5](https://dx.doi.org/10.1007/s00127-007-0164-5) | Excluded | <75% sample prescribed antipsychotics |
|  | #1411 | Roick et al., 2008 | Health habits of patients with schizophrenia: a general pattern? | No DOI | Excluded | <75% sample prescribed antipsychotics |
|  | #1412 | Rojas et al., 2009 | [Atypical antipsychotic induced weight gain and metabolic disorders] | DOI: <https://dx.doi.org/S0034-98872009000100017> | Excluded | Not in English |
|  | #1434 | Ryu et al., 2016 | Association Study of 60 Candidate Genes with Antipsychotic-induced Weight Gain in Schizophrenia Patients | DOI: [10.1055/s-0035-1569267](https://dx.doi.org/10.1055/s-0035-1569267) | Excluded | No eating behaviour measures/outcomes |
|  | #1436 | Ryu et al., 2013 | Eating-Behavior Changes Associated With Antipsychotic Medications in Patients With Schizophrenia as Measured by the Drug-Related Eating Behavior Questionnaire | DOI: [10.1097/JCP.0b013e31827c2e2d](https://dx.doi.org/10.1097/JCP.0b013e31827c2e2d) | Excluded | Data of interest not reported |
|  | #1451 | Sanlier et al., 2019 | The evaluation on metabolic syndrome and nutrition in patients with schizophrenia | DOI: [10.23751/pn.v21i1-S.5938](https://dx.doi.org/10.23751/pn.v21i1-S.5938) | Excluded | Wrong topic |
|  | #1474 | Scheffler et al., 2018 | Effects of cannabis use on body mass, fasting glucose and lipids during the first 12 months of treatment in schizophrenia spectrum disorders | DOI: [10.1016/j.schres.2018.02.050](https://dx.doi.org/10.1016/j.schres.2018.02.050) | Excluded | Wrong topic |
|  | #2385 | Sevillano-Jimenez et al., 2022 | Nutritional Impact and Eating Pattern Changes in Schizophrenic Spectrum Disorders after Health Education Program on Symbiotic Dietary Modulation Offered by Specialised Psychiatric Nursing-Two-Arm Randomised Clinical Trial | DOI: [10.3390/nu14245388](https://dx.doi.org/10.3390/nu14245388) | Excluded | <75% sample prescribed antipsychotics |
|  | #1509 | Sharma et al., 2015 | Early increase in appetite - a predictor of response to antipsychotics | No DOI | Excluded | Abstract only |
|  | #1521 | Simonelli-Munoz et al., 2012 | Dietary habits of patients with schizophrenia: A self-reported questionnaire survey | DOI: [10.1111/j.1447-0349.2012.00821.x](https://dx.doi.org/10.1111/j.1447-0349.2012.00821.x) | Excluded | Wrong topic |
|  | #1549 | Smith et al., 2020 | Early Intervention in Psychosis: Effectiveness and Implementation of a Combined Exercise and Health Behavior Intervention Within Routine Care | DOI: [10.3389/fendo.2020.577691](https://dx.doi.org/10.3389/fendo.2020.577691) | Excluded | No eating behaviour measures/outcomes |
|  | #1550 | Smith et al., 2018 | Betahistine effects on weight-related measures in patients treated with antipsychotic medications: A double-blind placebo-controlled study | DOI: [10.1007/s00213-018-5079-1](https://dx.doi.org/10.1007/s00213-018-5079-1) | Excluded | No eating behaviour measures/outcomes |
|  | #5693 | Stassen et al., 2024 | Polypharmacy in psychiatry and weight gain: longitudinal study of 832 patients hospitalized for depression or schizophrenia, along with data of 3180 students from Europe, the US, South America, and China | DOI: [10.1007/s00406-024-01767-2](https://dx.doi.org/10.1007/s00406-024-01767-2) | Excluded | >25% sample eating disorders, autism, dementia or severe or profound learning disability |
|  | #1591 | Stauffer et al., 2009 | Predictors and correlates for weight changes in patients co-treated with olanzapine and weight mitigating agents; a post-hoc analysis | DOI: [10.1186/1471-244X-9-12](https://dx.doi.org/10.1186/1471-244X-9-12) | Excluded | Not primary research |
|  | #1599 | Stiles-Shields et al., 2013 | An Examination of Adults on Antipsychotic Medication at Risk for Metabolic Syndrome: A Comparison with Obese and Eating Disorder Populations | DOI: [10.1002/erv.2200](https://dx.doi.org/10.1002/erv.2200) | Excluded | <75% sample prescribed antipsychotics |
|  | #4818 | Strassnig et al., 2003 | Nutritional Assessment of Patients With Schizophrenia: A Preliminary Study | DOI: [10.1093/oxfordjournals.schbul.a007013](https://dx.doi.org/10.1093/oxfordjournals.schbul.a007013) | Excluded | Data of interest not reported |
|  | #1605 | Strassnig et al., 2005 | Dietary fatty acid and antioxidant intake in community-dwelling patients suffering from schizophrenia | DOI:10.1016/j.schres.2005.03.002 | Excluded | No eating behaviour measures/outcomes |
|  | #4886 | Tagami et al., 2016 | The atypical antipsychotic, olanzapine, potentiates ghrelin-induced receptor signaling: An in vitro study with cells expressing cloned human growth hormone secretagogue receptor | DOI: [10.1016/j.npep.2015.12.010](https://dx.doi.org/10.1016/j.npep.2015.12.010) | Excluded | Wrong topic |
|  | #1638 | Teasdale et al., 2016 | A nutrition intervention is effective in improving dietary components linked to cardiometabolic risk in youth with first-episode psychosis | DOI: [10.1017/S0007114516001033](https://dx.doi.org/10.1017/S0007114516001033) | Excluded | >25% sample eating disorders, autism, dementia or severe or profound learning disability |
|  | #4807 | Teasdale et sl., 2018 | Is Obesity in Young People With Psychosis a Foregone Conclusion? Markedly Excessive Energy Intake Is Evident Soon After Antipsychotic Initiation | DOI: [10.3389/fpsyt.2018.00725](https://dx.doi.org/10.3389/fpsyt.2018.00725) | Excluded | >25% sample eating disorders, autism, dementia or severe or profound learning disability |
|  | #1650 | Terao et al., 2008 | Unusual weight fluctuation under corticosteroid and psychotropic treatment | DOI: [10.1111/j.1440-1819.2008.01837.x](https://dx.doi.org/10.1111/j.1440-1819.2008.01837.x) | Excluded | Wrong topic |
|  | #1652 | Theisen et al., 2003 | Spectrum of binge eating symptomatology in patients treated with clozapine and olanzapine | DOI: [10.1007/s00702-002-0792-6](https://dx.doi.org/10.1007/s00702-002-0792-6) | Excluded | Data of interest not reported |
|  | #1654 | Theleritis et al., 2006 | Excessive weight gain after remission of depression in a schizophrenic patient treated with risperidone: case report | DOI: [10.1186/1471-244X-6-37](https://dx.doi.org/10.1186/1471-244X-6-37) | Excluded | No eating behaviour measures/outcomes |
|  | #1663 | Togo et al., 2004 | Serum ghrelin concentrations in patients receiving olanzapine or risperidone | DOI: [10.1007/s00213-003-1642-4](https://dx.doi.org/10.1007/s00213-003-1642-4) | Excluded | No eating behaviour measures/outcomes |
|  | #1686 | Tuncer et al., 2020 | An examination of emotional eating behavior in individuals with a severe mental disorder | DOI: [10.1016/j.apnu.2020.10.002](https://dx.doi.org/10.1016/j.apnu.2020.10.002) | Excluded | >25% sample eating disorders, autism, dementia or severe or profound learning disability |
|  | #4826 | Tweedell et al., 2004 | Managing neuroleptic weight gain: Consumers’ perspectives | No DOI | Excluded | Data of interest not reported |
|  | #1754 | Wang et al., 2012 | Both physical activity and food intake are associated with metabolic risks in patients with schizophrenia | DOI: [10.1016/j.schres.2012.05.008](https://dx.doi.org/10.1016/j.schres.2012.05.008) | Excluded | No eating behaviour measures/outcomes |
|  | #1771 | Weinbrenner et al., 2009 | Risperidone-associated increase in triglyceride levels | DOI: [10.1176/appi.ajp.2008.08081169](https://dx.doi.org/10.1176/appi.ajp.2008.08081169) | Excluded | No eating behaviour measures/outcomes |
|  | #4824 | Weissman et al., 2006 | Letters | DOI: 10.1176/ps.2006.57.5.724 | Excluded | Data of interest not reported |
|  | #1777 | Werneke et al., 2013 | Behavioral Interventions for Antipsychotic Induced Appetite Changes | DOI: 10.1007/s11920-012-0347-y | Excluded | Not primary research |
|  | #1837 | Yarborough et al., 2016 | Improving Lifestyle Interventions for People With Serious Mental Illnesses: Qualitative Results From the STRIDE Study | DOI: 10.1037/prj0000151 | Excluded | >25% sample eating disorders, autism, dementia or severe or profound learning disability |
|  | #1849 | Yum et al., 2021 | Atypical Antipsychotics are Disproportionately Associated with Patients Reporting Increased Eating Drives | DOI: 10.30773/pi.2020.0431 | Excluded | Data of interest not reported |
