# Supplementary material for: The impacts of antipsychotic medications on eating-related outcomes: A mixed methods systematic review
Source: PLoS One. 2025 Feb 3;20(2):e0308037. doi: 10.1371/journal.pone.0308037 (PMC11790239; doi:10.1371/journal.pone.0308037)
Supplement: S7 File — (DOCX) [file pone.0308037.s007.docx]

**S7 File. Scales/questionnaires used to assess eating-related outcomes.**

| **Scale/Questionnaire** | **Citation** | **Main academic publication** | **Brief overview** |
| --- | --- | --- | --- |
| **Appetite as a composite variable** | | | |
| Platypus Appetite Rating Scale (PARS) | **(Case et al., 2010; Karagianis et al., 2009)** | NA | - Developed by a pharmaceutical company (Lilly). - VAS (no further details provided). - Score increase was defined as ≥ 5 units on PARS; no change in score was defined as ≥-5 to ≤+5 units on PARS; score decrease was defined as < -5 units on PARS. - Psychometric properties not tested. |
| Visual analogue scale (VAS) | **(Bitter et al., 2010)** | NA | - Used to rate appetite/hunger (unclear); measured on a line with scores ranging from 0 to 100. |
| VAS | **(Park et al., 2013)** | NA | - 10-cm line method used to rate subjective hunger, fullness, desire to eat and prospective food consumption **collectively**. - Mean appetite scores were calculated, with higher scores indicating greater subjective appetite. |
| VAS | **(Ballon et al., 2018)** | NA | - 100 mm visual analogue rating scales indicating hunger, fullness, desire to eat. |
| VAS | **(Roerig et al., 2005)** | NA | - 100-mm line method used to rate hunger/appetite for breakfast, lunch and dinner time points during the feeding laboratory assessments. |
| VAS | **(Smith et al., 2012)** | NA | - 100-cm line method used to rate hunger, interest in food, and appetite **collectively** over the previous 24 hours. |
| VAS | **(Teff et al., 2015)** | NA | - Used to rate hunger and fullness daily, immediately before and following each meal. Participants were asked: “how hungry do you feel right now?” and “how full do you feel right now?” - Scores ranged on a VAS from 1 to 9 (1= not at all, 9 = extremely). |
| Questionnaire developed by authors | **(Huang et al., 2020)** | NA | - Appetite was physician-assessed daily, 30 min before lunch, with 4 standardised questions: Hungry; felt full; thinking about food; and overeating. - Grading: responses were rated on a scale from 0 to 10 (0 = not at all, 10 = extremely). - Appetite increase was defined as a >10% increases in baseline appetite scores. - Appetite decrease was defined as a >10% decreases in baseline appetite scores. |
| VAS | **(Blouin et al., 2008)** | NA | - 150-mm line method used to rate hunger and prospective food consumption before, and after the standardised breakfast test, and immediately before and after the ad-libitum buffet. - Questions used: How hungry do you feel? (ranging from ‘not hungry at all’ to ‘as hungry as I ever felt’) and how much food do you think you could eat? (ranging from ‘nothing at all’ to ‘a large amount’). - The appetite sensation responses to the standardised meal test were evaluated by calculating the area under the curve for the 1-h postprandial period. |
| Questionnaire developed by authors | **(Mathews et al., 2012)** | NA | - Hunger measured using a 5-point hunger rating scale ranging from 0 (not hungry) to 4 (very hungry), |
| Questionnaire developed by authors | **(Yang et al., 2021)** | NA | - 3-point Likert self-report scale (1= increased appetite, 2= decreased appetite, 3= no change in appetite. - Patients were asked to compare changes in the past week with changes in the week before. |
| Adapted version of the North American survey to assess subjective experiences of clozapine treatment (Waserman and Criollo, 2000) | **(Qurashi et al., 2015; Srour et al., 2023)** | (Waserman and Criollo, 2000) | - 27-item questionnaire used to assess subjective experiences; one of which is the effect of the medication on appetite. - Participants were asked to rate side effects and satisfaction with clozapine in comparison to previously prescribed APs on a 5-point Likert scale (much worse, worse, no different, better, much better), and to rate the effects experienced in terms of hedonic response (how they felt about them) on a 5-point Likert scale (very unhappy about it, unhappy about it, don’t mind either way, happy about it, very happy about it). - Items adapted from a North American survey (Waserman and Criollo, 2000). |
| **Craving** | | | |
| Food Craving Inventory (FCI) | **(Abbas and Liddle, 2013; Case et al., 2010; Garriga et al., 2019; Hoffman et al., 2009)** | (White et al., 2002) | - 28-item self-rated questionnaire measuring general and specific food cravings (high fats, carbohydrates/starches, sweets, and fast-food fats) over the past month. - Starts with a definition of craving “an intense desire for a specific food that is difficult to resist”, followed by a question: ‘Over the past month, how often you have experienced a craving for the food?’ and a list of 28 food items. - Grading: intensity of carving is rated using a 5-point Likert scale (1 = never, 5 = always/almost every day). - The general food craving score is the average of craving scores for the 28 food items. - Craving for specific food categories is calculated as the average of craving scores for food items included in each category. - Psychometrically validated (Martin, 2001; White and Grilo, 2005). - In the (Abbas and Liddle, 2013) study, names of specific food items were changed to be make it more relevant to the UK sample: ‘French fries’ to ‘chips’, ‘Cinnamon rolls’ to ‘Danish pastries’, ‘cookies’ to ‘biscuits’, ‘brownies’ to ‘chocolate cake’ and ‘chips’ to ‘crisps’. - In the (Garriga et al., 2019) study, the Spanish version of the FCI (FCI-SP) was used. It covers 28 Spanish sociocultural-adapted food types; its psychometric properties have been tested (Jauregui Lobera et al., 2010). - FCI-SP consists of 3 subcategories: complex carbohydrates/proteins, simple sugars/trans-fat, and saturated fats/high caloric content (fast food). |
| **Eating cognitions/behaviours** | | | |
| Eating Attitudes Test (EAT-26) | **(Khosravi, 2020)** | (Garner and Garfinkel, 1979; Garner et al., 1982) | - 26-item questionnaire that includes questions on dieting, bulimia and food preoccupation, and oral control. - EAT-26 (Garner et al., 1982) is a refinement of the original EAT-40 (Garner and Garfinkel, 1979), which was designed to assess symptoms of anorexia nervosa. - Freely available for download <https://psychology-tools.com/eat-26/>. - Consists of 3 sections: (a) BMI, (b) 26 items rated on a 6-point Likert scale based on how often the individual engages in specific behaviours (Always, Usually, Often, Sometimes, Rarely, and Never), and (c) 5 behavioural questions related to eating symptoms and weight loss. - Across the 26 items, Always = 3, Usually = 2, Often = 1, and Other answers = 0. - Psychometrically tested. - The Persian version (Gargari et al., 2010) of the EAT-26 was used in the (Khosravi, 2020) study. - Total score of ≥ 20 in the study represented disordered eating behaviours (DEB)s. |
| Eating Inventory (EI)/Three-Factor Eating Questionnaire (TFEQ) | **(Blouin et al., 2008; Case et al., 2010; Hoffman et al., 2009; Khazaal et al., 2009; Mathews et al., 2012; Sentissi et al., 2009; Stip et al., 2012)** | (Stunkard and Messick, 1985) | - Developed by Stunkard and Messick in 1985. It was first called the Three-Factor Eating Questionnaire, then renamed the Eating Inventory in 1988 by its authors. Sometimes referred to as the Stunkard-Messick Eating Questionnaire... - 51-item self-rated questionnaire used to assess 3 cognitive and behavioural domains (or ‘factors’) of eating: cognitive dietary restraint (cognitive restraint of food intake), disinhibition (lack of control over food intake) and hunger (susceptibility to hunger). - It consists of 3 subscales: cognitive restraint scale (TFEQ-R; 21 items), disinhibition scale (TFEQ-D; 16 items), and hunger scale (TFEQ-H; 14 items). - Grading: each item is scored as 0 or 1. - In the (Blouin et al., 2008) study, the researchers investigated these 3 factors further by using Bond et al. (2001) subcategories. - Cognitive dietary restraint was divided into 3 subscales: strategic dieting behaviours (specific behaviours used to control weight; 4 items), attitude to self-regulation (an individual’s perspective on eating and weight control; 5 items), and avoidance of fattening food (4 items). - Disinhibition was divided into 3 subscales: habitual susceptibility to disinhibition (behaviour associated with circumstances that may predispose recurrent disinhibition; 5 items; scores ranging from 0 to 5), emotional (behaviour associated with negative affective states; 3 items; scores ranging from 0 to 3), and situational susceptibility to disinhibition (overeating initiated by specific environmental cues; 5 items; scores ranging from 0 to 5). - Susceptibility to hunger is divided into 2 specific sub-scores (internal and external locus for hunger; 6 items each; scores ranging from 0 to 6). Internal hunger refers to hunger that is interpreted and regulated internally, whereas external hunger is triggered by environmental cues. - Psychometric properties tested. - French version of the TFEQ was used in the study by Sentissi et al. (2009). |
| Three-Factor Eating Questionnaire-Revised 21-Item Version (TFEQ-R21) | **(Kang et al., 2024; Kouidrat et al., 2018)** | (Cappelleri et al., 2009; Karlsson et al., 2000) | - 21-item self-rated questionnaire used to assess 3 cognitive and behavioural domains (or ‘factors’) of eating: cognitive dietary restraint (6 items), emotional eating (overeating behaviour associated with negative affective states; 6 items), and uncontrolled eating (tendency to lose control overeating when feeling hungry or when exposed to external stimuli; 9 items). - Grading: scores for each factor range from 0 to 100. Higher scores indicate higher levels of the factor. - Psychometric properties tested. |
| Dutch Eating Behavior Questionnaire (DEBQ) | **(Sentissi et al., 2009)** | (van Strien et al., 1986) | - 33-item self-report questionnaire used to assess 3 eating behaviours: emotional eating (tendency to increased food intake in response to negative stimuli; 13 items), external eating (reflects vulnerability to external triggers of eating behaviour; 10 items), and restrained eating (similar to cognitive dietary restrain in TEFQ; 10 items). - Grading: rated using a 5-point Likert scale (1= never, 5= very often). - Validated for the French population (Lluch et al., 1996). |
| Revised version of the Mizes Anorectic Cognitions questionnaire (MAC-R) | **(Khazaal et al., 2007; Khazaal et al., 2006)** | (Mizes et al., 2000) | - 24-item self-rated questionnaire used to assess eating disorder cognitions. - Divided into 3 subscales: (1) rigid weight regulation and fear of weight gain (RWFW), (2) self-control as the basis of self-esteem (SCSE), and (3) Weight and eating behaviour as the basis of approval from Others (WAFO). - Each subscale consists of 8 items; each item is rated using a 5-point Likert scale ranging from 1 to 5 (1 = strongly disagree, 5 = strongly agree). *N.B.: Dietary restraint as measured by TFEQ reflects behavioural mechanisms for restraining food intake which differs from the cognitive restraint dimension (the intention to consciously restrict food intake) of the MAC-R (Stunkard and Messick, 1985).* - Psychometric properties of MAC-R were tested in clinical (Carrard et al., 2017; Mizes et al., 2000) and non-clinical (Osman et al., 2001)populations |
| **Collective assessment tools** |  |  |  |
| Eating Behavior Assessment (EBA) | **(Case et al., 2010) Study 1 (Hardy et al., 2009); (Smith et al., 2012)** | NA | - Developed by a pharmaceutical company (Lilly). - 9-item questionnaire - Measures appetite, hunger, craving, fullness and eating behaviour collectively. - Questions included: How hungry? Appetite? Craved sweets? Craved fatty food? Felt full? Ate excessive amount? Thinking of food? Overeating? Out of control eating? - Grading responses on a scale from 0 to 4, where 0 = not at all and 4 = extremely - Score increase was defined as positive value on EBA; no change in score was defined as 0 units on EBA; score decrease was defined as negative value on EBA (Case et al., 2010). - Psychometric properties not tested. |
| Eating Appetite Scale/Eating Attitude Scale (EAS) | **(Case et al., 2010; Treuer et al., 2009)** |  | - Developed by a pharmaceutical company (Lilly). - Psychometric properties not tested. - Number of items: unclear. The Case et al. (2010) paper stated that EAS consists of 10 standardised categories. However, data for only 7 items were presented. The paper by Treuer et al. (2009) stated that EAS is a 7-item, physician-rated questionnaire measuring changes in appetite, hunger, fullness and eating behaviours **collectively** over the past 4 weeks. - Questions included in the EAS: Were you more hungry than usual? Did you have a stronger appetite than usual? Were you comfortably full when meals were finished? Did you require an excessive amount of food to feel full? Were your thoughts preoccupied with food? Did you eat until uncomfortably full? Did you feel you could not stop eating or control how much was eaten? - There were 5 possible responses to these questions: never; seldom; sometimes; often; or always. - Score increase was defined as > 0 units on EAS; no change in score was defined as 0 units on EAS; score decrease was defined as < 0 units on EAS (Case et al., 2010). |
| **Food intake** |  |  |  |
| ***Food frequency questionnaires*** | | | |
| Dietary Fat Screener and the Fruit and Vegetable and Fibre Screener | **(Archie et al., 2007)** | (Block et al., 2000) | - 22-item, self-rated food frequency questionnaire (FFQ) measuring dietary fats, and fruit and vegetable intake (including fibre and micronutrients found in fruits and vegetables). |
| Cuestionario de Frecuencia de Consumo de Alimentos (CFCA) | **(Garriga et al., 2019)** | (Rodriguez et al., 2008) | - 45-item self-administered food frequency questionnaire (FFQ) measuring dietary habits in the Spanish general population. - Psychometrically tested. |
| Block Food Frequency Questionnaire (FFQ) | **(Henderson et al., 2006)** | (Block et al., 1986) | - The food frequency questionnaire (FFQ) provides information on nutrient intake, food groups and dietary habits. - Interviewers asked the frequency and portion size of specific food items consumed over the past month. |
| Adapted Dietary Instrument for Nutrition Education (DINE) questionnaire | **(Holt et al., 2018)** | (Roe et al., 1994) | - Self-reported questionnaire that measures 6 items: intake of fibre, fat, unsaturated fat, sugar, alcohol and meal type. |
| Food Frequency Questionnaire (FFQ) dietary quality score (DQS) | **(Jakobsen et al., 2018a; Jakobsen et al., 2018b)** | (Toft et al., 2007) | - 48-item food frequency questionnaire (FFQ) included questions about type of bread, spread and fats used for cooking, frequency of consumption of 27 food items during the last week. - The dietary quality score (DQS) was calculated using the food frequency questionnaire (FFQ) (Toft et al., 2007), based on a 3-point scoring system that was developed for each of the 4 food groups: fish, fruits, vegetables, fats (see Supplementary Table 2 in paper). - A higher dietary quality score (DQS) was associated with higher dietary quality, including low fat intake (especially saturated fat), high fibre intake; intake of vitamins and minerals; and fruit, fish, vegetables and whole-grain products. |
| Food frequency questionnaire | **(Lappin et al., 2018)** | NA | - 10-item, semi-structured, picture-guided, food intake questionnaire. - ‘Developed by 3 mental health dietitians and a researcher experienced in validation of dietary assessment tools.’ (unpublished). - Not psychometrically tested. - Evaluates intake of 6 ‘healthy’, 4 ‘unhealthy’ food categories. Healthy food categories: fruit, vegetables, wholegrain foods, unsweetened dairy/alternatives, healthy fats, protein foods. Unhealthy food categories: sugary drinks, sweet foods, savoury discretionary foods, alcoholic beverages. - Likert-scale responses: i) < 1/week, ii) multiple times/week, iii) once/day, iv) multiple times/day. |
| Food frequency questionnaire | **(Morell et al., 2019)** | NR | - 10-item picture-guided food intake questionnaire developed to evaluate food consumption patterns in people with mental illness. - The questionnaire assessed the frequency of intake of 5 core foods (fruit, vegetables, wholegrain foods, unsweetened dairy and alternatives, protein) and 5 non-core foods (unhealthy fats, sugary drinks, sweet foods, savoury discretionary foods, alcoholic beverages). - Participants were asked to consider average intake over the last month and select from multiple choice responses; (i) <1/week, (ii) multiple times/week, (iii) 1/day, and (iv) multiple times/day. |
| Food frequency questionnaire | **(Nunes et al., 2014)** | (Zanolla et al., 2009) | - Psychometrically tested food frequency question containing 127 food items, considered suitable for use in the Brazilian population. |
| Mediterranean diet score (MedDietScore) questionnaire | **(Ntalkitsi et al., 2022)** | (Panagiotakos et al., 2006) | - Dietician-administered questionnaire used to record the weekly frequency of consumption of potatoes, unrefined grains, legumes, vegetables, fruit, red meat, poultry, fish, dairy, olive oil, and alcoholic beverages in 6 categories ranging from rarely to daily. |
| European Prospective Investigation into Cancer and Nutrition Questionnaire (EPIC) | **(Saugo et al., 2020)** | (Pisani et al., 1997) | - Semiquantitative questionnaire composed of 248 items investigating the consumption of foods, group of foods, macro and micronutrients; refers to the eating habits over the 12 months preceding administration. |
| Frequency of food consumption | **(Treuer et al., 2009)** | NA | - Physician-administered questionnaire to assess frequency of consumption of specific food groups (several times a day; once a day; every few days; or never or rarely). - Meal regularity (regular or irregular), average number of meals consumed each day, and snack consumption at certain times of the day were also assessed. |
| ***Food records*** | | | |
| Four-day dietary record | **(Henderson et al., 2006)** |  | - Participants recorded their food and beverage consumption for 4 consecutive days (3 weekdays, 1 weekend day). - Estimated intake of individual nutrient totals were calculated using the Minnesota Nutrient Database (NDS). |
| Record for 7 consecutive days | **(Jakobsen et al., 2018b)** |  | - Participants kept a food record for 7 consecutive days in a pre-coded (semi-closed) questionnaire with answering categories for the most commonly consumed foods and dishes in the Danish diet. |
| Recording food consumption over 3 consecutive days | **(Saugo et al., 2020)** |  | - Participants kept a food record for 3 consecutive days on hard-copy diaries structured by meal (Leclercq et al., 2009) |
| Four-day dietary record | **(Henderson et al., 2010)** |  | - Participants recorded their food and beverage consumption for 4 consecutive days (3 weekdays and 1 weekend day). - Estimated intake of individual nutrient totals were calculated by the University of Minnesota Nutrition Data System for Research software (version 2.6; Food Database 6A; Nutrient Database 23; Nutrition Coordinating Centre, University of Minnesota, Minneapolis, MN). |
| ***Recall method*** | | | |
| 24-hour dietary recall method | **(Daurignac et al., 2015; Henderson et al., 2006; Jakobsen et al., 2018b; Kurpad et al., 2010; Ntalkitsi et al., 2022; Stefanska et al., 2017; Stefanska et al., 2018)** | NA | - Participants were asked to recall food intake on a typical day. - A software programme was used to calculate energy and macronutrient intake. |
| **Eating disorders** | | | |
| Questionnaire developed by authors | **(Kurpad et al., 2010)** | NA | - Eating behaviours, binge eating, binge eating disorder (BED): assessed using a questionnaire developed by the authors that checks the presence/absence of DSM-IV criteria for BED. - Psychometric properties not tested. |
| Yale Food Addiction Scale (YFAS) | **(Goluza et al., 2017)** | (Gearhardt et al., 2009) | - 25-item, self-report questionnaire used to assess the presence of food addiction (FA) based on the 7 substance dependence criteria in DSM-5. - Participants were instructed to refer to the past 12 months when answering questions. - FA diagnosis: ≥3 symptoms of addiction met in the presence of clinically significant impairment or distress. - Questionnaire items available in the paper (table 2). - Psychometrically validated (Meule and Gearhardt, 2019). |

DSM-5= Diagnostic and Statistical Manual of Mental Disorders, Fifth Edition; FCI-SP= Spanish version of the Food Craving Inventory; NA= not applicable; NR= not reported; VAS= Visual analogue scale.

**References**

Abbas, M. J. & Liddle, P. F. (2013). ‘Olanzapine and food craving: A case control study’ *Hum Psychopharmacol*, 28 (1), pp. 97-101. DOI: 10.1002/hup.2278 Available at: <https://www.ncbi.nlm.nih.gov/pubmed/23169487>.

Archie, S. M., et al. (2007). ‘Psychotic disorders, eating habits, and physical activity: Who is ready for lifestyle changes?’ *Psychiatric Services*, 58 (2), pp. 233-239. DOI: 10.1176/ps.2007.58.2.233.

Ballon, J. S., et al. (2018). ‘Pathophysiology of drug induced weight and metabolic effects: Findings from an rct in healthy volunteers treated with olanzapine, iloperidone, or placebo’ *J Psychopharmacol*, 32 (5), pp. 533-540. DOI: 10.1177/0269881118754708 Available at: <https://www.ncbi.nlm.nih.gov/pubmed/29444618>.

Bitter, I., et al. (2010). ‘Patients' preference for olanzapine orodispersible tablet compared with conventional oral tablet in a multinational, randomized, crossover study’ *World J Biol Psychiatry*, 11 (7), pp. 894-903. DOI: 10.3109/15622975.2010.505663 Available at: <https://www.ncbi.nlm.nih.gov/pubmed/20653494>.

Block, G., Gillespie, C., Rosenbaum, E. H. & Jenson, C. (2000). ‘A rapid food screener to assess fat and fruit and vegetable intake’ *Am J Prev Med*, 18 (4), pp. 284-8. DOI: 10.1016/s0749-3797(00)00119-7 Available at: <https://www.ncbi.nlm.nih.gov/pubmed/10788730>.

Block, G., et al. (1986). ‘A data-based approach to diet questionnaire design and testing’ *Am J Epidemiol*, 124 (3), pp. 453-69. DOI: 10.1093/oxfordjournals.aje.a114416 Available at: <https://www.ncbi.nlm.nih.gov/pubmed/3740045> (Accessed: 4/14/2023).

Blouin, M., et al. (2008). ‘Adiposity and eating behaviors in patients under second generation antipsychotics’ *Obesity (Silver Spring)*, 16 (8), pp. 1780-7. DOI: 10.1038/oby.2008.277 Available at: <https://www.ncbi.nlm.nih.gov/pubmed/18535555>.

Bond, M. J., McDowell, A. J. & Wilkinson, J. Y. (2001). ‘The measurement of dietary restraint, disinhibition and hunger: An examination of the factor structure of the three factor eating questionnaire (tfeq)’ *Int J Obes Relat Metab Disord*, 25 (6), pp. 900-6. DOI: 10.1038/sj.ijo.0801611 Available at: <https://www.ncbi.nlm.nih.gov/pubmed/11439306>.

Cappelleri, J. C., et al. (2009). ‘Psychometric analysis of the three-factor eating questionnaire-r21: Results from a large diverse sample of obese and non-obese participants’ *Int J Obes (Lond)*, 33 (6), pp. 611-20. DOI: 10.1038/ijo.2009.74 Available at: <https://www.ncbi.nlm.nih.gov/pubmed/19399021>.

Carrard, I., Rothen, S., Kruseman, M. & Khazaal, Y. (2017). ‘Assessment of dysfunctional cognitions in binge-eating disorder: Factor structure and validity of the mizes anorectic cognitions questionnaire-revised (mac-r)’ *Front Psychol*, 8 p. 208. DOI: 10.3389/fpsyg.2017.00208 Available at: <https://www.ncbi.nlm.nih.gov/pubmed/28261139>.

Case, M., Treuer, T., Karagianis, J. & Hoffmann, V. P. (2010). ‘The potential role of appetite in predicting weight changes during treatment with olanzapine’ *BMC Psychiatry*, 10 p. 72. DOI: 10.1186/1471-244X-10-72 Available at: <https://www.ncbi.nlm.nih.gov/pubmed/20840778>.

Daurignac, E., Leonard, K. E. & Dubovsky, S. L. (2015). ‘Increased lean body mass as an early indicator of olanzapine-induced weight gain in healthy men’ *Int Clin Psychopharmacol*, 30 (1), pp. 23-8. DOI: 10.1097/YIC.0000000000000052 Available at: <https://www.ncbi.nlm.nih.gov/pubmed/25350366>.

Gargari, B. P., et al. (2010). ‘Eating attitudes, self-esteem and social physique anxiety among iranian females who participate in fitness programs’ *J Sports Med Phys Fitness*, 50 (1), pp. 79-84. Available at: <https://www.ncbi.nlm.nih.gov/pubmed/20308977>.

Garner, D. M. & Garfinkel, P. E. (1979). ‘The eating attitudes test: An index of the symptoms of anorexia nervosa’ *Psychol Med*, 9 (2), pp. 273-9. DOI: 10.1017/s0033291700030762 Available at: <https://www.ncbi.nlm.nih.gov/pubmed/472072>.

Garner, D. M., Olmsted, M. P., Bohr, Y. & Garfinkel, P. E. (1982). ‘The eating attitudes test: Psychometric features and clinical correlates’ *Psychol Med*, 12 (4), pp. 871-8. DOI: 10.1017/s0033291700049163 Available at: <https://www.ncbi.nlm.nih.gov/pubmed/6961471>.

Garriga, M., et al. (2019). ‘Food craving and consumption evolution in patients starting treatment with clozapine’ *Psychopharmacology (Berl)*, 236 (11), pp. 3317-3327. DOI: 10.1007/s00213-019-05291-3 Available at: <https://www.ncbi.nlm.nih.gov/pubmed/31197435>.

Gearhardt, A. N., Corbin, W. R. & Brownell, K. D. (2009). ‘Preliminary validation of the yale food addiction scale’ *Appetite*, 52 (2), pp. 430-6. DOI: 10.1016/j.appet.2008.12.003 Available at: <https://www.ncbi.nlm.nih.gov/pubmed/19121351>.

Goluza, I., et al. (2017). ‘Exploration of food addiction in people living with schizophrenia’ *Asian J Psychiatr*, 27 pp. 81-84. DOI: 10.1016/j.ajp.2017.02.022 Available at: <https://www.ncbi.nlm.nih.gov/pubmed/28558903>.

Hardy, T., et al. 'Insulin sensitivity in patients with schizophrenia or schizoaffective disorder treated with olanzapine or risperidone', *162nd Annual Meeting Shaping our Future: Science and Service*, San Francisco: American Psychiatric Association, pp. 14-15.

Henderson, D. C., et al. (2006). ‘Dietary intake profile of patients with schizophrenia’ *Ann Clin Psychiatry*, 18 (2), pp. 99-105. DOI: 10.1080/10401230600614538 Available at: <https://www.ncbi.nlm.nih.gov/pubmed/16754415>.

Henderson, D. C., et al. (2010). ‘Dietary saturated fat intake and glucose metabolism impairments in nondiabetic, nonobese patients with schizophrenia on clozapine or risperidone’ *Ann Clin Psychiatry*, 22 (1), pp. 33-42. Available at: <https://www.ncbi.nlm.nih.gov/pubmed/20196981>.

Hoffman, V. P., Case, M. & Jacobson, J. G. 'Algorithms including amantadine, metformin and zonisamide for mitigation of weight gain during olanzapine treatment in outpatients with schizophrenia', *APA San Francisco*

Holt, R. I., et al. (2018). ‘Structured lifestyle education to support weight loss for people with schizophrenia, schizoaffective disorder and first episode psychosis: The stepwise rct’ *Health Technol Assess*, 22 (65), pp. 1-160. DOI: 10.3310/hta22650 Available at: <https://www.ncbi.nlm.nih.gov/pubmed/30499443>.

Huang, J., et al. (2020). ‘Corrigendum: Increased appetite plays a key role in olanzapine-induced weight gain in first-episode schizophrenia patients’ *Front Pharmacol*, 11 p. 878. DOI: 10.3389/fphar.2020.00878 Available at: <https://www.ncbi.nlm.nih.gov/pubmed/32587520>.

Jakobsen, A. S., et al. (2018a). ‘Associations between clinical and psychosocial factors and metabolic and cardiovascular risk factors in overweight patients with schizophrenia spectrum disorders - baseline and two-years findings from the change trial’ *Schizophr Res*, 199 pp. 96-102. DOI: 10.1016/j.schres.2018.02.047 Available at: <https://doi.org/10.1016/j.schres.2018.02.047>.

Jakobsen, A. S., et al. (2018b). ‘Dietary patterns and physical activity in people with schizophrenia and increased waist circumference’ *Schizophr Res*, 199 pp. 109-115. DOI: 10.1016/j.schres.2018.03.016 Available at: <https://www.ncbi.nlm.nih.gov/pubmed/29555213>.

Jauregui Lobera, I., Bolanos, P., Carbonero, R. & Valero Blanco, E. (2010). ‘Psychometric properties of the spanish version of food craving inventory (fci-sp)’ *Nutr Hosp*, 25 (6), pp. 984-92. Available at: <https://www.ncbi.nlm.nih.gov/pubmed/21519770>.

Kang, D., et al. (2024). ‘The effect of continuous theta burst stimulation on antipsychotic-induced weight gain in first-episode drug-naive individuals with schizophrenia: A double-blind, randomized, sham-controlled feasibility trial’ *Transl Psychiatry*, 14 (1), p. 61. DOI: 10.1038/s41398-024-02770-w Available at: <https://www.ncbi.nlm.nih.gov/pubmed/38272892>.

Karagianis, J., et al. (2009). ‘A randomized controlled trial of the effect of sublingual orally disintegrating olanzapine versus oral olanzapine on body mass index: The platypus study’ *Schizophr Res*, 113 (1), pp. 41-8. DOI: 10.1016/j.schres.2009.05.024 Available at: <https://www.ncbi.nlm.nih.gov/pubmed/19535229>.

Karlsson, J., Persson, L. O., Sjostrom, L. & Sullivan, M. (2000). ‘Psychometric properties and factor structure of the three-factor eating questionnaire (tfeq) in obese men and women. Results from the swedish obese subjects (sos) study’ *Int J Obes Relat Metab Disord*, 24 (12), pp. 1715-25. DOI: 10.1038/sj.ijo.0801442 Available at: <https://www.ncbi.nlm.nih.gov/pubmed/11126230>.

Khazaal, Y., et al. (2009). ‘Hunger and negative alliesthesia to aspartame and sucrose in patients treated with antipsychotic drugs and controls’ *Eat Weight Disord*, 14 (4), pp. e225-30. DOI: 10.1007/BF03325121 Available at: <https://www.ncbi.nlm.nih.gov/pubmed/20179410>.

Khazaal, Y., et al. (2007). ‘Cognitive behavioural therapy for weight gain associated with antipsychotic drugs’ *Schizophr Res*, 91 (1-3), pp. 169-77. DOI: 10.1016/j.schres.2006.12.025 Available at: <https://www.ncbi.nlm.nih.gov/pubmed/17306507>.

Khazaal, Y., et al. (2006). ‘Eating and weight related cognitions in people with schizophrenia : A case control study’ *Clin Pract Epidemiol Ment Health*, 2 p. 29. DOI: 10.1186/1745-0179-2-29 Available at: <https://www.ncbi.nlm.nih.gov/pubmed/17076886>.

Khosravi, M. (2020). ‘Biopsychosocial factors associated with disordered eating behaviors in schizophrenia’ *Ann Gen Psychiatry*, 19 (1), p. 67. DOI: 10.1186/s12991-020-00314-2 Available at: <https://www.ncbi.nlm.nih.gov/pubmed/33292324>.

Kouidrat, Y., et al. (2018). ‘Disordered eating behaviors as a potential obesogenic factor in schizophrenia’ *Psychiatry Res*, 269 pp. 450-454. DOI: 10.1016/j.psychres.2018.08.083 Available at: <https://www.ncbi.nlm.nih.gov/pubmed/30195737>.

Kurpad, S. S., George, S. A. & Srinivasan, K. (2010). ‘Binge eating and other eating behaviors among patients on treatment for psychoses in india’ *Eat Weight Disord*, 15 (3), pp. e136-43. DOI: 10.1007/BF03325293 Available at: <https://www.ncbi.nlm.nih.gov/pubmed/21150249>.

Lappin, J. M., et al. (2018). ‘Cardio-metabolic risk and its management in a cohort of clozapine-treated outpatients’ *Schizophr Res*, 199 pp. 367-373. DOI: 10.1016/j.schres.2018.02.035 Available at: <https://www.ncbi.nlm.nih.gov/pubmed/29486959>.

Leclercq, C., et al. (2009). ‘The italian national food consumption survey inran-scai 2005-06: Main results in terms of food consumption’ *Public Health Nutr*, 12 (12), pp. 2504-32. DOI: 10.1017/S1368980009005035 Available at: <https://www.ncbi.nlm.nih.gov/pubmed/19278564>.

Lluch, A., et al. (1996). ‘Internal validation of a french version of the dutch eating behaviour questionnaire’ *Eur Psychiatry*, 11 (4), pp. 198-203. DOI: 10.1016/0924-9338(96)88391-X Available at: <https://www.ncbi.nlm.nih.gov/pubmed/19698450>.

Martin, C. K. (2001). *The association of food cravings and preferences with food intake* PhD, Louisiana State University and Agricultural & Mechanical College.

Mathews, J., et al. (2012). ‘Neural correlates of weight gain with olanzapine’ *Arch Gen Psychiatry*, 69 (12), pp. 1226-37. DOI: 10.1001/archgenpsychiatry.2012.934 Available at: <https://www.ncbi.nlm.nih.gov/pubmed/22868896>.

Meule, A. & Gearhardt, A. N. (2019). ‘Ten years of the yale food addiction scale: A review of version 2.0’ *Current Addiction Reports*, 6 (3), pp. 218-228. DOI: 10.1007/s40429-019-00261-3 Available at: <https://doi.org/10.1007/s40429-019-00261-3>.

Mizes, J. S., et al. (2000). ‘Development of the mizes anorectic cognitions questionnaire-revised: Psychometric properties and factor structure in a large sample of eating disorder patients’ *Int J Eat Disord*, 28 (4), pp. 415-21. DOI: 10.1002/1098-108x(200012)28:4<415::aid-eat9>3.0.co;2-z Available at: <https://www.ncbi.nlm.nih.gov/pubmed/11054788> (Accessed: 2022/12/30).

Morell, R., et al. (2019). ‘Cardio-metabolic risk in individuals prescribed long-acting injectable antipsychotic medication’ *Psychiatry Res*, 281 p. 112606. DOI: 10.1016/j.psychres.2019.112606 Available at: <https://www.ncbi.nlm.nih.gov/pubmed/31629301>.

Ntalkitsi, S., Efthymiou, D., Bozikas, V. & Vassilopoulou, E. (2022). 'Halting the metabolic complications of antipsychotic medication in patients with a first episode of psychosis: How far can we go with the mediterranean diet? A pilot study', *Nutrients*, 14(23) [Online]. DOI: 10.3390/nu14235012 Available at: <https://doi.org/10.3390/nu14235012>.

Nunes, D., et al. (2014). ‘Nutritional status, food intake and cardiovascular disease risk in individuals with schizophrenia in southern brazil: A case-control study’ *Rev Psiquiatr Salud Ment*, 7 (2), pp. 72-9. DOI: 10.1016/j.rpsm.2013.07.001 Available at: <https://www.ncbi.nlm.nih.gov/pubmed/24054065>.

Osman, A., et al. (2001). ‘Factor structure and psychometric properties of the brief mizes anorectic cognitions questionnaire’ *J Clin Psychol*, 57 (6), pp. 785-99. DOI: 10.1002/jclp.1049 Available at: <https://www.ncbi.nlm.nih.gov/pubmed/11344465> (Accessed: 2022/12/30).

Panagiotakos, D. B., Pitsavos, C. & Stefanadis, C. (2006). ‘Dietary patterns: A mediterranean diet score and its relation to clinical and biological markers of cardiovascular disease risk’ *Nutr Metab Cardiovasc Dis*, 16 (8), pp. 559-68. DOI: 10.1016/j.numecd.2005.08.006 Available at: <https://www.ncbi.nlm.nih.gov/pubmed/17126772>.

Park, S., Yi, K. K., Kim, M. S. & Hong, J. P. (2013). ‘Effects of ziprasidone and olanzapine on body composition and metabolic parameters: An open-label comparative pilot study’ *Behav Brain Funct*, 9 p. 27. DOI: 10.1186/1744-9081-9-27 Available at: <https://www.ncbi.nlm.nih.gov/pubmed/23866300>.

Pisani, P., et al. (1997). ‘Relative validity and reproducibility of a food frequency dietary questionnaire for use in the italian epic centres’ *Int J Epidemiol*, 26 Suppl 1 (suppl_1), pp. S152-60. DOI: 10.1093/ije/26.suppl_1.s152 Available at: <https://www.ncbi.nlm.nih.gov/pubmed/9126543>.

Qurashi, I., et al. (2015). ‘An evaluation of subjective experiences, effects and overall satisfaction with clozapine treatment in a uk forensic service’ *Ther Adv Psychopharmacol*, 5 (3), pp. 146-50. DOI: 10.1177/2045125315581996 Available at: <https://www.ncbi.nlm.nih.gov/pubmed/26199716> (Accessed: 2023/06/30).

Rodriguez, I. T., et al. (2008). ‘[validation of a short questionnaire on frequency of dietary intake: Reproducibility and validity]’ *Nutr Hosp*, 23 (3), pp. 242-52. Available at: <https://www.ncbi.nlm.nih.gov/pubmed/18560701>.

Roe, L., et al. (1994). ‘Dietary intervention in primary care: Validity of the dine method for diet assessment’ *Fam Pract*, 11 (4), pp. 375-81. DOI: 10.1093/fampra/11.4.375 Available at: <https://www.ncbi.nlm.nih.gov/pubmed/7895964> (Accessed: 1/5/2023).

Roerig, J. L., et al. (2005). ‘A comparison of the effects of olanzapine and risperidone versus placebo on eating behaviors’ *J Clin Psychopharmacol*, 25 (5), pp. 413-8. DOI: 10.1097/01.jcp.0000177549.36585.29 Available at: <https://www.ncbi.nlm.nih.gov/pubmed/16160615>.

Saugo, E., et al. (2020). ‘Dietary habits and physical activity in first-episode psychosis patients treated in community services. Effect on early anthropometric and cardio-metabolic alterations’ *Schizophr Res*, 216 pp. 374-381. DOI: 10.1016/j.schres.2019.11.010 Available at: <https://www.ncbi.nlm.nih.gov/pubmed/31806524>.

Sentissi, O., et al. (2009). ‘Impact of antipsychotic treatments on the motivation to eat: Preliminary results in 153 schizophrenic patients’ *Int Clin Psychopharmacol*, 24 (5), pp. 257-64. DOI: 10.1097/YIC.0b013e32832b6bf6 Available at: <https://www.ncbi.nlm.nih.gov/pubmed/19606055>.

Smith, R. C., Rachakonda, S., Dwivedi, S. & Davis, J. M. (2012). ‘Olanzapine and risperidone effects on appetite and ghrelin in chronic schizophrenic patients’ *Psychiatry Res*, 199 (3), pp. 159-63. DOI: 10.1016/j.psychres.2012.03.011 Available at: <https://www.ncbi.nlm.nih.gov/pubmed/22475524>.

Srour, A., et al. (2023). ‘Patients' and primary carers' views on clozapine treatment for schizophrenia: A cross-sectional study in qatar’ *Saudi Pharm J*, 31 (2), pp. 214-221. DOI: 10.1016/j.jsps.2022.12.005 Available at: <https://www.ncbi.nlm.nih.gov/pubmed/36942276>.

Stefanska, E., et al. (2017). ‘Eating habits and nutritional status of patients with affective disorders and schizophrenia’ *Psychiatr Pol*, 51 (6), pp. 1107-1120. DOI: 10.12740/PP/74558 Available at: <https://www.ncbi.nlm.nih.gov/pubmed/29432506>.

Stefanska, E., et al. (2018). ‘The assessment of the nutritional value of meals consumed by patients with recognized schizophrenia’ *Rocz Panstw Zakl Hig*, 69 (2), pp. 183-192. Available at: <https://www.ncbi.nlm.nih.gov/pubmed/29766697>.

Stip, E., et al. (2012). ‘Neural changes associated with appetite information processing in schizophrenic patients after 16 weeks of olanzapine treatment’ *Transl Psychiatry*, 2 (6), p. e128. DOI: 10.1038/tp.2012.53 Available at: <https://www.ncbi.nlm.nih.gov/pubmed/22714121>.

Stunkard, A. J. & Messick, S. (1985). ‘The three-factor eating questionnaire to measure dietary restraint, disinhibition and hunger’ *J Psychosom Res*, 29 (1), pp. 71-83. DOI: 10.1016/0022-3999(85)90010-8 Available at: <https://www.ncbi.nlm.nih.gov/pubmed/3981480>.

Teff, K. L., Rickels, K., Alshehabi, E. & Rickels, M. R. (2015). ‘Metabolic impairments precede changes in hunger and food intake following short-term administration of second-generation antipsychotics’ *J Clin Psychopharmacol*, 35 (5), pp. 579-82. DOI: 10.1097/JCP.0000000000000393 Available at: <https://www.ncbi.nlm.nih.gov/pubmed/26274045>.

Toft, U., et al. (2007). ‘The dietary quality score: Validation and association with cardiovascular risk factors: The inter99 study’ *Eur J Clin Nutr*, 61 (2), pp. 270-8. DOI: 10.1038/sj.ejcn.1602503 Available at: <https://www.ncbi.nlm.nih.gov/pubmed/16929244>.

Treuer, T., et al. (2009). ‘Factors associated with weight gain during olanzapine treatment in patients with schizophrenia or bipolar disorder: Results from a six-month prospective, multinational, observational study’ *World J Biol Psychiatry*, 10 (4 Pt 3), pp. 729-40. DOI: 10.1080/15622970903079507 Available at: <https://www.ncbi.nlm.nih.gov/pubmed/19606406>.

van Strien, T., Frijters, J. E. R., Bergers, G. P. A. & Defares, P. B. (1986). ‘The dutch eating behavior questionnaire (debq) for assessment of restrained, emotional, and external eating behavior’ *International Journal of Eating Disorders*, 5 (2), pp. 295-315. DOI: 10.1002/1098-108x(198602)5:2<295::Aid-eat2260050209>3.0.Co;2-t Available at: <https://doi.org/10.1002/1098-108X(198602)5:2><295::AID-EAT2260050209>3.0.CO;2-T (Accessed: 2023/04/17).

Waserman, J. & Criollo, M. (2000). ‘Subjective experiences of clozapine treatment by patients with chronic schizophrenia’ *Psychiatr Serv*, 51 (5), pp. 666-8. DOI: 10.1176/appi.ps.51.5.666 Available at: <https://www.ncbi.nlm.nih.gov/pubmed/10783189> (Accessed: 2023/07/03).

White, M. A. & Grilo, C. M. (2005). ‘Psychometric properties of the food craving inventory among obese patients with binge eating disorder’ *Eat Behav*, 6 (3), pp. 239-45. DOI: 10.1016/j.eatbeh.2005.01.001 Available at: <https://www.ncbi.nlm.nih.gov/pubmed/15854870>.

White, M. A., et al. (2002). ‘Development and validation of the food-craving inventory’ *Obes Res*, 10 (2), pp. 107-14. DOI: 10.1038/oby.2002.17 Available at: <https://www.ncbi.nlm.nih.gov/pubmed/11836456> (Accessed: 2022/12/09).

Yang, Y., et al. (2021). ‘Effect of bifidobacterium on olanzapine-induced body weight and appetite changes in patients with psychosis’ *Psychopharmacology (Berl)*, 238 (9), pp. 2449-2457. DOI: 10.1007/s00213-021-05866-z Available at: <https://www.ncbi.nlm.nih.gov/pubmed/34002246>.

Zanolla, A. F., et al. (2009). ‘[assessment of reproducibility and validity of a food frequency questionnaire in a sample of adults living in porto alegre, rio grande do sul state, brazil]’ *Cad Saude Publica*, 25 (4), pp. 840-8. DOI: 10.1590/s0102-311x2009000400015 Available at: <https://www.ncbi.nlm.nih.gov/pubmed/19347210>.
