# Supplementary material for: The impacts of antipsychotic medications on eating-related outcomes: A mixed methods systematic review
Source: PLoS One. 2025 Feb 3;20(2):e0308037. doi: 10.1371/journal.pone.0308037 (PMC11790239; doi:10.1371/journal.pone.0308037)
Supplement: S8 File — (DOCX) [file pone.0308037.s008.docx]

**S8 File. Risk of bias assessments of the 55 included quantitative studies using the Mixed Methods Assessment Tool (MMAT).**

- 1. **Randomised controlled trials**

| **CITATION** | **SCREENING QUESTIONS** | | **RANDOMISED CONTROLLED TRIALS** | | | | |
| --- | --- | --- | --- | --- | --- | --- | --- |
|  | S1. Are there clear research questions? | S2. Do the collected data allow to address the research questions? | Is randomisation appropriately performed? | Are the groups comparable at baseline? | Are there complete outcome data? | Are outcome assessors blinded to the intervention provided? | Did the participants adhere to the assigned intervention? |
| (Ballon et al., 2018) | Yes | Yes | Can't tell | Yes | No | Can't tell | Yes |
| (Bitter et al., 2010) | Yes | Yes | Yes | Yes | No | No | Yes |
| (Case et al., 2010) Study 1 (Hardy et al., 2009) | Yes | Yes | Can't tell | Yes | No | Can't tell | Can't tell |
| (Case et al., 2010) Study 2 (Karagianis et al., 2009) | Yes | Yes | Can't tell | Yes | Yes | Can't tell | Yes |
| (Case et al., 2010) Study 3 (Hoffman et al., 2009) | Yes | Yes | Can't tell | Can't tell | No | Can't tell | Can't tell |
| (Daurignac et al., 2015) | Yes | Yes | Can’t tell | Yes | No | Can’t tell | Yes |
| (Fountaine et al., 2010) | Yes | Yes | Can’t tell | Can’t tell | No | Can’t tell | Can’t tell |
| (Holt et al., 2018) | Yes | Yes | Yes | Yes | Yes | Yes | Yes |
| (Kane et al., 2001) | Yes | Yes | Can’t tell | Yes | No | Can’t tell | Yes |
| (Kang et al., 2024) | Yes | Yes | Yes | Yes | Yes | Yes | Yes |
| (Khazaal et al., 2007) | Yes | Yes | Can't tell | No | Can't tell | Can't tell | Can't tell |
| (Kluge et al., 2007) | Yes | Yes | Can't tell | Yes | Yes | Can't tell | Can't tell |
| (Park et al., 2013) | Yes | Yes | Can't tell | Yes | Yes | Can't tell | Can't tell |
| (Roerig et al., 2005) | Yes | Yes | Can't tell | Yes | No | Can't tell | Can't tell |
| (Smith et al., 2012) | Yes | Yes | Yes | Yes | Yes | Can't tell | Can't tell |
| (Teff et al., 2015; Teff et al., 2013) | Yes | Yes | Can't tell | Yes | Yes | Can't tell | Can't tell |
| (Tollefson et al., 1997) | Yes | Yes | Can’t tell | Yes | No | Can’t tell | Can’t tell |
| (Yang et al., 2021) | Yes | Yes | Can’t tell | Yes | Yes | No | Can’t tell |

MMAT= Mixed Methods Appraisal Tool, version 2018 (Hong et al., 2018)

- 1. **Non-randomised studies**

| **CITATION** | **SCREENING QUESTIONS** | | **NON-RANDOMISED STUDIES** | | | | |
| --- | --- | --- | --- | --- | --- | --- | --- |
|  | S1. Are there clear research questions? | S2. Do the collected data allow to address the research questions? | Are the participants representative of the target population ^a^? | Are measurements appropriate regarding both the outcome and intervention (or exposure)? | Are there complete outcome data? | Are the confounders accounted for in the design and analysis ^b^? | During the study period, is the intervention administered (or exposure occurred) as intended? |
| (Stip et al., 2012) | Yes | Yes | No | Yes | No | No | Can’t tell |
| (Briffa and Meehan, 1998) | Yes | Yes | No | Can't tell | No | No | Yes |
| (Friedrich et al., 2023) | Yes | Yes | Can’t tell | No | Yes | No | Yes |
| (Ntalkitsi et al., 2022) | Yes | Yes | Can’t tell | Yes | Yes | No | Can’t tell |
| (Costa E Silva et al., 2001) | Yes | Yes | Can’t tell | Can’t tell | Yes | No | Can’t tell |
| (Huang et al., 2020) | Yes | Yes | Can’t tell | Can't tell | No | No | Can't tell |
| (Mathews et al., 2012) | Yes | Yes | No | Yes | Can't tell | No | Yes |
| (Garriga et al., 2019) | Yes | Yes | No | Yes | Can't tell | No | Yes |
| (Gothelf et al., 2002) | Yes | Yes | No | Yes | Yes | No | Can't tell |
| (Saugo et al., 2020) | Yes | Yes | No | Yes | Yes | No | Yes |
| (Treuer et al., 2009)  (Case et al., 2010) study 4 (Treuer et al., 2009) | Yes | Yes | Can’t tell | Can't tell | No | No | Yes |
| (Abbas and Liddle, 2013) | Yes | Yes | No | Yes | Yes | No | Can't tell |
| (Archie et al., 2007) | Yes | Yes | No | Yes | Yes | No | Can't tell |
| (Blouin et al., 2008) | Yes | Yes | No | Yes | Yes | No | Can't tell |
| (de Beaurepaire, 2021) | Yes | Yes | Yes | Yes | Yes | No | Can't tell |
| (Henderson et al., 2006) | Yes | Yes | No | Yes | Yes | No | Can't tell |
| (Henderson et al., 2010) | Yes | Yes | Can’t tell | Yes | Yes | No | Can't tell |
| (Jakobsen et al., 2018b) | Yes | Yes | No | Yes | No | No | Can't tell |
| (Khazaal et al., 2006a; Khazaal et al., 2006b) | Yes | Yes | No | Yes | Yes | No | Can't tell |
| (Khazaal et al., 2009) | Yes | Yes | No | Yes | Yes | No | Can't tell |
| (Khosravi, 2020) | Yes | Yes | No | Yes | No | No | Can't tell |
| (Kouidrat et al., 2018) | Yes | Yes | No | Yes | Yes | No | Can't tell |
| (Nunes et al., 2014) | Yes | Yes | No | Yes | Yes | No | Can’t tell |
| (Sentissi et al., 2009) | Yes | Yes | Yes | Yes | Yes | No | Can't tell |
| (Stefanska et al., 2017) | Yes | Yes | No | Yes | Yes | No | Can’t tell |
| (Stefanska et al., 2018) | Yes | Yes | No | Yes | Yes | No | Can’t tell |

a= Indicators of representativeness include a clear description of the target population, the sample (inclusion and exclusion criteria), sampling technique used, differences between responders and non-responders.

b= Indicators of controlling confounding factors include the use of appropriate methods to control for confounders. Investigating effect modifiers is not an alternative to controlling for important confounders.

MMAT= Mixed Methods Appraisal Tool, version 2018 (Hong et al., 2018)

- 1. **Quantitative descriptive studies**

| **CITATION** | **SCREENING QUESTIONS** | | **QUANTITATIVE DESCRIPTIVE STUDIES** | | | | |
| --- | --- | --- | --- | --- | --- | --- | --- |
|  | S1. Are there clear research questions? | S2. Do the collected data allow to address the research questions? | Is the sampling strategy relevant to address the research question? | Is the sample representative of the target population? | Are the measurements appropriate? | Is the risk of nonresponse bias low? | Is the statistical analysis appropriate to answer the research question? |
| (Goluza et al., 2017) | Yes | Yes | No | Yes | Yes | Can’t tell | Yes |
| (Jakobsen et al., 2018a) | Yes | Yes | Yes | Yes | Yes | Can’t tell | Yes |
| (Kirkegaard et al., 1982) | Yes | Yes | Can't tell | Can’t tell | Can't tell | Can't tell | Yes |
| (Kurpad et al., 2010) | Yes | Yes | Can't tell | Can’t tell | Yes | Can't tell | Yes |
| (Lappin et al., 2018) | Yes | Yes | Yes | Yes | Can’t tell | No | Yes |
| (Llorca et al., 2017) | Yes | Yes | No | No | Can't tell | Can't tell | No |
| (Morell et al., 2019) | Yes | Yes | Yes | Yes | Can’t tell | No | Yes |
| (Qurashi et al., 2015) | Yes | Yes | Yes | Yes | Can’t tell | Can’t tell | Yes |
| (Srour et al., 2023) | Yes | Yes | Yes | No | No | No | Yes |
| (Murashita et al., 2005) | Yes | Yes | Can’t tell | No | No | Can't tell | No |
| (Horiguchi et al., 1999) | Yes | Yes | Yes | No | Yes | Yes | No |

MMAT= Mixed Methods Appraisal Tool, version 2018 (Hong et al., 2018)

**References**

Abbas, M. J. & Liddle, P. F. (2013). ‘Olanzapine and food craving: A case control study’ *Hum Psychopharmacol*, 28 (1), pp. 97-101. DOI: 10.1002/hup.2278 Available at: <https://www.ncbi.nlm.nih.gov/pubmed/23169487>.

Archie, S. M., et al. (2007). ‘Psychotic disorders, eating habits, and physical activity: Who is ready for lifestyle changes?’ *Psychiatric Services*, 58 (2), pp. 233-239. DOI: 10.1176/ps.2007.58.2.233.

Ballon, J. S., et al. (2018). ‘Pathophysiology of drug induced weight and metabolic effects: Findings from an rct in healthy volunteers treated with olanzapine, iloperidone, or placebo’ *J Psychopharmacol*, 32 (5), pp. 533-540. DOI: 10.1177/0269881118754708 Available at: <https://www.ncbi.nlm.nih.gov/pubmed/29444618>.

Bitter, I., et al. (2010). ‘Patients' preference for olanzapine orodispersible tablet compared with conventional oral tablet in a multinational, randomized, crossover study’ *World J Biol Psychiatry*, 11 (7), pp. 894-903. DOI: 10.3109/15622975.2010.505663 Available at: <https://www.ncbi.nlm.nih.gov/pubmed/20653494>.

Blouin, M., et al. (2008). ‘Adiposity and eating behaviors in patients under second generation antipsychotics’ *Obesity (Silver Spring)*, 16 (8), pp. 1780-7. DOI: 10.1038/oby.2008.277 Available at: <https://www.ncbi.nlm.nih.gov/pubmed/18535555>.

Briffa, D. & Meehan, T. (1998). ‘Weight changes during clozapine treatment’ *Aust N Z J Psychiatry*, 32 (5), pp. 718-21. DOI: 10.3109/00048679809113128 Available at: <https://www.ncbi.nlm.nih.gov/pubmed/9805596>.

Case, M., Treuer, T., Karagianis, J. & Hoffmann, V. P. (2010). ‘The potential role of appetite in predicting weight changes during treatment with olanzapine’ *BMC Psychiatry*, 10 p. 72. DOI: 10.1186/1471-244X-10-72 Available at: <https://www.ncbi.nlm.nih.gov/pubmed/20840778>.

Costa E Silva, J. A., et al. (2001). ‘Olanzapine as alternative therapy for patients with haloperidol-induced extrapyramidal symptoms: Results of a multicenter, collaborative trial in latin america’ *J Clin Psychopharmacol*, 21 (4), pp. 375-81. DOI: 10.1097/00004714-200108000-00004 Available at: <https://www.ncbi.nlm.nih.gov/pubmed/11476121>.

Daurignac, E., Leonard, K. E. & Dubovsky, S. L. (2015). ‘Increased lean body mass as an early indicator of olanzapine-induced weight gain in healthy men’ *Int Clin Psychopharmacol*, 30 (1), pp. 23-8. DOI: 10.1097/YIC.0000000000000052 Available at: <https://www.ncbi.nlm.nih.gov/pubmed/25350366>.

de Beaurepaire, R. (2021). ‘Binge eating disorders in antipsychotic-treated patients with schizophrenia: Prevalence, antipsychotic specificities, and changes over time’ *J Clin Psychopharmacol*, 41 (2), pp. 114-120. DOI: 10.1097/JCP.0000000000001357 Available at: <https://www.ncbi.nlm.nih.gov/pubmed/33587392>.

Fountaine, R. J., et al. (2010). ‘Increased food intake and energy expenditure following administration of olanzapine to healthy men’ *Obesity (Silver Spring)*, 18 (8), pp. 1646-51. DOI: 10.1038/oby.2010.6 Available at: <https://www.ncbi.nlm.nih.gov/pubmed/20134408> (Accessed: 2023/06/22).

Friedrich, M., Fugiel, J. & Sadowska, J. (2023). 'Assessing effects of diet alteration on carbohydrate–lipid metabolism of antipsychotic-treated schizophrenia patients in interventional study', *Nutrients*, 15(8) [Online]. DOI: 10.3390/nu15081871 Available at: <https://doi.org/10.3390/nu15081871>.

Garriga, M., et al. (2019). ‘Food craving and consumption evolution in patients starting treatment with clozapine’ *Psychopharmacology (Berl)*, 236 (11), pp. 3317-3327. DOI: 10.1007/s00213-019-05291-3 Available at: <https://www.ncbi.nlm.nih.gov/pubmed/31197435>.

Goluza, I., et al. (2017). ‘Exploration of food addiction in people living with schizophrenia’ *Asian J Psychiatr*, 27 pp. 81-84. DOI: 10.1016/j.ajp.2017.02.022 Available at: <https://www.ncbi.nlm.nih.gov/pubmed/28558903>.

Gothelf, D., et al. (2002). ‘Weight gain associated with increased food intake and low habitual activity levels in male adolescent schizophrenic inpatients treated with olanzapine’ *Am J Psychiatry*, 159 (6), pp. 1055-7. DOI: 10.1176/appi.ajp.159.6.1055 Available at: <https://www.ncbi.nlm.nih.gov/pubmed/12042200>.

Hardy, T., et al. 'Insulin sensitivity in patients with schizophrenia or schizoaffective disorder treated with olanzapine or risperidone', *162nd Annual Meeting Shaping our Future: Science and Service*, San Francisco: American Psychiatric Association, pp. 14-15.

Henderson, D. C., et al. (2006). ‘Dietary intake profile of patients with schizophrenia’ *Ann Clin Psychiatry*, 18 (2), pp. 99-105. DOI: 10.1080/10401230600614538 Available at: <https://www.ncbi.nlm.nih.gov/pubmed/16754415>.

Henderson, D. C., et al. (2010). ‘Dietary saturated fat intake and glucose metabolism impairments in nondiabetic, nonobese patients with schizophrenia on clozapine or risperidone’ *Ann Clin Psychiatry*, 22 (1), pp. 33-42. Available at: <https://www.ncbi.nlm.nih.gov/pubmed/20196981>.

Hoffman, V. P., Case, M. & Jacobson, J. G. 'Algorithms including amantadine, metformin and zonisamide for mitigation of weight gain during olanzapine treatment in outpatients with schizophrenia', *APA San Francisco*

Holt, R. I., et al. (2018). ‘Structured lifestyle education to support weight loss for people with schizophrenia, schizoaffective disorder and first episode psychosis: The stepwise rct’ *Health Technol Assess*, 22 (65), pp. 1-160. DOI: 10.3310/hta22650 Available at: <https://www.ncbi.nlm.nih.gov/pubmed/30499443>.

Hong, Q. N., et al. (2018). *Mixed methods appraisal tool (mmat), version 2018.* . Canada: IC Canadian Intellectual Property Office. Available at: <http://mixedmethodsappraisaltoolpublic.pbworks.com/w/file/fetch/127916259/MMAT_2018_criteria-manual_2018-08-01_ENG.pdf> (Accessed: 24 February 2023).

Horiguchi, J., et al. (1999). ‘Nocturnal eating/drinking syndrome and neuroleptic-induced restless legs syndrome’ *Int Clin Psychopharmacol*, 14 (1), pp. 33-6. Available at: <https://www.ncbi.nlm.nih.gov/pubmed/10221640>.

Huang, J., et al. (2020). ‘Corrigendum: Increased appetite plays a key role in olanzapine-induced weight gain in first-episode schizophrenia patients’ *Front Pharmacol*, 11 p. 878. DOI: 10.3389/fphar.2020.00878 Available at: <https://www.ncbi.nlm.nih.gov/pubmed/32587520>.

Jakobsen, A. S., et al. (2018a). ‘Associations between clinical and psychosocial factors and metabolic and cardiovascular risk factors in overweight patients with schizophrenia spectrum disorders - baseline and two-years findings from the change trial’ *Schizophr Res*, 199 pp. 96-102. DOI: 10.1016/j.schres.2018.02.047 Available at: <https://doi.org/10.1016/j.schres.2018.02.047>.

Jakobsen, A. S., et al. (2018b). ‘Dietary patterns and physical activity in people with schizophrenia and increased waist circumference’ *Schizophr Res*, 199 pp. 109-115. DOI: 10.1016/j.schres.2018.03.016 Available at: <https://www.ncbi.nlm.nih.gov/pubmed/29555213>.

Kane, J. M., et al. (2001). ‘Clozapine and haloperidol in moderately refractory schizophrenia: A 6-month randomized and double-blind comparison’ *Arch Gen Psychiatry*, 58 (10), pp. 965-72. DOI: 10.1001/archpsyc.58.10.965 Available at: <https://www.ncbi.nlm.nih.gov/pubmed/11576036> (Accessed: 6/23/2023).

Kang, D., et al. (2024). ‘The effect of continuous theta burst stimulation on antipsychotic-induced weight gain in first-episode drug-naive individuals with schizophrenia: A double-blind, randomized, sham-controlled feasibility trial’ *Transl Psychiatry*, 14 (1), p. 61. DOI: 10.1038/s41398-024-02770-w Available at: <https://www.ncbi.nlm.nih.gov/pubmed/38272892>.

Karagianis, J., et al. (2009). ‘A randomized controlled trial of the effect of sublingual orally disintegrating olanzapine versus oral olanzapine on body mass index: The platypus study’ *Schizophr Res*, 113 (1), pp. 41-8. DOI: 10.1016/j.schres.2009.05.024 Available at: <https://www.ncbi.nlm.nih.gov/pubmed/19535229>.

Khazaal, Y., et al. (2009). ‘Hunger and negative alliesthesia to aspartame and sucrose in patients treated with antipsychotic drugs and controls’ *Eat Weight Disord*, 14 (4), pp. e225-30. DOI: 10.1007/BF03325121 Available at: <https://www.ncbi.nlm.nih.gov/pubmed/20179410>.

Khazaal, Y., Fresard, E., Borgeat, F. & Zullino, D. (2006a). ‘Binge eating symptomatology in overweight and obese patients with schizophrenia: A case control study’ *Ann Gen Psychiatry*, 5 p. 15. DOI: 10.1186/1744-859X-5-15 Available at: <https://www.ncbi.nlm.nih.gov/pubmed/16968528>.

Khazaal, Y., et al. (2007). ‘Cognitive behavioural therapy for weight gain associated with antipsychotic drugs’ *Schizophr Res*, 91 (1-3), pp. 169-77. DOI: 10.1016/j.schres.2006.12.025 Available at: <https://www.ncbi.nlm.nih.gov/pubmed/17306507>.

Khazaal, Y., et al. (2006b). ‘Eating and weight related cognitions in people with schizophrenia : A case control study’ *Clin Pract Epidemiol Ment Health*, 2 p. 29. DOI: 10.1186/1745-0179-2-29 Available at: <https://www.ncbi.nlm.nih.gov/pubmed/17076886>.

Khosravi, M. (2020). ‘Biopsychosocial factors associated with disordered eating behaviors in schizophrenia’ *Ann Gen Psychiatry*, 19 (1), p. 67. DOI: 10.1186/s12991-020-00314-2 Available at: <https://www.ncbi.nlm.nih.gov/pubmed/33292324>.

Kirkegaard, A., Hammershoj, E. & Ostergard, P. (1982). ‘Evaluation of side effects due to clozapine in long-term treatment of psychosis’ *Arzneimittel-Forschung*, 32 (4), pp. 465-8. Available at: <http://ovidsp.ovid.com/ovidweb.cgi?T=JS&PAGE=reference&D=med2&NEWS=N&AN=7201818>.

Kluge, M., et al. (2007). ‘Clozapine and olanzapine are associated with food craving and binge eating: Results from a randomized double-blind study’ *J Clin Psychopharmacol*, 27 (6), pp. 662-6. DOI: 10.1097/jcp.0b013e31815a8872 Available at: <https://www.ncbi.nlm.nih.gov/pubmed/18004133>.

Kouidrat, Y., et al. (2018). ‘Disordered eating behaviors as a potential obesogenic factor in schizophrenia’ *Psychiatry Res*, 269 pp. 450-454. DOI: 10.1016/j.psychres.2018.08.083 Available at: <https://www.ncbi.nlm.nih.gov/pubmed/30195737>.

Kurpad, S. S., George, S. A. & Srinivasan, K. (2010). ‘Binge eating and other eating behaviors among patients on treatment for psychoses in india’ *Eat Weight Disord*, 15 (3), pp. e136-43. DOI: 10.1007/BF03325293 Available at: <https://www.ncbi.nlm.nih.gov/pubmed/21150249>.

Lappin, J. M., et al. (2018). ‘Cardio-metabolic risk and its management in a cohort of clozapine-treated outpatients’ *Schizophr Res*, 199 pp. 367-373. DOI: 10.1016/j.schres.2018.02.035 Available at: <https://www.ncbi.nlm.nih.gov/pubmed/29486959>.

Llorca, P. M., et al. (2017). ‘Assessing the burden of treatment-emergent adverse events associated with atypical antipsychotic medications’ *BMC Psychiatry*, 17 (1), p. 67. DOI: 10.1186/s12888-017-1213-6 Available at: <https://www.ncbi.nlm.nih.gov/pubmed/28193195>.

Mathews, J., et al. (2012). ‘Neural correlates of weight gain with olanzapine’ *Arch Gen Psychiatry*, 69 (12), pp. 1226-37. DOI: 10.1001/archgenpsychiatry.2012.934 Available at: <https://www.ncbi.nlm.nih.gov/pubmed/22868896>.

Morell, R., et al. (2019). ‘Cardio-metabolic risk in individuals prescribed long-acting injectable antipsychotic medication’ *Psychiatry Res*, 281 p. 112606. DOI: 10.1016/j.psychres.2019.112606 Available at: <https://www.ncbi.nlm.nih.gov/pubmed/31629301>.

Murashita, M., et al. (2005). ‘Olanzapine increases plasma ghrelin level in patients with schizophrenia’ *Psychoneuroendocrinology*, 30 (1), pp. 106-10. DOI: 10.1016/j.psyneuen.2004.05.008 Available at: <https://www.ncbi.nlm.nih.gov/pubmed/15358448>.

Ntalkitsi, S., Efthymiou, D., Bozikas, V. & Vassilopoulou, E. (2022). 'Halting the metabolic complications of antipsychotic medication in patients with a first episode of psychosis: How far can we go with the mediterranean diet? A pilot study', *Nutrients*, 14(23) [Online]. DOI: 10.3390/nu14235012 Available at: <https://doi.org/10.3390/nu14235012>.

Nunes, D., et al. (2014). ‘Nutritional status, food intake and cardiovascular disease risk in individuals with schizophrenia in southern brazil: A case-control study’ *Rev Psiquiatr Salud Ment*, 7 (2), pp. 72-9. DOI: 10.1016/j.rpsm.2013.07.001 Available at: <https://www.ncbi.nlm.nih.gov/pubmed/24054065>.

Park, S., Yi, K. K., Kim, M. S. & Hong, J. P. (2013). ‘Effects of ziprasidone and olanzapine on body composition and metabolic parameters: An open-label comparative pilot study’ *Behav Brain Funct*, 9 p. 27. DOI: 10.1186/1744-9081-9-27 Available at: <https://www.ncbi.nlm.nih.gov/pubmed/23866300>.

Qurashi, I., et al. (2015). ‘An evaluation of subjective experiences, effects and overall satisfaction with clozapine treatment in a uk forensic service’ *Ther Adv Psychopharmacol*, 5 (3), pp. 146-50. DOI: 10.1177/2045125315581996 Available at: <https://www.ncbi.nlm.nih.gov/pubmed/26199716> (Accessed: 2023/06/30).

Roerig, J. L., et al. (2005). ‘A comparison of the effects of olanzapine and risperidone versus placebo on eating behaviors’ *J Clin Psychopharmacol*, 25 (5), pp. 413-8. DOI: 10.1097/01.jcp.0000177549.36585.29 Available at: <https://www.ncbi.nlm.nih.gov/pubmed/16160615>.

Saugo, E., et al. (2020). ‘Dietary habits and physical activity in first-episode psychosis patients treated in community services. Effect on early anthropometric and cardio-metabolic alterations’ *Schizophr Res*, 216 pp. 374-381. DOI: 10.1016/j.schres.2019.11.010 Available at: <https://www.ncbi.nlm.nih.gov/pubmed/31806524>.

Sentissi, O., et al. (2009). ‘Impact of antipsychotic treatments on the motivation to eat: Preliminary results in 153 schizophrenic patients’ *Int Clin Psychopharmacol*, 24 (5), pp. 257-64. DOI: 10.1097/YIC.0b013e32832b6bf6 Available at: <https://www.ncbi.nlm.nih.gov/pubmed/19606055>.

Smith, R. C., Rachakonda, S., Dwivedi, S. & Davis, J. M. (2012). ‘Olanzapine and risperidone effects on appetite and ghrelin in chronic schizophrenic patients’ *Psychiatry Res*, 199 (3), pp. 159-63. DOI: 10.1016/j.psychres.2012.03.011 Available at: <https://www.ncbi.nlm.nih.gov/pubmed/22475524>.

Srour, A., et al. (2023). ‘Patients' and primary carers' views on clozapine treatment for schizophrenia: A cross-sectional study in qatar’ *Saudi Pharm J*, 31 (2), pp. 214-221. DOI: 10.1016/j.jsps.2022.12.005 Available at: <https://www.ncbi.nlm.nih.gov/pubmed/36942276>.

Stefanska, E., et al. (2017). ‘Eating habits and nutritional status of patients with affective disorders and schizophrenia’ *Psychiatr Pol*, 51 (6), pp. 1107-1120. DOI: 10.12740/PP/74558 Available at: <https://www.ncbi.nlm.nih.gov/pubmed/29432506>.

Stefanska, E., et al. (2018). ‘The assessment of the nutritional value of meals consumed by patients with recognized schizophrenia’ *Rocz Panstw Zakl Hig*, 69 (2), pp. 183-192. Available at: <https://www.ncbi.nlm.nih.gov/pubmed/29766697>.

Stip, E., et al. (2012). ‘Neural changes associated with appetite information processing in schizophrenic patients after 16 weeks of olanzapine treatment’ *Transl Psychiatry*, 2 (6), p. e128. DOI: 10.1038/tp.2012.53 Available at: <https://www.ncbi.nlm.nih.gov/pubmed/22714121>.

Teff, K. L., Rickels, K., Alshehabi, E. & Rickels, M. R. (2015). ‘Metabolic impairments precede changes in hunger and food intake following short-term administration of second-generation antipsychotics’ *J Clin Psychopharmacol*, 35 (5), pp. 579-82. DOI: 10.1097/JCP.0000000000000393 Available at: <https://www.ncbi.nlm.nih.gov/pubmed/26274045>.

Teff, K. L., et al. (2013). ‘Antipsychotic-induced insulin resistance and postprandial hormonal dysregulation independent of weight gain or psychiatric disease’ *Diabetes*, 62 (9), pp. 3232-40. DOI: 10.2337/db13-0430 Available at: <https://www.ncbi.nlm.nih.gov/pubmed/23835329> (Accessed: 5/1/2023).

Tollefson, G. D., et al. (1997). ‘Olanzapine versus haloperidol in the treatment of schizophrenia and schizoaffective and schizophreniform disorders: Results of an international collaborative trial’ *Am J Psychiatry*, 154 (4), pp. 457-65. DOI: 10.1176/ajp.154.4.457 Available at: <https://www.ncbi.nlm.nih.gov/pubmed/9090331> (Accessed: 2023/06/30).

Treuer, T., et al. (2009). ‘Factors associated with weight gain during olanzapine treatment in patients with schizophrenia or bipolar disorder: Results from a six-month prospective, multinational, observational study’ *World J Biol Psychiatry*, 10 (4 Pt 3), pp. 729-40. DOI: 10.1080/15622970903079507 Available at: <https://www.ncbi.nlm.nih.gov/pubmed/19606406>.

Yang, Y., et al. (2021). ‘Effect of bifidobacterium on olanzapine-induced body weight and appetite changes in patients with psychosis’ *Psychopharmacology (Berl)*, 238 (9), pp. 2449-2457. DOI: 10.1007/s00213-021-05866-z Available at: <https://www.ncbi.nlm.nih.gov/pubmed/34002246>.
