# Supplementary material for: The impacts of antipsychotic medications on eating-related outcomes: A mixed methods systematic review
Source: PLoS One. 2025 Feb 3;20(2):e0308037. doi: 10.1371/journal.pone.0308037 (PMC11790239; doi:10.1371/journal.pone.0308037)
Supplement: S9 File — (DOCX) [file pone.0308037.s009.docx]

# S9 File. Detailed account of the quantitative syntheses.

## Within-group syntheses - Summary of effect measures of antipsychotic medications on appetite sensations

| BMI | Citation | Study design | End of study assessment | Sample size | Antipsychotic | Duration of prior AP treatment/ other medications | Baseline BMI,  M (SD) | Outcome of interest | Measurement scale | Analysis | Posttreatment mean | Posttreatment SD | Baseline mean | Baseline SD | **Mean difference** | p-value | 95% CI |
| --- | --- | --- | --- | --- | --- | --- | --- | --- | --- | --- | --- | --- | --- | --- | --- | --- | --- |
|  | **APPETITE** | | | | | | | | | | | | | | | | |
| **HIGH** | Case et al., 2010 Study 1 (Hardy et al., 2009) | 2-arm parallel, double-blind RCT | 2 weeks | 68 | Olanzapine | Participants excluded if they had olanzapine, risperidone, or depot APs within 4 weeks of study entry, or clozapine within 2 years of entry.  Use of other APs, mood stabilizers (not SSRIs) was not allowed during the study.  Patients discontinued previous AP 3-10 days before enrolment.  Participants received 4 dietary consultations and were encouraged to have regular physical activity. | 28.96 (5.17) | Appetite (natural setting) | EBA ^a, b^ | Difference in mean EBA scores from baseline to week 2 | 11.10* | NA | 9.90* | NA | **1.20*** | NA | NR |
|  | Case et al., 2010 Study 2 (Karagianis et al., 2009) | 2-arm parallel, double-blind RCT | 2 weeks | 65 | Olanzapine | At enrolment patients had been taking 5-20 mg standard olanzapine tablets/day between ≥4 and ≤52 weeks and experienced weight gain ≥5 kg or a change of ≥1 kg/m2 BMI.  Duration of prior olanzapine treatment in weeks, mean (SD): 19.9 (13.8).  BMI increase on prior olanzapine treatment, mean (SD): 2.6 (1.21). Taking other medications that could affect weight or participating in a weight loss program were exclusion criteria. | 28.30 (4.80) | Appetite (natural setting) | PARS ^a, b^ | Difference in mean PARS scores from baseline to week 2 | 58.90 | NA | 65.70 | 19.20 | **-6.80*** | 0.04 | NR |
|  | (Smith et al., 2012) | 2-arm, parallel-group, open-label RCT | 5 months | 23 | Olanzapine | All patients had been treated with multiple APs in the past (SGA n=14, combination of FGA and SGA n= 9).  APs at baseline:  Olanzapine n= 13; risperidone n=6; neither olanzapine nor risperidone n=4).  Duration of prior AP treatment: not reported.  Other medications received:  antidepressants 3, lithium 4, valproate 11. | 29.96 (6.50) | Appetite (natural setting) | VAS^a,b^, EBA ^a, b^ | Difference in mean scores from baseline to month 5 | NR | NR | NR | NR | **Negative** | NR: results illustrated as a line graph. | NR |
|  | (Smith et al., 2012) | 2-arm, parallel-group, open-label RCT | 5 months | 23 | Risperidone | All patients had been treated with multiple antipsychotics in the past (SGA n=12, FGA n= 4, combination of FGA and SGA n=7).  APs at baseline:  Olanzapine n= 8; risperidone n=11; neither olanzapine nor risperidone n=4).  Duration of prior AP treatment: not reported.  Other medications:  antidepressants n= 4; lithium n= 4; valproate n= 9. | 28.85 (5.71) | Appetite (natural setting) | VAS^a,b^, EBA ^a, b^ | Difference in mean scores from baseline to month 5 | NR | NR | NR | NR | **Negative** | NR: results illustrated as a line graph. | NR |
| **NORMAL** | (Park et al., 2013) | 2-arm parallel open-label RCT | 12 weeks | 10 | Ziprasidone | Participants were excluded if they had received APs during past 3 months or had received medications (other than lorazepam, clonazepam, zolpidem, benztropine, or propranolol) for any medical or other psychiatric condition. | Median (IQR): 21.50 (19.20, 24.00) | Appetite (natural setting) | VAS ^a, b^ | Difference in median appetite scores from baseline to week 12 | 5 (median) | NA | 5 (median) | NA | **0** | 0.212 | NR |
|  | (Park et al., 2013) | 2-arm parallel open-label RCT | 12 weeks | 10 | Olanzapine | Participants were excluded if they had received APs during the past 3 months or had received medications (other than lorazepam, clonazepam, zolpidem, benztropine, or propranolol) for any medical or other psychiatric condition. | Median (IQR): 21.85 (17.90, 23.65) | Appetite (natural setting) | VAS ^a, b^ | Difference in median appetite scores from baseline to week 12 | 5 (median) | NR | 5 (median) | NR | **0** | 0.399 | NR |
|  | Case et al., 2010  Study 4 (Treuer et al., 2009) | Secondary analysis of a prospective 6-month study | 4 weeks | 606 | Olanzapine | Of 622 participants initially enrolled in the original study, 463 (74.5%) patients had received prior AP therapy before study entry.  Other medications: 123 (19.9%) had taken mood stabilizers, and 337 (54.2%) had taken other psychiatric medications (i.e., antidepressants, anticholinergics, sedatives, anxiolytics, hypnotics, and/or benzodiazepines).  Participants who had been participating in a weight control program (WCP) at study entry, n (%): 147/622 (23.6). Of the remaining 475 patients, 99 (20.8%) started a WCP after study entry visit and during olanzapine therapy.  During the study, 68 (11%) of the patients at each visit had taken other AP, in addition to olanzapine since the previous visit (SGA, n=35 (5.7%) and FGA, n=33 (5.3%); and 105 (17.0%) of the patients had taken mood stabilizers (valproate, carbamazepine). | 23.2 (3.9) | Appetite (natural setting) | EAS ^a, b^ | Difference in mean EAS scores from baseline to week 4 | 8.1* | NA | 9.3* | NA | **-0.17*** | NA | NR |
| **CRAVING** | | | | | | | | | | | | | | | | |  |
| **General food cravings** | | | | | | | | | | | | | | | | |  |
| **HIGH** | Case et al., 2010 Study 3 (Hoffman et al., 2009) | 3-arm parallel, open label RCT | 2 weeks | 50 | Olanzapine | Brief weight management education was provided at baseline. | 27.0 (4.5) | Craving (natural setting)  General food craving | FCI | Difference in mean total food craving scores from baseline to week 2 | 61.5 | NR | 65.40 | 20.10 | **-3.9*** | <0.0001 | NR |
|  | (Garriga et al., 2019) | Cohort | 18 weeks | OWO group: 21 | Clozapine | Prior AP treatment: FGA, n=0; SGA, n= 21 (100%).  Duration of prior AP treatment, years mean (SD): 10.90 (11.00) | 30.3 0 (3.70) | Craving (natural setting)  General food craving | FCI-SP | Difference in mean total food craving scores from baseline to week 18 in OWO group | NR | NR | 2.51 | NR | **-0.16*** | 0.311 | (-0.47, 0.15) |
| **NORMAL** | (Garriga et al., 2019) | Cohort | 18 weeks | NW group: 13 | Clozapine | Prior AP treatment: FGA, n=3 (23.10%); SGA, n= 7 (53.80%), none, n=3 (23.10%).  Duration of prior AP treatment, years mean (SD): 5.20 (7.60) | 22.00 (2.70) | Craving (natural setting)  General food craving | FCI-SP | Difference in mean total food craving scores from baseline to week 18 in NW group | NR | NR | 2.19 | NR | **0.44*** | 0.185 | (-0.21, 1.09) |
| **Carbohydrates** | | | | | | | | | | | | | | | | |  |
| **Complex carbohydrates/proteins** | | | | | | | | | | | | | | | | |  |
| **HIGH** | (Garriga et al., 2019) | Cohort | 18 weeks | OWO group: 21 | Clozapine | Prior AP treatment: FGA, n=0; SGA, n= 21 (100%).  Duration of prior AP treatment, years mean (SD): 10.90 (11.00) | 30.3 0 (3.70) | Craving (natural setting)  Complex carbohydrates/proteins | FCI-SP | Difference in mean complex carbohydrates/protein craving scores from baseline to week 18 in OWO group | NR | NR | 2.62 | NR | **0.08*** | 0.64 | (-0.25, 0.42) |
| **NORMAL** | (Garriga et al., 2019) | Cohort | 18 weeks | NW group: 13 | Clozapine | Prior AP treatment: FGA, n=3 (23.10%); SGA, n= 7 (53.80%), none, n=3 (23.10%).  Duration of prior AP treatment, years mean (SD): 5.20 (7.60) | 22.00 (2.70) | Craving (natural setting)  Complex carbohydrates/proteins | FCI-SP | Changes in mean complex carbohydrates/protein craving scores from baseline to week 18 in NW group | NR | NR | 2.45 | NR | **0.67*** | 0.01 | (0.19, 1.15) |
| **Simple sugar/trans fat** | | | | | | | | | | | | | | | | |  |
| **HIGH** | (Garriga et al., 2019) | Cohort | 18 weeks | OWO group: 21 | Clozapine | Prior AP treatment: FGA, n=0; SGA, n= 21 (100%).  Duration of prior AP treatment, years mean (SD): 10.90 (11.00). | 30.3 0 (3.70) | Craving (natural setting)  Simple sugar/trans fat | FCI-SP | Difference in mean simple sugar/transfat craving scores from baseline to week 18 in OWO group | NR | NR | 2.29 | NR | **-0.13*** | 0.458 | (-0.48, 0.22) |
| **NORMAL** | (Garriga et al., 2019) | Cohort | 18 weeks | NW group: 13 | Clozapine | Prior AP treatment: FGA, n=3 (23.10%); SGA, n= 7 (53.80%), none, n=3 (23.10%).  Duration of prior AP treatment, years mean (SD): 5.20 (7.60) | 22.00 (2.70) | Craving (natural setting)  Simple sugar/trans fat | FCI-SP | Difference in mean simple sugar/transfat craving scores from baseline to week 18 in NW group | NR | NR | 2.21 | NR | **0.44*** | 0.113 | (-0.11, 0.98) |
| **Fast-food fats (FFF)** | | | | | | | | | | | | | | | | |  |
| **HIGH** | (Garriga et al., 2019) | Cohort | 18 weeks | OWO group: 21 | Clozapine | Prior AP treatment: FGA, n=0; SGA, n= 21 (100%).  Duration of prior AP treatment, years mean (SD): 10.90 (11.00) | 30.3 0 (3.70) | Craving (natural setting)  FFF | FCI-SP | Difference in mean fast-food fats craving scores from baseline to week 18 in OWO group | NR | NR | 2.81 | NR | **-0.42*** | 0.054 | (-0.85, 0.01) |
| **NORMAL** | (Garriga et al., 2019) | Cohort | 18 weeks | NW group: 13 | Clozapine | Prior AP treatment: FGA, n=3 (23.10%); SGA, n= 7 (53.80%), none, n=3 (23.10%).  Duration of prior AP treatment, years mean (SD): 5.20 (7.60) | 22.00 (2.70) | Craving (natural setting)  Fast-food fats | FCI-SP | Difference in mean fast-food fats craving scores from baseline to week 18 in NW group | NR | NR | 2.05 | NR | **0.31*** | 0.435 | (-0.47, 1.09) |
| **HUNGER** | | | | | | | | | | | | | | | | | |
| ***HEALTHY VOLUNTEERS*** | | | | | | | | | | | | | | | | | |
| **NORMAL** | (Ballon et al., 2018) | 3-arm double-blind, parallel RCT | 28 days | 7 | Olanzapine | Healthy volunteers | 22.70 (0.30) | Hunger (lab setting)  Self-reported pre-meal hunger (measured prior to the standardised lab lunch meal) | VAS | Difference in mean pre-meal hunger between baseline and day 28 | 89.00 | 11.00 | 82.00 | 17.00 | **7.00*** | 0.045 | NR |
|  | (Ballon et al., 2018) | 3-arm double-blind, parallel RCT | 28 days | 7 | Iloperidone | Healthy volunteers | 23.60 (0.60) | Hunger (lab setting)  Self-reported pre-meal hunger (measured prior to the standardised lab lunch meal) | VAS | Difference in mean pre-meal hunger between baseline and day 28 | 86.00 | 19.00 | 84.00 | 18.00 | **2.00*** | 0.64 | NR |
|  | (Roerig et al., 2005) | 3-arm parallel, double-blind RCT | 2 weeks | 16 | Olanzapine | Healthy volunteers | 23.625 (NR) | Hunger (lab setting)  Self-reported post-meal hunger (measured over 4 hours after a standardised breakfast meal) | VAS | Difference in breakfast hunger curves between baseline and at the end of week 2 | NR | NR | NR | NR | **Positive** (illustrated in line graph) | 0.086 | NR |
|  | (Teff et al., 2013) | 3-arm parallel, double-blind RCT | 12 days | 10 | Olanzapine | Healthy volunteers | 22.10 (1.40) | Hunger (lab setting)  Cumulative daily score of hunger over the course of the study. | VAS | Difference in cumulative daily score of hunger between baseline and day 12. | NR | NR | NR | NR | **Positive** (illustrated in line graph) | NR | NR |
|  | (Teff et al., 2013) | 3-arm parallel, double-blind RCT | 12 days | 10 | Aripiprazole | Healthy volunteers | 22.40 (1.30) | Hunger (lab setting)  Cumulative daily score of hunger over the course of the study. | VAS | Difference in cumulative daily score of hunger between baseline and day 12. | NR | NR | NR | NR | **Positive** (illustrated in line graph) | NR | NR |

Mean difference (or difference in means) in within group analyses= posttreatment mean – baseline mean

a= unvalidated measurement scale; b= collective scale (i.e., measure appetite and food craving or eating cognitions; *= calculated/converted from data reported in record

AP(s)= antipsychotic(s); CI= confidence interval; EAS= Eating Attitude Scale; EBA= Eating Behavior Assessment; FCI= Food Craving Inventory; FCI-SP= the Spanish version of the Food Craving Inventory; FGA= first-generation antipsychotic; IQR= interquartile range; M= mean; n= number; NR= not reported; PARS= Platypus Appetite Rating Scale; RCT= randomised-controlled trail; SD= standard deviation; SGA= second-generation antipsychotic; VAS= visual analogue scale.

## Within-group syntheses - Summary of effect measures of antipsychotic medications on food intake and dietary composition

| BMI | Citation | Study design | End of study assessment | Sample size | Antipsychotic | Duration of prior AP treatment/other medications | Baseline BMI, M (SD) | Outcome of interest | Measurement scale | Analysis | Posttreatment mean | Posttreatment SD | Baseline mean | Baseline SD | **Mean difference** | p-value | 95% CI |
| --- | --- | --- | --- | --- | --- | --- | --- | --- | --- | --- | --- | --- | --- | --- | --- | --- | --- |
|  | **ENERGY INTAKE** | | | | | | | | | | | | | | | | |
| **NORMAL** | (Gothelf et al., 2002) | 4-week prospective study | 4 weeks | 10 | Olanzapine | Participants were excluded if they received other medications that affect weight (such as lithium, antidepressants, or valproate).  Prior AP treatment: drug naïve n=1, clomipramine n=1, AP other than olanzapine n=8. | 24.50 (5.90) | Total caloric intake (kcal/day)  Inpatient | Dietician closely monitored food intake for 2 consecutive days. All food products and beverages consumed, as well as snacks, were weighed before and after the meal. | Difference in mean total caloric intake between baseline and week 4 | 2716 | 958 | 2127 | 1032 | **589.00*** | 0.03 | NR |
| ***HEALTHY VOLUNTEERS*** | | | | | | | | | | | | | | | | | |
| **NORMAL** | (Ballon et al., 2018) | 3-arm double-blind, parallel RCT | 28 days | 7 | Olanzapine | Healthy volunteers | 22.70 (0.30) | Caloric intake (kcal) of food consumed.  Lab setting | Energy intake (kcal) was calculated for all food consumed from a standarised lab lunch meal | Difference in mean total caloric intake between baseline and day 28 | NR | NR | NR | NR | **268.00 (SD= 77)** | 0.01 | NR |
|  | (Ballon et al., 2018) | 3-arm double-blind, parallel RCT | 28 days | 7 | Iloperidone | Healthy volunteers | 23.60 (0.60) | Caloric intake (kcal) of food consumed.  Lab setting | Energy intake (kcal) was calculated for all food consumed from a standarised lab lunch meal | Difference in mean total caloric intake between baseline and day 28 | NR | NR | NR | NR | **Negative**  (presented in graph) | NR | NR |
|  | (Roerig et al., 2005) | 3-arm parallel, double-blind RCT | 2 weeks | 16 | Olanzapine | Healthy volunteers | 23.625 (NR) | Energy intake (kcal) consumed at dinner session.  Lab setting | Energy intake (kcal) was calculated for all food consumed after the dinner session (lab). | Difference in mean total caloric intake between baseline and week 2 | NR | NR | NR | NR | **151.856 (SE= 67.10)** | NR | NR |
|  | (Roerig et al., 2005) | 3-arm parallel, double-blind RCT | 2 weeks | 16 | Risperidone | Healthy volunteers | 24.969 (NR) | Energy intake (kcal) consumed at dinner session.  Lab setting. | Energy intake (kcal) was calculated for all food consumed after the dinner session (lab). | Difference in mean total caloric intake between baseline and week 2 | NR | NR | NR | NR | **-31.037 (SE= 61.01)** | NR | NR |
|  | (Teff et al., 2015) | 3-arm parallel, double-blind RCT | 12 days | 10 | Olanzapine | Healthy volunteers | 22.10 (1.40) | Energy intake (kcal)  Lab setting | Energy intake (kcal) was calculated for all food consumed on days 2 (pre-intervention) and 11 (post-intervention) | Difference in mean total caloric intake between days 2 (pre- intervention) and 11 (post-intervention) | NR | NR | NR | NR | **363.00 (SD= 380.60)** | 0.70 | NR |
|  | (Teff et al., 2015) | 3-arm parallel, double-blind RCT | 12 days | 10 | Aripiprazole | Healthy volunteers | 22.40 (1.30) | Energy intake (kcal)  Lab setting | Energy intake (kcal) was calculated for all food consumed on days 2 (pre-intervention) and 11 (post-intervention) | Difference in mean total caloric intake between days 2 (pre- intervention) and 11 (post-intervention) | NR | NR | NR | NR | **−269.9 (SD= 552.60)** | 0.53 | NR |
| **FAT** | | | | | | | | | | | | | | | | | |
| **NORMAL** | (Gothelf et al., 2002) | 4-week prospective study | 4 weeks | 10 | Olanzapine | Participants were excluded if they received other medications that affect weight (such as lithium, antidepressants, or valproate).  Prior AP treatment: drug naïve n=1, clomipramine n=1, AP medication other than olanzapine n=8. | 24.50 (5.90) | Diet composition for fats (%)  Inpatient setting | A clinical dietician closely monitored food intake for 2 consecutive days. All food products and beverages consumed were weighed before and after meals. | Difference in diet composition for fats between baseline and week 4 | 27.00% | 5.50% | 29.90% | 8.70% | **-2.90** | NR | NR |
| ***HEALTHY VOLUNTEERS*** | | | | | | | | | | | | | | | | | |
| **NORMAL** | (Ballon et al., 2018) | 3-arm double-blind, parallel RCT | 28 days | 7 | Olanzapine | Healthy volunteers | 22.70 (0.30) | Fat intake (g)  Lab setting | Gram weight were calculated for all food consumed from a standardised lab lunch meal. | Difference in mean fat intake between baseline and day 28 | NR | NR | NR | NR | **13.00 (SD= 7.00)** | 0.09 | NR |
| **CARBOHYDRATES** | | | | | | | | | | | | | | | | | |
| **NORMAL** | (Gothelf et al., 2002) | 4-week prospective study | 4 weeks | 10 | Olanzapine | Participants were excluded if they received other medications that affect weight (such as lithium, antidepressants, or valproate).  Prior AP treatment: drug naïve n=1, clomipramine n=1, AP other than olanzapine n=8. | 24.50 (5.90) | Diet composition for carbohydrates (%)  Inpatient setting | A clinical dietician closely monitored food intake for 2 consecutive days. All food products and beverages consumed, as well as snacks, were weighed before and after the meal. | Difference in diet composition for carbohydrates between baseline and week 4 | 56.20% | 5.40% | 53.40% | 5.90% | **2.80*** | NR | NR |
| ***HEALTHY VOLUNTEERS*** | | | | | | | | | | | | | | | | | |
| **NORMAL** | (Ballon et al., 2018) | 3-arm double-blind, parallel RCT | 28 days | 7 | Olanzapine | Healthy volunteers | 22.70 (0.30) | Carbohydrate intake (g)  Lab setting | Gram weight were calculated for all food consumed from a standardised lab lunch meal. | Difference in mean carbohydrate intake between baseline and day 28 | NR | NR | NR | NR | **22.00**  **(SD= 11.00)** | 0.09 | NR |
| **PROTEIN** | | | | | | | | | | | | | | | | | |
| **NORMAL** | (Gothelf et al., 2002) | 4-week prospective study | 4 weeks | 10 | Olanzapine | Participants were excluded if they received other medications that affect weight (such as lithium, antidepressants, or valproate).  Prior AP treatment: drug naïve n=1, clomipramine n=1, AP medication other than olanzapine n=8. | 24.50 (5.90) | Diet composition for proteins (%)  Inpatient setting | A clinical dietician closely monitored food intake for 2 consecutive days. All food products and beverages consumed, as well as snacks, were weighed before and after the meal. | Difference in diet composition for proteins between baseline and week 4 | 16.50% | 3.10% | 16.70% | 4.30% | **-0.20*** | NR | NR |
| ***HEALTHY VOLUNTEERS*** | | | | | | | | | | | | | | | | | |
| **HIGH** | (Daurignac et al., 2015) | 2-arm double-blind parallel RCT | 2 weeks | 13 | Olanzapine | Healthy volunteers | 25.3 (3.0) | Protein intake (g)  Lab setting | Gram weight were calculated for all food consumed from a standardised lab breakfast meal | Difference in mean protein intake between baseline and day 12 | NR | NR | NR | NR | **8.40 (SD= 7.5)** | 0.04 | NR |
| **NORMAL** | (Ballon et al., 2018) | 3-arm double-blind, parallel RCT | 28 days | 7 | Olanzapine | Healthy volunteers | 22.70 (0.30) | Protein intake (g)  Lab setting | Gram weight were calculated for all food consumed from a standardised lab lunch meal | Difference in mean protein intake between baseline and day 28 | NR | NR | NR | NR | **14.00 (SD= 11)** | 0.23 | NR |

Mean difference (or difference in means) in within group analyses= posttreatment mean – baseline mean

%= percentage; AP= antipsychotic medication; CI= confidence interval; g= gram; kcal= kilocalories; M= mean; n= number; NR= not reported; RCT= randomised-controlled trail; SD= standard deviation; SE= standard error.

## Within-group syntheses - Summary of effect measures of antipsychotics on eating cognitions and behaviours

| BMI | Citation | Study design | End of study assessment | Sample size | Antipsychotic | Duration of AP treatment/ other medications | Baseline BMI, M (SD) | Outcome of interest | Measurement scale | Analysis | Posttreatment mean | Posttreatment SD | Baseline mean | Baseline SD | Mean difference | p-value | 95% CI |
| --- | --- | --- | --- | --- | --- | --- | --- | --- | --- | --- | --- | --- | --- | --- | --- | --- | --- |
| **OVERALL SCORE** | | | | | | | | | | | | | | | | |  |
| **NORMAL** | (Kang et al., 2024) | 2-arm parallel, double-blind RCT | 5 days | 19 | Olanzapine | No previous AP treatment | 21.70 (3.71) | Eating behaviour | TFEQ-R21 total score | Difference in mean TEFQ-R21 scores before and after the 5-day olanzapine treatment. | 54.32 | 6.42 | 56.13 | 6.01 | **-1.81** | 0.143 | NR |
| ***HEALTHY VOLUNTEERS*** | | | | | | | | | | | | | | | | | |
| **HIGH** | (Mathews et al., 2012) | Pre-post study | 7 days | 19 | Olanzapine | Healthy volunteers | 25.78 (4.82) | Eating behaviour | TFEQ total score | Difference in mean total TFEQ scores before and after the 7-day olanzapine treatment | 37.89 | 6.85 | 35.55 | 7.07 | **2.34*** | 0.009 | NR |
| **DIETARY RESTRIANT** | | | | | | | | | | | | | | | | |  |
| **HIGH** | Case et al., 2010  Study 3 (Hoffman et al., 2009) | 3-arm parallel, open label RCT | 2 weeks | 17 | Olanzapine | Brief weight management education was provided at baseline. | 27.0 (4.5) | Cognitive dietary restraint | EI/TEFQ subscale 1 | Difference in mean TEFQ subscale 1 scores between baseline and week 2 | 11.3 | NR | 7.60 | 5.20 | **3.70*** | 0.09 | NR |
| **NORMAL** | (Kang et al., 2024) | 2-arm parallel, double-blind RCT | 5 days | 19 | Olanzapine | No previous AP treatment | 21.70 (3.71) | Cognitive dietary restraint | TEFQ-R21 cognitive restraint subscale | Difference in mean TEFQ -R21 cognitive restraint subscale scores between baseline and day 6 | 15.48 | 1.49 | 15.64 | 1.23 | **-0.16*** | 0.674 | NR |
| **HIGH** | (Stip et al., 2012) | Non-randomised controlled trial | 16 weeks | 15 | Olanzapine | No washout period | NR | Cognitive dietary restraint | TEFQ subscale 1 | Difference in mean TEFQ subscale 1 scores between weeks 1 and 16 | 8.40 | 3.83 | 8.60 | 3.83 | **-0.2*** | 0.64* | NR |
| **DISINHIBITION** | | | | | | | | | | | | | | | | |  |
| **HIGH** | Case et al., 2010  Study 3 (Hoffman et al., 2009) | 3-arm parallel, open label RCT | 2 weeks | 17 | Olanzapine | Brief weight management education was provided at baseline. | 27.0 (4.5) | Disinhibition | EI/TEFQ subscale 2 | Difference in mean TEFQ subscale 2 scores between baseline and week 2 | 5.4 | NR | 8.70 | 4.60 | **-3.30*** | 0.16 | NR |
| **HIGH** | (Stip et al., 2012) | Non-randomised controlled trial | 16 weeks | 15 | Olanzapine |  | NR | Disinhibition | TEFQ subscale 2 | Difference in mean TEFQ subscale 2 scores between weeks 1 and 16 | 5.46 | 2.85 | 5.46 | 2.69 | **0*** | 0.54* | NR |
| ***HEALTHY VOLUNTEERS*** | | | | | | | | | | | | | | | | |  |
| **HIGH** | (Mathews et al., 2012) | Pre-post study | 7 days | 19 | Olanzapine | Healthy volunteers | 25.78 (4.82) | Disinhibition | TEFQ subscale 2 | Difference in mean disinhibited eating subscale score before and after the 7-day olanzapine treatment | 17.94 | 4.98 | 16.50 | 3.97 | **1.44*** | 0.03 | NR |
| **HUNGER** | | | | | | | | | | | | | | | | |  |
| **HIGH** | Case et al., 2010  Study 3 (Hoffman et al., 2009) | 3-arm parallel, open label RCT | 2 weeks | 17 | Olanzapine | Brief weight management education was provided at baseline. | 27.0 (4.5) | Susceptibility to hunger | EI/TEFQ subscale 3 | Difference in mean TEFQ subscale 3 scores between baseline and week 2 | 4.8 | NR | 7.90 | 4.50 | **-3.1*** | 0.17 | NR |
| **HIGH** | (Stip et al., 2012) | Non-randomised controlled trial | 16 weeks | 15 | Olanzapine |  | NR | Susceptibility to hunger | TEFQ subscale 3 | Difference in mean TEFQ subscale 3 scores between weeks 1 and 16 | 4.13 | 3.04 | 4.06 | 2.84 | **0.07*** | 0.54* | NR |
| **UNCONTROLLED EATING** | | | | | | | | | | | | | | | | | |
| **NORMAL** | (Kang et al., 2024) | 2-arm parallel, double-blind RCT | 5 days | 19 | Olanzapine | No previous AP treatment | 21.70 (3.71) | Uncontrolled eating | TEFQ-R21 uncontrolled eating subscale | Difference in mean TEFQ-R21 uncontrolled eating subscale scores between baseline and day 6 | 22.43 | 3.26 | 23.57 | 4.11 | **-1.14** | 0.143 | NR |
| **EMOTIONAL EATING** | | | | | | | | | | | | | | | | | |
| **NORMAL** | (Kang et al., 2024) | 2-arm parallel, double-blind RCT | 5 days | 19 | Olanzapine | No previous AP treatment | 21.70 (3.71) | Emotional eating | TEFQ-R21 emotional eating subscale | Difference in mean TEFQ-R21 emotional eating subscale scores between baseline and day 6 | 15.99 | 2.58 | 16.92 | 2.48 | **-0.92** | 0.04 | NR |

Mean difference (or difference in means) in within-group analyses= posttreatment mean – baseline mean

%= percentage; CI= confidence interval; EI= Eating Inventory; g= gram; kcal= kilocalories; M= mean; n= number; NR= not reported; RCT= randomised controlled trial; SD= standard deviation; SE= standard error; TFEQ= Three-Factor eating Questionnaire.

## Between-group syntheses - Summary of effect measures of antipsychotics on appetite sensations

| BMI | Citation | Study design | End of study assessment | Sample size | Antipsychotic | Duration of prior AP treatment/ other medications | Baseline BMI,  M (SD) | Outcome of interest | Measurement scale | Analysis | AP-treated group mean | AP-treated group SD | Unexposed group mean | Unexposed group SD | Mean difference | p-value | OR [p, 95% CI]/or 95% CI for between-group mean differences |
| --- | --- | --- | --- | --- | --- | --- | --- | --- | --- | --- | --- | --- | --- | --- | --- | --- | --- |
| **APPETITE** | | | | | | | | | | | | | | | | | |
| ***HEALTHY VOLUNTEERS*** | | | | | | | | | | | | | | | | | |
| **NORMAL** | (Roerig et al., 2005) | 3-arm parallel, double-blind RCT | 2 weeks | Risperidone /placebo  n= 16/16 | Risperidone | Healthy volunteers | Risperidone arm:  24.969 (NR)  Placebo arm: 24.106 (NR) | Self-reported increased appetite as an adverse effect | Self-report | Odds of increased appetite in risperidone arm compared to placebo arm over the 2-week period. | NR | NR | NR | NR | NR | NR | Odds of increased appetite in risperidone arm= 7/9; in placebo arm= 4/12 (OR: 2.33, CI: 0.52, 10.48).* |
|  | (Roerig et al., 2005) | 3-arm parallel, double-blind RCT | 2 weeks | Olanzapine/ placebo  n= 16/16 | Olanzapine | Healthy volunteers | Olanzapine arm:  23.625 (NR)  Placebo arm: 24.106 (NR) | Self-reported increased appetite as an adverse effect | Self-report | Odds of increased appetite in olanzapine arm compared to placebo arm over the 2-week period. | NR | NR | NR | NR | NR | NR | Odds of increased appetite in olanzapine arm= 6/10; in placebo arm= 4/12 (OR: 1.80, CI: 0.39, 8.22) * |
| **CRAVING** | | | | | | | | | | | | | | | | | |
| **General food craving** | | | | | | | | | | | | | | | | | |
| **HIGH** | (Abbas and Liddle, 2013) | Cross-sectional | NA | Olanzapine-treated group/ unexposed group  n= 20/20 | Olanzapine | Duration of AP treatment in months, mean (SD): 15.10 (19).  Use of other medications (SSRI, TCA, mirtazapine, venlafaxine), n=8. | Olanzapine-treated group:  29.50 (5.40)    Unexposed group:  25.80 (5.0) | Craving (natural setting)  General food craving | FCI | Difference in general food craving score means between olanzapine-treated group and unexposed group | 1.95 | 0.70 | 1.88 | 0.53 | **0.07*** | 0.72* | (-0.33, 0.47)* |
|  | (Abbas and Liddle, 2013) | Cross-sectional | NA | FGA-treated group/ unexposed group  n= 20/20 | FGA | Duration of AP treatment in months, mean (SD): 19.7 (11).  Use of other medications (SSRI, TCA, mirtazapine, venlafaxine), n= 7 | FGA-treated group:  27.30 (5.40)  Unexposed group:  25.8 (5.0) | Craving (natural setting)  General food craving | FCI | Difference in general food craving score means between FGA-treated group and unexposed group | 2.23 | 0.86 | 1.88 | 0.53 | **0.35*** | 0.13* | (-0.11, 0.81)* |
| **Carbohydrates** | | | | | | | | | | | | | | | | | |
| **HIGH** | (Abbas and Liddle, 2013) | Cross-sectional | NA | Olanzapine-treated group/ unexposed group  n= 20/20 | Olanzapine | Duration of AP treatment in months, mean (SD): 15.10 (19).  Use of other medications (SSRI, TCA, mirtazapine, venlafaxine), n=8. | Olanzapine-treated group:  29.50 (5.40)    Unexposed group:  25.80 (5.0) | Craving (natural setting)  General food craving | FCI | Difference in carbohydrate craving score means between olanzapine-treated group and unexposed group | 2.09 | 0.88 | 1.94 | 0.78 | **0.15*** | 0.57* | (-0.38, 0.68)* |
|  | (Abbas and Liddle, 2013) | Cross-sectional | NA | FGA-treated group/ unexposed group  n= 20/20 | FGA | Duration of AP treatment in months, mean (SD): 19.7 (11).  Use of other medications (SSRI, TCA, mirtazapine, venlafaxine), n= 7 | FGA-treated group:  27.30 (5.40)  Unexposed group:  25.8 (5.0) | Craving (natural setting)  General food craving | FCI | Difference in carbohydrate craving score means between FGA-treated group and unexposed group | 2.37 | 0.98 | 1.94 | 0.78 | **0.43*** | 0.13* | (-0.13, 1.00)* |
| **Sweets** | | | | | | | | | | | | | | | | |  |
| **HIGH** | (Abbas and Liddle, 2013) | Cross-sectional | NA | Olanzapine-treated group/ unexposed group  n= 20/20 | Olanzapine | Duration of AP treatment in months, mean (SD): 15.10 (19).  Use of other medications (SSRI, TCA, mirtazapine, venlafaxine), n=8. | Olanzapine-treated group:  29.50 (5.40)    Unexposed group:  25.80 (5.0) | Craving (natural setting)  General food craving | FCI | Difference in sweet craving score means between olanzapine-treated group and unexposed group | 2.03 | 0.89 | 2.07 | 0.71 | **-0.04*** | 0.88* | (-0.56, 0.48)* |
|  | (Abbas and Liddle, 2013) | Cross-sectional | NA | FGA-treated group /unexposed group  n= 20/20 | FGA | Duration of AP treatment in months, mean (SD): 19.7 (11).  Use of other medications (SSRI, TCA, mirtazapine, venlafaxine), n= 7 | FGA-treated group:  27.30 (5.40)  Unexposed group:  25.8 (5.0) | Craving (natural setting)  General food craving | FCI | Difference in sweet craving score means between FGA-treated group and unexposed group | 2.18 | 0.90 | 2.07 | 0.71 | **0.11*** | 0.67* | (-0.41, 0.63)* |
| **Fast-food fats (FFF)** | | | | | | | | | | | | | | | | | |
| **HIGH** | (Abbas and Liddle, 2013) | Cross-sectional | NA | Olanzapine-treated group/ unexposed group  n= 20/20 | Olanzapine | Duration of AP treatment in months, mean (SD): 15.10 (19).  Use of other medications (SSRI, TCA, mirtazapine, venlafaxine), n=8. | Olanzapine-treated group:  29.50 (5.40)    Unexposed group:  25.80 (5.0) | Craving (natural setting)  General food craving | FCI | Difference in FFF craving score means between olanzapine-treated group and unexposed group | 1.90 | 0.67 | 2.00 | 0.60 | **-0.10*** | 0.62* | (-0.51, 0.31) |
|  | (Abbas and Liddle, 2013) | Cross-sectional | NA | FGA-treated group/ unexposed group  n= 20/20 | FGA | Duration of AP treatment in months, mean (SD): 19.7 (11).  Use of other medications (SSRI, TCA, mirtazapine, venlafaxine), n= 7 | FGA-treated group:  27.30 (5.40)  Unexposed group:  25.8 (5.0) | Craving (natural setting)  General food craving | FCI | Difference in FFF craving score means between FGA-treated group and unexposed group | 2.25 | 0.69 | 2.00 | 0.60 | **0.25*** | 0.23* | (-0.16, 0.66)* |
| **High fats** | | | | | | | | | | | | | | | | |  |
| **HIGH** | (Abbas and Liddle, 2013) | Cross-sectional | NA | Olanzapine-treated group/ unexposed group  n= 20/20 | Olanzapine | Duration of AP treatment in months, mean (SD): 15.10 (19).  Use of other medications (SSRI, TCA, mirtazapine, venlafaxine), n=8. | Olanzapine-treated group:  29.50 (5.40)    Unexposed group:  25.80 (5.0) | Craving (natural setting)  General food craving | FCI | Difference in high fats craving score means between olanzapine-treated group and unexposed group | 1.73 | 0.67 | 1.58 | 0.59 | **0.15*** | 0.46* | (-0.25, 0.55)* |
|  | (Abbas and Liddle, 2013) | Cross-sectional | NA | FGA-treated group/ unexposed group  n= 20/20 | FGA | Duration of AP treatment in months, mean (SD): 19.7 (11).  Use of other medications (SSRI, TCA, mirtazapine, venlafaxine), n= 7 | FGA-treated group:  27.30 (5.40)  Unexposed group:  25.8 (5.0) | Craving (natural setting)  General food craving | FCI | Difference in high fats craving score means between FGA-treated group and unexposed group | 2.15 | 1.00 | 1.58 | 0.59 | **0.57*** | 0.03* | (0.04, 1.10)* |
| **HUNGER** | | | | | | | | | | | | | | | | | |
| **HIGH** | (Blouin et al., 2008) | Cross-sectional | NA | SGA-treated group/ Unexposed group  n= 18/20 | SGAs including clozapine: n = 2; olanzapine:  n = 9; risperidone: n = 2; quetiapine: n = 3; ziprasidone: n = 2. | Duration of current SGA treatment in months, mean (SD): 24.60 (19.70).  Duration of prior AP exposure (FGA or SGA) in SGA-treated group months, mean (SD): 35.30 (22.00).  SGA-treated group had to be sedentary (practicing <30 min of continuous physical activity per week).  Unexposed group matched by age and physical activity. | SGA-treated group:  28.80 (5.0)  Unexposed group:  25.0 (3.30) | Hunger (lab setting)  Post-prandial degree of hunger (during 60 minutes after the standardised breakfast) | VAS | Difference in degree of hunger after the standardised breakfast between SGA-treated group and unexposed group, after adjusting for baseline values. | NR | NR | NR | NR | **positive** (presented as a graph) | 0.03 (adj for baseline BMI) | NA |
| ***HEALTHY VOLUNTEERS*** | | | | | | | | | | | | | | | | | |
| **NORMAL** | (Ballon et al., 2018) | 3-arm double-blind, parallel RCT | 28 days | Olanzapine/ placebo  n= 7/10 | Olanzapine | Healthy volunteers | Olanzapine arm:  22.70 (0.30)  Placebo:  22.70 (0.50) | Premeal hunger (lab setting) | VAS | Difference in premeal hunger scores between olanzapine and placebo arms on day 28 | 89 | 11 | 88 | 10 | **1.00*** | 0.85 * | (-9.94, 11.94)* |
|  | (Ballon et al., 2018) | 3-arm double-blind, parallel RCT | 28 days | Iloperidone/ placebo  n= 7/10 | Iloperidone | Healthy volunteers | Iloperidone arm:  22.70 (0.30)  Placebo arm: 22.70 (0.50) | Premeal hunger (lab setting) | VAS | Difference in premeal hunger scores between iloperidone and placebo arms on day 28 | 86 | 19 | 88 | 10 | **-2.00*** | 0.81 * | (-20.00, 16.00)* |
|  | (Teff et al., 2015) | 3-arm parallel, double-blind RCT | 12 days | Olanzapine/ placebo  n=10/10 | Olanzapine | Healthy volunteers | Olanzapine arm:  22.10 (1.40)  Placebo arm: 21.80 (1.90) | Hunger (lab setting)  Total hunger score over the 12-day follow-up period | VAS | Difference in total hunger score between olanzapine and placebo arms | NR | NR | NR | NR | **positive** (presented as graph) | NR | NA |
|  | (Teff et al., 2015) | 3-arm parallel, double-blind RCT | 12 days | Aripiprazole/ placebo  n=10/10 | Aripiprazole | Healthy volunteers | Aripiprazole arm:  22.40 (1.30)  Placebo arm: 21.8j0 (1.90) | Hunger (lab setting)  Total hunger score over the 12-day follow-up period. | VAS | Difference in total hunger score between aripiprazole and placebo arms | NR | NR | NR | NR | **negative**  (presented as graph) | NR | NA |
| **Satiety Quotient** | | | | | | | | | | | | | | | | | |
| **HIGH** | (Blouin et al., 2008) | Cross-sectional | NA | SGA-treated group/ Unexposed group  n= 18/20 | SGAs including clozapine: n = 2; olanzapine:  n = 9; risperidone: n = 2; quetiapine: n = 3; ziprasidone: n = 2. | Duration of current SGA treatment in months, mean (SD): 24.60 (19.70).  Duration of prior AP exposure (FGA or SGA) in SGA-treated group months, mean (SD): 35.30 (22.00).  SGA-treated group had to be sedentary (practicing <30 min of continuous physical activity per week).  Unexposed group matched by age and physical activity. | SGA-treated group:  28.80 (5.0)  Unexposed group:  25.0 (3.30) | SQ (lab setting)  Mean postprandial SQ (satiating efficiency) of standardised breakfast on the degree of hunger | VAS, calculated total energy | Difference in mean postprandial SQ after standardised breakfast between SGA-treated group and unexposed group, after adjusting for BMI. | NR | NR | NR | NR | **negative** (presented as graph)) | 0.002 (adj for BMI) | NA |
|  | (Blouin et al., 2008) | Cross-sectional | NA | SGA-treated group/ Unexposed group  n= 18/20 | SGAs including clozapine: n = 2; olanzapine:  n = 9; risperidone: n = 2; quetiapine: n = 3; ziprasidone: n = 2. | Duration of current SGA treatment in months, mean (SD): 24.60 (19.70).  Duration of prior AP exposure (FGA or SGA) in SGA-treated group months, mean (SD): 35.30 (22.00).  SGA-treated group had to be sedentary (practicing <30 min of continuous physical activity per week).  Unexposed group matched by age and physical activity. | SGA-treated group:  28.80 (5.0)  Unexposed group:  25.0 (3.30) | SQ (lab setting)  Satiety Quotient (SQ) for degree of hunger measured immediately after ad-libitum buffet-type meal | VAS, calculated total energy | Difference in mean postprandial SQ immediately after ad-libitum buffet-type meal between SGA-treated group and unexposed group | 9.80 | 7.50 | 8.80 | 3.80 | **1.00*** | 0.61* | (-3.04, 5.04)* |
|  | (Blouin et al., 2008) | Cross-sectional | NA | SGA-treated group/ Unexposed group  n= 18/20 | SGAs including clozapine: n = 2; olanzapine:  n = 9; risperidone: n = 2; quetiapine: n = 3; ziprasidone: n = 2. | Duration of current SGA treatment in months, mean (SD): 24.60 (19.70).  Duration of prior AP exposure (FGA or SGA) in SGA-treated group months, mean (SD): 35.30 (22.00).  SGA-treated group had to be sedentary (practicing <30 min of continuous physical activity per week).  Unexposed group matched by age and physical activity. | SGA-treated group:  28.80 (5.0)  Unexposed group:  25.0 (3.30) | SQ (lab setting)  Satiety quotient (SQ) for prospective food measured immediately after ad-libitum buffet-type meal | VAS, calculated total energy | Difference in mean postprandial SQ immediately after ad-libitum buffet-type meal between SGA-treated group and unexposed group | 11.00 | 7.80 | 8.00 | 3.80 | **3.00*** | 0.15* | (-1.18, 7.18)* |
| **FULLNESS** | | | | | | | | | | | | | | | | | |
| **NORMAL** | (Teff et al., 2015) | 3-arm parallel, double-blind RCT | 12 days | Olanzapine/ placebo  n=10/10 | Olanzapine | Healthy volunteers | Olanzapine arm:  22.10 (1.40)  Placebo arm: 21.80 (1.90) | Fullness (lab setting)  Total fullness score over the 12-day follow-up period | VAS | Difference in total fullness score between olanzapine and placebo arms | NR | NR | NR | NR | **negative** (presented graph) | NR | NA |

Mean difference (or difference in means) in between-group analyses= AP-treated group mean – unexposed group mean

a= unvalidated measurement scale; b= collective scale (i.e., measure appetite and food craving or eating cognitions); *= calculated/converted from data reported in record.

%= percentage; adj= adjusted; AP= antipsychotic; BMI= body mass index; CI= confidence interval; FCI= Food Craving Inventory; M= mean; n= number; NA= not applicable; NR= not reported; OR= odds ratio; SD= standard deviation; SQ= satiety quotient; SSRI= selective serotonin reuptake inhibitor; TCA= tricyclic antidepressant, VAS= visual analogue scale

## Between-group syntheses - Summary of effect measures of antipsychotics on food intake and dietary composition

| BMI | Citation | Study design | End of study assessment | Sample size | Antipsychotic | Duration of prior AP treatment/other medications | Baseline BMI, M (SD) | Outcome of interest | Measurement scale | Analysis | AP-treated group mean | AP-treated group SD | Unexposed group mean | Unexposed group SD | **Mean difference** | p-value | OR [p, 95% CI] /or 95% CI for between-group mean differences |
| --- | --- | --- | --- | --- | --- | --- | --- | --- | --- | --- | --- | --- | --- | --- | --- | --- | --- |
|  | **ENERGY INTAKE** | | | | | | | | | | | | | | | | |
| **HIGH** | (Blouin et al., 2008) | Cross-sectional | NA | SGA-treated group/Unexposed group  n= 18/20 | SGAs including clozapine: n = 2; olanzapine:  n = 9; risperidone: n = 2; quetiapine: n = 3; ziprasidone: n = 2. | Duration of current SGA treatment in months, mean (SD): 24.60 (19.70).  Duration of prior AP exposure (FGA or SGA) in SGA-treated group months, mean (SD): 35.30 (22.00).  SGA-treated group had to be sedentary (practicing <30 min of continuous physical activity per week).  Unexposed group matched by age and physical activity. | SGA-treated group:  28.80 (5.0)  Unexposed group:  25.0 (3.30) | Energy intake (kcal)  Lab-setting (ad libitum buffet-type meal) | Food was weighed before and after the buffet. Energy and nutrient intakes were calculated using the Canadian Nutrient File and/or information on food labels. | Difference in mean total caloric intake between SGA-treated group and unexposed group | 850 | 318 | 983 | 292 | **-133*** | 0.19* | (-333.68, 67.68)* |
|  | (Henderson et al., 2006) | Cross-sectional | NA | SGA-treated group/Unexposed group  n= 88/723 | Olanzapine: n= 42; clozapine: n=25; risperidone: n= 15; quetiapine: n=3; ziprasidone: n= 1; med-free: n=2 | Duration of treatment in SGA-treated group: NR | SGA-treated group: 31.30 (12.67)  Unexposed group: 28.30 (6.62) | Energy intake (kcal)  Natural setting | SGA-treated group: 4-day dietary record.  Unexposed group: 24-hour recall method | Difference in mean total caloric intake between SGA-treated group and unexposed group. | 1943 | 913 | 2630 | 1384 | **-687*** | 0.00001 | (-984.25, -389.75)* |
|  | (Jakobsen et al., 2018) | Cross-sectional | NA | SGA-treated group/ Unexposed group  n=346/3016 | Olanzapine, clozapine, quetiapine | Duration of treatment in SGA-treated group: NR.  Use of other medications: NR | SGA-treated group: NR.  Having a waist circumference ≥88 cm for females and ≥102 cm for males was an inclusion criterion.  Unexposed group: NR | Energy intake (MJ/day)  Natural setting | AP-treated group: 24-hr recall.  Unexposed group: a food record kept for 7 consecutive days. | Difference in mean total caloric intake between SGA-treated and unexposed groups | 8.20  (1959.85 kcal) | 3.50  (836.52 kcal) | 9.80  (2342.26 kcal) | 3.10  (740.92 kcal) | **-1.60***  (-382.41 kcal)* | <0.001 | (-1.95, -1.25)*  (-8193.26, 7428.44)* |
|  | (Nunes et al., 2014) | Cross-sectional | NA | AP-treated group/Unexposed group  n= 25/25 | FGA: n= 7; SGA: n= 17; used both FGA and SGA: n= 1. | Duration of AP treatment: NR.  Use of other medications: NR | AP-treated group: 29.09 (6.30)  Unexposed group: 26.91 (4.39) | Energy intake (kcal)  Natural setting | Food frequency questionnaire | Difference in mean total caloric intake between AP-treated group and unexposed group. | 3305 | 1113 | 2692 | 661 | **613.00*** | 0.02* | (89.34, 1136.64)* |
|  | (Stefanska et al., 2017) | Cross-sectional | NA | Female AP-treated/ female unexposed:  n= 32/60 | FGAs, SGAs | Duration of treatment in AP-treated group: ≥ 1 year.  Use of other medications: NR | AP-treated female group: 27.2 (5.7),  Female unexposed group: 25.8 (5.2), | Energy intake (kcal/day)  Natural setting | 24-hr recall | Difference in mean total caloric intake between AP-treated and unexposed groups | 1923.80 | 573.90 | 1777.70 | 562.00 | **146.10*** | 0.24* | (-100.10, 392.30)* |
|  | (Stefanska et al., 2017) | Cross-sectional | NA | Male AP-treated/male unexposed  n= 28/38 | FGAs, SGAs | Duration of treatment in AP-treated group: ≥ 1 year.  Use of other medications: NR | AP-treated male group: 27.6 (5.9)  Male unexposed group: 27.3 (5.7) | Energy intake (kcal/day)  Natural setting | 24-hr recall | Difference in mean total caloric intake between AP-treated and unexposed groups | 2323.70 | 568.70 | 2179.00 | 710.60 | **144.70*** | 0.38* | (-180.95, 470.35)* |
|  | (Stefanska et al., 2018) | Cross-sectional | NA | Female AP-treated/ female unexposed:  n=45/40 | FGAs, SGAs | Duration of treatment in AP-treated group: ≥ 1 year.  Use of other medications: NR | AP-treated female group: 25.1 (5.3)  Female unexposed group: 24.4 (5.0) | Energy intake (kcal/day)  Natural setting | 24-hr recall | Difference in mean total caloric intake between AP-treated and unexposed groups | 1785.00 | 558.30 | 1478.50 | 446.30 | **306.50*** | 0.01* | (86.61, 526.39)* |
|  | (Stefanska et al., 2018) | Cross-sectional | NA | Male AP-treated/male unexposed  n=40/30 | FGAs, SGAs | Duration of treatment in AP-treated group: ≥ 1 year.  Use of other medications: NR | AP-treated male group: 25.0 (4.6)  Male unexposed group: 25.9 (4.2) | Energy intake (kcal/day)  Natural setting | 24-hr recall | Difference in mean total caloric intake between AP-treated and unexposed groups | 2052.40 | 639.90 | 2464.90 | 800.80 | **-412.50*** | 0.02* | (-756.12, -68.88)* |
| **NORMAL** | (Saugo et al., 2020) | Cross-sectional analysis of data collected from subsample of GETUP study (prospective 9-month study) | NA | Female AP-treated/ female unexposed:  n=21/1245 | FGA: n=6; SGA: n=14; both: n=1 | FEP patients; duration of treatment: NR. | AP-treated female group: 21.40 (2.10)  Female unexposed group: 23.20 (3.80). | Energy (kcal/day)  Natural setting | AP-treated group: EPIC Questionnaire  Unexposed group: self-recorded food consumption for 3 consecutive days. | Difference in mean total caloric intake between AP-treated female and unexposed groups | 2134 | 1040 | 1939 | 526 | **195*** | 0.40 | NA (data were not normally distributed, non-parametric tests applied) |
|  | (Saugo et al., 2020) | Cross-sectional analysis of data collected from subsample of GETUP study (prospective 9-month study) | NA | Male AP-treated/male unexposed  n=33/1068 | FGA: n=5; SGA: n=25; both: n= 3 | FEP patients; duration of treatment: NR. | AP-treated male group: 24.50 (4.40)  Male unexposed group: 25.40 (3.40) | Energy (kcal/day)  Natural setting | AP-treated group: EPIC Questionnaire  Unexposed group: self-recorded food consumption for 3 consecutive days. | Difference in mean total caloric intake between AP-treated male and unexposed groups | 2386 | 1050 | 2390 | 650 | **- 4.0*** | 0.99 | NA (data were not normally distributed, non-parametric tests applied) |
| ***HEALTHY VOLUNTEERS*** | | | | | | | | | | | | | | | | | |
| **NORMAL** | (Fountaine et al., 2010) | Randomised, double blind, placebo controlled 2- period crossover trial | 15 days, 15 days (12-day washout) | N= 30 (21 completers) | Olanzapine | Healthy volunteers | 22.60 (2.20) | Energy intake (kcal)  Lab setting | Energy intake (kcal) was calculated for all food consumed on day 14 of study periods 1 and 2. | Difference in mean total caloric intake between olanzapine and placebo arms on day 14 | 4230 | NR | 3860 | NR | **370** | **0.052** | NA |
|  | (Teff et al., 2013) | 3-arm parallel, double-blind RCT | 12 days | Olanzapine/  placebo  n=10/10 | Olanzapine | Healthy volunteers | Olanzapine arm:  22.10 (1.40) Placebo arm:  21.80 (1.90) | Energy intake (kcal)  Lab setting | Energy intake (kcal) was calculated for all food consumed on days 2 (pre-intervention) and 11 (post-intervention) | Difference in mean total caloric intake between olanzapine and placebo arms on day 11 (postintervention) | NR | NR | NR | NR | **Positive**  (presented as graph) | NA | NA |
|  | (Teff et al., 2013) | 3-arm parallel, double-blind RCT | 12 days | Aripiprazole/ placebo  n=10/10 | Aripiprazole | Healthy volunteers | Aripiprazole arm:  22.40 (1.30)  Placebo arm: 21.80 (1.90) | Caloric intake (kcal)  Lab setting | Energy intake (kcal) was calculated for all food consumed on days 2 (pre-intervention) and 11 (post-intervention) | Difference in mean total caloric intake between aripiprazole and placebo arms on day 11 (postintervention) | NR | NR | NR | NR | **Negative**  (presented as graph) | NA | NA |
| **FRUIT AND VEGETABLE** | | | | | | | | | | | | | | | | | |
| **HIGH** | (Archie et al., 2007) | Cross-sectional | NA | Exposed group/ Unexposed group  n=101/208 | FGA or SGA including  risperidone: n= 29; olanzapine: n= 26; clozapine: n= 16; combination: n=15; FGA: n=15 | Duration of treatment in cases not reported.  (FEP: n=11/101) | Exposed group: 28.88 (6.16)*  Unexposed group: NR | Fruit and vegetable intake (servings/day)  Natural setting | Fruit and Vegetable and Fibre Screener | OR of low fruit and vegetable intake in exposed group versus unexposed group | NR | NR | NR | NR | NR | NR | Low fruit and vegetable intake:  OR in exposed versus unexposed **0.52***  p=0.04* |
|  | (Jakobsen et al., 2018) | Cross-sectional | NA | SGA-treated group/ Unexposed group  n=346/3016 | SGAs including olanzapine, clozapine, quetiapine | Duration of treatment in SGA-treated group: NR.  Use of other medications: NR | SGA-treated group: NR.  Having a waist circumference ≥88 cm for females and ≥102 cm for males was an inclusion criterion.  Unexposed group: NR | Fruits and vegetable intake (g/day)  Natural setting | SGA-treated group: FFQ  Unexposed group: Food record kept for 7 consecutive days | Difference in mean fruit and vegetable intake between AP-treated and unexposed groups | 120.10 | 63.50 | 389.00 | NR | **-268.90*** | NR | NA |
| **FAT** | | | | | | | | | | | | | | | | | |
| **HIGH** | (Archie et al., 2007) | Cross-sectional | NA | Exposed group/ Unexposed group  n=101/208 | FGA or SGA including  risperidone: n= 29; olanzapine: n= 26; clozapine: n= 16; combination: n=15; FGA: n=15 | Duration of treatment in cases not reported.  (FEP: n=11/101) | Exposed group: 28.88 (6.16)*  Unexposed group: NR | Dietary fat intake (g/day)  Natural setting | Dietary Fat Screener | OR of high fat intake in cases compared to controls | NR | NR | NR | NR | NR | NR | OR of high fat intake in exposed versus unexposed: **2.48***  p= 0.0004* |
|  | (Blouin et al., 2008) | Cross-sectional | NA | SGA-treated group/Unexposed group  n= 18/20 | SGAs including clozapine: n = 2; olanzapine:  n = 9; risperidone: n = 2; quetiapine: n = 3; ziprasidone: n = 2. | Duration of current SGA treatment in months, mean (SD): 24.60 (19.70).  Duration of prior AP exposure (FGA or SGA) in SGA-treated group months, mean (SD): 35.30 (22.00).  SGA-treated group had to be sedentary (practicing <30 min of continuous physical activity per week).  Unexposed group matched by age and physical activity. | SGA-treated group:  28.80 (5.00)  Unexposed group:  25.0 (3.30) | Lipid intake (g)  Lab-setting (ad libitum buffet-type meal) | Food was weighed before and after the buffet. Energy and nutrient intakes were calculated using the Canadian Nutrient File and/or information on food labels. | Difference in mean lipid intake between SGA-treated group and unexposed group | 31.60 | 13.10 | 37.60 | 16.60 | **-6.00*** | 0.23* | (-15.92, 3.92)* |
|  | (Blouin et al., 2008) | Cross-sectional | NA | SGA-treated group/Unexposed group  n= 18/20 | SGAs including clozapine: n = 2; olanzapine:  n = 9; risperidone: n = 2; quetiapine: n = 3; ziprasidone: n = 2. | Duration of current SGA treatment in months, mean (SD): 24.60 (19.70).  Duration of prior AP exposure (FGA or SGA) in SGA-treated group months, mean (SD): 35.30 (22.00).  SGA-treated group had to be sedentary (practicing <30 min of continuous physical activity per week).  Unexposed group matched by age and physical activity. | SGA-treated group:  28.80 (5.00)  Unexposed group:  25.0 (3.30) | Lipid intake  (% of meal)  Lab-setting (ad libitum buffet-type meal) | Food was weighed before and after the buffet. Energy and nutrient intakes were calculated using the Canadian Nutrient File and/or information on food labels. | Difference in mean lipid intake (% of meal) between SGA-treated group and unexposed group | 33.30 | 4.60 | 33.40 | 8.00 | **-0.10*** | 0.96* | (-4.46, 4.26)* |
|  | (Henderson et al., 2006) | Cross-sectional | NA | SGA-treated group/Unexposed group  n= 88/723 | Olanzapine: n= 42; clozapine: n=25; risperidone: n= 15; quetiapine: n=3; ziprasidone: n= 1; med-free: n=2 | Duration of treatment in SGA-treated group: NR | SGA-treated group: 31.30 (12.67)  Unexposed group: 28.30 (6.62) | Total fat intake (g)  Natural setting | SGA-treated group: 4-day dietary record    Unexposed group: 24-hour recall | Difference in mean lipid intake between SGA-treated group and unexposed group | 75.00 | 40.10 | 96.50 | 59.70 | **-21.50*** | 0.001 | (-34.33, -8.67)* |
|  | (Jakobsen et al., 2018) | Cross-sectional | NA | SGA-treated group/ Unexposed group  n=346/3016 | SGAs including olanzapine, clozapine, quetiapine | Duration of treatment in SGA-treated group: NR.  Use of other medications: NR | AP-treated group: NR.  Having a waist circumference ≥88 cm for females and ≥102 cm for males was an inclusion criterion.  Unexposed group: NR | Fat intake (% of energy)  Natural setting | Exposed group: 24-hr recall  Unexposed group: food record kept for 7 consecutive days | Difference in mean lipid intake (% of energy) between SGA-treated and unexposed groups | 33.90 | 10.60 | 37.00 | 5.40 | **-3.10*** | <0.001 | (-3.78, -2.42)* |
|  | (Nunes et al., 2014) | Cross-sectional | NA | AP-treated group/Unexposed group  n= 25/25 | FGA: n= 7; SGA: n= 17; used both FGA and SGA: n= 1. | Duration of AP treatment: NR.  Use of other medications: NR | AP-treated group: 29.09 (6.30)  Unexposed group: 26.91 (4.39) | Fat intake (% of energy)  Natural setting | Food frequency questionnaire | Difference in fat intake (% of energy) between AP-treated group and unexposed group. | 24.9 | 3.4 | 30.0 | 7.4 | **-5.10*** | 0.004 | (-8.41,  -1.79)* |
|  | (Stefanska et al., 2017) | Cross-sectional | NA | Female AP-treated/ female unexposed:  n= 32/60 | FGAs, SGAs | Duration of treatment in AP-treated group: ≥ 1 year.  Use of other medications: NR | AP-treated female group: 27.2 (5.7),  Female unexposed group: 25.8 (5.2), | Fat intake (g/day)  Natural setting | 24-hr recall | Difference in mean total fat intake between AP-treated and unexposed groups | 64.0 | 25.9 | 52.1 | 28.4 | **11.90*** | 0.05* | (-0.87, 23.89)* |
|  | (Stefanska et al., 2017) | Cross-sectional | NA | Female AP-treated/ female unexposed:  n= 32/60 | FGAs, SGAs | Duration of treatment in AP-treated group: ≥ 1 year.  Use of other medications: NR | AP-treated female group: 27.2 (5.7),  Female unexposed group: 25.8 (5.2), | Fat intake (% of energy)  Natural setting | 24-hr recall | Difference in mean fat intake (% of energy) between AP-treated and unexposed groups | 30.8 | 9.3 | 31.6 | 10.3 | **-0.80*** | 0.71* | (-5.13, 3.53)* |
|  | (Stefanska et al., 2017) | Cross-sectional | NA | Male AP-treated/male unexposed  n= 28/38 | FGAs, SGAs | Duration of treatment in AP-treated group: ≥ 1 year.  Use of other medications: NR | AP-treated male group: 27.6 (5.9)  Male unexposed group: 27.3 (5.7) | Fat intake (g/day)  Natural setting | 24-hr recall | Difference in mean total fat intake between AP-treated and unexposed groups | 100.0 | 42.3 | 78.2 | 44.1 | **21.80*** | 0.05* | (0.23, 43.37)* |
|  | (Stefanska et al., 2017) | Cross-sectional | NA | Male AP-treated/male unexposed  n= 28/38 | FGAs, SGAs | Duration of treatment in AP-treated group: ≥ 1 year.  Use of other medications: NR | AP-treated male group: 27.6 (5.9)  Male unexposed group: 27.3 (5.7) | Fat intake (% of energy)  Natural setting | 24-hr recall | Difference in mean fat intake (% of energy) between AP-treated and unexposed groups | 37.4 | 10.5 | 31.3 | 10.6 | **6.10*** | 0.02* | (0.85, 11.35)* |
|  | (Stefanska et al., 2018) | Cross-sectional | NA | Female AP-treated/ female unexposed:  n=45/40 | FGAs, SGAs | Duration of treatment in AP-treated group: ≥ 1 year.  Use of other medications: NR | AP-treated female group: 25.1 (5.3)  Female unexposed group: 24.4 (5.0) | Fat intake (g/day)  Natural setting | 24-hr recall | Difference in mean total fat intake between AP-treated and unexposed groups | 60.0 | 23.0 | 49.3 | 25.6 | **10.70*** | 0.05* | (0.22, 21.12)* |
|  | (Stefanska et al., 2018) | Cross-sectional | NA | Female AP-treated/ female unexposed:  n=45/40 | FGAs, SGAs | Duration of treatment in AP-treated group: ≥ 1 year.  Use of other medications: NR | AP-treated female group: 25.1 (5.3)  Female unexposed group: 24.4 (5.0) | Fat intake (% of energy)  Natural setting | 24-hr recall | Difference in mean fat intake (& of energy) between AP-treated and unexposed groups | 31.0 | 7.9 | 30.3 | 10.1 | **0.70*** | 0.72* | (-3.19, 4.59)* |
|  | (Stefanska et al., 2018) | Cross-sectional | NA | Male AP-treated/male unexposed  n=40/30 | FGAs, SGAs | Duration of treatment in AP-treated group: ≥ 1 year.  Use of other medications: NR | AP-treated male group: 25.0 (4.6)  Male unexposed group: 25.9 (4.2) | Fat intake (g/day)  Natural setting | 24-hr recall | Difference in mean total fat intake between AP-treated and unexposed groups | 86.7 | 40.0 | 91.4 | 37.1 | **-4.70*** | 0.62* | (-23.39, 13.99)* |
|  | (Stefanska et al., 2018) | Cross-sectional | NA | Male AP-treated/male unexposed  n=40/30 | FGAs, SGAs | Duration of treatment in AP-treated group: ≥ 1 year.  Use of other medications: NR | AP-treated male group: 25.0 (4.6)  Male unexposed group: 25.9 (4.2) | Fat intake (% of energy)  Natural setting | 24-hr recall | Difference in mean fat intake (% of energy) between AP-treated and unexposed groups | 36.5 | 9.0 | 33.4 | 9.9 | **3.10*** | 0.18* | (-1.43, 7.63)* |
| **NORMAL** | (Saugo et al., 2020) | Cross-sectional analysis of data collected from subsample of GETUP study (prospective 9-month study) | NA | Female AP-treated/ female unexposed:  n=21/1245 | FGA: n=6; SGA: n=14; both: n=1 | Exposed group: FEP, had not been prescribed antipsychotics in the previous 3 months. | AP-treated female group: 21.40 (2.10)  Female unexposed group: 23.20 (3.80). | Fats (g/day)  Natural setting | Exposed group: EPIC Questionnaire  Unexposed group: self-recorded food consumption for 3 consecutive days | Difference in mean lipid intake between female exposed and unexposed groups. | 83.10 | 44.80 | 79.10 | 23.40 | **4.00*** | 0.863 | NA (data were not normally distributed, non-parametric tests applied) |
|  | (Saugo et al., 2020) | Cross-sectional analysis of data collected from subsample of GETUP study (prospective 9-month study) | NA | Male AP-treated/male unexposed  n=33/1068 | FGA: n=5; SGA: n=25; both: n= 3 | Exposed group: FEP, had not been prescribed antipsychotics in the previous 3 months. | AP-treated male group: 24.50 (4.40)  Male unexposed group: 25.40 (3.40) | Fats (g/day)  Natural setting | Exposed group: EPIC Questionnaire  Unexposed group: self-recorded food consumption for 3 consecutive days | Difference in mean lipid intake between male exposed and unexposed groups. | 95.20 | 45.90 | 95.40 | 29.50 | **-0.20*** | 0.978 | NA (data were not normally distributed, non-parametric tests applied) |
| **SFA** | | | | | | | | | | | | | | | | | |
| **HIGH** | (Henderson et al., 2006) | Cross-sectional | NA | SGA-treated group/Unexposed group  n= 88/723 | Olanzapine: n= 42; clozapine: n=25; risperidone: n= 15; quetiapine: n=3; ziprasidone: n= 1; med-free: n=2 | Duration of treatment in SGA-treated group: NR | SGA-treated group: 31.30 (12.67)  Unexposed group: 28.30 (6.62) | SFA intake (g)  Natural setting | SGA-treated group: 4-day dietary record  Unexposed group: 24-hour recall | Difference in mean saturated fat intake between SGA-treated group and unexposed group | 26.90 | 16.70 | 32.30 | 21.20 | **-5.40** | 0.02 | (-10.00, -0.80)* |
|  | (Jakobsen et al., 2018) | Cross-sectional | NA | SGA-treated group/ Unexposed group  n=346/3016 | SGAs including olanzapine, clozapine, quetiapine | Duration of treatment in SGA-treated group: NR.  Use of other medications: NR | SGA-treated group: NR.  Having a waist circumference ≥88 cm for females and ≥102 cm for males was an inclusion criterion.  Unexposed group: NR | SFA intake (% of energy)  Natural setting | SGA-treated group: 24-hr recall in the  Unexposed group: food record kept for 7 consecutive days | Difference in mean SFA intake between SGA-treated and unexposed groups | 25.70 | 17.40 | 15.00 | 2.90 | **10.70*** | <0.001 | (10.01, 11.39)* |
|  | (Nunes et al., 2014) | Cross-sectional | NA | AP-treated group/Unexposed group  n= 25/25 | FGA: n= 7; SGA: n= 17; used both FGA and SGA: n= 1. | Duration of AP treatment: NR.  Use of other medications: NR | AP-treated group: 29.09 (6.30)  Unexposed group: 26.91 (4.39) | SFA intake (% of energy)  Natural setting | Food frequency questionnaire | Difference in SFA intake (% of energy) between AP-treated group and unexposed group. | 8.8 | 1.3 | 10.1 | 2.6 | **-1.30*** | 0.03 | (-2.47, -0.13)* |
|  | (Stefanska et al., 2017) | Cross-sectional | NA | Female AP-treated/ female unexposed:  n= 32/60 | FGAs, SGAs | Duration of treatment in AP-treated group: ≥ 1 year.  Use of other medications: NR | AP-treated female group: 27.2 (5.7),  Female unexposed group: 25.8 (5.2), | SFA intake (g/day)  Natural setting | 24-hr recall | Difference in mean SFA intake between AP-treated and unexposed groups | 28.1 | 12.7 | 22.8 | 14.8 | **5.30*** | 0.09* | (-0.84, 11.45)* |
|  | (Stefanska et al., 2017) | Cross-sectional | NA | Female AP-treated/ female unexposed:  n= 32/60 | FGAs, SGAs | Duration of treatment in AP-treated group: ≥ 1 year.  Use of other medications: NR | AP-treated female group: 27.2 (5.7),  Female unexposed group: 25.8 (5.2), | SFA intake (% of energy)  Natural setting | 24-hr recall | Difference in mean SFA intake (% of energy) between AP-treated and unexposed groups | 13.0 | 5.9 | 11.5 | 7.4 | **1.50*** | 0.32* | (-1.51, 4.51)* |
|  | (Stefanska et al., 2017) | Cross-sectional | NA | Male AP-treated/male unexposed  n= 28/38 | FGAs, SGAs | Duration of treatment in AP-treated group: ≥ 1 year.  Use of other medications: NR | AP-treated male group: 27.6 (5.9)  Male unexposed group: 27.3 (5.7) | SFA intake (g/day)  Natural setting | 24-hr recall | Difference in mean SFA intake between AP-treated and unexposed groups | 49.3 | 22.7 | 32.1 | 15.4 | **17.20*** | 0.0005* | (7.83, 26.57)* |
|  | (Stefanska et al., 2017) | Cross-sectional | NA | Male AP-treated/male unexposed  n= 28/38 | FGAs, SGAs | Duration of treatment in AP-treated group: ≥ 1 year.  Use of other medications: NR | AP-treated male group: 27.6 (5.9)  Male unexposed group: 27.3 (5.7) | SFA intake (% of energy)  Natural setting | 24-hr recall | Difference in mean SFA intake (% of energy) between AP-treated and unexposed groups | 19.0 | 8.8 | 13.2 | 6.4 | **5.80*** | 0.003* | (2.07, 9.53)* |
|  | (Stefanska et al., 2018) | Cross-sectional | NA | Female AP-treated/ female unexposed:  n=45/40 | FGAs, SGAs | Duration of treatment in AP-treated group: ≥ 1 year.  Use of other medications: NR | AP-treated female group: 25.1 (5.3)  Female unexposed group: 24.4 (5.0) | SFA intake (g/day)  Natural setting | 24-hr recall | Difference in mean SFA intake between AP-treated and unexposed groups | 27.6 | 15.5 | 17.8 | 12.0 | **9.80*** | 0.002* | (3.76, 15.84)* |
|  | (Stefanska et al., 2018) | Cross-sectional | NA | Female AP-treated/ female unexposed:  n=45/40 | FGAs, SGAs | Duration of treatment in AP-treated group: ≥ 1 year.  Use of other medications: NR | AP-treated female group: 25.1 (5.3)  Female unexposed group: 24.4 (5.0) | SFA intake (% of energy)  Natural setting | 24-hr recall | Difference in mean SFA intake (% of energy) between AP-treated and unexposed groups | 13.4 | 4.1 | 10.8 | 4.7 | **2.60*** | 0.008* | (0.70, 4.50)* |
|  | (Stefanska et al., 2018) | Cross-sectional | NA | Male AP-treated/male unexposed  n=40/30 | FGAs, SGAs | Duration of treatment in AP-treated group: ≥ 1 year.  Use of other medications: NR | AP-treated male group: 25.0 (4.6)  Male unexposed group: 25.9 (4.2) | SFA intake (g/day)  Natural setting | 24-hr recall | Difference in mean SFA intake between AP-treated and unexposed groups | 38.6 | 20.9 | 37.6 | 16.3 | **1.00*** | 0.83* | (-8.19, 10.19)* |
|  | (Stefanska et al., 2018) | Cross-sectional | NA | Male AP-treated/male unexposed  n=40/30 | FGAs, SGAs | Duration of treatment in AP-treated group: ≥ 1 year.  Use of other medications: NR | AP-treated male group: 25.0 (4.6)  Male unexposed group: 25.9 (4.2) | SFA intake (% of energy)  Natural setting | 24-hr recall | Difference in mean SFA intake (% of energy) between AP-treated and unexposed groups | 16.9 | 3.9 | 13.7 | 3.1 | **3.20*** | 0.0004* | (1.47, 4.93)* |
| **NORMAL** | (Saugo et al., 2020) | Cross-sectional analysis of data collected from subsample of GETUP study (prospective 9-month study) | NA | Female AP-treated/ female unexposed:  n=21/1245 | FGA: n=6; SGA: n=14; both: n=1 | Exposed group: FEP, had not been prescribed antipsychotics in the previous 3 months. | AP-treated female group: 21.40 (2.10)  Female unexposed group: 23.20 (3.80).  N.B.: the reported mean BMI is that of the overall male AP-treated sample (n=38) | SFA intake  Natural setting | Exposed group: EPIC Questionnaire  Unexposed group: self-recorded food consumption for 3 consecutive days | Difference in mean SFA intake between female exposed and unexposed groups | 31.10 | 19.40 | 24.40 | 4.80 | **6.70*** | 0.131 | NA (data were not normally distributed, non-parametric tests applied) |
|  | (Saugo et al., 2020) | Cross-sectional analysis of data collected from subsample of GETUP study (prospective 9-month study) | NA | Male AP-treated/male unexposed  n=33/1068 | FGA: n=5; SGA: n=25; both: n= 3 | Exposed group: FEP, had not been prescribed antipsychotics in the previous 3 months. | AP-treated male group: 24.50 (4.40)  Male unexposed group: 25.40 (3.40)  N.B.: the reported mean BMI is that of the overall male AP-treated sample (n=58) | SFA intake  Natural setting | Exposed group: EPIC Questionnaire  Unexposed group: self-recorded food consumption for 3 consecutive days | Difference in mean SFA intake between male exposed and unexposed groups. | 36.40 | 20.40 | 29.70 | 11.30 | **6.70*** | 0.066 | NA (data were not normally distributed, non-parametric tests applied) |
| **MUFA** | | | | | | | | | | | | | | | | | |
| **HIGH** | (Henderson et al., 2006) | Cross-sectional | NA | SGA-treated group/Unexposed group  n= 88/723 | Olanzapine: n= 42; clozapine: n=25; risperidone: n= 15; quetiapine: n=3; ziprasidone: n= 1; med-free: n=2 | Duration of treatment in SGA-treated group: NR | SGA-treated group: 31.30 (12.67)  Unexposed group: 28.30 (6.62) | MUFA intake (g)  Natural setting | SGA-treated group: 4-day dietary record  Unexposed group: 24-hour recall method | Difference in mean MUFA intake between SGA-treated group and unexposed group | 28.20 | 15.50 | 37.40 | 24.40 | **-9.20*** | 0.001 | (-14.43, -3.97)* |
|  | (Nunes et al., 2014) | Cross-sectional | NA | AP-treated group/Unexposed group  n= 25/25 | FGA: n= 7; SGA: n= 17; used both FGA and SGA: n= 1. | Duration of AP treatment: NR.  Use of other medications: NR | AP-treated group: 29.09 (6.30)  Unexposed group: 26.91 (4.39) | MUFA intake (% of energy)  Natural setting | Food frequency questionnaire | Difference in MUFA intake (% of energy) between AP-treated group and unexposed group. | 9.1 | 1.7 | 11.5 | 3.7 | **-2.40*** | 0.005 | (-4.04, -0.76)* |
|  | (Stefanska et al., 2017) | Cross-sectional | NA | Female AP-treated/ female unexposed:  n= 32/60 | FGAs, SGAs | Duration of treatment in AP-treated group: ≥ 1 year.  Use of other medications: NR | AP-treated female group: 27.2 (5.7),  Female unexposed group: 25.8 (5.2), | MUFA intake (g/day)  Natural setting | 24-hr recall | Difference in mean MUFA intake between AP-treated and unexposed groups | 25.6 | 10.4 | 24.4 | 10.9 | **1.20*** | 0.61* | (-3.47, 5.87)* |
|  | (Stefanska et al., 2017) | Cross-sectional | NA | Female AP-treated/ female unexposed:  n= 32/60 | FGAs, SGAs | Duration of treatment in AP-treated group: ≥ 1 year.  Use of other medications: NR | AP-treated female group: 27.2 (5.7),  Female unexposed group: 25.8 (5.2), | MUFA intake (% of energy)  Natural setting | 24-hr recall | Difference in mean MUFA intake (% of energy) between AP-treated and unexposed groups | 12.0 | 4.8 | 12.3 | 5.5 | **-0.30*** | 0.80* | (-2.59, 1.99)* |
|  | (Stefanska et al., 2017) | Cross-sectional | NA | Male AP-treated/male unexposed  n= 28/38 | FGAs, SGAs | Duration of treatment in AP-treated group: ≥ 1 year.  Use of other medications: NR | AP-treated male group: 27.6 (5.9)  Male unexposed group: 27.3 (5.7) | MUFA intake (g/day)  Natural setting | 24-hr recall | Difference in mean MUFA intake between AP-treated and unexposed groups | 39.5 | 16.7 | 33.4 | 16.2 | **6.10*** | 0.14* | (-2.07, 14.27)* |
|  | (Stefanska et al., 2017) | Cross-sectional | NA | Male AP-treated/male unexposed  n= 28/38 | FGAs, SGAs | Duration of treatment in AP-treated group: ≥ 1 year.  Use of other medications: NR | AP-treated male group: 27.6 (5.9)  Male unexposed group: 27.3 (5.7) | MUFA intake (% of energy)  Natural setting | 24-hr recall | Difference in mean MUFA intake (% of energy) between AP-treated and unexposed groups | 15.3 | 6.5 | 13.8 | 6.7 | **1.50*** | 0.37* | (-1.79, 4.79)* |
|  | (Stefanska et al., 2018) | Cross-sectional | NA | Female AP-treated/ female unexposed:  n=45/40 | FGAs, SGAs | Duration of treatment in AP-treated group: ≥ 1 year.  Use of other medications: NR | AP-treated female group: 25.1 (5.3)  Female unexposed group: 24.4 (5.0) | MUFA intake (g/day)  Natural setting | 24-hr recall | Difference in mean MUFA intake between AP-treated and unexposed groups | 24.5 | 15.5 | 19.2 | 12.0 | **5.30*** | 0.08* | (-0.74, 11.34)* |
|  | (Stefanska et al., 2018) | Cross-sectional | NA | Female AP-treated/ female unexposed:  n=45/40 | FGAs, SGAs | Duration of treatment in AP-treated group: ≥ 1 year.  Use of other medications: NR | AP-treated female group: 25.1 (5.3)  Female unexposed group: 24.4 (5.0) | MUFA intake (% of energy)  Natural setting | 24-hr recall | Difference in mean MUFA intake (% of energy) between AP-treated and unexposed groups | 12 | 3.8 | 14.9 | 5.2 | **-2.90*** | 0.004* | (-4.85, -0.95)* |
|  | (Stefanska et al., 2018) | Cross-sectional | NA | Male AP-treated/male unexposed  n=40/30 | FGAs, SGAs | Duration of treatment in AP-treated group: ≥ 1 year.  Use of other medications: NR | AP-treated male group: 25.0 (4.6)  Male unexposed group: 25.9 (4.2) | MUFA intake (g/day)  Natural setting | 24-hr recall | Difference in mean MUFA intake between AP-treated and unexposed groups | 33.9 | 17.5 | 37.3 | 15.8 | **-3.40*** | 0.40* | (-11.49, 4.69)* |
|  | (Stefanska et al., 2018) | Cross-sectional | NA | Male AP-treated/male unexposed  n=40/30 | FGAs, SGAs | Duration of treatment in AP-treated group: ≥ 1 year.  Use of other medications: NR | AP-treated male group: 25.0 (4.6)  Male unexposed group: 25.9 (4.2) | MUFA intake (% of energy)  Natural setting | 24-hr recall | Difference in mean MUFA intake (% of energy) between AP-treated and unexposed groups | 14.7 | 4.1 | 13.6 | 3.0 | **1.10*** | 0.22* | (-0.67, 2.87)* |
| **NORMAL** | (Saugo et al., 2020) | Cross-sectional analysis of data collected from subsample of GETUP study (prospective 9-month study) | NA | Female AP-treated/ female unexposed:  n=21/1245 | FGA: n=6; SGA: n=14; both: n=1 | Exposed group: FEP, had not been prescribed antipsychotics in the previous 3 months. | AP-treated female group: 21.40 (2.10)  Female unexposed group: 23.20 (3.80). | MUFA intake  Natural setting | Exposed group: EPIC Questionnaire  Unexposed group: self-recorded food consumption for 3 consecutive days | Difference in mean MUFA intake between female exposed and unexposed groups. | 36.30 | 17.40 | 38.30 | 11.40 | **-2.00*** | 0.607 | NA (data were not normally distributed, non-parametric tests applied) |
|  | (Saugo et al., 2020) | Cross-sectional analysis of data collected from subsample of GETUP study (prospective 9-month study) | NA | Male AP-treated/male unexposed  n=33/1068 | FGA: n=5; SGA: n=25; both: n= 3 | Exposed group: FEP, had not been prescribed antipsychotics in the previous 3 months. | AP-treated male group: 24.50 (4.40)  Male unexposed group: 25.40 (3.40) | MUFA intake  Natural setting | Exposed group: EPIC Questionnaire  Unexposed group: self-recorded food consumption for 3 consecutive days | Difference in mean MUFA intake between male exposed and unexposed groups. | 41.40 | 18.50 | 45.40 | 13.90 | **-4.00*** | 0.172 | NA (data were not normally distributed, non-parametric tests applied) |
| **PUFA** | | | | | | | | | | | | | | | | | |
| **HIGH** | (Henderson et al., 2006) | Cross-sectional | NA | SGA-treated group/Unexposed group  n= 88/723 | Olanzapine: n= 42; clozapine: n=25; risperidone: n= 15; quetiapine: n=3; ziprasidone: n= 1; med-free: n=2 | Duration of treatment in SGA-treated group: NR | SGA-treated group: 31.30 (12.67)  Unexposed group: 28.30 (6.62) | PUFA intake (g)  Natural setting | SGA-treated group: 4-day dietary record  Unexposed group: 24-hour recall method | Difference in mean PUFA intake between SGA-treated group and unexposed group | 13.70 | 8.00 | 19.50 | 13.80 | **-5.80*** | 0.0001 | (-8.75, -2.85)* |
|  | (Nunes et al., 2014) | Cross-sectional | NA | AP-treated group/Unexposed group  n= 25/25 | FGA: n= 7; SGA: n= 17; used both FGA and SGA: n= 1. | Duration of AP treatment: NR.  Use of other medications: NR | AP-treated group: 29.09 (6.30)  Unexposed group: 26.91 (4.39) | PUFA intake (% of energy)  Natural setting | Food frequency questionnaire | Difference in PUFA intake (% of energy) between AP-treated group and unexposed group. | 4.5 | 1.2 | 5.9 | 2.5 | **-1.40*** | 0.016 | (-2.53, -0.27)* |
|  | (Stefanska et al., 2017) | Cross-sectional | NA | Female AP-treated/ female unexposed:  n= 32/60 | FGAs, SGAs | Duration of treatment in AP-treated group: ≥ 1 year.  Use of other medications: NR | AP-treated female group: 27.2 (5.7),  Female unexposed group: 25.8 (5.2), | PUFA intake (g/day)  Natural setting | 24-hr recall | Difference in mean PUFA intake between AP-treated and unexposed groups | 8.9 | 6.2 | 8.8 | 4.7 | **0.10*** | 0.93* | (-2.19, 2.39)* |
|  | (Stefanska et al., 2017) | Cross-sectional | NA | Female AP-treated/ female unexposed:  n= 32/60 | FGAs, SGAs | Duration of treatment in AP-treated group: ≥ 1 year.  Use of other medications: NR | AP-treated female group: 27.2 (5.7),  Female unexposed group: 25.8 (5.2), | PUFA intake (% of energy)  Natural setting | 24-hr recall | Difference in mean PUFA intake (% of energy) between AP-treated and unexposed groups | 4.2 | 2.9 | 4.4 | 2.3 | **-0.20*** | 0.72* | (-1.30, 0.90)* |
|  | (Stefanska et al., 2017) | Cross-sectional | NA | Male AP-treated/male unexposed  n= 28/38 | FGAs, SGAs | Duration of treatment in AP-treated group: ≥ 1 year.  Use of other medications: NR | AP-treated male group: 27.6 (5.9)  Male unexposed group: 27.3 (5.7) | PUFA intake (g/day)  Natural setting | 24-hr recall | Difference in mean PUFA intake between AP-treated and unexposed groups | 10.7 | 4.5 | 10.4 | 6.2 | **0.30*** | 0.83* | (-2.46, 3.06)* |
|  | (Stefanska et al., 2017) | Cross-sectional | NA | Male AP-treated/male unexposed  n= 28/38 | FGAs, SGAs | Duration of treatment in AP-treated group: ≥ 1 year.  Use of other medications: NR | AP-treated male group: 27.6 (5.9)  Male unexposed group: 27.3 (5.7) | PUFA intake (% of energy)  Natural setting | 24-hr recall | Difference in mean PUFA intake (% of energy) between AP-treated and unexposed groups | 4.1 | 1.7 | 4.3 | 2.6 | **-0.20*** | 0.72* | (-1.33, 0.93)* |
|  | (Stefanska et al., 2018) | Cross-sectional | NA | Female AP-treated/ female unexposed:  n=45/40 | FGAs, SGAs | Duration of treatment in AP-treated group: ≥ 1 year.  Use of other medications: NR | AP-treated female group: 25.1 (5.3)  Female unexposed group: 24.4 (5.0) | PUFA intake (g/day)  Natural setting | 24-hr recall | Difference in mean PUFA intake between AP-treated and unexposed groups | 8.0 | 7.0 | 8.4 | 5.0 | **-0.40*** | 0.77* | (-3.05, 2.25)* |
|  | (Stefanska et al., 2018) | Cross-sectional | NA | Female AP-treated/ female unexposed:  n=45/40 | FGAs, SGAs | Duration of treatment in AP-treated group: ≥ 1 year.  Use of other medications: NR | AP-treated female group: 25.1 (5.3)  Female unexposed group: 24.4 (5.0) | PUFA intake  (% of energy)  Natural setting | 24-hr recall | Difference in mean PUFA intake (% of energy) between AP-treated and unexposed groups | 3.9 | 2.1 | 4.8 | 2.3 | **-0.90*** | 0.06* | (-1.85, 0.05)* |
|  | (Stefanska et al., 2018) | Cross-sectional | NA | Male AP-treated/male unexposed  n=40/30 | FGAs, SGAs | Duration of treatment in AP-treated group: ≥ 1 year.  Use of other medications: NR | AP-treated male group: 25.0 (4.6)  Male unexposed group: 25.9 (4.2) | PUFA intake (g/day)  Natural setting | 24-hr recall | Difference in mean PUFA intake between AP-treated and unexposed groups | 8.0 | 4.2 | 12.3 | 5.1 | **-4.30*** | 0.0003* | (-6.52, -2.08)* |
|  | (Stefanska et al., 2018) | Cross-sectional | NA | Male AP-treated/male unexposed  n=40/30 | FGAs, SGAs | Duration of treatment in AP-treated group: ≥ 1 year.  Use of other medications: NR | AP-treated male group: 25.0 (4.6)  Male unexposed group: 25.9 (4.2) | PUFA intake (% of energy)  Natural setting | 24-hr recall | Difference in mean PUFA intake (% of energy) between AP-treated and unexposed groups | 3.5 | 1.7 | 4.5 | 2.0 | **-1.00*** | 0.03* | (-1.88, -0.12)* |
| **NORMAL** | (Saugo et al., 2020) | Cross-sectional analysis of data collected from subsample of GETUP study (prospective 9-month study) | NA | Female AP-treated/ female unexposed:  n=21/1245 | FGA: n=6; SGA: n=14; both: n=1 | Exposed group: FEP, had not been prescribed antipsychotics in the previous 3 months. | AP-treated female group: 21.40 (2.10)  Female unexposed group: 23.20 (3.80). | PUFA  Natural setting | Exposed group: EPIC Questionnaire  Unexposed group: self-recorded food consumption for 3 consecutive days. | Difference in mean PUFA intake between female exposed and unexposed groups. | 10.90 | 6.80 | 10.00 | 3.70 | **0.90*** | 0.559 | NA (data were not normally distributed, non-parametric tests applied) |
|  | (Saugo et al., 2020) | Cross-sectional analysis of data collected from subsample of GETUP study (prospective 9-month study) | NA | Male AP-treated/male unexposed  n=33/1068 | FGA: n=5; SGA: n=25; both: n= 3 | Exposed group: FEP, had not been prescribed antipsychotics in the previous 3 months. | AP-treated male group: 24.50 (4.40)  Male unexposed group: 25.40 (3.40) | PUFA  Natural setting | Exposed group: EPIC Questionnaire  Unexposed group: self-recorded food consumption for 3 consecutive days | Difference in mean PUFA intake between male exposed and unexposed groups | 11.20 | 5.70 | 12.20 | 4.60 | **-1.00*** | 0.341 | NA (data were not normally distributed, non-parametric tests applied) |
| **CHOLESTEROL** | | | | | | | | | | | | | | | | | |
| **HIGH** | (Henderson et al., 2006) | Cross-sectional | NA | SGA-treated group/Unexposed group  n= 88/723 | Olanzapine: n= 42; clozapine: n=25; risperidone: n= 15; quetiapine: n=3; ziprasidone: n= 1; med-free: n=2 | Duration of treatment in SGA-treated group: NR | SGA-treated group: 31.30 (12.67)  Unexposed group: 28.30 (6.62) | Cholesterol intake (mg)  Natural setting | SGA-treated group: 4-day dietary record  Unexposed group: 24-hour recall method | Difference in mean cholesterol intake between SGA-treated group and unexposed group | 276.40 | 204.90 | 334.90 | 292.20 | **-58.50** | 0.069 | (-121.46, 4.46)* |
|  | (Nunes et al., 2014) | Cross-sectional | NA | AP-treated group/Unexposed group  n= 25/25 | FGA: n= 7; SGA: n= 17; used both FGA and SGA: n= 1. | Duration of AP treatment: NR.  Use of other medications: NR | AP-treated group: 29.09 (6.30)  Unexposed group: 26.91 (4.39) | Cholesterol intake (mg/1000 kcal)  Natural setting | Food frequency questionnaire | Difference in mean cholesterol intake between AP-treated group and unexposed group. | 110.8 | 25.3 | 117.8 | 54.0 | **-7.00*** | 0.563 | (-30.98, 16.98)* |
|  | (Stefanska et al., 2017) | Cross-sectional | NA | Female AP-treated/ female unexposed:  n= 32/60 | FGAs, SGAs | Duration of treatment in AP-treated group: ≥ 1 year.  Use of other medications: NR | AP-treated female group: 27.2 (5.7),  Female unexposed group: 25.8 (5.2), | Cholesterol intake (g/day)  Natural setting | 24-hr recall | Difference in mean cholesterol intake between AP-treated and unexposed groups | 233.4 | 140.4 | 236.9 | 200.5 | **-3.50*** | 0.93* | (-82.67, 75.67)* |
|  | (Stefanska et al., 2017) | Cross-sectional | NA | Male AP-treated/male unexposed  n= 28/38 | FGAs, SGAs | Duration of treatment in AP-treated group: ≥ 1 year.  Use of other medications: NR | AP-treated male group: 27.6 (5.9)  Male unexposed group: 27.3 (5.7) | Cholesterol intake (g/day)  Natural setting | 24-hr recall | Difference in mean cholesterol intake between AP-treated and unexposed groups | 439.1 | 286.6 | 398.7 | 310.0 | **40.40*** | 0.59* | (-109.04, 189.84)* |
|  | (Stefanska et al., 2018) | Cross-sectional | NA | Female AP-treated/ female unexposed:  n=45/40 | FGAs, SGAs | Duration of treatment in AP-treated group: ≥ 1 year.  Use of other medications: NR | AP-treated female group: 25.1 (5.3)  Female unexposed group: 24.4 (5.0) | Cholesterol intake (g/day)  Natural setting | 24-hr recall | Difference in mean cholesterol intake between AP-treated and unexposed groups | 226.0 | 128.3 | 265.0 | 224.2 | **-39.00*** | 0.32 | (-116.73, 38.73)* |
|  | (Stefanska et al., 2018) | Cross-sectional | NA | Male AP-treated/male unexposed  n=40/30 | FGAs, SGAs | Duration of treatment in AP-treated group: ≥ 1 year.  Use of other medications: NR | AP-treated male group: 25.0 (4.6)  Male unexposed group: 25.9 (4.2) | Cholesterol intake (g/day)  Natural setting | 24-hr recall | Difference in mean cholesterol intake between AP-treated and unexposed groups | 379.5 | 261.9 | 465.8 | 331.6 | **-86.30*** | 0.23* | (-227.83, 55.23)* |
| **NORMAL** | (Saugo et al., 2020) | Cross-sectional analysis of data collected from subsample of GETUP study (prospective 9-month study) | NA | Female AP-treated/ female unexposed:  n=21/1245 | FGA: n=6; SGA: n=14; both: n=1 | Exposed group: FEP, had not been prescribed antipsychotics in the previous 3 months. | AP-treated female group: 21.40 (2.10)  Female unexposed group: 23.20 (3.80). | Cholesterol  Natural setting | Exposed group: EPIC Questionnaire  Unexposed group: self-recorded food consumption for 3 consecutive days | Difference in mean cholesterol intake between female exposed and unexposed groups. | 361.80 | 207.00 | 265.00 | 125.00 | **96.80*** | 0.044 | NA (data were not normally distributed, non-parametric tests applied) |
|  | (Saugo et al., 2020) | Cross-sectional analysis of data collected from subsample of GETUP study (prospective 9-month study) | NA | Male AP-treated/male unexposed  n=33/1068 | FGA: n=5; SGA: n=25; both: n= 3 | Exposed group: FEP, had not been prescribed antipsychotics in the previous 3 months. | AP-treated male group: 24.50 (4.40)  Male unexposed group: 25.40 (3.40) | Cholesterol  Natural setting | Exposed group: EPIC Questionnaire  Unexposed group: self-recorded food consumption for 3 consecutive days | Difference in mean cholesterol intake between male exposed and unexposed groups. | 450.00 | 241.00 | 331.00 | 157.00 | **119.00*** | 0.007 | NA (data were not normally distributed, non-parametric tests applied) |
| **CARBOHYDRATES** | | | | | | | | | | | | | | | | | |
| **HIGH** | (Blouin et al., 2008) | Cross-sectional | NA | SGA-treated group/Unexposed group  n= 18/20 | SGAs including clozapine: n = 2; olanzapine:  n = 9; risperidone: n = 2; quetiapine: n = 3; ziprasidone: n = 2. | Duration of current SGA treatment in months, mean (SD): 24.60 (19.70).  Duration of prior AP exposure (FGA or SGA) in SGA-treated group months, mean (SD): 35.30 (22.00).  SGA-treated group had to be sedentary (practicing <30 min of continuous physical activity per week).  Unexposed group matched by age and physical activity. | SGA-treated group:  28.80 (5.0)  Unexposed group:  25.0 (3.30) | Carbohydrate intake (g)  Lab-setting (ad libitum buffet-type meal) | Food was weighed before and after the buffet. Energy and nutrient intakes were calculated using the Canadian Nutrient File and/or information on food labels. | Difference in mean carbohydrate intake between SGA-treated group and unexposed group | 102.00 | 42.00 | 118.40 | 33.80 | **-16.40*** | 0.19* | (-41.37, 8.57)* |
| **HIGH** | (Blouin et al., 2008) | Cross-sectional | NA | SGA-treated group/Unexposed group  n= 18/20 | SGAs including clozapine: n = 2; olanzapine:  n = 9; risperidone: n = 2; quetiapine: n = 3; ziprasidone: n = 2. | Duration of current SGA treatment in months, mean (SD): 24.60 (19.70).  Duration of prior AP exposure (FGA or SGA) in SGA-treated group months, mean (SD): 35.30 (22.00).  SGA-treated group had to be sedentary (practicing <30 min of continuous physical activity per week).  Unexposed group matched by age and physical activity. | SGA-treated group:  28.80 (5.0)  Unexposed group:  25.0 (3.30) | Carbohydrate intake (% of meal)  Lab-setting (ad libitum buffet-type meal) | Food was weighed before and after the buffet, energy and nutrient intakes were calculated using the Canadian Nutrient File and/or information on food labels. | Difference in mean carbohydrate intake between SGA-treated group and unexposed group | 46.60 | 7.40 | 49.10 | 9.20 | **-2.50*** | 0.37* | (-8.03, 3.03)* |
|  | (Henderson et al., 2006) | Cross-sectional | NA | SGA-treated group/Unexposed group  n= 88/723 | Olanzapine: n= 42; clozapine: n=25; risperidone: n= 15; quetiapine: n=3; ziprasidone: n= 1; med-free: n=2 | Duration of treatment in SGA-treated group: NR | SGA-treated group: 31.30 (12.67)  Unexposed group: 28.30 (6.62) | Carbohydrate intake (g)  Natural setting | SGA-treated group: 4-day dietary record  Unexposed group: 24-hour recall method | Difference in mean carbohydrate intake between SGA-treated group and unexposed group | 236.70 | 104.60 | 323.70 | 179.70 | **-87.00*** | 0.00001 | (-125.38, -48.62)* |
|  | (Jakobsen et al., 2018) | Cross-sectional | NA | SGA-treated group/ Unexposed group  n=346/3016 | SGAs including olanzapine, clozapine, quetiapine | Duration of treatment in SGA-treated group: NR.  Use of other medications: NR | SGA-treated group: NR.  Having a waist circumference ≥88 cm for females and ≥102 cm for males was an inclusion criterion.  Unexposed group: NR | Carbohydrate intake (% of energy)  Natural setting | SGA-treated group: 24-hr recall  Unexposed group: food record kept for 7 consecutive days | Difference in mean carbohydrate intake between SGA-treated and unexposed groups | 48.70 | 11.50 | 47.00 | 6.10 | **1.70*** | 0.007 | (0.94, 2.46)* |
|  | (Nunes et al., 2014) | Cross-sectional | NA | AP-treated group/Unexposed group  n= 25/25 | FGA: n= 7; SGA: n= 17; used both FGA and SGA: n= 1. | Duration of AP treatment: NR.  Use of other medications: NR | AP-treated group: 29.09 (6.30)  Unexposed group: 26.91 (4.39) | Carbohydrate intake (% of energy)  Natural setting | Food frequency questionnaire | Difference in mean carbohydrate intake (% of energy) between AP-treated group and unexposed group. | 56.7 | 4.8 | 50.4 | 7.4 | **6.30*** | 0.001 | (2.75, 9.85)* |
|  | (Stefanska et al., 2017) | Cross-sectional | NA | Female AP-treated/ female unexposed:  n= 32/60 | FGAs, SGAs | Duration of treatment in AP-treated group: ≥ 1 year.  Use of other medications: NR | AP-treated female group: 27.2 (5.7),  Female unexposed group: 25.8 (5.2), | Carbohydrate intake (g/day)  Natural setting | 24-hr recall | Difference in mean carbohydrate intake between AP-treated and unexposed groups | 266.7 | 109.7 | 191.6 | 84.5 | **75.10*** | 0.0004* | (34.24, 115.96)* |
|  | (Stefanska et al., 2017) | Cross-sectional | NA | Female AP-treated/ female unexposed:  n= 32/60 | FGAs, SGAs | Duration of treatment in AP-treated group: ≥ 1 year.  Use of other medications: NR | AP-treated female group: 27.2 (5.7),  Female unexposed group: 25.8 (5.2), | Carbohydrate intake (% of energy)  Natural setting | 24-hr recall | Difference in mean carbohydrate intake (% of energy) between AP-treated and unexposed groups | 55.3 | 9.1 | 49.7 | 10.7 | **5.60*** | 0.01* | (2.23, 1.17)* |
|  | (Stefanska et al., 2017) | Cross-sectional | NA | Male AP-treated/male unexposed  n= 28/38 | FGAs, SGAs | Duration of treatment in AP-treated group: ≥ 1 year.  Use of other medications: NR | AP-treated male group: 27.6 (5.9)  Male unexposed group: 27.3 (5.7) | Carbohydrate intake (g/day)  Natural setting | 24-hr recall | Difference in mean carbohydrate intake between AP-treated and unexposed groups | 295.2 | 96.9 | 280.1 | 114.0 | **15.10*** | 0.57* | (-38.20, 68.40)* |
|  | (Stefanska et al., 2017) | Cross-sectional | NA | Male AP-treated/male unexposed  n= 28/38 | FGAs, SGAs | Duration of treatment in AP-treated group: ≥ 1 year.  Use of other medications: NR | AP-treated male group: 27.6 (5.9)  Male unexposed group: 27.3 (5.7) | Carbohydrate intake (% of energy)  Natural setting | 24-hr recall | Difference in mean carbohydrate intake (% of energy) between AP-treated and unexposed groups | 49.0 | 11.7 | 47.9 | 11.4 | **1.10*** | 0.70* | (-4.64, 6.84)* |
|  | (Stefanska et al., 2018) | Cross-sectional | NA | Female AP-treated/ female unexposed:  n=45/40 | FGAs, SGAs | Duration of treatment in AP-treated group: ≥ 1 year.  Use of other medications: NR | AP-treated female group: 25.1 (5.3)  Female unexposed group: 24.4 (5.0) | Carbohydrate intake (g/day)  Natural setting | 24-hr recall | Difference in mean carbohydrate intake between AP-treated and unexposed groups | 248.9 | 100.4 | 193.0 | 78.2 | **55.90*** | 0.006* | (16.72, 95.08)* |
|  | (Stefanska et al., 2018) | Cross-sectional | NA | Female AP-treated/ female unexposed:  n=45/40 | FGAs, SGAs | Duration of treatment in AP-treated group: ≥ 1 year.  Use of other medications: NR | AP-treated female group: 25.1 (5.3)  Female unexposed group: 24.4 (5.0) | Carbohydrate intake (% of energy)  Natural setting | 24-hr recall | Difference in mean carbohydrate intake (% of energy) between AP-treated and unexposed groups | 54.8 | 7.9 | 50.8 | 11.0 | **4.00*** | 0.06* | (-0.10, 8.10)* |
|  | (Stefanska et al., 2018) | Cross-sectional | NA | Male AP-treated/male unexposed  n=40/30 | FGAs, SGAs | Duration of treatment in AP-treated group: ≥ 1 year.  Use of other medications: NR | AP-treated male group: 25.0 (4.6)  Male unexposed group: 25.9 (4.2) | Carbohydrate intake (g/day)  Natural setting | 24-hr recall | Difference in mean carbohydrate intake between AP-treated and unexposed groups | 259.7 | 90.8 | 319.7 | 135.4 | **-60.00*** | 0.03* | (-113.99, -6.01)* |
|  | (Stefanska et al., 2018) | Cross-sectional | NA | Male AP-treated/male unexposed  n=40/30 | FGAs, SGAs | Duration of treatment in AP-treated group: ≥ 1 year.  Use of other medications: NR | AP-treated male group: 25.0 (4.6)  Male unexposed group: 25.9 (4.2) | Carbohydrate intake (% of energy)  Natural setting | 24-hr recall | Difference in mean carbohydrate intake (% of energy) between AP-treated and unexposed groups | 48.7 | 10.5 | 47.7 | 9.7 | **1.00*** | 0.69* | (-3.90, 5.90)* |
| **NORMAL** | (Saugo et al., 2020) | Cross-sectional analysis of data collected from subsample of GETUP study (prospective 9-month study) | NA | Female AP-treated/ female unexposed:  n=21/1245 | FGA: n=6; SGA: n=14; both: n=1 | Exposed group: FEP, had not been prescribed antipsychotics in the previous 3 months | AP-treated female group: 21.40 (2.10)  Female unexposed group: 23.20 (3.80). | Carbohydrates (g/day)  Natural setting | Exposed group: EPIC Questionnaire  Unexposed group: self-recorded food consumption for 3 consecutive days | Difference in mean carbohydrate intake between female exposed and unexposed groups. | 272.80 | 138.40 | 236.50 | 75.30 | **36.30*** | 0.24 3 | NA (data were not normally distributed, non-parametric tests applied) |
|  | (Saugo et al., 2020) | Cross-sectional analysis of data collected from subsample of GETUP study (prospective 9-month study) | NA | Male AP-treated/male unexposed  n=33/1068 | FGA: n=5; SGA: n=25; both: n= 3 | Exposed group: FEP, had not been prescribed antipsychotics in the previous 3 months | AP-treated male group: 24.50 (4.40)  Male unexposed group: 25.40 (3.40) | Carbohydrates (g/day)  Natural setting | Exposed group: EPIC Questionnaire  Unexposed group: self-recorded food consumption for 3 consecutive days | Difference in mean carbohydrate intake between male exposed and unexposed groups. | 291.50 | 141.30 | 283.10 | 88.70 | **8.40*** | 0.736 | NA (data were not normally distributed, non-parametric tests applied) |
| Sugar/soluble carbohydrates | | | | | | | | | | | | | | | | | |
| **HIGH** | (Jakobsen et al., 2018) | Cross-sectional | NA | AP-treated group/ Unexposed group  n=346/3016 | Olanzapine, clozapine, quetiapine | Duration of treatment in AP-treated group: NR.  Use of other medications: NR | AP-treated group: NR.  Having a waist circumference ≥88 cm for females and ≥102 cm for males was an inclusion criterion.  Unexposed group: NR | Sugar intake (% of energy)  Natural setting | Exposed group: 24-hr recall  Unexposed group: food record kept for 7 consecutive days | Difference in mean sugar intake between exposed and unexposed groups | 11.80 | 2.80 | 9.00 | 5.30 | **2.80*** | <0.001 | (2.23, 3.37)* |
|  | (Nunes et al., 2014) | Cross-sectional | NA | AP-treated group/Unexposed group  n= 25/25 | FGA: n= 7; SGA: n= 17; used both FGA and SGA: n= 1. | Duration of AP treatment: NR.  Use of other medications: NR | AP-treated group: 29.09 (6.30)  Unexposed group: 26.91 (4.39) | Sugar intake (% of energy)  Natural setting | Food frequency questionnaire | Difference in sugar intake (% of energy) between AP-treated group and unexposed group. | 19.2 | 5.0 | 18.3 | 5.7 | **0.70*** | 0.477 | (-2.14, 3.95)* |
|  | (Saugo et al., 2020) | Cross-sectional analysis of data collected from subsample of GETUP study (prospective 9-month study) | NA | Female AP-treated/ female unexposed:  n=21/1245 | FGA: n=6; SGA: n=14; both: n=1 | Exposed group: FEP, had not been prescribed antipsychotics in the previous 3 months | AP-treated female group: 21.40 (2.10)  Female unexposed group: 23.20 (3.80). | Soluble carbohydrates (g/day)  Natural setting | Exposed group: EPIC Questionnaire  Unexposed group: self-recorded food consumption for 3 consecutive days | Difference in mean soluble carbohydrate intake between female exposed and unexposed groups | 142.60 | 89.00 | 79.50 | 33.40 | **63.10*** | 0.004 | NA (data were not normally distributed, non-parametric tests applied) |
|  | (Saugo et al., 2020) | Cross-sectional analysis of data collected from subsample of GETUP study (prospective 9-month study) | NA | Male AP-treated/male unexposed  n=33/1068 | FGA: n=5; SGA: n=25; both: n= 3 | Exposed group: FEP, had not been prescribed antipsychotics in the previous 3 months | AP-treated male group: 24.50 (4.40)  Male unexposed group: 25.40 (3.40) | Soluble carbohydrates (g/day)  Natural setting | Exposed group: EPIC Questionnaire  Unexposed group: self-recorded food consumption for 3 consecutive days | Difference in mean soluble carbohydrate intake between male exposed and unexposed groups | 138.50 | 84.40 | 86.00 | 37.70 | **52.50*** | 0.001 | NA (data were not normally distributed, non-parametric tests applied) |
| Starch | | | | | | | | | | | | | | | | | |
| **NORMAL** | (Saugo et al., 2020) | Cross-sectional analysis of data collected from subsample of GETUP study (prospective 9-month study) | NA | Female AP-treated/ female unexposed:  n=21/1245 | FGA: n=6; SGA: n=14; both: n=1 | Exposed group: FEP, had not been prescribed antipsychotics in the previous 3 months | AP-treated female group: 21.40 (2.10)  Female unexposed group: 23.20 (3.80). | Starch (g/day)  Natural setting | Exposed group: EPIC Questionnaire  Unexposed group: self-recorded food consumption for 3 consecutive days | Difference in mean starch intake between female exposed and unexposed groups. | 129.50 | 75.90 | 141.80 | 51.50 | **-12.30*** | 0.465 | NA (data were not normally distributed, non-parametric tests applied) |
|  | (Saugo et al., 2020) | Cross-sectional analysis of data collected from subsample of GETUP study (prospective 9-month study) | NA | Male AP-treated/male unexposed  n=33/1068 | FGA: n=5; SGA: n=25; both: n= 3 | Exposed group: FEP, had not been prescribed antipsychotics in the previous 3 months | AP-treated male group: 24.50 (4.40)  Male unexposed group: 25.40 (3.40) | Starch (g/day)  Natural setting | Exposed group: EPIC Questionnaire  Unexposed group: self-recorded food consumption for 3 consecutive days | Difference in mean starch intake between male exposed and unexposed groups. | 152.60 | 78.20 | 178.60 | 62.30 | **-26.00*** | 0.064 | NA (data were not normally distributed, non-parametric tests applied) |
| **PROTEIN** | | | | | | | | | | | | | | | | | |
| **HIGH** | (Blouin et al., 2008) | Cross-sectional | NA | SGA-treated group/Unexposed group  n= 18/20 | SGAs including clozapine: n = 2; olanzapine:  n = 9; risperidone: n = 2; quetiapine: n = 3; ziprasidone: n = 2. | Duration of current SGA treatment in months, mean (SD): 24.60 (19.70).  Duration of prior AP exposure (FGA or SGA) in SGA-treated group months, mean (SD): 35.30 (22.00).  SGA-treated group had to be sedentary (practicing <30 min of continuous physical activity per week).  Unexposed group matched by age and physical activity. | SGA-treated group:  28.80 (5.0)  Unexposed group:  25.0 (3.30) | Protein intake (g)  Lab-setting (ad libitum buffet-type meal) | food was weighed before and after the buffet, energy and nutrient intakes were calculated using the Canadian Nutrient File and/or information on food labels. | Difference in mean protein intake between SGA-treated group and unexposed group | 40.30 | 16.30 | 42.70 | 15.20 | **-2.40*** | 0.64* | (-12.76, 7.96)* |
|  | (Blouin et al., 2008) | Cross-sectional | NA | SGA-treated group/Unexposed group  n= 18/20 | SGAs including clozapine: n = 2; olanzapine:  n = 9; risperidone: n = 2; quetiapine: n = 3; ziprasidone: n = 2. | Duration of current SGA treatment in months, mean (SD): 24.60 (19.70).  Duration of prior AP exposure (FGA or SGA) in SGA-treated group months, mean (SD): 35.30 (22.00).  SGA-treated group had to be sedentary (practicing <30 min of continuous physical activity per week).  Unexposed group matched by age and physical activity. | SGA-treated group:  28.80 (5.0)  Unexposed group:  25.0 (3.30) | Protein intake (% of meal)  Lab-setting (ad libitum buffet-type meal) | Food was weighed before and after the buffet. Energy and nutrient intakes were calculated using the Canadian Nutrient File and/or information on food labels. | Difference in mean protein intake between SGA-treated group and unexposed group | 20.10 | 6.60 | 17.50 | 3.60 | **2.60*** | 0.15* | (-0.85, 6.05)* |
|  | (Henderson et al., 2006) | Cross-sectional | NA | SGA-treated group/Unexposed group  n= 88/723 | Olanzapine: n= 42; clozapine: n=25; risperidone: n= 15; quetiapine: n=3; ziprasidone: n= 1; med-free: n=2 | Duration of treatment in SGA-treated group: NR | SGA-treated group: 31.30 (12.67)  Unexposed group: 28.30 (6.62) | Protein intake (g)  Natural setting | SGA-treated group: 4-day dietary record  Unexposed group: 24-hour recall | Difference in mean protein intake between SGA-treated group and unexposed group | 72.90 | 38.00 | 95.00 | 54.50 | **-22.10** | 0.00001 | (-31.06, -13.14)* |
|  | (Jakobsen et al., 2018) | Cross-sectional | NA | AP-treated group/ Unexposed group  n=346/3016 | Olanzapine, clozapine, quetiapine | Duration of treatment in AP-treated group: NR.  Use of other medications: NR | AP-treated group: NR.  Having a waist circumference ≥88 cm for females and ≥102 cm for males was an inclusion criterion.  Unexposed group: NR | Protein intake (% of energy)  Natural setting | Exposed group: 24-hr recall  Unexposed group: food record kept for 7 consecutive days | Difference in mean protein intake between exposed and unexposed groups | 16.00 | 5.00 | 16.00 | 2.80 | **0.00*** | 0.99 | (-0.34, 0.34)* |
|  | (Nunes et al., 2014) | Cross-sectional | NA | AP-treated group/Unexposed group  n= 25/25 | FGA: n= 7; SGA: n= 17; used both FGA and SGA: n= 1. | Duration of AP treatment: NR.  Use of other medications: NR | AP-treated group: 29.09 (6.30)  Unexposed group: 26.91 (4.39) | Protein intake (% of energy)  Natural setting | Food frequency questionnaire | Difference in protein intake (% of energy) between AP-treated group and unexposed group. | 18.3 | 2.8 | 17.2 | 3.9 | **1.10*** | 0.274 | (-0.83, 3.03)* |
|  | (Stefanska et al., 2017) | Cross-sectional | NA | Female AP-treated/ female unexposed:  n= 32/60 | FGAs, SGAs | Duration of treatment in AP-treated group: ≥ 1 year.  Use of other medications: NR | AP-treated female group: 27.2 (5.7),  Female unexposed group: 25.8 (5.2), | Protein intake (g/day)  Natural setting | 24-hr recall | Difference in mean protein intake between AP-treated and unexposed groups | 63.6 | 16.8 | 62.4 | 25.1 | **1.20*** | 0.81* | (-8.62, 11.02)* |
|  | (Stefanska et al., 2017) | Cross-sectional | NA | Female AP-treated/ female unexposed:  n= 32/60 | FGAs, SGAs | Duration of treatment in AP-treated group: ≥ 1 year.  Use of other medications: NR | AP-treated female group: 27.2 (5.7),  Female unexposed group: 25.8 (5.2), | Protein intake (% of energy)  Natural setting | 24-hr recall | Difference in mean protein intake (% of energy) between AP-treated and unexposed groups | 13.7 | 3.0 | 18.3 | 5.4 | **-4.60*** | <0.0001* | (-6.65, -2.55)* |
|  | (Stefanska et al., 2017) | Cross-sectional | NA | Male AP-treated/male unexposed  n= 28/38 | FGAs, SGAs | Duration of treatment in AP-treated group: ≥ 1 year.  Use of other medications: NR | AP-treated male group: 27.6 (5.9)  Male unexposed group: 27.3 (5.7) | Protein intake (g/day)  Natural setting | 24-hr recall | Difference in mean protein intake between AP-treated and unexposed groups | 76.7 | 22.3 | 103.3 | 38.2 | **-26.60*** | 0.002* | (-42.75, -10.45)* |
|  | (Stefanska et al., 2017) | Cross-sectional | NA | Male AP-treated/male unexposed  n= 28/38 | FGAs, SGAs | Duration of treatment in AP-treated group: ≥ 1 year.  Use of other medications: NR | AP-treated male group: 27.6 (5.9)  Male unexposed group: 27.3 (5.7) | Protein intake (% of energy)  Natural setting | 24-hr recall | Difference in mean protein intake (% of energy) between AP-treated and unexposed groups | 13.5 | 3.2 | 19.7 | 5.8 | **-6.20*** | <0.0001* | (-8.63, -3.77)* |
|  | (Stefanska et al., 2018) | Cross-sectional | NA | Female AP-treated/ female unexposed:  n=45/40 | FGAs, SGAs | Duration of treatment in AP-treated group: ≥ 1 year.  Use of other medications: NR | AP-treated female group: 25.1 (5.3)  Female unexposed group: 24.4 (5.0) | Protein intake (g/day)  Natural setting | 24-hr recall | Difference in mean total protein intake between AP-treated and unexposed groups | 63.4 | 16.6 | 65.7 | 20.4 | **-2.30*** | 0.57* | (-10.29, 5.69)* |
|  | (Stefanska et al., 2018) | Cross-sectional | NA | Female AP-treated/ female unexposed:  n=45/40 | FGAs, SGAs | Duration of treatment in AP-treated group: ≥ 1 year.  Use of other medications: NR | AP-treated female group: 25.1 (5.3)  Female unexposed group: 24.4 (5.0) | Protein intake (% of energy)  Natural setting | 24-hr recall | Difference in mean total protein intake (% of energy) between AP-treated and unexposed groups | 14.3 | 2.3 | 19.4 | 5.4 | **-5.10*** | <0.0001 | (-6.86, -3.34)* |
|  | (Stefanska et al., 2018) | Cross-sectional | NA | Male AP-treated/male unexposed  n=40/30 | FGAs, SGAs | Duration of treatment in AP-treated group: ≥ 1 year.  Use of other medications: NR | AP-treated male group: 25.0 (4.6)  Male unexposed group: 25.9 (4.2) | Protein intake (g/day)  Natural setting | 24-hr recall | Difference in mean total protein intake between AP-treated and unexposed groups | 72.7 | 20.0 | 111.7 | 30.4 | **-39.00*** | <0.0001 | (-51.03, -26.97)* |
|  | (Stefanska et al., 2018) | Cross-sectional | NA | Male AP-treated/male unexposed  n=40/30 | FGAs, SGAs | Duration of treatment in AP-treated group: ≥ 1 year.  Use of other medications: NR | AP-treated male group: 25.0 (4.6)  Male unexposed group: 25.9 (4.2) | Protein intake (% of energy)  Natural setting | 24-hr recall | Difference in mean total protein intake (% of energy) between AP-treated and unexposed groups | 14.6 | 3.0 | 18.8 | 4.9 | **-4.20*** | <0.0001* | (-6.09, -2.31)* |
| **NORMAL** | (Saugo et al., 2020) | Cross-sectional analysis of data collected from subsample of GETUP study (prospective 9-month study) | NA | Female AP-treated/ female unexposed:  n=21/1245 | FGA: n=6; SGA: n=14; both: n=1 | Exposed group: FEP, had not been prescribed antipsychotics in the previous 3 months | AP-treated female group: 21.40 (2.10)  Female unexposed group: 23.20 (3.80). | Proteins (g/day)  Natural setting | Exposed group: EPIC Questionnaire  Unexposed group: self-recorded food consumption for 3 consecutive days | Difference in mean protein intake between female exposed and unexposed groups. | 78.30 | 35.40 | 76.00 | 19.50 | **2.30*** | 0.766 | NA (data were not normally distributed, non-parametric tests applied) |
|  | (Saugo et al., 2020) | Cross-sectional analysis of data collected from subsample of GETUP study (prospective 9-month study) | NA | Male AP-treated/male unexposed  n=33/1068 | FGA: n=5; SGA: n=25; both: n= 3 | Exposed group: FEP, had not been prescribed antipsychotics in the previous 3 months | AP-treated male group: 24.50 (4.40)  Male unexposed group: 25.40 (3.40) | Proteins (g/day)  Natural setting | Exposed group: EPIC Questionnaire  Unexposed group: self-recorded food consumption for 3 consecutive days | Difference in mean protein intake between male exposed and unexposed groups | 98.90 | 40.40 | 92.60 | 25.30 | **6.30*** | 0.38 | NA (data were not normally distributed, non-parametric tests applied) |
| **FIBRE INTAKE** | | | | | | | | | | | | | | | | | |
| **HIGH** | (Henderson et al., 2006) | Cross-sectional | NA | SGA-treated group/Unexposed group  n= 88/723 | Olanzapine: n= 42; clozapine: n=25; risperidone: n= 15; quetiapine: n=3; ziprasidone: n= 1; med-free: n=2 | Duration of treatment in SGA-treated group: NR | SGA-treated group: 31.30 (12.67)  Unexposed group: 28.30 (6.62) | Fibre intake (g)  Natural setting | SGA-treated group: 4-day dietary record  Unexposed group: 24-hour recall method (controls) | Difference in mean fibre intake between SGA-treated group and unexposed group | 13.60 | 6.30 | 16.90 | 11.90 | **-3.30** | 0.01* | (-5.83, -0.77)* |
|  | (Jakobsen et al., 2018) | Cross-sectional | NA | AP-treated group/ Unexposed group  n=346/3016 | Olanzapine, clozapine, quetiapine | Duration of treatment in AP-treated group: NR.  Use of other medications: NR | AP-treated group: NR.  Having a waist circumference ≥88 cm for females and ≥102 cm for males was an inclusion criterion.  Unexposed group: NR | Fibre intake (g)  Natural setting | Exposed group: 24-hr recall  Unexposed group: food record kept for 7 consecutive days | Difference in mean fibre intake between exposed and unexposed groups | 18.30 | 13.50 | 22.00 | 7.90 | **-3.70*** | <0.001 | (-4.66, -2.74)* |
|  | (Nunes et al., 2014) | Cross-sectional | NA | AP-treated group/Unexposed group  n= 25/25 | FGA: n= 7; SGA: n= 17; used both FGA and SGA: n= 1. | Duration of AP treatment: NR.  Use of other medications: NR | AP-treated group: 29.09 (6.30)  Unexposed group: 26.91 (4.39) | Fibre intake g/1000 kcal)  Natural setting | Food frequency questionnaire | Difference in fibre intake (g/1000 kcal) between AP-treated group and unexposed group. | 12.3 | 2.9 | 12.5 | 3.2 | **-0.20*** | 0.864 | (-1.94, 1.54) |
|  | (Stefanska et al., 2017) | Cross-sectional | NA | Female AP-treated/ female unexposed:  n= 32/60 | FGAs, SGAs | Duration of treatment in AP-treated group: ≥ 1 year.  Use of other medications: NR | AP-treated female group: 27.2 (5.7),  Female unexposed group: 25.8 (5.2), | Fibre intake (g/day)  Natural setting | 24-hr recall | Difference in mean fibre intake between AP-treated and unexposed groups | 20.6 | 8.8 | 19.3 | 8.3 | **1.30*** | 0.49* | (-2.39, 4.99)* |
|  | (Stefanska et al., 2017) | Cross-sectional | NA | Male AP-treated/male unexposed  n= 28/38 | FGAs, SGAs | Duration of treatment in AP-treated group: ≥ 1 year.  Use of other medications: NR | AP-treated male group: 27.6 (5.9)  Male unexposed group: 27.3 (5.7) | Fibre intake (g/day)  Natural setting | 24-hr recall | Difference in mean fibre intake between AP-treated and unexposed groups | 17.3 | 5.2 | 20.7 | 10.6 | **-3.40*** | 0.12* | (-7.75, 0.95)* |
|  | (Stefanska et al., 2018) | Cross-sectional | NA | Female AP-treated/ female unexposed:  n=45/40 | FGAs, SGAs | Duration of treatment in AP-treated group: ≥ 1 year.  Use of other medications: NR | AP-treated female group: 25.1 (5.3)  Female unexposed group: 24.4 (5.0) | Fibre intake (g/day)  Natural setting | 24-hr recall | Difference in mean fibre intake between AP-treated and unexposed groups | 18.2 | 7.0 | 19.9 | 7.9 | **-1.70*** | 0.30* | (-4.91, 1.51)* |
|  | (Stefanska et al., 2018) | Cross-sectional | NA | Male AP-treated/male unexposed  n=40/30 | FGAs, SGAs | Duration of treatment in AP-treated group: ≥ 1 year.  Use of other medications: NR | AP-treated male group: 25.0 (4.6)  Male unexposed group: 25.9 (4.2) | Fibre intake (g/day)  Natural setting | 24-hr recall | Difference in mean fibre intake between AP-treated and unexposed groups | 15.9 | 5.0 | 23.3 | 9.7 | **-7.40*** | 0.0001* | (-10.96, -3.84)* |
| **NORMAL** | (Saugo et al., 2020) | Cross-sectional analysis of data collected from subsample of GETUP study (prospective 9-month study) | NA | Female AP-treated/ female unexposed:  n=21/1245 | FGA: n=6; SGA: n=14; both: n=1 | Exposed group: FEP, had not been prescribed antipsychotics in the previous 3 months | AP-treated female group: 21.40 (2.10)  Female unexposed group: 23.20 (3.80). | Dietary fibre (g/day)  Natural setting | Exposed group: EPIC Questionnaire  Unexposed group: self-recorded food consumption for 3 consecutive days | Difference in mean fibre intake between female exposed and unexposed groups | 18.70 | 10.10 | 17.70 | 6.30 | **1.00*** | 0.657 | NA (data were not normally distributed, non-parametric tests applied) |
|  | (Saugo et al., 2020) | Cross-sectional analysis of data collected from subsample of GETUP study (prospective 9-month study) | NA | Male AP-treated/male unexposed  n=33/1068 | FGA: n=5; SGA: n=25; both: n= 3 | Exposed group: FEP, had not been prescribed antipsychotics in the previous 3 months | AP-treated male group: 24.50 (4.40)  Male unexposed group: 25.40 (3.40) | Dietary fibre (g/day)  Natural setting | Exposed group: EPIC Questionnaire  Unexposed group: self-recorded food consumption for 3 consecutive days | Difference in mean fibre intake between male exposed and unexposed groups | 16.20 | 7.80 | 19.60 | 7.30 | **-3.40*** | 0.017 | NA (data were not normally distributed, non-parametric tests applied) |
| **ALCOHOL** | | | | | | | | | | | | | | | | | |
| **HIGH** | (Henderson et al., 2006) | Cross-sectional | NA | SGA-treated group/Unexposed group  n= 88/723 | Olanzapine: n= 42; clozapine: n=25; risperidone: n= 15; quetiapine: n=3; ziprasidone: n= 1; med-free: n=2 | Duration of treatment in SGA-treated group: NR | SGA-treated group: 31.30 (12.67)  Unexposed group: 28.30 (6.62) | Alcohol intake (g)  Natural setting | SGA-treated group: 4-day dietary record  Unexposed group: 24-hour recall | Difference in mean alcohol intake between SGA-treated group and unexposed group | 1.80 | 5.10 | 17.20 | 62.90 | **-15.40*** | 0.02 | (-28.57, -2.23)* |
|  | (Jakobsen et al., 2018) | Cross-sectional | NA | AP-treated group/ Unexposed group  n=346/3016 | Olanzapine, clozapine, quetiapine | Duration of treatment in AP-treated group: NR.  Use of other medications: NR | AP-treated group: NR.  Having a waist circumference ≥88 cm for females and ≥102 cm for males was an inclusion criterion.  Unexposed group: NR | Alcohol intake (g/week)  Natural setting | Exposed group: 24-hr recall  Unexposed group: food record kept for 7 consecutive days | Difference in mean alcohol intake between exposed and unexposed groups | 27.70 | 108.40 | 15.50 | 18.20 | **12.20*** | 0.037 | (0.72, 23.68)* |
|  | (Nunes et al., 2014) | Cross-sectional | NA | AP-treated group/Unexposed group  n= 25/25 | FGA: n= 7; SGA: n= 17; used both FGA and SGA: n= 1. | Duration of AP treatment: NR.  Use of other medications: NR | AP-treated group: 29.09 (6.30)  Unexposed group: 26.91 (4.39) | Alcohol intake (% of energy)  Natural setting | Food frequency questionnaire | Difference in alcohol intake (% of energy) between AP-treated group and unexposed group. | 0 (median) | NA | 1.42  (median) | NA | **1.42*** | NA | NA |
| **NORMAL** | (Saugo et al., 2020) | Cross-sectional analysis of data collected from subsample of GETUP study (prospective 9-month study) | NA | Female AP-treated/ female unexposed:  n=21/1245 | FGA: n=6; SGA: n=14; both: n=1 | Exposed group: FEP, had not been prescribed antipsychotics in the previous 3 months | AP-treated female group: 21.40 (2.10)  Female unexposed group: 23.20 (3.80). | Alcohol (g/day)  Natural setting | Exposed group: EPIC Questionnaire  Unexposed group: self-recorded food consumption for 3 consecutive days | Difference in mean alcohol intake between female exposed and unexposed groups | 6.80 | 11.80 | 5.00 | 8.30 | **1.80*** | 0.063 | NA (data were not normally distributed, non-parametric tests applied) |
|  | (Saugo et al., 2020) | Cross-sectional analysis of data collected from subsample of GETUP study (prospective 9-month study) | NA | Male AP-treated/male unexposed  n=33/1068 | FGA: n=5; SGA: n=25; both: n= 3 | Exposed group: FEP, had not been prescribed antipsychotics in the previous 3 months | AP-treated male group: 24.50 (4.40)  Male unexposed group: 25.40 (3.40) | Alcohol (g/day)  Natural setting | Exposed group: EPIC Questionnaire  Unexposed group: self-recorded food consumption for 3 consecutive days. | Difference in mean alcohol intake between male exposed and unexposed groups | 5.80 | 10.90 | 13.90 | 17.00 | **-8.10*** | 0.358 | NA (data were not normally distributed, non-parametric tests applied) |
| **QUALITY OF DIET** | | | | | | | | | | | | | | | | | |
| **HIGH** | (Jakobsen et al., 2018) | Cross-sectional | NA | AP-treated group/ Unexposed group  n=346/3016 | Olanzapine, clozapine, quetiapine | Duration of treatment in AP-treated group: NR.  Use of other medications: NR | AP-treated group: NR.  Having a waist circumference ≥88 cm for females and ≥102 cm for males was an inclusion criterion.  Unexposed group: NR | Healthy diet  Natural setting | Measured using the DQS in the FFQ | OR of consuming unhealthy diet (% with DQS S ≤ 6) in exposed group compared to unexposed group | NR | NR | NR | NR | NR | NR | OR of consuming unhealthy diet in exposed group compared to unexposed group= 4.89, p<0.0001 * |

Mean difference (or difference in means) in between-group analyses= AP-treated group mean – unexposed group mean

a= unvalidated measurement scale; b= collective scale (i.e., measure appetite and food craving or eating cognitions); *= calculated/converted from data reported in record.

%= percentage; adj= adjusted; AP= antipsychotic; BMI= body mass index; CI= confidence interval; FFQ= food frequency questionnaire; FGA= first-generation antipsychotic; g= gram; hr= hour; kcal= kilocalories; M= mean; MJ= megajoules; n= number; NA= not applicable; NR= not reported; OR= odds ratio; SD= standard deviation; SGA= second-generation antipsychotic

## Between-group syntheses - Summary of effect measures of antipsychotics on the odds of developing eating disorders

| BMI | Citation | Study design | End of study assessment | Sample size | Antipsychotic | Duration of prior AP treatment/other medications | Baseline BMI, M (SD) | Outcome of interest | Measurement scale | Odds of eating disorder in AP-treated group (exposed) | Odds of eating disorder in unexposed group | **OR** | LLCI | ULCI | p-value |
| --- | --- | --- | --- | --- | --- | --- | --- | --- | --- | --- | --- | --- | --- | --- | --- |
| **BINGE SYMTPOMS** | | | | | | | | | | | | | | | |
| **HIGH** | (Khazaal et al., 2006a) | Cross-sectional | NA | SGA-treated/unexposed, among those with BMI ≥28  n= 20/20 | SGAs including olanzapine, clozapine, quetiapine, risperidone. | All patients in the SGA-treated group (n=40):  Duration of treatment with SGA in years, mean (SD): 8.30 (6.20) | SGA-treated: 32.9 (6.1).  Unexposed: 33.8 (4.9). | Binge symptoms (binge episodes <2 days/week) | Binge eating status assessed as per DSM-IV criteria, classified into 4 categories:  (1) no binge eating  (2) binge episodes < 2 days/week (BS),  (3) BED or  (4) BN. | 5/15 | 4/16 | **1.33*** | 0.23* | 8.04* | 1* |
| **NORMAL** | (Khazaal et al., 2006a) | Cross-sectional | NA | SGA-treated/unexposed, among those with BMI < 28  n= 20/20 | SGAs including olanzapine, clozapine, quetiapine, risperidone. | All patients in the SGA-treated group (n=40):  Duration of treatment with SGA in years, mean (SD): 8.30 (6.20) | SGA-treated: 23.6 (2.2).  Unexposed: 21.1 (2.5). | Binge symptoms (binge episodes <2 days/week) | Binge eating status assessed as per DSM-IV criteria, classified into 4 categories:  (1) no binge eating  (2) binge episodes < 2 days/week (BS),  (3) BED or  (4) BN. | 3/17 | 4/16 | **0.71*** | 0.09* | 4.95* | 1* |
| **DISORDERED EATING** | | | | | | | | | | | | | | | |
| **HIGH** | (Khazaal et al., 2006a) | Cross-sectional | NA | SGA-treated/unexposed, among those with BMI ≥28  n= 20/20 | SGAs including olanzapine, clozapine, quetiapine, risperidone. | All patients in the SGA-treated group (n=40):  Duration of treatment with SGA in years, mean (SD): 8.30 (6.20) | SGA-treated group:  32.9 (6.1).  Unexposed group: 33.8 (4.9). p= 0.61 | BED | Binge eating status assessed as per DSM-IV criteria, classified into 4 categories:  (1) no binge eating  (2) binge episodes < 2 days/week (BS),  (3) BED or  (4) BN. | 7/13 | 2/18 | **4.85*** | 0.73* | 53.12* | 0.13* |
| **NORMAL** | (Khazaal et al., 2006a) | Cross-sectional | NA | SGA-treated/unexposed, among those with BMI < 28  n= 20/20 | SGAs including olanzapine, clozapine, quetiapine, risperidone. | All patients in the SGA-treated group (n=40):  Duration of treatment with SGA in years, mean (SD): 8.30 (6.20) | SGA-treated: 23.6 (2.2).  Unexposed: 21.1 (2.5). | BED | Binge eating status assessed as per DSM-IV criteria, classified into 4 categories:  (1) no binge eating  (2) binge episodes < 2 days/week (BS),  (3) BED or  (4) BN. | 2/18 | 0/20 | **Positive**  (OR cannot be calculated in the presence of a ‘zero’ cell) | NA | NA | NA |
| **NOT REPORTED** | (Khosravi, 2020) | Cross-sectional | NA | AP-treated/Unexposed  n= 154/154 | FGA, SGA | Duration of treatment: not reported (use of antidepressants or mood stabilizers in the previous 3 months was an exclusion criterion) | Not reported. | Disordered eating behaviours | Defined as a total score of ≥20 on the EAT-26 | 64/90 | 16/138 | **6.13*** | 3.24* | 12.04* | <0.00001* |

%= percentage; AP= antipsychotic; BED= binge eating disorder; BMI= body mass index; BN= bulimia nervosa; CI= confidence interval; DSM-IV: Diagnostic and Statistical Manual of Mental Disorders, 4th Edition; FGA= first-generation antipsychotic; LLCI= lower level of the 95% confidence interval; M= mean; n= number; NA= not applicable; NR= not reported; SD= standard deviation; SGA= second-generation antipsychotic; OR= odds ratio; ULCI= upper level of the 95% confidence interval; EAT-26: Eating Attitude Test.

## Between-group syntheses - Summary of effect measures of antipsychotics on eating cognitions and behaviours

| BMI | Citation | Study design | End of study assessment | Sample size | Antipsychotic | Duration of AP treatment/ other medications | Baseline BMI, M (SD) | Outcome of interest | Measurement scale | Analysis | AP-treated group mean | AP-treated group SD | Unexposed group mean | Unexposed group SD | **Mean difference** | p-value | OR [p, 95% CI] /or 95% CI for between-group mean differences differences |
| --- | --- | --- | --- | --- | --- | --- | --- | --- | --- | --- | --- | --- | --- | --- | --- | --- | --- |
| **OVERALL SCORE** | | | | | | | | | | | | | | | | |  |
| **High** | (Khazaal et al., 2006b) | Cross-sectional | NA | SGA-treated/unexposed, among those with BMI ≥28  n= 20/20 | SGA including olanzapine, clozapine, quetiapine, risperidone | All patients in the SGA-treated group: Duration of treatment with SGA in years, mean (SD): 8.3 (6.2) | SGA-treated: 32.90 (6.10)  Unexposed group: 33.80 (4.90) | Cognitive distortions | MAC-R total score | Difference in mean total MAC-R scores between cases and controls | 72.50 | 14.40 | 74.10 | 11.50 | **-1.60*** | 0.70* | (-9.94, 6.74)* |
|  | (Khazaal et al., 2009) | Cross-sectional | NA | SGA-treated/unexposed, among those with weight gain (minimum 2 kg in the last month)  n= 10/5 | All patients in the SGA-treated group: olanzapine, n=14; clozapine, n=2; risperidone, n=2; quetiapine, n=4 | Duration of treatment in SGA-treated group: NR | SGA-treated with weight gain: 28.60 (4.20) Unexposed with weight gain: 29.20 (4.30) | Eating cognitions | TFEQ total score | Difference in mean total TFEQ scores between cases and controls | 21.40 | 6.00 | 17.40 | 6.30 | **4.00*** | 0.25* | (-3.21, 11.21)* |
| **Normal** | (Khazaal et al., 2006b) | Cross-sectional | NA | SGA-treated group/unexposed group, among those with BMI <28  n= 20/20 | SGA including olanzapine, clozapine, quetiapine, risperidone | All patients in the SGA-treated group:  Duration of prior treatment with SGA in years, mean (SD): 8.3 (6.2) | SGA-treated: 23.60 (2.20)  Unexposed group: 21.10 (2.50) | Cognitive distortions | MAC-R total score | Difference in mean total MAC-R scores between cases and controls | 66.20 | 14.70 | 53.40 | 13.80 | **12.80*** | 0.007* | (3.67, 21.93)* |
|  | (Khazaal et al., 2009) | Cross-sectional | NA | SGA-treated/unexposed, among those without weight gain (neither gained nor lost more than 1 kg in the last month)  n= 12/10 | All patients in the SGA-treated group: olanzapine, n=14; clozapine, n=2; risperidone, n=2; quetiapine, n=4. | Duration of treatment in SGA-treated group: NR | SGA-treated without weight gain: 24.00 (5.60) Unexposed without weight gain: 22.50 (1.70) | Eating cognitions | TFEQ total score | Difference in mean total TFEQ scores between cases and controls | 19.80 | 9.40 | 12.20 | 3.90 | **7.60*** | 0.03* | (0.95, 14.25)* |
| **DIETARY RESTRIANT** | | | | | | | | | | | | | | | | |  |
| **HIGH** | (Blouin et al., 2008) | Cross-sectional | NA | SGA-treated group/Unexposed group  n= 18/20 | SGAs including clozapine: n = 2; olanzapine:  n = 9; risperidone: n = 2; quetiapine: n = 3; ziprasidone: n = 2. | Duration of current SGA treatment in months, mean (SD): 24.60 (19.70).  Duration of prior AP exposure (FGA or SGA) in SGA-treated group months, mean (SD): 35.30 (22.00).  SGA-treated group had to be sedentary (practicing <30 min of continuous physical activity per week).  Unexposed group matched by age and physical activity. | SGA-treated group:  28.80 (5.0)  Unexposed group:  25.0 (3.30)  *(Analysis adjusted for baseline BMI)* | Cognitive dietary restraint | TFEQ subscale 1 | Difference in mean TFQ subscale 1 scores between SGA-treated/unexposed | 7.10 | 4.00 | 4.70 | 3.10 | **2.40*** | 0.05 (adj for BMI) | NA |
|  | *(Blouin et al., 2008)* | *Cross-sectional* | *NA* | *SGA-treated group/Unexposed group*  *n= 18/20* | *SGAs including clozapine: n = 2; olanzapine:*  *n = 9; risperidone: n = 2; quetiapine: n = 3; ziprasidone: n = 2.* | *Duration of current SGA treatment in months, mean (SD): 24.60 (19.70).*  *Duration of prior AP exposure (FGA or SGA) in SGA-treated group months, mean (SD): 35.30 (22.00).*  *SGA-treated group had to be sedentary (practicing <30 min of continuous physical activity per week).*  *Unexposed group matched by age and physical activity.* | *SGA-treated group:*  *28.80 (5.0)*  *Unexposed group:*  *25.0 (3.30)*  *(Analysis adjusted for baseline BMI)* | *Cognitive dietary restraint subscale: Strategic dieting behaviour* | *TFEQ subscale 1* | *Difference in mean TFQ subscale 1 (strategic dieting behaviour) scores between SGA-treated and unexposed groups* | *1.10* | *1.20* | *0.40* | *0.90* | ***0.70**** | *0.05 (adj for BMI)* | *NA* |
|  | *(Blouin et al., 2008)* | *Cross-sectional* | *NA* | *SGA-treated group/Unexposed group*  *n= 18/20* | *SGAs including clozapine: n = 2; olanzapine:*  *n = 9; risperidone: n = 2; quetiapine: n = 3; ziprasidone: n = 2.* | *Duration of current SGA treatment in months, mean (SD): 24.60 (19.70).*  *Duration of prior AP exposure (FGA or SGA) in SGA-treated group months, mean (SD): 35.30 (22.00).*  *SGA-treated group had to be sedentary (practicing <30 min of continuous physical activity per week).*  *Unexposed group matched by age and physical activity.* | *SGA-treated group:*  *28.80 (5.0)*  *Unexposed group:*  *25.0 (3.30)* | *Cognitive dietary restraint subscale: Attitude to self-regulation* | *TFEQ subscale 1* | *Difference in mean TFQ subscale 1 (attitude to self-regulation) scores between SGA-treated and unexposed groups* | *1.40* | *0.90* | *1.30* | *0.90* | ***0.10**** | *0.73* (unadj because was not reported in paper)* | *(-0.49, 0.69)** |
|  | *(Blouin et al., 2008)* | *Cross-sectional* | *NA* | *SGA-treated group/Unexposed group*  *n= 18/20* | *SGAs including clozapine: n = 2; olanzapine:*  *n = 9; risperidone: n = 2; quetiapine: n = 3; ziprasidone: n = 2.* | *Duration of current SGA treatment in months, mean (SD): 24.60 (19.70).*  *Duration of prior AP exposure (FGA or SGA) in SGA-treated group months, mean (SD): 35.30 (22.00).*  *SGA-treated group had to be sedentary (practicing <30 min of continuous physical activity per week).*  *Unexposed group matched by age and physical activity.* | *SGA-treated group:*  *28.80 (5.0)*  *Unexposed group:*  *25.0 (3.30)*  *(Analysis adjusted for baseline BMI)* | *Cognitive dietary restraint subscale: Avoidance of fattening food* | *TFEQ subscale 1* | *Difference in mean TFQ subscale 1 (avoidance of fattening food) scores between SGA-treated and unexposed groups* | *2.50* | *1.20* | *1.50* | *1.00* | ***1.00**** | *0.01 (adj for BMI)* | *NA* |
|  | (Khazaal et al., 2006b) | Cross-sectional | NA | SGA-treated/unexposed, among those with BMI ≥28  n= 20/20 | SGA including olanzapine, clozapine, quetiapine, risperidone | All patients in the SGA-treated group: Duration of treatment with SGA in years, mean (SD): 8.3 (6.2) | SGA-treated: 32.90 (6.10)  Unexposed group: 33.80 (4.90) | Rigid Weight Regulation and Fear of Weight Gain | MAC-R subscale 2 | Difference in mean MAC-R subscale 2 scores between cases and controls | 24.10 | 7.90 | 24.00 | 5.80 | **0.10*** | 0.96* | (-4.34, 4.54)* |
|  | (Khazaal et al., 2009) | Cross-sectional | NA | SGA-treated/unexposed, among those with weight gain (minimum 2 kg in the last month)  n= 10/5 | All patients in the SGA-treated group: olanzapine, n=14; clozapine, n=2; risperidone, n=2; quetiapine, n=4. | Duration of treatment in SGA-treated group: NR | SGA-treated with weight gain: 28.60 (4.20) Unexposed with weight gain: 29.20 (4.30) | Cognitive dietary restraint | TFEQ subscale 1 | Difference in mean TEFQ subscale 1 scores between SGA-treated and unexposed groups | 7.70 | 2.40 | 4.60 | 3.80 | **3.10*** | 0.07* | (-0.34, 6.54)* |
|  | (Kouidrat et al., 2018) | Cross-sectional | NA | AP-treated/ unexposed  n= 66/81 | FGA, n=25; SGA, n= 20; both, n= 16 | AP-treated:  Duration of AP treatment: NR  Other medications, n (%): 26 (40) used antidepressants; 55 (84) used anxiolytics, 40 (60) used hypnotics. | AP-treated:  30.30 (8.20)  Unexposed:  24.00 (3.30)  *Analysis adjusted for BMI* | Cognitive dietary restraint | TEFQ-R21 subscale 1 | Difference in mean TFQ subscale 1 scores between cases and controls | 57.50 | 17.70 | 34.60 | 21.20 | **22.90**  **Adj MD: 27.6**  **[18.7-36.6]** | p<0.0001 (adj for sex, age, BMI and smoking status. | NA |
|  | (Sentissi et al., 2009) | Cross-sectional | NA | SGA-treated patients/Untreated patients  n= 93/33 | SGAs including clozapine, n=20; olanzapine, n=23; amisulpride, n=14; risperidone, n=20; aripiprazole, n=16. | Duration of prior AP treatment in AP-treated group (collectively n=120), months mean (SD): 36.20 (49.70)  Duration of prior AP treatment in untreated group (n=33):  AP naïve, n=23;  Without treatment for at least 3 months, n=10; mean duration without treatment: 7 months (7.9). | SGA-treated group:  26.90 (5.50)  Untreated group: NR | Cognitive dietary restraint | TEFQ subscale 1 | Difference in mean TEFQ subscale 1 scores between SGA-treated/untreated groups | 8.70 | 4.70 | 8.50 | 4.53 | **0.20*** | 0.83* | (-1.67, 2.07)* |
|  | (Sentissi et al., 2009) | Cross-sectional | NA | SGA-treated patients/Untreated patients  n= 93/33 | Clozapine, n=20; olanzapine, n=23; amisulpride, n=14; risperidone, n=20; aripiprazole, n=16. | Duration of prior AP treatment in AP-treated group (collectively n=120), months mean (SD): 36.20 (49.70)  Duration of prior AP treatment in untreated group (n=33):  AP naïve, n=23;  Without treatment for at least 3 months, n=10; mean duration without treatment: 7 months (7.9). | SGA-treated group:  26.90 (5.50)  Untreated group: NR | Cognitive dietary restraint | DEBQ subscale 1 | Difference in mean DEBQ subscale 1 scores between SGA-treated/untreated groups | 2.50 | 0.80 | 2.50 | 0.80 | **0*** | 1.00* | (-0.32, 0.32)* |
|  | (Sentissi et al., 2009) | Cross-sectional | NA | FGA-treated patients/Untreated patients  n= 27/33 | FGA, n=27 (mainly haloperidol: n = 16/27) | Duration of prior AP treatment in AP-treated group (collectively n=120), months mean (SD): 36.20 (49.70)  Duration of prior AP treatment in untreated group (n=33):  AP naïve, n=23;  Without treatment for at least 3 months, n=10; mean duration without treatment: 7 months (7.9). | FGA-treated group:  25.50 (6.00)  Untreated group: NR | Cognitive dietary restraint | TEFQ subscale 1 | Difference in mean TEFQ subscale 1 scores between FGA-treated/untreated groups | 7.60 | 4.90 | 8.50 | 4.53 | **-0.90*** | 0.46* | (-3.34, 1.54)* |
|  | (Sentissi et al., 2009) | Cross-sectional | NA | FGA-treated patients/Untreated patients  n= 27/33 | FGA, n=27 (mainly haloperidol: n = 16/27) | Duration of prior AP treatment in AP-treated group (collectively n=120), months mean (SD): 36.20 (49.70)  Duration of prior AP treatment in untreated group (n=33):  AP naïve, n=23;  Without treatment for at least 3 months, n=10; mean duration without treatment: 7 months (7.9). | FGA-treated group:  25.50 (6.00)  Untreated group: NR | Cognitive dietary restraint | DEBQ subscale 1 | Difference in mean DEBQ subscale 1 scores between FGA-treated/untreated groups | 2.30 | 0.60 | 2.50 | 0.80 | **-0.20*** | 0.29* | (-0.57, 0.17)* |
| **NORMAL** | (Khazaal et al., 2006b) | Cross-sectional | NA | SGA-treated group/unexposed group, among those with BMI <28  n= 20/20 | SGA including olanzapine, clozapine, quetiapine, risperidone | All patients in the SGA-treated group:  Duration of prior treatment with SGA in years, mean (SD): 8.3 (6.2) | SGA-treated: 23.60 (2.20)  Unexposed group: 21.10 (2.50) | Rigid Weight Regulation and Fear of Weight Gain | MAC-R subscale 2 | Difference in mean MAC-R subscale 2 scores between cases and controls | 20.50 | 6.60 | 14.70 | 5.70 | **5.80*** | 0.005* | (1.85, 9.75)* |
|  | (Khazaal et al., 2009) | Cross-sectional | NA | SGA-treated/unexposed, among those without weight gain (neither gained nor lost more than 1 kg in the last month)  n= 12/10 | All patients in the SGA-treated group: olanzapine, n=14; clozapine, n=2; risperidone, n=2; quetiapine, n=4. | Duration of treatment in SGA-treated group: NR | SGA-treated without weight gain: 24.00 (5.60) Unexposed without weight gain: 22.50 (1.70) | Cognitive dietary restraint | TFEQ subscale 1 | Difference in mean TFQ subscale 1 between SGA-treated and unexposed groups | 6.60 | 5.10 | 4.00 | 2.90 | **2.60*** | 0.17* | (-1.20, 6.40)* |
| **DISINHIBITION** | | | | | | | | | | | | | | | | |  |
| **HIGH** | (Blouin et al., 2008) | Cross-sectional | NA | SGA-treated group/Unexposed group  n= 18/20 | SGAs including clozapine: n = 2; olanzapine:  n = 9; risperidone: n = 2; quetiapine: n = 3; ziprasidone: n = 2. | Duration of current SGA treatment in months, mean (SD): 24.60 (19.70).  Duration of prior AP exposure (FGA or SGA) in SGA-treated group months, mean (SD): 35.30 (22.00).  SGA-treated group had to be sedentary (practicing <30 min of continuous physical activity per week).  Unexposed group matched by age and physical activity. | SGA-treated group:  28.80 (5.0)  Unexposed group:  25.0 (3.30)  *(Analysis adjusted for baseline BMI)* | Disinhibition | TFEQ subscale 2 | Difference in mean TFQ subscale 2 scores between cases and controls | 5.60 | 2.30 | 3.80 | 2.30 | **1.80*** | 0.03 (adj for BMI) | NA |
|  | (Blouin et al., 2008) | Cross-sectional | NA | SGA-treated group/Unexposed group  n= 18/20 | SGAs including clozapine: n = 2; olanzapine:  n = 9; risperidone: n = 2; quetiapine: n = 3; ziprasidone: n = 2. | Duration of current SGA treatment in months, mean (SD): 24.60 (19.70).  Duration of prior AP exposure (FGA or SGA) in SGA-treated group months, mean (SD): 35.30 (22.00).  SGA-treated group had to be sedentary (practicing <30 min of continuous physical activity per week).  Unexposed group matched by age and physical activity. | SGA-treated group:  28.80 (5.0)  Unexposed group:  25.0 (3.30)  *(Analysis adjusted for baseline BMI)* | *Disinhibition subscale: Habitual susceptibility* | *TFEQ subscale 2* | *Difference in mean TFQ subscale 2 (habitual susceptibility) scores between cases and controls* | *0.80* | *0.90* | *0.10* | *0.40* | ***0.70**** | *0.005 (adj for BMI)* | *NA* |
|  | (Blouin et al., 2008) | Cross-sectional | NA | SGA-treated group/Unexposed group  n= 18/20 | SGAs including clozapine: n = 2; olanzapine:  n = 9; risperidone: n = 2; quetiapine: n = 3; ziprasidone: n = 2. | Duration of current SGA treatment in months, mean (SD): 24.60 (19.70).  Duration of prior AP exposure (FGA or SGA) in SGA-treated group months, mean (SD): 35.30 (22.00).  SGA-treated group had to be sedentary (practicing <30 min of continuous physical activity per week).  Unexposed group matched by age and physical activity. | SGA-treated group:  28.80 (5.0)  Unexposed group:  25.0 (3.30)  *(Analysis adjusted for baseline BMI)* | *Disinhibition subscale: emotional susceptibility* | *TFEQ subscale 2* | *Difference in mean TFQ subscale 2 (emotional susceptibility) scores between cases and controls* | *0.90* | *1.10* | *0.30* | *0.90* | ***0.60**** | *0.05 (adj for BMI)* | *NA* |
|  | (Blouin et al., 2008) | Cross-sectional | NA | SGA-treated group/Unexposed group  n= 18/20 | SGAs including clozapine: n = 2; olanzapine:  n = 9; risperidone: n = 2; quetiapine: n = 3; ziprasidone: n = 2. | Duration of current SGA treatment in months, mean (SD): 24.60 (19.70).  Duration of prior AP exposure (FGA or SGA) in SGA-treated group months, mean (SD): 35.30 (22.00).  SGA-treated group had to be sedentary (practicing <30 min of continuous physical activity per week).  Unexposed group matched by age and physical activity. | SGA-treated group:  28.80 (5.0)  Unexposed group:  25.0 (3.30) | *Disinhibition subscale: situational susceptibility* | *TFEQ subscale 2* | *Difference in mean TFQ subscale 2 (situational susceptibility) scores between cases and controls* | *2.00* | *1.50* | *2.00* | *1.40* | ***0*** | *NA*  *(adj for BMI but not reported in the paper)* | *NA* |
|  | (Khazaal et al., 2009) | Cross-sectional | NA | SGA-treated/unexposed, among those with weight gain (minimum 2 kg in the last month)  n= 10/5 | All patients in the SGA-treated group: olanzapine, n=14; clozapine, n=2; risperidone, n=2; quetiapine, n=4. | Duration of treatment in SGA-treated group: NR | SGA-treated with weight gain: 28.60 (4.20) Unexposed with weight gain: 29.20 (4.30) | Disinhibition | TFEQ subscale 2 | Difference in mean TFQ subscale 2 scores between cases and controls | 7.10 | 2.80 | 6.60 | 2.30 | **0.50*** | 0.74* | (-2.64, 3.64)* |
|  | (Sentissi et al., 2009) | Cross-sectional | NA | SGA-treated patients/Untreated patients  n= 93/33 | Clozapine, n=20; olanzapine, n=23; amisulpride, n=14; risperidone, n=20; aripiprazole, n=16. | Duration of prior AP treatment in AP-treated group (collectively n=120), months mean (SD): 36.20 (49.70)  Duration of prior AP treatment in untreated group (n=33):  AP naïve, n=23;  Without treatment for at least 3 months, n=10; mean duration without treatment: 7 months (7.9). | SGA-treated group:  26.90 (5.50)  Untreated group: NR | Disinhibition | TEFQ subscale 2 | Difference in mean TEFQ subscale 2 scores between SGA-treated/untreated groups | 6.10 | 3.60 | 6.30 | 3.90 | **-0.20*** | 0.79* | (-1.68, 1.28)* |
|  | (Sentissi et al., 2009) | Cross-sectional | NA | FGA-treated patients/Untreated patients  n= 27/33 | FGA, n=27 (mainly haloperidol: n = 16/27) | Duration of prior AP treatment in AP-treated group (collectively n=120), months mean (SD): 36.20 (49.70)  Duration of prior AP treatment in untreated group (n=33):  AP naïve, n=23;  Without treatment for at least 3 months, n=10; mean duration without treatment: 7 months (7.9). | FGA-treated group:  25.50 (6.00)  Untreated group: NR | Disinhibition | TEFQ subscale 2 | Difference in mean TEFQ subscale 2 scores between FGA-treated/untreated groups | 4.50 | 2.80 | 6.30 | 3.90 | **-1.80*** | 0.05* | (-3.60, -0.01)* |
| **NORMAL** | (Khazaal et al., 2009) | Cross-sectional |  | SGA-treated/unexposed, among those without weight gain (neither gained nor lost more than 1 kg in the last month)  n= 12/10 | All patients in the SGA-treated group: olanzapine, n=14; clozapine, n=2; risperidone, n=2; quetiapine, n=4. | Duration of treatment in SGA-treated group: NR | SGA-treated without weight gain: 24.00 (5.60) Unexposed without weight gain: 22.50 (1.70) | Disinhibition | TFEQ subscale 2 | Difference in mean TFQ subscale 2 scores between cases and controls | 5.90 | 2.80 | 4.00 | 1.40 | **1.90*** | 0.07* | (-0.14, 3.94)* |
| ***UNCONTROLLED EATING*** | | | | | | | | | | | | | | | | |  |
| **HIGH** | (Kouidrat et al., 2018) | Cross-sectional | NA | AP-treated/ unexposed  n= 66/81 | FGA, n=25; SGA, n= 20; both, n= 16 | AP-treated:  Duration of AP treatment: NR  Other medications, n (%): 26 (40) used antidepressants; 55 (84) used anxiolytics, 40 (60) used hypnotics. | AP-treated:  30.30 (8.20)  Unexposed:  24.00 (3.30)  *Analysis adjusted for BMI* | Uncontrolled eating | TEFQ-21 subscale 2 | Difference in mean TFQ subscale 2 scores between cases and controls | 57.50 | 16.90 | 35.00 | 21.10 | **22.50**  **Adj MD: 27.3 [18.6-35.9]** | p<0.0001 (adj for sex, age, BMI,smoking status). | NA |
|  | (Sentissi et al., 2009) | Cross-sectional | NA | SGA-treated patients/Untreated patients  n= 93/33 | Clozapine, n=20; olanzapine, n=23; amisulpride, n=14; risperidone, n=20; aripiprazole, n=16. | Duration of prior AP treatment in AP-treated group (collectively n=120), months mean (SD): 36.20 (49.70)  Duration of prior AP treatment in untreated group (n=33):  AP naïve, n=23;  Without treatment for at least 3 months, n=10; mean duration without treatment: 7 months (7.9). | SGA-treated group:  26.90 (5.50)  Untreated group: NR | External eating factor | DEBQ subscale 3 | Difference in mean DEBQ subscale 3 scores between SGA-treated/untreated groups | 2.50 | 0.60 | 2.50 | 0.70 | **0*** | 1.00* | (-0.25, 0.25)* |
|  | (Sentissi et al., 2009) | Cross-sectional | NA | FGA-treated patients/Untreated patients  n= 27/33 | FGA, n=27 (mainly haloperidol: n = 16/27) | Duration of prior AP treatment in AP-treated group (collectively n=120), months mean (SD): 36.20 (49.70)  Duration of prior AP treatment in untreated group (n=33):  AP naïve, n=23;  Without treatment for at least 3 months, n=10; mean duration without treatment: 7 months (7.9). | FGA-treated group:  25.50 (6.00)  Untreated group: NR | External eating factor | DEBQ subscale 3 | Difference in mean DEBQ subscale 3 scores between FGA-treated/untreated groups | 2.20 | 0.50 | 2.50 | 0.70 | **-0.30*** | 0.07* | (-0.62, 0.02)* |
| ***EMOTIONAL EATING*** | | | | | | | | | | | | | | | | |  |
| **HIGH** | (Kouidrat et al., 2018) | Cross-sectional | NA | AP-treated/ unexposed  n= 66/81 | FGA, n=25; SGA, n= 20; both, n= 16 | AP-treated:  Duration of AP treatment: NR  Other medications, n (%): 26 (40) used antidepressants; 55 (84) used anxiolytics, 40 (60) used hypnotics. | AP-treated:  30.30 (8.20)  Unexposed:  24.00 (3.30)  *Analysis adjusted for BMI* | Emotional Eating | TEFQ-21 subscale 3 | Difference in mean TEFQ-21 subscale 3 scores between cases and controls | 69.10 | 29.50 | 41.40 | 27.20 | **27.70**  **Adj MD: 27.6 [18.7-36.6]** | p<0.0001 (adj for sex, age, BMI and smoking status.) | NA |
|  | (Sentissi et al., 2009) | Cross-sectional | NA | SGA-treated patients/Untreated patients  n= 93/33 | Clozapine, n=20; olanzapine, n=23; amisulpride, n=14; risperidone, n=20; aripiprazole, n=16. | Duration of prior AP treatment in AP-treated group (collectively n=120), months mean (SD): 36.20 (49.70)  Duration of prior AP treatment in untreated group (n=33):  AP naïve, n=23;  Without treatment for at least 3 months, n=10; mean duration without treatment: 7 months (7.9). | SGA-treated group:  26.90 (5.50)  Untreated group: NR | Emotional eating | DEBQ subscale 2 | Difference in mean DEBQ subscale 2 scores between SGA-treated/untreated groups | 2.70 | 0.60 | 2.60 | 0.70 | **0.1*** | 0.43* | (-0.15, 0.35)* |
|  | (Sentissi et al., 2009) | Cross-sectional | NA | FGA-treated patients/Untreated patients  n= 27/33 | FGA, n=27 (mainly haloperidol: n = 16/27) | Duration of prior AP treatment in AP-treated group (collectively n=120), months mean (SD): 36.20 (49.70)  Duration of prior AP treatment in untreated group (n=33):  AP naïve, n=23;  Without treatment for at least 3 months, n=10; mean duration without treatment: 7 months (7.9)’ | FGA-treated group:  25.50 (6.00)  Untreated group: NR | Emotional eating | DEBQ subscale 2 | Difference in mean DEBQ subscale 2 scores between FGA-treated/untreated groups | 2.50 | 0.60 | 2.60 | 0.70 | **-0.1*** | 0.56* | (-0.44, 0.24)* |
| **HUNGER** | | | | | | | | | | | | | | | | |  |
| **HIGH** | (Blouin et al., 2008) | Cross-sectional | NA | SGA-treated group/Unexposed group  n= 18/20 | SGAs including clozapine: n = 2; olanzapine:  n = 9; risperidone: n = 2; quetiapine: n = 3; ziprasidone: n = 2. | Duration of current SGA treatment in months, mean (SD): 24.60 (19.70).  Duration of prior AP exposure (FGA or SGA) in SGA-treated group months, mean (SD): 35.30 (22.00).  SGA-treated group had to be sedentary (practicing <30 min of continuous physical activity per week).  Unexposed group matched by age and physical activity. | SGA-treated group:  28.80 (5.0)  Unexposed group:  25.0 (3.30)  *(Analysis adjusted for baseline BMI)* | Susceptibility to hunger | TFEQ subscale 3 | Difference in mean TFQ subscale 3 scores between cases and controls | 5.70 | 2.40 | 2.40 | 2.30 | **3.30*** | 0.0001 (adj for BMI) | NA |
|  | (Blouin et al., 2008) | Cross-sectional | NA | SGA-treated group/Unexposed group  n= 18/20 | SGAs including clozapine: n = 2; olanzapine:  n = 9; risperidone: n = 2; quetiapine: n = 3; ziprasidone: n = 2. | Duration of current SGA treatment in months, mean (SD): 24.60 (19.70).  Duration of prior AP exposure (FGA or SGA) in SGA-treated group months, mean (SD): 35.30 (22.00).  SGA-treated group had to be sedentary (practicing <30 min of continuous physical activity per week).  Unexposed group matched by age and physical activity. | SGA-treated group:  28.80 (5.0)  Unexposed group:  25.0 (3.30)  *(Analysis adjusted for baseline BMI)* | *Susceptibility to hunger subscale: internal locus* | *TFEQ subscale 3* | *Difference in mean TFQ subscale 3 (internal locus) scores between cases and controls* | *2.00* | *1.80* | *0.60* | *1.00* | ***1.40*** | *0.009 (adj for BMI)* | *NA* |
|  | (Blouin et al., 2008) | Cross-sectional | NA | SGA-treated group/Unexposed group  n= 18/20 | SGAs including clozapine: n = 2; olanzapine:  n = 9; risperidone: n = 2; quetiapine: n = 3; ziprasidone: n = 2. | Duration of current SGA treatment in months, mean (SD): 24.60 (19.70).  Duration of prior AP exposure (FGA or SGA) in SGA-treated group months, mean (SD): 35.30 (22.00).  SGA-treated group had to be sedentary (practicing <30 min of continuous physical activity per week).  Unexposed group matched by age and physical activity. | SGA-treated group:  28.80 (5.0)  Unexposed group:  25.0 (3.30)  *(Analysis adjusted for baseline BMI)* | *Susceptibility to hunger subscale: external locus* | *TFEQ subscale 3* | *Difference in mean TFQ subscale 3 (external locus) scores between cases and controls* | *2.40* | *0.70* | *1.20* | *1.10* | ***1.20*** | *0.0005 (adj for BMI)* | *NA* |
|  | (Khazaal et al., 2009) | Cross-sectional | NA | SGA-treated/unexposed, among those with weight gain (minimum 2 kg in the last month)  n= 10/5 | All patients in the SGA-treated group: olanzapine, n=14; clozapine, n=2; risperidone, n=2; quetiapine, n=4. | Duration of treatment in SGA-treated group: NR | SGA-treated with weight gain: 28.60 (4.20) Unexposed with weight gain: 29.20 (4.30) | Susceptibility to hunger | TFEQ subscale 3 | Difference in mean TFQ subscale 3 scores between cases and controls | 6.60 | 3.00 | 6.20 | 4.00 | **0.40*** | 0.83 * | (-3.55, 4.35)* |
|  | (Sentissi et al., 2009) | Cross-sectional | NA | SGA-treated patients/Untreated patients  n= 93/33 | Clozapine, n=20; olanzapine, n=23; amisulpride, n=14; risperidone, n=20; aripiprazole, n=16. | Duration of prior AP treatment in AP-treated group (collectively n=120), months mean (SD): 36.20 (49.70)  Duration of prior AP treatment in untreated group (n=33):  AP naïve, n=23;  Without treatment for at least 3 months, n=10; mean duration without treatment: 7 months (7.9). | SGA-treated group:  26.90 (5.50)  Untreated group: NR | Susceptibility to hunger | TFEQ subscale 3 | Difference in mean TEFQ subscale 3 scores between SGA-treated/untreated groups | 5.70 | 3.10 | 6.00 | 3.80 | **-0.3*** | 0.65* | (-1.62, 1.02)* |
|  | (Sentissi et al., 2009) | Cross-sectional | NA | FGA-treated patients/Untreated patients  n= 27/33 | FGA, n=27 (mainly haloperidol: n = 16/27) | Duration of prior AP treatment in AP-treated group (collectively n=120), months mean (SD): 36.20 (49.70)  Duration of prior AP treatment in untreated group (n=33):  AP naïve, n=23;  Without treatment for at least 3 months, n=10; mean duration without treatment: 7 months (7.9). | FGA-treated group:  25.50 (6.00)  Untreated group: NR | Susceptibility to hunger | TFEQ subscale 3 | Difference in mean TEFQ subscale 3 scores between FGA-treated/untreated groups | 4.80 | 3.30 | 6.00 | 3.80 | **-1.20*** | 0.20* | (-3.06, -0.66)* |
| **NORMAL** | (Khazaal et al., 2009) | Cross-sectional | NA | SGA-treated/unexposed, among those without weight gain (neither gained nor lost more than 1 kg in the last month)  n= 12/10 | All patients in the SGA-treated group: olanzapine, n=14; clozapine, n=2; risperidone, n=2; quetiapine, n=4. | Duration of treatment in SGA-treated group: NR | SGA-treated without weight gain: 24.00 (5.60) Unexposed without weight gain: 22.50 (1.70) | Susceptibility to hunger | TFEQ subscale 3 | Difference in mean TFQ subscale 3 scores between cases and controls | 7.40 | 3.50 | 4.00 | 3.00 | **3.40*** | 0.03 * | (0.47, 6.33)* |
| **APPEARANCE, WEIGHT AND APPROVAL** | | | | | | | | | | | | | | | | |  |
| **HIGH** | (Khazaal et al., 2006b) | Cross-sectional | NA | SGA-treated/unexposed, among those with BMI ≥28  n= 20/20 | SGA including olanzapine, clozapine, quetiapine, risperidone | All patients in the SGA-treated group: Duration of treatment with SGA in years, mean (SD): 8.3 (6.2) | SGA-treated: 32.90 (6.10)  Unexposed group: 33.80 (4.90) | Approval | MAC-R subscale 1 | Difference in mean MAC-R subscale 1 scores between cases and controls | 21.30 | 6.80 | 20.60 | 3.50 | **0.70*** | 0.70* | (-2.76, 4.16)* |
| **NORMAL** | (Khazaal et al., 2006b) | Cross-sectional | NA | SGA-treated group/unexposed group, among those with BMI <28  n= 20/20 | SGA including olanzapine, clozapine, quetiapine, risperidone | All patients in the SGA-treated group:  Duration of prior treatment with SGA in years, mean (SD): 8.3 (6.2) | SGA-treated: 23.60 (2.20)  Unexposed group: 21.10 (2.50) | Approval | MAC-R subscale 1 | Difference in mean MAC-R subscale 1 scores between cases and controls | 20.00 | 6.20 | 16.50 | 4.60 | **3.50*** | 0.05* | (0.01, 6.99)* |
| **SELF-CONTROL OF EATING AND SELF-ESTEEM** | | | | | | | | | | | | | | | | |  |
| **HIGH** | (Khazaal et al., 2006b) | Cross-sectional | NA | SGA-treated/unexposed, among those with BMI ≥28  n= 20/20 | SGA including olanzapine, clozapine, quetiapine, risperidone | All patients in the SGA-treated group: Duration of treatment with SGA in years, mean (SD): 8.3 (6.2) | SGA-treated: 32.90 (6.10)  Unexposed group: 33.80 (4.90) | Self-control | MAC-R subscale 3 | Difference in mean MAC-R subscale 3 scores between cases and controls | 27.20 | 5.80 | 28.20 | 6.50 | **-1.0*** | 0.61* | (-4.94, 2.94)* |
| **NORMAL** | (Khazaal et al., 2006b) | Cross-sectional | NA | SGA-treated group/unexposed group, among those with BMI <28  n= 20/20 | SGA including olanzapine, clozapine, quetiapine, risperidone | All patients in the SGA-treated group:  Duration of prior treatment with SGA in years, mean (SD): 8.3 (6.2) | SGA-treated: 23.60 (2.20)  Unexposed group: 21.10 (2.50) | Self-control | MAC-R subscale 3 | Difference in mean MAC-R subscale 3 scores between cases and controls | 26.20 | 5.80 | 21.70 | 7.60 | **4.50*** | 0.04* | (0.17, 8.83)* |

AP= antipsychotic; BMI= body mass index; DEBQ= Dutch Eating Behavior Questionnaire; FGA= first-generation antipsychotic; MAC-R= Revised version of the Mizes Anorectic Cognitions questionnaire; n= number; NA= not applicable; SGA= second-generation antipsychotic; TFEQ= Three-Factor Eating Questionnaire; TFEQ-R21= Three-Factor Eating Questionnaire-Revised 21-Item Version.

# References

Abbas, M. J. & Liddle, P. F. (2013). ‘Olanzapine and food craving: A case control study’ *Hum Psychopharmacol*, 28 (1), pp. 97-101. DOI: 10.1002/hup.2278 Available at: <https://www.ncbi.nlm.nih.gov/pubmed/23169487>.

Archie, S. M., et al. (2007). ‘Psychotic disorders, eating habits, and physical activity: Who is ready for lifestyle changes?’ *Psychiatric Services*, 58 (2), pp. 233-239. DOI: 10.1176/ps.2007.58.2.233.

Ballon, J. S., et al. (2018). ‘Pathophysiology of drug induced weight and metabolic effects: Findings from an rct in healthy volunteers treated with olanzapine, iloperidone, or placebo’ *J Psychopharmacol*, 32 (5), pp. 533-540. DOI: 10.1177/0269881118754708 Available at: <https://www.ncbi.nlm.nih.gov/pubmed/29444618>.

Blouin, M., et al. (2008). ‘Adiposity and eating behaviors in patients under second generation antipsychotics’ *Obesity (Silver Spring)*, 16 (8), pp. 1780-7. DOI: 10.1038/oby.2008.277 Available at: <https://www.ncbi.nlm.nih.gov/pubmed/18535555>.

Daurignac, E., Leonard, K. E. & Dubovsky, S. L. (2015). ‘Increased lean body mass as an early indicator of olanzapine-induced weight gain in healthy men’ *Int Clin Psychopharmacol*, 30 (1), pp. 23-8. DOI: 10.1097/YIC.0000000000000052 Available at: <https://www.ncbi.nlm.nih.gov/pubmed/25350366>.

Fountaine, R. J., et al. (2010). ‘Increased food intake and energy expenditure following administration of olanzapine to healthy men’ *Obesity (Silver Spring)*, 18 (8), pp. 1646-51. DOI: 10.1038/oby.2010.6 Available at: <https://www.ncbi.nlm.nih.gov/pubmed/20134408> (Accessed: 2023/06/22).

Garriga, M., et al. (2019). ‘Food craving and consumption evolution in patients starting treatment with clozapine’ *Psychopharmacology (Berl)*, 236 (11), pp. 3317-3327. DOI: 10.1007/s00213-019-05291-3 Available at: <https://www.ncbi.nlm.nih.gov/pubmed/31197435>.

Gothelf, D., et al. (2002). ‘Weight gain associated with increased food intake and low habitual activity levels in male adolescent schizophrenic inpatients treated with olanzapine’ *Am J Psychiatry*, 159 (6), pp. 1055-7. DOI: 10.1176/appi.ajp.159.6.1055 Available at: <https://www.ncbi.nlm.nih.gov/pubmed/12042200>.

Henderson, D. C., et al. (2006). ‘Dietary intake profile of patients with schizophrenia’ *Ann Clin Psychiatry*, 18 (2), pp. 99-105. DOI: 10.1080/10401230600614538 Available at: <https://www.ncbi.nlm.nih.gov/pubmed/16754415>.

Hoffman, V. P., Case, M. & Jacobson, J. G. 'Algorithms including amantadine, metformin and zonisamide for mitigation of weight gain during olanzapine treatment in outpatients with schizophrenia', *APA San Francisco*

Jakobsen, A. S., et al. (2018). ‘Dietary patterns and physical activity in people with schizophrenia and increased waist circumference’ *Schizophr Res*, 199 pp. 109-115. DOI: 10.1016/j.schres.2018.03.016 Available at: <https://www.ncbi.nlm.nih.gov/pubmed/29555213>.

Kang, D., et al. (2024). ‘The effect of continuous theta burst stimulation on antipsychotic-induced weight gain in first-episode drug-naive individuals with schizophrenia: A double-blind, randomized, sham-controlled feasibility trial’ *Transl Psychiatry*, 14 (1), p. 61. DOI: 10.1038/s41398-024-02770-w Available at: <https://www.ncbi.nlm.nih.gov/pubmed/38272892>.

Khazaal, Y., et al. (2009). ‘Hunger and negative alliesthesia to aspartame and sucrose in patients treated with antipsychotic drugs and controls’ *Eat Weight Disord*, 14 (4), pp. e225-30. DOI: 10.1007/BF03325121 Available at: <https://www.ncbi.nlm.nih.gov/pubmed/20179410>.

Khazaal, Y., Fresard, E., Borgeat, F. & Zullino, D. (2006a). ‘Binge eating symptomatology in overweight and obese patients with schizophrenia: A case control study’ *Ann Gen Psychiatry*, 5 p. 15. DOI: 10.1186/1744-859X-5-15 Available at: <https://www.ncbi.nlm.nih.gov/pubmed/16968528>.

Khazaal, Y., et al. (2006b). ‘Eating and weight related cognitions in people with schizophrenia : A case control study’ *Clin Pract Epidemiol Ment Health*, 2 p. 29. DOI: 10.1186/1745-0179-2-29 Available at: <https://www.ncbi.nlm.nih.gov/pubmed/17076886>.

Khosravi, M. (2020). ‘Biopsychosocial factors associated with disordered eating behaviors in schizophrenia’ *Ann Gen Psychiatry*, 19 (1), p. 67. DOI: 10.1186/s12991-020-00314-2 Available at: <https://www.ncbi.nlm.nih.gov/pubmed/33292324>.

Kouidrat, Y., et al. (2018). ‘Disordered eating behaviors as a potential obesogenic factor in schizophrenia’ *Psychiatry Res*, 269 pp. 450-454. DOI: 10.1016/j.psychres.2018.08.083 Available at: <https://www.ncbi.nlm.nih.gov/pubmed/30195737>.

Mathews, J., et al. (2012). ‘Neural correlates of weight gain with olanzapine’ *Arch Gen Psychiatry*, 69 (12), pp. 1226-37. DOI: 10.1001/archgenpsychiatry.2012.934 Available at: <https://www.ncbi.nlm.nih.gov/pubmed/22868896>.

Nunes, D., et al. (2014). ‘Nutritional status, food intake and cardiovascular disease risk in individuals with schizophrenia in southern brazil: A case-control study’ *Rev Psiquiatr Salud Ment*, 7 (2), pp. 72-9. DOI: 10.1016/j.rpsm.2013.07.001 Available at: <https://www.ncbi.nlm.nih.gov/pubmed/24054065>.

Park, S., Yi, K. K., Kim, M. S. & Hong, J. P. (2013). ‘Effects of ziprasidone and olanzapine on body composition and metabolic parameters: An open-label comparative pilot study’ *Behav Brain Funct*, 9 p. 27. DOI: 10.1186/1744-9081-9-27 Available at: <https://www.ncbi.nlm.nih.gov/pubmed/23866300>.

Roerig, J. L., et al. (2005). ‘A comparison of the effects of olanzapine and risperidone versus placebo on eating behaviors’ *J Clin Psychopharmacol*, 25 (5), pp. 413-8. DOI: 10.1097/01.jcp.0000177549.36585.29 Available at: <https://www.ncbi.nlm.nih.gov/pubmed/16160615>.

Saugo, E., et al. (2020). ‘Dietary habits and physical activity in first-episode psychosis patients treated in community services. Effect on early anthropometric and cardio-metabolic alterations’ *Schizophr Res*, 216 pp. 374-381. DOI: 10.1016/j.schres.2019.11.010 Available at: <https://www.ncbi.nlm.nih.gov/pubmed/31806524>.

Sentissi, O., et al. (2009). ‘Impact of antipsychotic treatments on the motivation to eat: Preliminary results in 153 schizophrenic patients’ *Int Clin Psychopharmacol*, 24 (5), pp. 257-64. DOI: 10.1097/YIC.0b013e32832b6bf6 Available at: <https://www.ncbi.nlm.nih.gov/pubmed/19606055>.

Smith, R. C., Rachakonda, S., Dwivedi, S. & Davis, J. M. (2012). ‘Olanzapine and risperidone effects on appetite and ghrelin in chronic schizophrenic patients’ *Psychiatry Res*, 199 (3), pp. 159-63. DOI: 10.1016/j.psychres.2012.03.011 Available at: <https://www.ncbi.nlm.nih.gov/pubmed/22475524>.

Stefanska, E., et al. (2017). ‘Eating habits and nutritional status of patients with affective disorders and schizophrenia’ *Psychiatr Pol*, 51 (6), pp. 1107-1120. DOI: 10.12740/PP/74558 Available at: <https://www.ncbi.nlm.nih.gov/pubmed/29432506>.

Stefanska, E., et al. (2018). ‘The assessment of the nutritional value of meals consumed by patients with recognized schizophrenia’ *Rocz Panstw Zakl Hig*, 69 (2), pp. 183-192. Available at: <https://www.ncbi.nlm.nih.gov/pubmed/29766697>.

Stip, E., et al. (2012). ‘Neural changes associated with appetite information processing in schizophrenic patients after 16 weeks of olanzapine treatment’ *Transl Psychiatry*, 2 (6), p. e128. DOI: 10.1038/tp.2012.53 Available at: <https://www.ncbi.nlm.nih.gov/pubmed/22714121>.

Teff, K. L., Rickels, K., Alshehabi, E. & Rickels, M. R. (2015). ‘Metabolic impairments precede changes in hunger and food intake following short-term administration of second-generation antipsychotics’ *J Clin Psychopharmacol*, 35 (5), pp. 579-82. DOI: 10.1097/JCP.0000000000000393 Available at: <https://www.ncbi.nlm.nih.gov/pubmed/26274045>.

Teff, K. L., et al. (2013). ‘Antipsychotic-induced insulin resistance and postprandial hormonal dysregulation independent of weight gain or psychiatric disease’ *Diabetes*, 62 (9), pp. 3232-40. DOI: 10.2337/db13-0430 Available at: <https://www.ncbi.nlm.nih.gov/pubmed/23835329> (Accessed: 5/1/2023).

Treuer, T., et al. (2009). ‘Factors associated with weight gain during olanzapine treatment in patients with schizophrenia or bipolar disorder: Results from a six-month prospective, multinational, observational study’ *World J Biol Psychiatry*, 10 (4 Pt 3), pp. 729-40. DOI: 10.1080/15622970903079507 Available at: <https://www.ncbi.nlm.nih.gov/pubmed/19606406>.
