# Supplementary material for: The impacts of antipsychotic medications on eating-related outcomes: A mixed methods systematic review
Source: PLoS One. 2025 Feb 3;20(2):e0308037. doi: 10.1371/journal.pone.0308037 (PMC11790239; doi:10.1371/journal.pone.0308037)
Supplement: S10 File — (DOCX) [file pone.0308037.s010.docx]

**S10 File. The GRADE Evidence Profile for key outcomes.**

# Within-group syntheses: GRADE assessment for the certainty of evidence

**GRADE evidence profile**

**Question: What are the effects of antipsychotic medications (exposure) on key eating-related outcomes in-within group syntheses?**

| **Certainty assessment** | | | | | | | | **Summary of findings** | |
| --- | --- | --- | --- | --- | --- | --- | --- | --- | --- |
| **Outcome** | **Study design** | **No. of participants (studies)** | **Follow-up/measurement scale** | **Risk of bias** | **Inconsistency** | **Indirectness** | **Imprecision** | **Vote counting with direction of effect** | **Quality** |
| **Appetite** | RCTs  (Hardy et al., 2009; Karagianis et al., 2009; Park et al., 2013; Smith et al., 2012) | 219 (4) | Follow-up: range from 2 weeks to 5 months; assessed with EBA Hardy et al. (2009), PARS (Karagianis et al., 2009), VAS and EBA (Smith et al., 2012), VAS (Park et al., 2013) | Very serious:  The 4 RCTs (Hardy et al., 2009; Karagianis et al., 2009; Park et al., 2013; Smith et al., 2012) did not provide details of concealment of allocation, 3 RCTs did not provide details of randomisation (Hardy et al., 2009; Karagianis et al., 2009; Park et al., 2013), and 2 were unblinded (Park et al., 2013; Smith et al., 2012).  Across the 4 RCTS, appetite was measured as a collective construct, including subjective appetite, hunger, fullness, desire to eat and/or eating cognitions and non-psychometrically tested measures were used.  Difference in outcome measures in terms of time frame and constructs being tested. | Not serious:  Variation in results may be attributed to baseline BMI. | Serious:  Difference in outcome measures in terms of constructs being tested. | Very serious:  Small sample size (OIS could not be calculated. CI of effect estimates not reported). | Three of four samples (Karagianis et al., 2009; Smith et al., 2012) with high baseline BMI receiving olanzapine or risperidone reported decline in appetite from baseline, 1 reported increase in appetite (Hardy et al., 2009).  Two samples with normal baseline BMI (Park et al., 2013) receiving ziprasidone or olanzapine reported no effect. | Very low  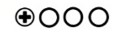 |
|  | Prospective, observational (Treuer et al., 2009) | 606 (1) | Follow-up: 4 weeks; assessed using EAS (Treuer et al., 2009) | Very serious:  Convenience sampling; use of non-validated outcome measure; failure to adequately control confounding. | NA | Serious:  In terms of constructs being tested using EAS. | Serious:  (OIS could not be calculated. CI of effect estimates not reported). | A prospective, observational study including olanzapine-treated patients with normal baseline BMI (Treuer et al., 2009) reported a decline in appetite scores over a four-week period. | Very low  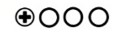 |
| **General food cravings** | RCT  (Hoffman et al., 2009) | 50 (1) | Follow-up: 2 weeks; assessed using FCI | Serious:  RCT (Hoffman et al., 2009) did not provide details of randomisation or concealment of allocation and was unblinded. | NA | Not serious | Very serious:  OIS could not be calculated. CI of effect estimate not reported. | One RCT (Hoffmann et al., 2012), including participants with high baseline BMI, reported a decline in general food craving scores (using the FCI) over a two-week olanzapine treatment. | Low  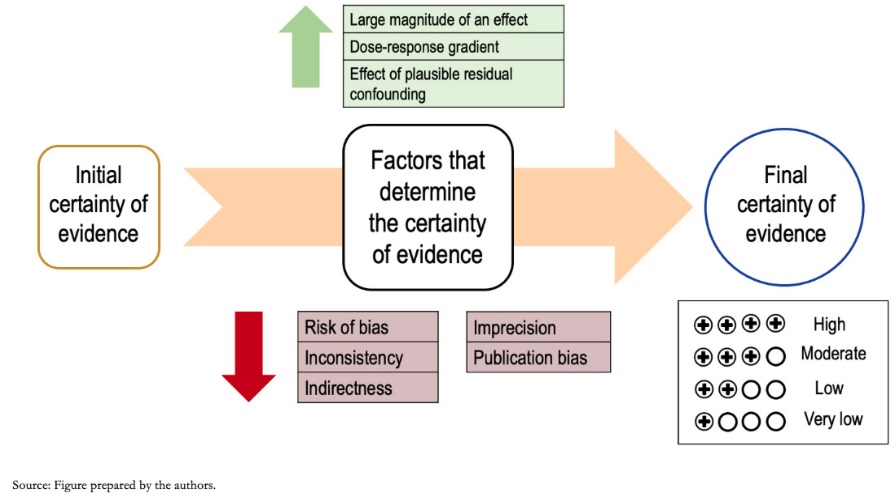 |
|  | Prospective, observational (Garriga et al., 2019) | 34 (1) | Follow-up: 18 weeks; using FCI-SP | Serious:  Convenience sampling | NA | Not serious | Serious:  OIS could not be calculated. Wide CI of effect estimates. | A prospective, observational study (Garriga et al., 2019), including two independent samples stratified by baseline BMI, investigated the effect of an 18-week clozapine treatment on food cravings. Normal weight patients experienced a consistent increase in general and specific food cravings from baseline, whereas overweight or obese patients experienced a decline in general and specific food carvings, except for complex carbohydrates/proteins (Garriga et al., 2019). | Very low  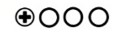 |
| **Hunger** | RCTs of healthy volunteers (Ballon et al., 2018; Roerig et al., 2005; Teff et al., 2013) | 50 (3) | Follow-up: ranging from 12 to 28 days; assessed using VAS. | Very serious:  3 RCTs did not provide details of randomisation or concealment of allocation; used non-validated outcome measures. | Not serious | Serious:  RCTs of healthy volunteers, not representative of target population.  Difference in outcome measures in terms of time frame and construct. | Very serious:  Small sample size. OIS could not be calculated. CI of effect estimates not reported. | The impact of olanzapine, iloperidone and aripiprazole on hunger was assessed in a laboratory setting in 3 RCTs of healthy volunteers with normal baseline BMI who were followed up for 28, 14 and 12 days, respectively (Ballon et al., 2018; Roerig et al., 2005; Teff et al., 2013). They all reported an increase in hunger scores from baseline. | Very low  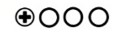 |
| **Energy intake** | Observational, prospective study (Gothelf et al., 2002) | 10 (1) | Follow-up: 4 weeks; assessed by clinical dietician calculating intake | Very serious:  No information on sampling technique; failure to adequately control confounding. | NA | Serious  Study included only male participants. | Very serious:  Small sample size. OIS could not be calculated. CI of effect estimate not reported. | Only 1 observational, prospective study (Gothelf et al., 2002) examined the change in energy and macronutrient intake among olanzapine-treated patients with normal baseline BMI. It reported an increase in energy intake over 4 weeks. | Very low  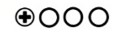 |
|  | RCTs of healthy volunteers (Ballon et al., 2018; Roerig et al., 2005; Teff et al., 2013) | 66 (3) | Follow-up: ranging from 12 to 28 days; calculated in a lab setting. | Serious:  3 RCTs did not provide details of randomisation or concealment of allocation. | Not serious:  Variation in results may be attributed to type of AP being studied. | Serious:  RCTs of healthy volunteers, not representative of target population.  Difference in outcome measures in terms of time frame. | Very serious:  Small sample size. OIS could not be calculated. CI of effect estimates not reported. | 3 RCTs of healthy volunteers with normal baseline BMI (Ballon et al., 2018; Roerig et al., 2005; Teff et al., 2015) found an increase in energy intake from baseline in olanzapine-treated participants. However, a contradictory effect was observed in healthy volunteers receiving iloperidone (Ballon et al., 2018), risperidone (Roerig et al., 2005) and aripiprazole (Teff et al., 2015). | Very low  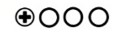 |
| **Eating cognitions and behaviour:** **dietary restraint** | RCT (Hoffman et al., 2009; Kang et al., 2024) | 36 (2) | Follow-up: ranging from 5 days to 2 weeks; assessed using TFEQ-R21 and TFEQ.. | Very serious:  1 RCT (Kang et al., 2024) provided details of randomisation, concealment of allocation and blinding; 1 RCT (Hoffman et al., 2009) did not provide details of randomisation or concealment of allocation and was unblinded. | NA | Not serious | Very serious:  Small sample sizes. OIS could not be calculated. CI of effect estimate not reported. | RCT of patients with high BMI (Hoffman et al., 2009) showed an increase in restraint scores over a 2-week olanzapine treatment, while RCT of AP-naïve patients with normal BMI (Kang et al., 2024) showed a decrease in restraint scores over a 5-day olanzapine treatment. | Very low  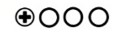 |
|  | Non-randomised controlled trial (Stip et al., 2012) | 15 (1) | Follow-up: 16 weeks; assessed using TFEQ | Very serious:  Convenience sample, >20% of participants in olanzapine arm lost to follow-up/dropped out. | NA | Not serious | Very serious:  Small sample size. OIS could not be calculated. CI of effect estimate not reported. | A non-randomised trial (Stip et al., 2012) of overweight patients studied the effect of a 16-week olanzapine treatment on TEFQ scores and showed a negative effect on restraint. | Very low  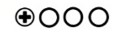 |
| **Eating cognitions and behaviours: dietary disinhibition** | RCT (Hoffman et al., 2009) | 17 (1) | Follow-up: 2 weeks; assessed using TFEQ | Very serious:  RCT (Hoffman et al., 2009) did not provide details of randomisation or concealment of allocation and was unblinded. | NA | Not serious | Very serious:  Small sample sizes. OIS could not be calculated. CI of effect estimate not reported. | RCT of patients with high baseline BMI (Hoffman et al., 2009) showed a decline in disinhibition scores over a 2-week olanzapine treatment. | Very low  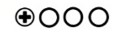 |
|  | Non-randomised controlled trial (Stip et al., 2012) | 15 (1) | Follow-up: 16 weeks; assessed using TFEQ | Very serious:  Convenience sample, >20% of participants in olanzapine arm lost to follow-up/dropped out. | NA | Not serious | Very serious:  Small sample size. OIS could not be calculated. CI of effect estimate not reported. | A non-randomised trial (Stip et al., 2012) of overweight patients studied the effect of a 16-week olanzapine treatment on TEFQ scores and showed no effect on disinhibition. | Very low  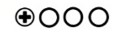 |
|  | Pre-post study of healthy volunteers (Mathews et al., 2012) | 19 (1) | Follow-up: 7 days; assessed using TEFQ | Very serious:  Convenience sampling technique; failure to adequately control for confounding. | NA | Serious:  Healthy volunteers, not representative of target population. | Very serious:  Small sample size. OIS could not be calculated. CI of effect estimate not reported. | A pre-post study of healthy volunteers (Mathews et al., 2012)with high baseline BMI investigated the effect of a 7-day olanzapine treatment on TFEQ scores and found an increase in the overall and disinhibition scores from baseline. | Very low  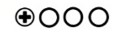 |
| **Eating cognitions and behaviours: hunger** | RCT (Hoffman et al., 2009) | 17 (1) | Follow-up: 2 weeks; assessed using TFEQ | Very serious:  RCT (Hoffman et al., 2009) did not provide details of randomisation or concealment of allocation and was unblinded. | NA | Not serious | Very serious:  Small sample size. OIS could not be calculated. CI of effect estimate not reported. | RCT of patients with high baseline BMI (Hoffman et al., 2009) showed a decline in hunger scores over a 2-week olanzapine treatment. | Very low  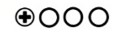 |
|  | Non-randomised controlled trial (Stip et al., 2012) | 15 (1) | Follow-up: 16 weeks; assessed using TFEQ | Very serious:  Convenience sample, >20% of participants in olanzapine arm lost to follow-up/dropped out. | NA | Not serious | Very serious:  Small sample size. OIS could not be calculated. CI of effect estimate not reported. | A non-randomised trial (Stip et al., 2012) of overweight patients studied the effect of a 16-week olanzapine treatment on TEFQ scores and showed a positive effect on susceptibility to hunger. | Very low  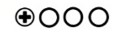 |

BMI= body mass index; CI= confidence interval; EAS= Eating Attitude Scale; EBA= Eating Behavior Assessment; GRADE= Grading of Recommendations, Assessment, Development and Evaluation; OIS= optimal information size; RCT= randomised controlled study; PARS= Platypus Appetite Rating scale; TFEQ= Three-Factor Eating Questionnaire; TFEQ-R21= Three-Factor Eating Questionnaire-Revised 21-item Version; VAS= visual analogue scale.

# Between-group syntheses: GRADE assessment for the certainty of evidence

**Evidence profile**

**Question: What are the effects of antipsychotic medications (exposure) on key eating-related outcomes in between-group syntheses?**

| **Certainty assessment** | | | | | | | | **Summary of findings** | |
| --- | --- | --- | --- | --- | --- | --- | --- | --- | --- |
| **Outcome** | **Study design** | **No. of participants: [exposed/unexposed] (studies)** | **Follow-up/**  **measurement scale** | **Risk of bias** | **Inconsistency** | **Indirectness** | **Imprecision** | **Vote counting with direction of effect** | **Quality** |
| **Appetite** | RCT (Roerig et al., 2005) | 48 [olanzapine arm: 16, risperidone arm: 16/ placebo:16]  (1) | Follow-up: 2 weeks; assessed using VAS. | Very serious:  Did not provide details of randomisation or concealment of allocation; used non-validated outcome measures. | NA | Serious:  RCT of healthy volunteers, not representative of target population. | Very serious:  Small sample size. OIS could not be calculated. 95% CI of effect estimates: wide. | Only one RCT (Roerig et al., 2005) of healthy volunteers with normal baseline BMI examined the effect of olanzapine and risperidone on appetite. It reported higher appetite scores in the olanzapine and risperidone arms compared to placebo over a 2-week period. | Very low  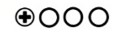 |
| **General food cravings** | Cross-sectional (Abbas and Liddle, 2013) | 60 [olanzapine group: 20; FGA group: 20/ unexposed group: 20]  (1) | Assessed using FCI. | Very serious:  Convenience sampling; failure to adequately control for confounding. | NA | Very serious:  Unexposed group were not representative of target population. | Not serious:  Rationale for sample size calculation provided for the study (Abbas and Liddle, 2013), narrow CI of effect estimates. | Food cravings were assessed in one cross-sectional study (Abbas and Liddle, 2013) using the FCI. It revealed higher general food, carbohydrates and high fats cravings among olanzapine- and FGA-treated patients with high baseline BMI compared to unexposed healthy participants. | Very low  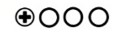 |
| **Hunger** | Cross-sectional (Blouin et al., 2008) | 38 [Exposed group: 18/Unexposed group: 20]  (1) | Assessed using VAS | Very serious:  Sampling strategy not explained; non-validated outcome measure. | NA | Not serious:  Unexposed group matched to the SGA-treated group for age, weekly physical activity levels. | Serious:  Small sample size. OIS could not be calculated. CI of effect estimate not reported (presented as graph) | The effect of SGAs on hunger and satiety were investigated in a laboratory setting in one cross-sectional study (Blouin et al., 2008) SGA-treated patients with high baseline BMI had higher hunger scores compared to the unexposed healthy participants. | Very low  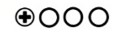 |
|  | RCT (Ballon et al., 2018; Teff et al., 2015) | 54 (2)  (Ballon et al., 2018): Sample size: 24  [olanzapine arm: 7;  iloperidone arm: 7;  placebo: 10]  (Teff et al., 2015):  Sample size: 30  [olanzapine arm: 10;  aripiprazole arm: 10;  Placebo: 10] | Assessed using VAS. | Very serious:  2 RCTs did not provide details of randomisation or concealment of allocation; used non-validated outcome measures. | Not serious:  Variation in results can be explained by type of antipsychotic studied. | Serious:  RCTs of healthy volunteers, not representative of target population. | Serious:  Small sample size. OIS could not be calculated. Wide CI of effect estimates in 1 study (Ballon et al., 2018); no data provided in 1 study (presented as graphs) (Teff et al., 2015). | 2 RCTs of healthy volunteers with normal baseline BMI (Ballon et al., 2018; Teff et al., 2015) measured hunger using VAS in laboratory setting; found higher pre-meal (Ballon et al., 2018) and total (Teff et al., 2015)hunger scores in olanzapine arm compared to placebo. A contradictory effect as reported in iloperidone (Ballon et al., 2018) and aripiprazole arms (Teff et al., 2015). | Low  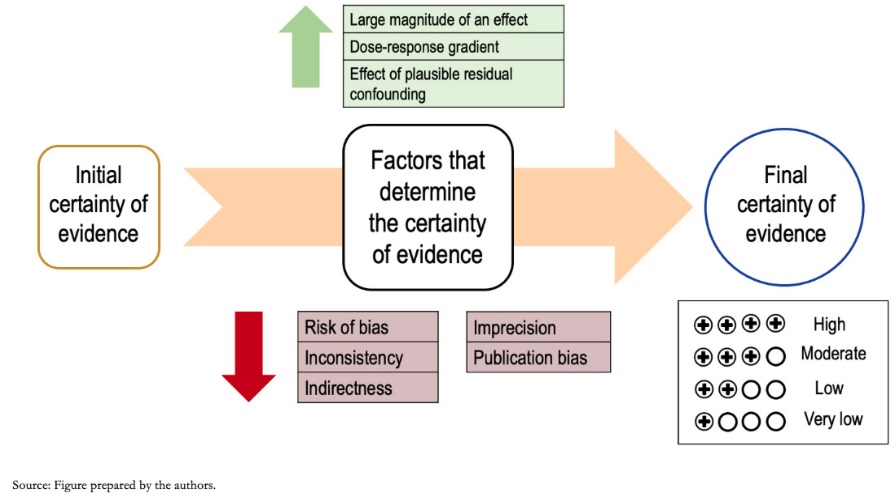 |
| **Energy intake** | Cross-sectional (Blouin et al., 2008; Henderson et al., 2006; Jakobsen et al., 2018b; Saugo et al., 2020; Stefanska et al., 2017; Stefanska et al., 2018) | 6941 (7)  (Blouin et al., 2008)  Sample size: 38  [Exposed group: 18; unexposed group: 20]  (Henderson et al., 2006)  Sample size: 811  [SGA-treated group: 88; unexposed group: 723]  (Jakobsen et al., 2018b)  Sample size: 3362  [Exposed group: 346/unexposed group: 3016]  (Saugo et al., 2020)  Sample size: 2367  [Exposed group: 54;  unexposed group: 2313]  (Stefanska et al., 2017)  Sample size: n= 158  [Exposed group: 60;  Unexposed group: 98]  (Stefanska et al., 2018)  Sample size: 155  [Exposed group: 85;  Unexposed group: 70]  (Nunes et al., 2014)  Sample size: 50  [Exposed group: 25;  Unexposed group: 25] | Different settings including lab (Blouin et al., 2008) and natural settings (Henderson et al., 2006; Jakobsen et al., 2018a; Nunes et al., 2014; Saugo et al., 2020; Stefanska et al., 2017; Stefanska et al., 2018), different assessment tools (dietary recall methods, EPIC Questionnaire) | Very serious:  Sampling strategy was not explained in 4 studies (Blouin et al., 2008; Henderson et al., 2006; Stefanska et al., 2017; Stefanska et al., 2018); different outcome measures; failure to adequately control for confounding in all 7 studies. | Very serious:  Variation in results may be partly attributed to baseline BMI, other potential confounders such as sex, AP medication, and treatment duration. | Very serious:  Unexposed groups in the 7 studies were not representative of target population. | Serious:  OIS could not be calculated. Wide 95% CIs of effect estimates across studies, except for (Jakobsen et al., 2018b; narrow 95% CI) and (Suago et al., 2020; CI not reported). | Antipsychotics had an inconsistent effect on total energy intake in patients with high baseline BMI (Blouin et al., 2008; Henderson et al., 2006; Jakobsen et al., 2018b; Nunes et al., 2014; Stefanska et al., 2017; Stefanska et al., 2018) and normal BMI (Saugo et al., 2020), compared to unexposed healthy participants. | Very low  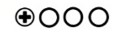 |
|  | RCT of healthy volunteers (Fountaine et al., 2010; Teff et al., 2013) | 60 (2)  (Fountaine et al., 2010)  Sample size: 30 (21 completers; randomised, placebo controlled 2-period crossover trial)  (Teff et al., 2013)  Sample size: 30  [olanzapine arm: 10; aripiprazole arm: 10;  placebo: 10] | Assessed by calculating energy intake in lab setting. | Not serious:  Did not provide details of randomisation (Teff et al., 2013) or concealment of allocation (Fountaine et al., 2010; Teff et al., 2013). | Not serious:  Results consistent. | Serious:  RCTs of healthy volunteers, not representative of target population. | Very serious:  OIS could not be calculated. 95% CI of effect estimates not reported; no data reported (presented as a graph) (Teff et al., 2013), missing data (Fountaine et al., 2010). | 2 RCTs of healthy volunteers with normal baseline BMI concluded that olanzapine-treated participants (for 15 and 12 days, respectively) consumed more calories (Fountaine et al., 2010; Teff et al., 2013), while aripiprazole-treated participants consumed less calories (Teff et al., 2013), than those in the placebo arm. | Very low  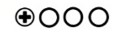 |
| **Eating disorders: BED** | Cross-sectional  (Khazaal et al., 2006a) | 80 (1)  [Stratified according to BMI: Exposed group: 40 (20 with BMI <28; 20 with BMI ≥28);  Unexposed group: 40 (20 with BMI <28; 20 with BMI ≥28) | Assessed using DSM-IV criteria | Very serious:  Convenience sample; BMI threshold determined post hoc. | NA | Serious:  Unexposed group was not representative of target population. | Very serious:  Small sample size OIS could not be calculated. Wide 95% CI of OR for sample with high BMI; OR could not be calculated for sample with normal BMI. | Second-generation antipsychotic-treated patients had higher odds of developing binge eating disorders (BED) compared to unexposed healthy participants (Khazaal et al., 2006a), regardless of baseline BMI. | Very low  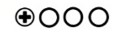 |
| **Eating cognitions: dietary restraint** | Cross-sectional | 455 (5)  (Blouin et al., 2008)  Sample size: 38  [Exposed group: 18/  Unexposed group: 20]  (Khazaal et al., 2009)  Sample size: 37  [Exposed group: 22 (10 with weight gain; 12 without weight gain);  Unexposed group: 15 (5 with weight gain; 10 without weight gain)]  (Khazaal et al., 2006b)  Sample size: 80 [(stratified according to BMI: Exposed group: 40 (20 with BMI <28; 20 with BMI ≥28); Unexposed group: 40 (20 with BMI <28; 20 with BMI ≥28)]  (Kouidrat et al., 2018)  Sample size: 147  [Exposed group: 66;  Unexposed group: 81]  (Sentissi et al., 2009)  Sample size: 153  [FGA-treated group: 27;  SGA-treated group: 93  Untreated group:33] | Assessed using TEFQ, TEFQ-R21, MAC-R | Vey serious:  Convenience sample (Khazaal et al., 2009; Khazaal et al., 2006b; Kouidrat et al., 2018; Sentissi et al., 2009) or sampling strategy was not explained (Blouin et al., 2008); BMI threshold determined post hoc (Khazaal et al., 2009; Khazaal et al., 2006b). | Not serious:  Overall. Most studies reported similar findings. | Serious:  Unexposed groups were not representative of target population, with the exception of the untreated group in Sentissi et al. (2009). | Serious:  OIS could not be calculated. Wide 95% CI of effect estimates in (Khazaal et al., 2009; Khazaal et al., 2006b; Sentissi et al., 2009); CI of effect estimate not reported in (Blouin et al., 2008; Kouidrat et al., 2018). | 5 of 6 samples of antipsychotic-treated patients with high baseline BMI (reported in 5 studies) (Blouin et al., 2008; Khazaal et al., 2009; Khazaal et al., 2006b; Kouidrat et al., 2018; Sentissi et al., 2009), and all (n=2/2) samples of antipsychotic-treated patients with normal baseline BMI (reported in 2 studies) (Khazaal et al., 2009; Khazaal et al., 2006b) reported higher restraint scores than unexposed groups. | Very low  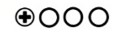 |
| **Eating cognitions: dietary disinhibition** | Cross-sectional | 228 (3)  (Blouin et al., 2008)  Sample size: 38  [Exposed group: 18/  Unexposed group: 20]  (Khazaal et al., 2009)  Sample size: 37  [Exposed group: 22 (10 with weight gain; 12 without weight gain);  Unexposed group: 15 (5 with weight gain; 10 without weight gain)]  (Sentissi et al., 2009)  Sample size: 153  [FGA-treated group: 27;  SGA-treated group: 93  Untreated group:33] | Assessed using TEFQ | Vey serious:  Convenience sample (Khazaal et al., 2009; Sentissi et al., 2009) or sampling strategy was not explained (Blouin et al., 2008); BMI threshold determined post hoc (Khazaal et al., 2009). | Not serious:  Overall. Most studies reported similar findings. | Serious:  Unexposed groups were not representative of target population, with the exception of the untreated group in Sentissi et al. (2009). | Serious:  OIS could not be calculated. 95% CI of effect estimates not reported (Blouin et al., 2008); wide 95% CI of effect estimates in (Khazaal et al., 2009; Sentissi et al., 2009). | Antipsychotics had an inconsistent effect on disinhibition scores among patients with high baseline BMI (Blouin et al., 2008; Khazaal et al., 2009; Sentissi et al., 2009), while having a positive effect among those with normal baseline BMI (Khazaal et al., 2009). | Very low  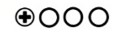 |
| **Eating cognitions: hunger** | Cross-sectional | 228 (3)  (Blouin et al., 2008)  Sample size: 38  [Exposed group: 18/  Unexposed group: 20]  (Khazaal et al., 2009)  Sample size: 37  [Exposed group: 22 (10 with weight gain; 12 without weight gain);  Unexposed group: 15 (5 with weight gain; 10 without weight gain)]  (Sentissi et al., 2009)  Sample size: 153  [FGA-treated group: 27;  SGA-treated group: 93  Untreated group:33] | Assessed using TEFQ | Vey serious:  Convenience sample (Khazaal et al., 2009; Sentissi et al., 2009) or sampling strategy was not explained (Blouin et al., 2008); BMI threshold determined post hoc (Khazaal et al., 2009). | Not serious:  Overall. Most studies reported similar findings. | Serious:  Unexposed groups were not representative of target population, with the exception of the untreated group in Sentissi et al. (2009). | Serious:  OIS could not be calculated. 95% CI of effect estimates not reported (Blouin et al., 2008); wide 95% CI of effect estimates reported in (Khazaal et al., 2009; Sentissi et al., 2009). | Antipsychotics had an inconsistent effect on hunger scores among patients with high baseline BMI (Blouin et al., 2008; Khazaal et al., 2009; Sentissi et al., 2009), while having a positive effect among those with normal baseline BMI (Khazaal et al., 2009). | Very low  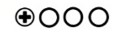 |

BMI= body mass index; CI= confidence interval; DSM-IV= Diagnostic and Statistical Manual of Mental Disorders, 4th Edition; EPIC Questionnaire= European Prospective Investigation into Cancer and Nutrition Questionnaire; FCI= Food Craving Inventory; FGA= first-generation antipsychotic; GRADE= Grading of Recommendations, Assessment, Development and Evaluation; MAC-R= Mizes Anorectic Cognitions questionnaire; NA= not applicable; OIS= optimal information size; OR= odds ratio; RCT= randomised controlled study; SGA= second-generation antipsychotic; TFEQ= Three-Factor Eating Questionnaire; TFEQ-R21= Three-Factor Eating Questionnaire Revised 21-item Version; VAS= visual analogue scale.

# References

Abbas, M. J. & Liddle, P. F. (2013). ‘Olanzapine and food craving: A case control study’ *Hum Psychopharmacol*, 28 (1), pp. 97-101. DOI: 10.1002/hup.2278 Available at: <https://www.ncbi.nlm.nih.gov/pubmed/23169487>.

Ballon, J. S., et al. (2018). ‘Pathophysiology of drug induced weight and metabolic effects: Findings from an rct in healthy volunteers treated with olanzapine, iloperidone, or placebo’ *J Psychopharmacol*, 32 (5), pp. 533-540. DOI: 10.1177/0269881118754708 Available at: <https://www.ncbi.nlm.nih.gov/pubmed/29444618>.

Blouin, M., et al. (2008). ‘Adiposity and eating behaviors in patients under second generation antipsychotics’ *Obesity (Silver Spring)*, 16 (8), pp. 1780-7. DOI: 10.1038/oby.2008.277 Available at: <https://www.ncbi.nlm.nih.gov/pubmed/18535555>.

Fountaine, R. J., et al. (2010). ‘Increased food intake and energy expenditure following administration of olanzapine to healthy men’ *Obesity (Silver Spring)*, 18 (8), pp. 1646-51. DOI: 10.1038/oby.2010.6 Available at: <https://www.ncbi.nlm.nih.gov/pubmed/20134408> (Accessed: 2023/06/22).

Garriga, M., et al. (2019). ‘Food craving and consumption evolution in patients starting treatment with clozapine’ *Psychopharmacology (Berl)*, 236 (11), pp. 3317-3327. DOI: 10.1007/s00213-019-05291-3 Available at: <https://www.ncbi.nlm.nih.gov/pubmed/31197435>.

Gothelf, D., et al. (2002). ‘Weight gain associated with increased food intake and low habitual activity levels in male adolescent schizophrenic inpatients treated with olanzapine’ *Am J Psychiatry*, 159 (6), pp. 1055-7. DOI: 10.1176/appi.ajp.159.6.1055 Available at: <https://www.ncbi.nlm.nih.gov/pubmed/12042200>.

Hardy, T., et al. 'Insulin sensitivity in patients with schizophrenia or schizoaffective disorder treated with olanzapine or risperidone', *162nd Annual Meeting Shaping our Future: Science and Service*, San Francisco: American Psychiatric Association, pp. 14-15.

Henderson, D. C., et al. (2006). ‘Dietary intake profile of patients with schizophrenia’ *Ann Clin Psychiatry*, 18 (2), pp. 99-105. DOI: 10.1080/10401230600614538 Available at: <https://www.ncbi.nlm.nih.gov/pubmed/16754415>.

Hoffman, V. P., Case, M. & Jacobson, J. G. 'Algorithms including amantadine, metformin and zonisamide for mitigation of weight gain during olanzapine treatment in outpatients with schizophrenia', *APA San Francisco*

Jakobsen, A. S., et al. (2018a). ‘Associations between clinical and psychosocial factors and metabolic and cardiovascular risk factors in overweight patients with schizophrenia spectrum disorders - baseline and two-years findings from the change trial’ *Schizophr Res*, 199 pp. 96-102. DOI: 10.1016/j.schres.2018.02.047 Available at: <https://doi.org/10.1016/j.schres.2018.02.047>.

Jakobsen, A. S., et al. (2018b). ‘Dietary patterns and physical activity in people with schizophrenia and increased waist circumference’ *Schizophr Res*, 199 pp. 109-115. DOI: 10.1016/j.schres.2018.03.016 Available at: <https://www.ncbi.nlm.nih.gov/pubmed/29555213>.

Kang, D., et al. (2024). ‘The effect of continuous theta burst stimulation on antipsychotic-induced weight gain in first-episode drug-naive individuals with schizophrenia: A double-blind, randomized, sham-controlled feasibility trial’ *Transl Psychiatry*, 14 (1), p. 61. DOI: 10.1038/s41398-024-02770-w Available at: <https://www.ncbi.nlm.nih.gov/pubmed/38272892>.

Karagianis, J., et al. (2009). ‘A randomized controlled trial of the effect of sublingual orally disintegrating olanzapine versus oral olanzapine on body mass index: The platypus study’ *Schizophr Res*, 113 (1), pp. 41-8. DOI: 10.1016/j.schres.2009.05.024 Available at: <https://www.ncbi.nlm.nih.gov/pubmed/19535229>.

Khazaal, Y., et al. (2009). ‘Hunger and negative alliesthesia to aspartame and sucrose in patients treated with antipsychotic drugs and controls’ *Eat Weight Disord*, 14 (4), pp. e225-30. DOI: 10.1007/BF03325121 Available at: <https://www.ncbi.nlm.nih.gov/pubmed/20179410>.

Khazaal, Y., Fresard, E., Borgeat, F. & Zullino, D. (2006a). ‘Binge eating symptomatology in overweight and obese patients with schizophrenia: A case control study’ *Ann Gen Psychiatry*, 5 p. 15. DOI: 10.1186/1744-859X-5-15 Available at: <https://www.ncbi.nlm.nih.gov/pubmed/16968528>.

Khazaal, Y., et al. (2006b). ‘Eating and weight related cognitions in people with schizophrenia : A case control study’ *Clin Pract Epidemiol Ment Health*, 2 p. 29. DOI: 10.1186/1745-0179-2-29 Available at: <https://www.ncbi.nlm.nih.gov/pubmed/17076886>.

Kouidrat, Y., et al. (2018). ‘Disordered eating behaviors as a potential obesogenic factor in schizophrenia’ *Psychiatry Res*, 269 pp. 450-454. DOI: 10.1016/j.psychres.2018.08.083 Available at: <https://www.ncbi.nlm.nih.gov/pubmed/30195737>.

Mathews, J., et al. (2012). ‘Neural correlates of weight gain with olanzapine’ *Arch Gen Psychiatry*, 69 (12), pp. 1226-37. DOI: 10.1001/archgenpsychiatry.2012.934 Available at: <https://www.ncbi.nlm.nih.gov/pubmed/22868896>.

Nunes, D., et al. (2014). ‘Nutritional status, food intake and cardiovascular disease risk in individuals with schizophrenia in southern brazil: A case-control study’ *Rev Psiquiatr Salud Ment*, 7 (2), pp. 72-9. DOI: 10.1016/j.rpsm.2013.07.001 Available at: <https://www.ncbi.nlm.nih.gov/pubmed/24054065>.

Park, S., Yi, K. K., Kim, M. S. & Hong, J. P. (2013). ‘Effects of ziprasidone and olanzapine on body composition and metabolic parameters: An open-label comparative pilot study’ *Behav Brain Funct*, 9 p. 27. DOI: 10.1186/1744-9081-9-27 Available at: <https://www.ncbi.nlm.nih.gov/pubmed/23866300>.

Roerig, J. L., et al. (2005). ‘A comparison of the effects of olanzapine and risperidone versus placebo on eating behaviors’ *J Clin Psychopharmacol*, 25 (5), pp. 413-8. DOI: 10.1097/01.jcp.0000177549.36585.29 Available at: <https://www.ncbi.nlm.nih.gov/pubmed/16160615>.

Saugo, E., et al. (2020). ‘Dietary habits and physical activity in first-episode psychosis patients treated in community services. Effect on early anthropometric and cardio-metabolic alterations’ *Schizophr Res*, 216 pp. 374-381. DOI: 10.1016/j.schres.2019.11.010 Available at: <https://www.ncbi.nlm.nih.gov/pubmed/31806524>.

Sentissi, O., et al. (2009). ‘Impact of antipsychotic treatments on the motivation to eat: Preliminary results in 153 schizophrenic patients’ *Int Clin Psychopharmacol*, 24 (5), pp. 257-64. DOI: 10.1097/YIC.0b013e32832b6bf6 Available at: <https://www.ncbi.nlm.nih.gov/pubmed/19606055>.

Smith, R. C., Rachakonda, S., Dwivedi, S. & Davis, J. M. (2012). ‘Olanzapine and risperidone effects on appetite and ghrelin in chronic schizophrenic patients’ *Psychiatry Res*, 199 (3), pp. 159-63. DOI: 10.1016/j.psychres.2012.03.011 Available at: <https://www.ncbi.nlm.nih.gov/pubmed/22475524>.

Stefanska, E., et al. (2017). ‘Eating habits and nutritional status of patients with affective disorders and schizophrenia’ *Psychiatr Pol*, 51 (6), pp. 1107-1120. DOI: 10.12740/PP/74558 Available at: <https://www.ncbi.nlm.nih.gov/pubmed/29432506>.

Stefanska, E., et al. (2018). ‘The assessment of the nutritional value of meals consumed by patients with recognized schizophrenia’ *Rocz Panstw Zakl Hig*, 69 (2), pp. 183-192. Available at: <https://www.ncbi.nlm.nih.gov/pubmed/29766697>.

Stip, E., et al. (2012). ‘Neural changes associated with appetite information processing in schizophrenic patients after 16 weeks of olanzapine treatment’ *Transl Psychiatry*, 2 (6), p. e128. DOI: 10.1038/tp.2012.53 Available at: <https://www.ncbi.nlm.nih.gov/pubmed/22714121>.

Teff, K. L., Rickels, K., Alshehabi, E. & Rickels, M. R. (2015). ‘Metabolic impairments precede changes in hunger and food intake following short-term administration of second-generation antipsychotics’ *J Clin Psychopharmacol*, 35 (5), pp. 579-82. DOI: 10.1097/JCP.0000000000000393 Available at: <https://www.ncbi.nlm.nih.gov/pubmed/26274045>.

Teff, K. L., et al. (2013). ‘Antipsychotic-induced insulin resistance and postprandial hormonal dysregulation independent of weight gain or psychiatric disease’ *Diabetes*, 62 (9), pp. 3232-40. DOI: 10.2337/db13-0430 Available at: <https://www.ncbi.nlm.nih.gov/pubmed/23835329> (Accessed: 5/1/2023).

Treuer, T., et al. (2009). ‘Factors associated with weight gain during olanzapine treatment in patients with schizophrenia or bipolar disorder: Results from a six-month prospective, multinational, observational study’ *World J Biol Psychiatry*, 10 (4 Pt 3), pp. 729-40. DOI: 10.1080/15622970903079507 Available at: <https://www.ncbi.nlm.nih.gov/pubmed/19606406>.
