# Supplementary material for: The impacts of antipsychotic medications on eating-related outcomes: A mixed methods systematic review
Source: PLoS One. 2025 Feb 3;20(2):e0308037. doi: 10.1371/journal.pone.0308037 (PMC11790239; doi:10.1371/journal.pone.0308037)
Supplement: S11 File — (DOCX) [file pone.0308037.s011.docx]

**S11 File. Study and participant characteristics of the 6 included qualitative studies.**

| **Citation** | **Country** | **Study characteristics** | | | | | **Data collection methods** | **Data collection tools** | **Data analysis** | **Funding** |
| --- | --- | --- | --- | --- | --- | --- | --- | --- | --- | --- |
|  |  | **Aim(s)** | **Methodology/ epistemology** | **Situational context** | **Study population** | **Recruitment/ sampling** | **Data collection method** | **Interview guide** |  |  |
| (Haracz et al., 2018) | Australia | To understand the perspectives of women with schizophrenia spectrum disorders (SSD) regarding their experience of weight gain, factors contributing to it, and its impact on them. | Constructivist grounded theory | Participants were recruited from community-based mental health services in 1 area health service in New South Wales, Australia, via staff in those services. | **Sample size: n= 11**  **Study population:**  Women diagnosed with SSD.  **Age (years), mean (range):** 36, (24-61)  **Sex, male n (%):** 0  **Baseline BMI (Kg/m^2^), mean (SD):** NR  **AP category, duration of treatment:** NR | Purposive followed by maximum variation sampling to include women with a range of characteristics including ‘age, time since diagnosis, educational level, living alone or with others, and whether they were working, studying, or neither.’  Preliminary analysis of data was followed by theoretical sampling, adaptation of topic guide, and revisiting earlier participants with new questions. | 24 individual, semi-structured interviews with 11 participants.  Participants engaged in 1-3 interviews over a 2-year period. | Topic guide questions:  Participants’ interests, how they spent their days, whether they worked, and who they lived with, their views on what constituted health, their experiences regarding the onset and course of schizophrenia, their beliefs about how schizophrenia affected their health, their beliefs about weight and the impact of schizophrenia on weight. | Grounded constructivist approach (Charmaz, 2000) | Funding: not stated  Conflicts: none |
| (Kaar et al., 2019) | UK | To explore patients’ experiences of antipsychotic medication decision-making in order to develop a patient  decision aid that promotes shared decision-making. | Qualitative descriptive | Participants were current mental health patients within the South London and Maudsley NHS Foundation Trust,  recruited through local  mental health teams. | **Sample size: n= 23**  **Study population, n (%):**  Patients diagnosed with a psychotic disorder and taking APs.  Schizophrenia 20 (87), schizoaffective disorder 2 (9), bipolar II disorder 1 (4)  Diagnostic criteria: ICD-10 criteria.  **Age (years), mean (SD), range:** 43 (10) 25–58  **Sex, male n (%):** 16 (70)  **Ethnicity, n (%):** White British 11 (48), Caribbean British 7 (30), Mixed British 1 (5), Other 4 (17)  **Baseline BMI (Kg/m^2^), mean (SD):** NR  **AP category, n (%):** Clozapine 11 (47), paliperidone 4 (17), risperidone 3 (13), aripiprazole 2 (9), olanzapine 1 (4), quetiapine 1 (4), chlorpromazine 1 (4), amisulpride 1 (4),  **Use of other medications:** Citalopram 2 (8)  **AP duration of treatment:** NR | Purposive | 4 focus groups with a range of 3–6 participants each.  Three 1:1 think aloud sessions were conducted with 3 participants to give feedback on the draft decision aid. | FGDs topic guide: benefits, adverse  effects, methods of administration, physical health monitoring, who was involved in decision-making process, emotional and practical aspects of these experiences.  Example questions: ‘Looking back on the antipsychotic medication you have received in the past, what comes to mind? Is there anything you wish you knew about an antipsychotic medication before you started taking it? Are there any particular experiences with medication that stay with you?’  Think aloud sessions:  Participants were asked to use and verbalise their thoughts about a draft of the decision aid. | Thematic analysis (Braun and Clarke, 2006) | Non-pharmaceutical funding  Conflicts: partly |
| (Teferra et al., 2013) | Ethiopia | To explore reasons for low adherence to medication in a rural Ethiopian setting from the perspectives of people with schizophrenia, their caregivers, research field workers and health workers. | Qualitative study  nested within a cohort study, 'Course and Outcome of Schizophrenia and Bipolar Disorder in Butajira' (Kebede et al., 2003) | Participants were recruited from a psychiatric nurse-led outpatient unit located in a general hospital in rural Ethiopia. | **Sample size: n= 51**  **Study population:**   - Persons diagnosed with schizophrenia (n= 24):   Diagnostic criteria: o ICD-10/DSM-IV  Age (years), range: 15-49  Sex, male n (%): NR  Baseline BMI (Kg/m^2^), mean (SD): NR  AP category: FGA  Duration of treatment: NR   - their caregivers (n= 19), - mental health research field workers who provide community outreach to patients (n= 7), - psychiatric nurse (n= 1) | Purposive sampling of patients stratified according to adherence to AP treatment.  Non-adherence was defined as ‘failing to take prescribed AP in the previous month as per patient and family member reports. Health professionals considered patients missing their appointments as non-adherent.’ | 6 FGDs (6-8 participants) and 9 individual, in-depth interviews:   - 3 FGDs, 4 interviews with patients, - 2 FGDs, 4 interviews with caregivers, - 1 FGD with mental health research field workers - 1 interview with a psychiatric nurse in the Butajira hospital psychiatric clinic. | Topic guide questions:  Explanatory models of illness (including symptoms, causes, course and appropriate interventions); experience of being treated with APs; perceived reasons for non-adherence; impact of substance use; nature of the interaction with mental health workers upon adherence.  N.B.: Interviews and FGDs were conducted by the head of the research project site, who was also the psychiatrist involved in treating patients and working with psychiatric nurses at the site. | Thematic analysis (Braun and Clarke, 2006) | Non-pharmaceutical funding  Conflicts: none |
| (Usher et al., 2013) | Australia | To describe experience of weight gain associated with SGAs from the perspective of patients. | Phenomenological qualitative study | Participants living independently in the community, recruited from local mental health consumer group organisations. | **Sample size: n= 8**  **Study population:**  Patients diagnosed with schizophrenia; prescribed SGAs or a combination of SGAs and FGAs; self-identified as having gained weight because of SGAs.  **Age (years), range:** 18-60  **Sex, male n (%):** 1 (12.5)  **Baseline BMI (Kg/m^2^), mean (SD):** NR  **AP category, duration of treatment:** NR | Purposive | 8 individual, semi-structured interviews. | Topic guide questions:  ‘What has it been like to gain weight as a result of taking SGAs? Can you share some of your experiences trying to manage the associated weight gain? Has the weight gain changed the way people interact with you? Has it has affected the way you feel about yourself?’ | Phenomenological analysis (Van Manen, 1990) | Funding: not stated  Conflicts: none |
| (Vandyk and Baker, 2012) | Canada | To explore  the experience of living with mental illness, attitudes towards weight and its management from the perspectives of people with schizophrenia. | Qualitative study set within the constructivist  paradigm | Participants recruited  from a psychiatric outpatient clinic which provides education sessions on healthy eating habits and cooking techniques; access to an exercise room and  gymnasium; a walking group to provide a lower-intensity exercise option.  Involvement in these activities was optional. | **Sample size: n= 18**  **Study population, n (%):**  Schizophrenia 10 (56)  Schizoaffective disorder 8 (44)  DSM-IV diagnostic criteria  **Age (years), mean (range):** 45 (31-64)  **Sex, male n (%):**15 (83)  **Baseline BMI (Kg/m^2^), mean (SD):** NR  **AP category:**  All participants were taking SGAs, 10 participants were prescribed FGAs (currently or in the past).  **AP duration of treatment:** NR  **Use of other medications, n (%):**  Mood stabilisers 7 (39) | Purposive | 18 individual, semi-structured interviews. | Examples of topic guide questions:  What do you think about your weight  right now? What, if anything, influences your weight? | Inductive, descriptive approach using constant comparison | Funding: not stated  Conflicts: not stated |
| (Xiao et al., 2012) | Canada | to generate a theory of the psychosocial processes influencing weight management among persons newly prescribed SGAs. | Constructivist grounded theory | Participants were recruited from an early intervention programme (EIP) outpatient clinic | **Sample size: n= 16**  **Study population:**  Persons diagnosed with FEP who had been prescribed SGAs for at least 8 weeks (n= 11), persons diagnosed with chronic schizophrenia who had been prescribed SGAs for at least 3 years (n= 5)  **Age (years), n (%):**  18-23 years: 11 (68.7),  24–29 years: 4 (25),  ≥30 years: 1 (6.2)  **Sex, male n (%):** 14 (87.5)  **Baseline BMI (Kg/m^2^), mean (SD):** NR  **Duration of AP treatment, n of participants:** < 3 years (n= 11), > 3 years (n= 5)  **APs:** olanzapine (n= 5), risperidone (n= 4)**,** other SGAs (n= 7) | Theoretical sampling followed by purposive sampling to actively recruit more female participants. | 16 individual, in-depth interviews | Unstructured interview guide | Comparative analysis (Strauss and Corbin, 1998) | Pharmaceutical funding (partly)  Conflicts: not stated |

AP=antipsychotic, BMI= body mass index; DSM-IV= Diagnostic and Statistical Manual of Mental Disorders, fourth edition; EIP= early intervention programme; FEP= first-episode psychosis; FGA= first-generation antipsychotic; FGD= focus group discussion; ICD-10= International Classification of Diseases 10^th^ Revision; N= number; SD= standard deviation; SGA= second-generation antipsychotics

**References**

Braun, V. & Clarke, V. (2006). ‘Using thematic analysis in psychology’ *Qualitative Research in Psychology*, 3 (2), pp. 77-101. DOI: 10.1191/1478088706qp063oa Available at: <https://doi.org/10.1191/1478088706qp063oa>.

Charmaz, K. (2000). 'Grounded theory: Objectivist and constructivist methods', in Denzin, N. & Lincoln, Y. (eds.) *Handbook of qualitative research.* Second edn. Thousand Oaks, CA: SAGE publications, pp. 509-535.

Haracz, K., Hazelton, M. & James, C. (2018). ‘The "double whammy": Women's experiences of weight gain after diagnosis and treatment for schizophrenia spectrum disorders’ *J Nerv Ment Dis*, 206 (5), pp. 303-309. DOI: 10.1097/NMD.0000000000000803 Available at: <https://www.ncbi.nlm.nih.gov/pubmed/29528882>.

Hong, Q. N., et al. (2018). *Mixed methods appraisal tool (mmat), version 2018.* . Canada: IC Canadian Intellectual Property Office. Available at: <http://mixedmethodsappraisaltoolpublic.pbworks.com/w/file/fetch/127916259/MMAT_2018_criteria-manual_2018-08-01_ENG.pdf> (Accessed: 24 February 2023).

Kaar, S. J., et al. (2019). ‘Making decisions about antipsychotics: A qualitative study of patient experience and the development of a decision aid’ *BMC Psychiatry*, 19 (1), p. 309. DOI: 10.1186/s12888-019-2304-3 Available at: <https://www.ncbi.nlm.nih.gov/pubmed/31646985>.

Kebede, D., et al. (2003). ‘Onset and clinical course of schizophrenia in butajira-ethiopia--a community-based study’ *Soc Psychiatry Psychiatr Epidemiol*, 38 (11), pp. 625-31. DOI: 10.1007/s00127-003-0678-4 Available at: <https://www.ncbi.nlm.nih.gov/pubmed/14614550>.

Strauss, A. & Corbin, J. (1998). *Basics of qualitative research: Techniques and procedures for developing grounded theory, 2nd ed*. *Basics of qualitative research: Techniques and procedures for developing grounded theory, 2nd ed.* Thousand Oaks, CA, US: Sage Publications, Inc.

Teferra, S., et al. (2013). ‘Perspectives on reasons for non-adherence to medication in persons with schizophrenia in ethiopia: A qualitative study of patients, caregivers and health workers’ *BMC Psychiatry*, 13 p. 168. DOI: 10.1186/1471-244X-13-168 Available at: <https://www.ncbi.nlm.nih.gov/pubmed/23773362>.

Usher, K., Park, T. & Foster, K. (2013). ‘The experience of weight gain as a result of taking second-generation antipsychotic medications: The mental health consumer perspective’ *J Psychiatr Ment Health Nurs*, 20 (9), pp. 801-6. DOI: 10.1111/jpm.12019 Available at: <https://www.ncbi.nlm.nih.gov/pubmed/23146024>.

Van Manen, M. (1990). *Researching lived experience: Human science for an action sensitive pedagogy*. Althouse, Ontario.

Vandyk, A. D. & Baker, C. (2012). ‘Qualitative descriptive study exploring schizophrenia and the everyday effect of medication-induced weight gain’ *Int J Ment Health Nurs*, 21 (4), pp. 349-57. DOI: 10.1111/j.1447-0349.2011.00790.x Available at: <https://www.ncbi.nlm.nih.gov/pubmed/22404848> (Accessed: 2023/08/16).

Xiao, S., Baker, C. & Oyewumi, L. K. (2012). ‘Psychosocial processes influencing weight management among persons newly prescribed atypical antipsychotic medications’ *J Psychiatr Ment Health Nurs*, 19 (3), pp. 241-7. DOI: 10.1111/j.1365-2850.2011.01773.x Available at: <https://www.ncbi.nlm.nih.gov/pubmed/22074295>.
