# Supplementary material for: The impacts of antipsychotic medications on eating-related outcomes: A mixed methods systematic review
Source: PLoS One. 2025 Feb 3;20(2):e0308037. doi: 10.1371/journal.pone.0308037 (PMC11790239; doi:10.1371/journal.pone.0308037)
Supplement: S12 File — (DOCX) [file pone.0308037.s012.docx]

**S12 File. Risk of bias assessments of the 6 included qualitative studies using the Mixed Methods Appraisal Tool (MMAT).**

| **CITATION** | **SCREENING QUESTIONS** | | **QUALITATIVE STUDIES** | | | | |
| --- | --- | --- | --- | --- | --- | --- | --- |
|  | S1. Are there clear research questions? | S2. Do the collected data allow to address the research questions? | Is the qualitative approach appropriate to answer the research question? | Are the qualitative data collection methods adequate to address the research question? | Are the findings adequately derived from the data? | Is the interpretation of results sufficiently substantiated by data? | Is there coherence between qualitative data sources, collection, analysis and interpretation? |
| (Haracz et al., 2018) | Yes | Yes | Yes | Yes | Yes | Yes | Yes |
| (Kaar et al., 2019) | Yes | Yes | Yes | Yes | Yes | Can’t tell (limited data to support interpretation of results) | Yes |
| (Teferra et al., 2013) | Yes | Yes | Yes | Yes | Yes | Can’t tell (limited data to support interpretation of results) | Yes |
| (Usher et al., 2013) | Yes | Yes | Yes | Yes | Yes | Yes | Yes |
| (Vandyk and Baker, 2012) | Yes | Yes | Yes | Yes | Yes | Can’t tell (limited data to support interpretation of results) | Yes |
| (Xiao et al., 2012) | Yes | Yes | Yes | Yes | Yes | Yes | Yes |

MMAT= Mixed Methods Appraisal Tool, version 2018 (Hong et al., 2018)

**References**

Haracz, K., Hazelton, M. & James, C. (2018). ‘The "double whammy": Women's experiences of weight gain after diagnosis and treatment for schizophrenia spectrum disorders’ *J Nerv Ment Dis*, 206 (5), pp. 303-309. DOI: 10.1097/NMD.0000000000000803 Available at: <https://www.ncbi.nlm.nih.gov/pubmed/29528882>.

Hong, Q. N., et al. (2018). *Mixed methods appraisal tool (mmat), version 2018.* . Canada: IC Canadian Intellectual Property Office. Available at: <http://mixedmethodsappraisaltoolpublic.pbworks.com/w/file/fetch/127916259/MMAT_2018_criteria-manual_2018-08-01_ENG.pdf> (Accessed: 24 February 2023).

Kaar, S. J., et al. (2019). ‘Making decisions about antipsychotics: A qualitative study of patient experience and the development of a decision aid’ *BMC Psychiatry*, 19 (1), p. 309. DOI: 10.1186/s12888-019-2304-3 Available at: <https://www.ncbi.nlm.nih.gov/pubmed/31646985>.

Teferra, S., et al. (2013). ‘Perspectives on reasons for non-adherence to medication in persons with schizophrenia in ethiopia: A qualitative study of patients, caregivers and health workers’ *BMC Psychiatry*, 13 p. 168. DOI: 10.1186/1471-244X-13-168 Available at: <https://www.ncbi.nlm.nih.gov/pubmed/23773362>.

Usher, K., Park, T. & Foster, K. (2013). ‘The experience of weight gain as a result of taking second-generation antipsychotic medications: The mental health consumer perspective’ *J Psychiatr Ment Health Nurs*, 20 (9), pp. 801-6. DOI: 10.1111/jpm.12019 Available at: <https://www.ncbi.nlm.nih.gov/pubmed/23146024>.

Vandyk, A. D. & Baker, C. (2012). ‘Qualitative descriptive study exploring schizophrenia and the everyday effect of medication-induced weight gain’ *Int J Ment Health Nurs*, 21 (4), pp. 349-57. DOI: 10.1111/j.1447-0349.2011.00790.x Available at: <https://www.ncbi.nlm.nih.gov/pubmed/22404848> (Accessed: 2023/08/16).

Xiao, S., Baker, C. & Oyewumi, L. K. (2012). ‘Psychosocial processes influencing weight management among persons newly prescribed atypical antipsychotic medications’ *J Psychiatr Ment Health Nurs*, 19 (3), pp. 241-7. DOI: 10.1111/j.1365-2850.2011.01773.x Available at: <https://www.ncbi.nlm.nih.gov/pubmed/22074295>.
