# Supplementary material for: The impacts of antipsychotic medications on eating-related outcomes: A mixed methods systematic review
Source: PLoS One. 2025 Feb 3;20(2):e0308037. doi: 10.1371/journal.pone.0308037 (PMC11790239; doi:10.1371/journal.pone.0308037)
Supplement: S13 File — (DOCX) [file pone.0308037.s013.docx]

**S13 File. The** **GRADE-CERQual assessment of confidence in qualitative evidence**.

**Evidence profile**

**Question: What is the effect of antipsychotic medications on eating behaviours?**

| **Summarised review finding** | **References** | **Methodological limitations** | **Coherence** | **Adequacy** | **Relevance** | **GRADE-CERQual assessment of confidence in the evidence** |
| --- | --- | --- | --- | --- | --- | --- |
| **Range of experiences:**  Most participants experienced an increase in appetite, hunger and low satiety.  Only one study mentioned that some participants experienced a decline in appetite (Kaar et al., 2019).  In settings in which food was available, an increase in appetite was translated into an increase in the frequency and quantity of food consumed.  Participants attributed these changes in eating behaviour to antipsychotic medications and reported that they were *‘unprepared for the hunger they experienced’.* | (Haracz et al., 2018; Kaar et al., 2019; Teferra et al., 2013; Usher et al., 2013; Vandyk and Baker, 2012; Xiao et al., 2012) | **Moderate concerns:**  Five studies (Kaar et al., 2019; Teferra et al., 2013; Usher et al., 2013; Vandyk and Baker, 2012; Xiao et al., 2012) were assessed as having methodological limitations related to researcher reflexivity - the researcher’s unclear or dual role both as a researcher and a healthcare provider – which may have potentially had an impact on what participants’ reported during the interviews.  Two studies (Teferra et al., 2013; Vandyk and Baker, 2012) provided limited or no quotes from patients to support the interpretation of the results.  Only one study (Haracz et al., 2018) used member checking to ensure the credibility of the findings. One study (Vandyk and Baker, 2012) mentioned member checking but did not provide further information. | **Minor concerns:**  Descriptive review finding- The data were varied, as 2 opposing effects on appetite were reported. The studies usually addressed the effect of antipsychotics on appetite in passing, and did not often explore in detail what participants meant by increased appetite and why they believed antipsychotic medications caused these effects. | **Moderate concerns:**  Descriptive review finding-  6 studies contributed to this finding, of which 3 (Kaar et al., 2019; Teferra et al., 2013; Vandyk and Baker, 2012) had relatively thin data. Opposing experiences were not further explored.  Only 1 study (Xiao et al., 2012) mentioned that participants were unprepared for these changes. | **Minor concerns:**  Review finding is directly relevant to the review question; 5 studies conducted in Western countries (food affordability issue) and 1 study in a non-Western country (food availability issue).  One study (Haracz et al., 2018) included only women. | **Very low:**  Moderate concerns regarding methodological limitations and adequacy of data; minor concerns regarding coherence and relevance of data. |
| **Onset of experiences:**  These experiences were believed to occur shortly after the introduction of antipsychotics. | (Usher et al., 2013; Vandyk and Baker, 2012) | **Moderate concerns:**  2 studies were assessed as having methodological limitations related to researcher reflexivity (the researcher’s unclear role with regards to their relationship with patients).  2 studies provided limited quotes to support the interpretation of the results.  1 study (Vandyk and Baker, 2012) mentioned member checking but did not provide further information. | **Minor concerns:**  Descriptive review finding- The data did not vary across both studies. However, the studies addressed this issue in passing, and did not explore in detail the onset of the reported experiences. | **Moderate concerns:**  Descriptive review finding-  Only 2 studies contributed to this finding and both had relatively thin data. | **Minor concerns:**  Review finding is partially relevant to the review question. 2 studies conducted in Western countries. | **Very low:**  Moderate concerns regarding methodological limitations and adequacy of data; minor concerns regarding coherence and relevance of data. |
| **Intensity of experiences:**  Strong word descriptors were used to portray the intensity of increased appetite and hunger. | (Haracz et al., 2018; Teferra et al., 2013; Usher et al., 2013; Xiao et al., 2012) | **Minor concerns:**  3 studies (Teferra et al., 2013) (Usher et al., 2013; Xiao et al., 2012) were assessed as having methodological limitations related to researcher reflexivity - the researcher’s unclear or dual role both as a researcher and a healthcare provider – which may have potentially had an impact on what participants’ reported during the interviews.  Only 1 study (Haracz et al., 2018) used member checking to ensure the credibility of the findings. | **Minor concerns:**  Descriptive review finding- The data did not vary across the studies. However, with the exception of study (Haracz et al., 2018), the studies explored changes in appetite and hunger in passing as this was not their primary objective. Participants were not asked about alternative explanations for increased appetite. | **Moderate concerns:**  Descriptive review finding-  Only 4 studies contributed to this finding and had relatively thin data. | **Minor concerns:**  Review finding is directly relevant to the review question; 3 studies conducted in Western countries (food affordability issue) and 1 study in a non-Western country (food availability issue). | **Low:**  Moderate concerns regarding adequacy of data; minor concerns regarding methodological limitations, coherence and relevance of data. |
| **Individual factor- beliefs about the role of food in negating side effects of antipsychotic medications:**  In the setting with limited food availability, participants (both patients and caregivers) believed that consuming ‘*good’* food was necessary prior to taking the ‘*strong’* antipsychotic medications that rendered their bodies weak. The authors suggested that adequate nutrition was believed by patients to counterbalance the adverse effects of these medications. This increased the likelihood of non-adherence among patients who were already burdened with limited food availability. | (Teferra et al., 2013) | **Minor concerns:**  This study was assessed as having methodological limitations related to researcher reflexivity - the researcher’s dual role both as a researcher and a healthcare provider – which may have potentially had an impact on what participants’ reported during the interviews.  No measures of credibility were discussed. | **No concerns:**  Evidence was derived from 1 study only. | **Serious concerns:**  Explanatory review finding-  Only 1 study contributed to this finding. Although it had relatively rich data, the phenomenon described was unexpected and specific to settings with food shortages. Based on an overall assessment of the richness of the data and the quantity of the data, we concluded that we had moderate concerns about data adequacy. | **Minor concerns:**  Review finding is partially relevant to the review question. Only 1 study in a non-Western country (food availability issue). | **Very low:**  Serious concerns regarding adequacy of data; minor concerns regarding methodological limitations and relevance of data; no concerns regarding coherence of data. |
| **Interpersonal factor- family reactions:**  In the setting with limited food availability, an increase in a patient’s appetite resulted in one of two outcomes based on their family’s reaction. Patients were denied access to more food when their families prioritised food access for healthy members who could secure livelihoods. Those patients experienced intolerable hunger which motivated them and their families to stop the medications. Families that resigned to their family member’s increased desire to eat despite limited food availability, were burdened by their decision.  In settings where food was available, an increase in appetite was translated into an increase in food consumption. The reactions of a patient’s family to their increased appetite and food consumption was confined to perceived stigma that drove patients to conceal the amount of food they consumed. | (Teferra et al., 2013; Usher et al., 2013) | **Minor concerns:**  The 2 studies (Teferra et al., 2013; Usher et al., 2013) were assessed as having methodological limitations related to researcher reflexivity - the researcher’s unclear or dual role both as a researcher and a healthcare provider – which may have potentially had an impact on what participants’ reported during the interviews. | **Serious concerns:**  Explanatory review finding – This review finding was not well-supported by the included studies.  Data from settings with limited food availability were extracted from 1 study only (Teferra et al., 2013). Data from studies where food was available were extracted from 1 study only (Usher et al., 2013) which included a quote from a participant regarding their family’s reactions to increased food consumption. This incomplete support for all aspects of this review finding have raised concerns about the coherence of the finding. | **Serious concerns:**  Explanatory review finding-  The study conducted in a setting where food was available (Usher et al., 2013) had relatively thin data. The study conducted in a setting with limited food availability (Teferra et al., 2013) had relatively thick data but the phenomenon described was unexpected and specific to this setting. Based on an overall assessment of the richness of the data and the quantity of the data, we concluded that we had serious concerns about data adequacy. | **Minor concerns:**  Review finding is directly relevant to the review question; One study was conducted in a Western country (possible food affordability issue) and 1 study in a non-Western country (food availability issue). One study (Teferra et al., 2013) included views of patients, their family members and psychiatric nurse. | **Very low:**  Serious concerns regarding coherence and adequacy of data; minor concerns regarding methodological limitations and relevance of data. |
| **External factor - Food environment:**  In settings where food was available, participants described how food prices (limited affordability) prohibited them from buying good quality food.  In settings with limited food availability, participants described how food shortages (limited availability) interacted with family reactions and priorities to determine whether increased appetite was translated into increased food intake. | (Teferra et al., 2013; Vandyk and Baker, 2012; Xiao et al., 2012) | **Minor concerns:**  3 studies were assessed as having methodological limitations related to researcher reflexivity (the researcher’s unclear role with regards to their relationship with patients).  Only 1 study (Vandyk and Baker, 2012) mentioned member checking but did not provide further information. | **Moderate concerns:**  Explanatory review finding – This review finding reflects the variation in the extracted data as per study setting. While the link between food affordability and the type of food consumed was supported by 2 studies, the link between food shortages and family reactions was supported by only 1 study. This incomplete support for all aspects of this review finding have raised concerns about the coherence of the finding. | **Serious concerns:**  Explanatory review finding-  2 studies were homogeneous with regards to setting (food availability), 1 (Vandyk and Baker, 2012) of which had relatively thin data. One study (Teferra et al., 2013) had relatively thick data but the phenomenon described was unexpected and specific to a setting with food shortages. Based on an overall assessment of the richness of the data and the quantity of the data, we concluded that we had serious concerns about data adequacy. | **Minor concerns:**  Review finding is directly relevant to the review question; 2 studies conducted in Western countries (food affordability issue) and 1 study in a non-Western country (food availability issue). One study (Teferra et al., 2013) included views of patients, their family members and psychiatric nurse. | **Very low:**  Serious concerns regarding adequacy of data; moderate concerns regarding coherence of data; minor concerns regarding methodological limitations and relevance of data. |

GRADE-CERQual= Grading of Recommendations, Assessment, Development and Evaluation-Confidence in the Evidence from Reviews of Qualitative research.

**References**

Haracz, K., Hazelton, M. & James, C. (2018). ‘The "double whammy": Women's experiences of weight gain after diagnosis and treatment for schizophrenia spectrum disorders’ *J Nerv Ment Dis*, 206 (5), pp. 303-309. DOI: 10.1097/NMD.0000000000000803 Available at: <https://www.ncbi.nlm.nih.gov/pubmed/29528882>.

Kaar, S. J., et al. (2019). ‘Making decisions about antipsychotics: A qualitative study of patient experience and the development of a decision aid’ *BMC Psychiatry*, 19 (1), p. 309. DOI: 10.1186/s12888-019-2304-3 Available at: <https://www.ncbi.nlm.nih.gov/pubmed/31646985>.

Teferra, S., et al. (2013). ‘Perspectives on reasons for non-adherence to medication in persons with schizophrenia in ethiopia: A qualitative study of patients, caregivers and health workers’ *BMC Psychiatry*, 13 p. 168. DOI: 10.1186/1471-244X-13-168 Available at: <https://www.ncbi.nlm.nih.gov/pubmed/23773362>.

Usher, K., Park, T. & Foster, K. (2013). ‘The experience of weight gain as a result of taking second-generation antipsychotic medications: The mental health consumer perspective’ *J Psychiatr Ment Health Nurs*, 20 (9), pp. 801-6. DOI: 10.1111/jpm.12019 Available at: <https://www.ncbi.nlm.nih.gov/pubmed/23146024>.

Vandyk, A. D. & Baker, C. (2012). ‘Qualitative descriptive study exploring schizophrenia and the everyday effect of medication-induced weight gain’ *Int J Ment Health Nurs*, 21 (4), pp. 349-57. DOI: 10.1111/j.1447-0349.2011.00790.x Available at: <https://www.ncbi.nlm.nih.gov/pubmed/22404848> (Accessed: 2023/08/16).

Xiao, S., Baker, C. & Oyewumi, L. K. (2012). ‘Psychosocial processes influencing weight management among persons newly prescribed atypical antipsychotic medications’ *J Psychiatr Ment Health Nurs*, 19 (3), pp. 241-7. DOI: 10.1111/j.1365-2850.2011.01773.x Available at: <https://www.ncbi.nlm.nih.gov/pubmed/22074295>.
