# Supplementary material for: The impacts of antipsychotic medications on eating-related outcomes: A mixed methods systematic review
Source: PLoS One. 2025 Feb 3;20(2):e0308037. doi: 10.1371/journal.pone.0308037 (PMC11790239; doi:10.1371/journal.pone.0308037)
Supplement: Attachment — Submitted filename: Response to Reviewers.docx [file pone.0308037.s014.docx]

**Manuscript Number: PONE-D-24-29459**

**The impacts of antipsychotic medications on eating experiences and eating behaviours: A mixed methods systematic review**

**Response to Reviewers**

Dear Dr Olashore,

Thank you for giving us the opportunity to submit a revised version of the manuscript “The impacts of antipsychotic medications on eating experiences and eating behaviours: A mixed methods systematic review” for publication in PLOS ONE.

We appreciate the time and effort that you and the reviewers dedicated in providing constructive feedback on our manuscript and are grateful for the insightful comments and valuable improvements to our paper.

We have incorporated the suggested revisions and amended the manuscript accordingly. The amendments are highlighted (in yellow) within the revised manuscript file with tracked changes (file name: Revised Manuscript with Track Changes). An unmarked version of the revised manuscript, without tracked changes, has also been submitted (file name: Manuscript).

Please see below, in blue, for a response to each point raised by the Academic Editor and Reviewers. Amended text is highlighted in yellow. All page, paragraph and line numbers refer to the revised manuscript file with tracked changes (file name: Revised Manuscript with Track Changes).

**Academic Editor Comments:**

1. Please ensure that your manuscript meets PLOS ONE's style requirements, including those for file naming.

<https://journals.plos.org/plosone/s/file?id=wjVg/PLOSOne_formatting_sample_main_body.pdf> [journals.plos.org] and

<https://journals.plos.org/plosone/s/file?id=ba62/PLOSOne_formatting_sample_title_authors_affiliations.pdf> [track.editorialmanager.com]

**Author response:**

Thank you very much for reviewing our manuscript and providing your constructive feedback. We deeply appreciate your time, effort and valuable guidance.

We have amended the manuscript and file naming to meet PLOS ONE’s style requirements, as requested:

- **Figure in-text citations, captions and files:**

We have amended the in-text figure citations (as Fig 1, Fig 2 etc) and the corresponding figure captions (labels, titles, legends) as per PLOS ONE’s guidelines.

The numbering order of the figures has been revised, and all corresponding figure citations, captions, and file names have been updated accordingly.

We have used the PACE tool to generate the figure files in TIFF format and to ensure adherence to PLOS ONE’s file requirements.

Figure files have been uploaded separately as individual files.

The following sections in the manuscript have been amended accordingly (amendments highlighted in yellow):

| **Page number** | **Line number** | **Amendment** | | **Amended text highlighted in yellow** |
| --- | --- | --- | --- | --- |
| 8 | 137 | ‘Box 1’ has been renamed ‘Fig 1’.  All figure in-text citations, caption labels and titles, and figure file names have been updated accordingly. | Updated and matched the figure in-text citation accordingly. | ‘The full eligibility criteria are presented in Fig 1.’ |
| 8 | 138 |  | Updated and matched the figure caption label and title accordingly. | **‘Fig 1. Eligibility criteria applied in the systematic review.’** |
|  |  |  | Updated and matched the figure file name accordingly.  Amended the dimensions of Figure 1 as per the guidelines. | Figure file name: Fig1.tif |
| 13 | 266 | Figure 2 citation | Updated and matched the figure in-text citation accordingly. | ‘excluded from the analyses and reasons for exclusion is provided in the S6 File. Fig. 2…’ |
| 13 | 269 | Figure 2 caption | Updated and matched the figure caption label and title accordingly. | **‘Fig 2. Flow of studies through the review.’** |
| 13 | 269-270 | Figure 2 legend | Changed font from 8 pt to standard font size 11 pt, to match the manuscript body text) |  |
|  |  | Figure 2 file name | Updated and matched the figure file name accordingly. | Figure file name: Fig2.tif |
| 17 | 358 | Figure 3 citation | Updated and matched the figure in-text citation accordingly. | ‘Fig 3 shows the effect direction plot for this section of the results. |
| 17 | 359-360 | Figure 3 caption | Updated and matched the figure caption label and title accordingly. | **‘Fig 3. Within-group syntheses - Effect direction plot of antipsychotics on appetite sensations.’** |
| 17 | 360-369 | Figure 3 legend | Changed font from 8 pt to standard font size 11 pt, to match the manuscript body text) |  |
|  |  | Figure 3 file name | Updated and matched the figure file name accordingly. | Figure file name: Fig3.tif |
| 19 | 411 | Figure 4 citation | Updated and matched the figure in-text citation accordingly. | ‘Fig 4 shows the effect direction plot for this section of the results.’ |
| 19 | 412-413 | Figure 4 caption | Updated and matched the figure caption label and title accordingly. | **‘Fig 4. Within-group syntheses - Effect direction plot of antipsychotics on food intake and dietary composition.’** |
| 19 | 413-417 | Figure 4 legend | Changed font from 8 pt to standard font size 11 pt, to match the manuscript body text) |  |
|  |  | Figure 4 file name | Updated and matched the figure file name accordingly. | Figure file name: Fig4.tif |
| 20 | 434 | Figure 5 citation | Updated and matched the figure in-text citation accordingly. | ‘Fig 5 shows the effect direction plot for this section of the results.’ |
| 20 | 435-436 | Figure 5 caption | Updated and matched the figure caption label and title accordingly. | **‘Fig 5. Within-group syntheses - Effect direction plot of antipsychotics on eating cognitions and behaviours.’** |
| 20 | 436-442 | Figure 5 legend | Changed font from 8 pt to standard font size 11 pt, to match the manuscript body text) |  |
|  |  | Figure 5 file name | Updated and matched the figure file name accordingly. | Figure file name: Fig5.tif |
| 21 | 463-464 | Renamed ‘Box 2’ as ‘Fig 6’. | Added an introductory sentence to figure 6 in the text.  Updated and matched the figure in-text citation accordingly. | ‘The GRADE Summary of Findings Table for key outcomes for within-group syntheses is presented in Fig 6.’ |
| 21 | 465 | Figure 6 caption | Updated and matched the figure caption label and title accordingly. | **‘Fig 6. Within-group syntheses GRADE Summary of Findings Table.’** |
| 21 | 465-467 | Figure 6 legend | Changed font from 8 pt to standard font size 11 pt, to match the manuscript body text) |  |
|  |  | Figure 6 file name | Updated and matched the figure file name accordingly. | Figure file name: Fig6.tif |
| 21 | 471 | Figure 7 citation | Updated and matched the figure in-text citation accordingly. | ‘Fig 7 shows the effect direction plot for this section of the results.’ |
| 22 | 472-473 | Figure 7 caption | Updated and matched the figure caption label and title accordingly. | **‘Fig 7. Between-group syntheses - Effect direction plot of antipsychotics on appetite sensations.’** |
| 22 | 473-479 | Figure 7 legend | Changed font from 8 pt to standard font size 11 pt, to match the manuscript body text) |  |
|  |  | Figure 7 file name | Updated and matched the figure file name accordingly. | Figure file name: Fig7.tif |
| 23 | 515 | Figure 8 citation | Updated and matched the figure in-text citation accordingly. | ‘Fig 8 shows the effect direction plot for this section of the results.’ |
| 23 | 516-517 | Figure 8 caption | Updated and matched the figure caption label and title accordingly. | **‘Fig 8. Between-group syntheses - Effect direction plot of antipsychotics on food intake and dietary composition.’** |
| 23-24 | 517-525 | Figure 8 legend | Changed font from 8 pt to standard font size 11 pt, to match the manuscript body text) |  |
|  |  | Figure 8 file name | Updated and matched the figure file name accordingly. | Figure file name: Fig8.tif |
| 25 | 560 | Figure 9 citation | Updated and matched the figure in-text citation accordingly. | ‘Fig 9 shows the effect direction plot for this section of the results.’ |
| 25 | 561-562 | Figure 9 caption | Updated and matched the figure caption label and title accordingly. | **‘Fig 9. Between-group syntheses - Effect direction plot of antipsychotics on the odds of developing eating disorders.’** |
| 25 | 562-571 | Figure 9 legend | Changed font from 8 pt to standard font size 11 pt, to match the manuscript body text) |  |
|  |  | Figure 9 file name | Updated and matched the figure file name accordingly. | Figure file name: Fig9.tif |
| 26 | 587 | Figure 10 citation | Updated and matched the figure in-text citation accordingly. | ‘Fig 10 shows the effect direction plot for this section of the results.’ |
| 26 | 588-589 | Figure 10 caption | Updated and matched the figure caption label and title accordingly. | **‘Fig 10. Between-group syntheses - Effect direction plot of antipsychotics on eating cognitions and behaviour.’** |
| 26 | 589-598 | Figure 10 legend | Changed font from 8 pt to standard font size 11 pt, to match the manuscript body text) |  |
|  |  | Figure 10 file name | Updated and matched the figure file name accordingly. | Figure file name: Fig10.tif |
| 28 | 639-640 | Renamed ‘Box 3’ as ‘Fig 11’. | Added an introductory sentence to figure 11 in the text.  Updated and matched the figure in-text citation accordingly. | ‘The GRADE Summary of Findings Table for key outcomes for between-group syntheses is presented in Fig 11.’ |
| 28 | 641 | Figure 11 caption | Updated and matched the figure caption label and title accordingly. | **‘Fig 11. Between-group syntheses GRADE Summary of Findings Table.’** |
| 28 | 641-643 | Figure 11 legend | Changed font from 8 pt to standard font size 11 pt, to match the manuscript body text) |  |
|  |  | Figure 11 file name | Updated and matched the figure file name accordingly. | Figure file name: Fig11.tif |
| 31 | 718 | Figure 12 citation | Updated and matched the figure in-text citation accordingly. | ‘…Fig 12.’ |
| 31 | 719 | Figure 12 caption | Updated and matched the figure caption label and title accordingly. | **‘Fig 12. Visual presentation of the thematic framework.’** |
| 31 | 719-720 | Figure 12 legend | Changed font from 8 pt to standard font size 11 pt, to match the manuscript body text). |  |
|  |  | Figure 12 file name | Updated and matched the figure file name accordingly. | Figure file name: Fig12.tif |
| 31 | 724 | Table 1 citation | Changed font to standard format as per the guidelines (i.e., not in bold). | ‘…provided in Table 1.’ |
| 32 | 727 | Table 1 caption | Changed font as per the guidelines. | **‘Table 1. GRADE-CERQual Summary of Qualitative Findings.’** |
| 32 | 728-729 | Table 1 footnotes | Changed font from 8 pt to standard font size 11 pt, to match the manuscript body text). |  |

- **Author affiliations:**

We have amended the author affiliations as per PLOS ONE’s ‘Title, author and affiliations formatting guidelines’.

The following sections in the title page have been amended accordingly (amendments highlighted in yellow):

| **Page number** | **Line number** | **Amendment** | **Amended text highlighted in yellow** |
| --- | --- | --- | --- |
| 1 | 11-22 | Affiliations have been listed individually and in full.  Abbreviations have been replaced with full names. | ^1^ Division of Nursing, Midwifery and Social Work, School of Health Sciences, Faculty of Biology, Medicine and Health, The University of Manchester, Manchester, UK.  ^2^ Manchester Academic Health Science Centre, The University of Manchester, Manchester, UK.  ^3^ National Institute for Health and Care Research Applied Research Collaboration (NIHR ARC) Greater Manchester Mental Health Theme, Manchester, UK.  ^4^ Division of Psychology and Mental Health, Faculty of Biology, Medicine and Health, The University of Manchester, Manchester, UK.  ^5^ Greater Manchester Mental Health NHS Foundation Trust, Manchester, UK.  ^6^ Pennine Care NHS Foundation Trust, Ashton-under-Lyne, Greater Manchester, UK.  ^7^ Faculty of Psychology, University College London, London, UK. |
| 1 | 8-10 | The affiliation numbers (in superscript) have been updated and matched to the corresponding authors accordingly. | Rasha Alkholy^1,2,3 *^, Karina Lovell^1,2,3^ , Penny Bee^1,2,3^, Rebecca Pedley^1,2,3^, Helen Louise Brooks^1,2^, Richard J Drake^2,4,5^, Prathiba Chitsabesan^3,6,7^, Anam Bhutta^1,2,3^, Abigail Brown^1^, Rebecca L Jenkins^1,2,3^, Andrew Grundy^1,2,3^ |

- We have adhered to PLOS ONE’s heading and manuscript body formatting guidelines.

**Academic Editor Comments to the Authors:**

2. Thank you for stating the following financial disclosure: [This paper presents research funded by the National Institute for Health and Care Research (NIHR) Applied Research Collaboration Greater Manchester (ARC-GM); Grant number NIHR200174 (https://arc-gm.nihr.ac.uk/mental-health [arc-gm.nihr.ac.uk]). KL is a co-applicant on the ARC award and the Mental Health Theme Lead. PB is the Deputy Theme Lead. The views expressed are those of the authors and not necessarily those of the NIHR or the Department of Health and Social Care.]. Please state what role the funders took in the study. If the funders had no role, please state: "The funders had no role in study design, data collection and analysis, decision to publish, or preparation of the manuscript." If this statement is not correct you must amend it as needed. Please include this amended Role of Funder statement in your cover letter; we will change the online submission form on your behalf.

**Author response:**

Thank you very much for your constructive feedback.

We confirm that the funders had no role in this study. We have added the Role of the Funder statement provided by the Academic Editor in the Cover Letter, as requested: "The funders had no role in study design, data collection and analysis, decision to publish, or preparation of the manuscript."

**Academic Editor Comments to the Authors:**

3. Please include captions for your Supporting Information files at the end of your manuscript, and update any in-text citations to match accordingly. Please see our Supporting Information guidelines for more information: <http://journals.plos.org/plosone/s/supporting-information> [track.editorialmanager.com].

**Author response:**

Thank you very much for your constructive feedback.

We have amended the Supporting Information files in-text citations, captions and file names to meet PLOS ONE’s Supporting Information guidelines:

- We have included captions for all Supporting Information files at the end of the manuscript (after the References section; including the file name, number and title).
- We have updated in-text citations of all Supporting Information files accordingly.
- Supporting information files have been uploaded separately as individual files.
- The following sections in the manuscript have been amended accordingly (amendments highlighted in yellow):

| **Page number** | **Line number** | **Amendment** | | **Amended text highlighted in yellow** |
| --- | --- | --- | --- | --- |
| 54 | 1437 | Added a “Supporting information" section. | Added the “Supporting information" section at the end of the manuscript, and adhered to PLOS ONE’s style requirements (i.e., using a Level 1 heading, bold type, 18pt font). | **13. Supporting information** |
| 7 | 129 | Added the S1 File including the PRISMA checklist. | Added the S1 File in-text citation accordingly. | ‘Meta-Analyses (PRISMA) [30] (S1 File) and the Enhancing transparency in reporting the…’ |
| 54 | 1438 |  | Added the S1 File caption (name, number, title) at the end of the manuscript in the section titled “Supporting information”. | **‘S1 File. PRISMA checklist.’** |
|  |  |  | Uploaded the S1 File as an individual file and adhered to the supporting information file naming guidelines. | File name: S1_File.docx |
| 8 | 147 | Added the S2 File including the information sources and search strategy for the systematic review. | Added the S2 File in-text citation accordingly. | ‘S2 File’. |
| 54 | 1439 |  | Added the S2 File caption (name, number, title) at the end of the manuscript in the section titled “Supporting information”. | **‘S2 File. Information sources and search strategy for the systematic review.’** |
|  |  |  | Uploaded the S2 File as an individual file and adhered to the supporting information file naming guidelines. | File name: S2_File.docx |
| 9 | 155 | Added the S3 File.  This file includes tables of all data extracted from the primary search sources for the systematic review including: name of data extractors and date of data extraction, confirmation that the study was eligible to be included in the review, all data extracted from each study for the reported systematic review that would be needed to replicate the analyses.  When data or supporting information were obtained from other relevant papers, this was clearly indicated and references for these papers were provided. | Added the S3 File in-text citation accordingly. | ‘(S3 File) and verified by another; disagreements were resolved by…’ |
| 54 | 1440 |  | Added the S3 File caption (name, number, title) at the end of the manuscript in the section titled “Supporting information”. | **‘S3 File. Data extraction forms.’** |
|  |  |  | Uploaded the S3 File as an individual file and adhered to the supporting information file naming guidelines. | File name: S3_File.xlsx |
| 11 | 210-211 | Added the S4 file. This file incudes the methods used to prepare the data for synthesis, including data conversion and handling of missing summary statistics | Added the S4 File in-text citation accordingly. | ‘The methods used to prepare the data for synthesis, including data conversion and handling of missing summary statistics, are provided in the S4 File.’ |
| 54 | 1441 |  | Added the S4 File caption (name, number, title) at the end of the manuscript in the section titled “Supporting information”. | **‘S4 File. Methods used to prepare the quantitative data for synthesis.’** |
|  |  |  | Uploaded the S4 File as an individual file and adhered to the supporting information file naming guidelines. | File name: S4_File.docx |
| 11 | 215 | Added the S5 file. This file incudes a table of study and participant characteristics of the 55 included quantitative studies. The table also includes data on the prevalence of antipsychotic-induced changes in eating-related outcomes and antipsychotic-related factors associated with these changes (e.g., head-to-head comparisons between antipsychotics, effect of different doses) summarised in narrative format. | Added the S5 File in-text citation accordingly. | ‘narrative format (S5 File).’ |
| 14 | 280 |  |  | ‘the S5 File. The studies varied in terms of study design, study…’ |
| 54 | 1442 |  | Added the S5 File caption (name, number, title) at the end of the manuscript in the section titled “Supporting information”. | **‘S5 File. Study and participant characteristics of the 55 included quantitative studies.’** |
|  |  |  | Uploaded the S5 File as an individual file and adhered to the supporting information file naming guidelines. | File name:  S5_File.docx |
| 13 | 265-266 | Added the S6 file. This file incudes a list of all studies retrieved for full-text examination, including those that were excluded from the analyses and reasons for exclusion. | Added the S6 File in-text citation accordingly. | ‘A list of all studies retrieved for full-text examination, including those that were excluded from the analyses and reasons for exclusion is provided in the S6 File.’ |
| 54 | 1443-1444 |  | Added the S6 File caption (name, number, title) at the end of the manuscript in the section titled “Supporting information”. | **‘S6 File. Studies retrieved for full-text examination, including those that were excluded from the analyses and reasons for exclusion.’** |
|  |  |  | Uploaded the S6 File as an individual file and adhered to the supporting information file naming guidelines. | File name:  S6_File.docx |
| 15 | 314 | Added the S7 file. This file incudes a list of all scales/ questionnaires used to assess eating-related outcomes. | Added the S7 File in-text citation accordingly. | S7 File.’ |
| 54 | 1445 |  | Added the S6 File caption (name, number, title) at the end of the manuscript in the section titled “Supporting information”. | **‘S7 File. Scales/questionnaires used to assess eating-related outcomes.’** |
|  |  |  | Uploaded the S7 File as an individual file and adhered to the supporting information file naming guidelines. | File name:  S7_File.docx |
| 16 | 344 | Added the S8 file. This file incudes the completed risk of bias assessments of the 55 included quantitative studies using the Mixed Methods Assessment Tool. | Added the S8 File in-text citation accordingly. | S8 File.’ |
| 54 | 1446-1447 |  | Added the S8 File caption (name, number, title) at the end of the manuscript in the section titled “Supporting information”. | **‘S8 File. Risk of bias assessment of the 55 included quantitative studies using the Mixed Methods Assessment Tool.’** |
|  |  |  | Uploaded the S8 File as an individual file and adhered to the supporting information file naming guidelines. | File name:  S8_File.docx |
| 17 | 355 | Added the S9 file. This file incudes a detailed account of the within- and between-group quantitative syntheses. | Added the S9 File in-text citation accordingly. | ‘…is available in the S9 File and S10 File, respectively.’ |
| 54 | 1448 |  | Added the S9 File caption (name, number, title) at the end of the manuscript in the section titled “Supporting information”. | **‘S9 File. Detailed account of the quantitative syntheses.’** |
|  |  |  | Uploaded the S9 File as an individual file and adhered to the supporting information file naming guidelines. | File name:  S9_File.docx |
| 17 | 355 | Added the S10 File. This file incudes the GRADE Evidence Profile for each key outcome. It contains information about the body of evidence, judgments about each of the quality of evidence factors, key results (using vote counting with direction of effect), and the quality of evidence rating for each outcome. | Added the S10 File in-text citation accordingly. | ‘…are available in the S9 File and S10 File, respectively.’ |
| 54 | 1449 |  | Added the S10 File caption (name, number, title) at the end of the manuscript in the section titled “Supporting information”. | **‘S10 File. The GRADE Evidence Profile for key outcomes.’** |
|  |  |  | Uploaded the S10 File as an individual file and adhered to the supporting information file naming guidelines. | File name:  S10_File.docx |
| 28 | 648 | Added the S11 file. This file incudes the study and participant characteristics of the 6 included qualitative studies. | Added the S11 File in-text citation accordingly. | ‘…the S11 File.’ |
| 54 | 1450 |  | Added the S11 File caption (name, number, title) at the end of the manuscript in the section titled “Supporting information”. | **‘S11 File. Study and participant characteristics of the 6 included qualitative studies.’** |
|  |  |  | Uploaded the S11 File as an individual file and adhered to the supporting information file naming guidelines. | File name:  S11_File.docx |
| 30 | 696 | Added the S12 file. This file incudes the risk of bias assessments of the 6 included qualitative studies using the Mixed Methods Appraisal Tool (MMAT). | Added the S12 File in-text citation accordingly. | ‘The risk of bias assessments of the 6 included qualitative studies is provided in the S12 File.’ |
| 54 | 1451-1452 |  | Added the S12 File caption (name, number, title) at the end of the manuscript in the section titled “Supporting information”. | **‘S12 File. Risk of bias assessments of the 6 included qualitative studies using the Mixed Methods Appraisal Tool (MMAT).’** |
|  |  |  | Uploaded the S12 File as an individual file and adhered to the supporting information file naming guidelines. | File name:  S12_File.docx |
| 31 | 724-725 | Added the S13 file. This file incudes the GRADE-CERQual Evidence Profile. | Added the S13 File in-text citation accordingly. | ‘Detailed description of our confidence assessments is provided in the GRADE-CERQual Evidence Profile (S13 File).’ |
| 54 | 1453 |  | Added the S13 File caption (name, number, title) at the end of the manuscript in the section titled “Supporting information”. | **‘S13 File. The GRADE-CERQual assessment of confidence in qualitative evidence.’** |
|  |  |  | Uploaded the S13 File as an individual file and adhered to the supporting information file naming guidelines. | File name:  S13_File.docx |

**Academic Editor Comments to the Authors:**

4. As required by our policy on Data Availability, please ensure your manuscript or supplementary information includes the following:

This information can be included in the main text, supplementary information, or relevant data repository. Please note that providing these underlying data is a requirement for publication in this journal, and if these data are not provided your manuscript might be rejected.

**Author response:**

Thank you very much for your constructive feedback. The location of the requested information within the main text and the supplementary information files is indicated in the following section.

- A numbered table of all studies identified in the literature search, including those that were excluded from the analyses.

**Author response:**

The requested table is provided in the **S6 File.** This file incudes a numbered table of all studies retrieved for full-text examination, including those that were excluded from the analyses and reasons for exclusion.

- For every excluded study, the table should list the reason(s) for exclusion.

**Author response:**

The requested information is provided in the **S6 File.**

- If any of the included studies are unpublished, include a link (URL) to the primary source or detailed information about how the content can be accessed.

**Author response:**

All of the studies included in the systematic review are published.

- A table of all data extracted from the primary research sources for the systematic review and/or meta-analysis. The table must include the following information for each study:
- Name of data extractors and date of data extraction
- Confirmation that the study was eligible to be included in the review.
- All data extracted from each study for the reported systematic review and/or meta-analysis that would be needed to replicate your analyses.
- If data or supporting information were obtained from another source (e.g. correspondence with the author of the original research article), please provide the source of data and dates on which the data/information were obtained by your research group.

**Author response:**

The requested table is provided in the **S3 File.** This file includes tables of all data extracted from the records included in the systematic review including: name of data extractors, date of data extraction, confirmation that the study was eligible to be included in the review, all data extracted from each study for the reported systematic review that would be needed to replicate the analyses. When data or supporting information were obtained from other relevant records, this was clearly indicated and references for these records (and dates of access) were provided.

- If applicable for your analysis, a table showing the completed risk of bias and quality/certainty assessments for each study or outcome. Please ensure this is provided for each domain or parameter assessed. For example, if you used the Cochrane risk-of-bias tool for randomized trials, provide answers to each of the signalling questions for each study. If you used GRADE to assess certainty of evidence, provide judgements about each of the quality of evidence factor. This should be provided for each outcome.

**Author response:**

The **S8 File** includes the completed risk of bias assessments of the 55 included quantitative studies using the Mixed Methods Assessment Tool (MMAT). The **S12 File** includes the completed risk of bias assessments of the 6 included qualitative studies using the Mixed Methods Appraisal Tool (MMAT). The GRADE Summary of Findings Table for key outcomes for within-group syntheses is presented in **Fig 6**. The GRADE Summary of Findings Table for key outcomes for between-group syntheses is presented in **Fig 11**. The **S10 File** incudes the GRADE Evidence Profile for each key outcome. It contains information about the body of evidence, judgments about each of the quality of evidence factors, key results (using vote counting with direction of effect), and the quality of evidence rating for each outcome. The GRADE-CERQual Summary of Qualitative Findings is presented in **Table 1.** The **S13 File** incudes the GRADE-CERQual Evidence Profile for each qualitative synthesis finding.

- An explanation of how missing data were handled.

This information can be included in the main text, supplementary information, or relevant data repository. Please note that providing these underlying data is a requirement for publication in this journal, and if these data are not provided your manuscript might be rejected.

**Author response:**

A detailed explanation of the methods used to prepare the data for synthesis, including data conversion and handling of missing summary statistics is provided in the **S4 File**. Overall, data required for the within-group and between-group syntheses were provided in numerical format. Occasionally, required data were presented in graphical format. In these cases, lead authors of the respective records were contacted via email and requested to provide the missing data. In the absence of a response, the graphical representation of results was used to determine the direction of effect. When this approach was employed, it was clearly indicated in the detailed ‘Summary of effect measures of antipsychotic medications on eating-related outcomes’ tables and ‘GRADE’ tables provided in the Supplementary Files.

**Academic Editor Comments**

5. Please review your reference list to ensure that it is complete and correct. If you have cited papers that have been retracted, please include the rationale for doing so in the manuscript text, or remove these references and replace them with relevant current references. Any changes to the reference list should be mentioned in the rebuttal letter that accompanies your revised manuscript. If you need to cite a retracted article, indicate the article’s retracted status in the References list and also include a citation and full reference for the retraction notice.

**Author response:**

Thank you for your constructive feedback. We have not cited any retracted papers in our manuscript. The following references have been amended to adhere with PLOS ONE’s referencing style (“Vancouver” style):

| **Reason for amendment** | **Amended reference** |
| --- | --- |
| Corrected the in-text citation on page 18, line 378 | ‘to three months [78], while in the study by Karagianis and colleagues [94] participants had…’ |
| Style requirements (title in sentence case) | 14. Pillinger T, Beck K, Gobjila C, Donocik JG, Jauhar S, Howes OD. Impaired glucose homeostasis in first-episode schizophrenia: A systematic review and meta-analysis. JAMA Psychiatry. 2017;74(3):261-9. doi: 10.1001/jamapsychiatry.2016.3803. |
| Style requirements (title in sentence case) | 23. Roberts LW, Louie AK, Guerrero APS, Balon R, Beresin EV, Brenner A, et al. Premature mortality among people with mental illness: Advocacy in academic psychiatry. Acad Psychiatry. 2017;41(4):441-6. doi: 10.1007/s40596-017-0738-9. |
| Added DOI | 25. Stogios N, Smith E, Asgariroozbehani R, Hamel L, Gdanski A, Selby P, et al. Exploring patterns of disturbed eating in psychosis: A scoping review. Nutrients. 2020;12(12). doi: 10.3390/nu12123883. |
| Style requirements (electronic book chapter) | 38. Borenstein M, Hedges LV, Higgins JPT, Rothstein HR. Meta-analysis methods based on direction and p-values. In: Introduction to meta‐analysis [Internet]. 2009. [cited 2023/10/09]; [325-30]. Available from: <https://doi.org/10.1002/9780470743386.ch36>. |
| Style requirements (title in sentence case) | 51. Ntalkitsi S, Efthymiou D, Bozikas V, Vassilopoulou E. Halting the metabolic complications of antipsychotic medication in patients with a first episode of psychosis: How far can we go with the Mediterranean Diet? A pilot study. Nutrients. 2022;14(23). doi: 10.3390/nu14235012. |
| Style requirements (title in sentence case) | 55. de Beaurepaire R. Binge Eating Disorders in antipsychotic-treated patients with schizophrenia: Prevalence, antipsychotic specificities, and changes over time. J Clin Psychopharmacol. 2021;41(2):114-20. doi: 10.1097/JCP.0000000000001357. |
| Style requirements (title in sentence case) | 64. Friedrich M, Fugiel J, Sadowska J. Assessing effects of diet alteration on carbohydrate–lipid metabolism of antipsychotic-treated schizophrenia patients in interventional study. Nutrients. 2023;15(8). doi: 10.3390/nu15081871. |
| Style requirements (title in sentence case) | 75. Archie SM, Goldberg JO, Akhtar-Danesh N, Landeen J, McColl L, McNiven J. Psychotic disorders, eating habits, and physical activity: Who is ready for lifestyle changes? Psychiatric Services. 2007;58(2):233-9. doi: 10.1176/ps.2007.58.2.233. |
| Included the correct DOI | 79. Huang J, Hei GR, Yang Y, Liu CC, Xiao JM, Long YJ, et al. Increased appetite plays a key role in olanzapine-induced weight gain in first-episode schizophrenia patients. Front Pharmacol. 2020;11:878. doi: 10.3389/fphar.2020.00739. |
| Style requirements (title in sentence case) | 99. Teff KL, Rickels K, Alshehabi E, Rickels MR. Metabolic impairments precede changes in hunger and food intake following short-term administration of second-generation antipsychotics. J Clin Psychopharmacol. 2015;35(5):579-82. doi: 10.1097/JCP.0000000000000393. |
| Style requirements (title in sentence case) | 104. Haracz K, Hazelton M, James C. The "double whammy": Women's experiences of weight gain after diagnosis and treatment for schizophrenia spectrum disorders. J Nerv Ment Dis. 2018;206(5):303-9. doi: 10.1097/NMD.0000000000000803. |

**Reviewer 1 Comments:**

The systematic review manuscript is scientifically sound with conclusions that are well supported by the data findings from the reviewed studies.

There was no statistical analysis required in the systematic review. Authors utilised vote counting on the direction of effects in view of the quality of studies regarding power.

Manuscript was presented in standard English. There are only two typographic errors noted by this reviewer in the write-up: Line 176 (page 8) “trails” for ‘trials’, and line 285 (page 13) “13,13,502” for “13,502”.

In sum, the manuscript presented a procedurally detailed mixed methods systematic review of the impacts of antipsychotic medications on eating experiences and eating behaviour of subjects, with adherence to up-to-date best practice guidelines.

The findings are germane, with appropriate interpretation, discussions and inferences on the existing quantitative and qualitative studies, on the subject. The involvement of a lived experience advisory panel adds to the validity of the review, with good recommendations for future studies.

**Author response:**

Thank you very much for reviewing our paper and providing your constructive feedback. We are extremely grateful for your time, effort and valuable guidance.

We apologise for the typographical errors and express our sincere gratitude to the Reviewer for identifying them.

We have amended the manuscript accordingly (amendments highlighted in yellow):

| **Page number** | **Line number** | **Amended text highlighted in yellow** |
| --- | --- | --- |
| 3 | 46 | ‘high-quality placebo-controlled trials, it is pertinent to interpret the quantitative findings’ |
| 10 | 177 | Due to lack of direct evidence from high-quality placebo-controlled trials, we had to’ |
| 14 | 289 | ‘The sample sizes ranged from 1 to 3,362 participants and included a total of 13,502’ |

N.B.: The original line numbers and pages have been revised following the incorporation of all amendments.

**Reviewer 2 Comments:**

This is a well researched and technically sound paper that highlights the paucity of high level evidence on an important topic. Reading the title one would think that the paper will highlight some distinction between outcomes related to eating experiences and eating behaviours hence the need to explicitly specify them in the title, however there is no distinction in the body of the paper between the two. I would therefore suggest that perhaps a better title would be "The impacts of antipsychotic medications on eating related outcomes: A mixed methods systematic review".

**Author response:**

Thank you very much for reviewing our paper and providing your constructive feedback. We are extremely grateful for your time, effort and valuable guidance.

We agree with the Reviewer’s recommendations regarding the title. Using ‘eating-related outcomes’ instead of ‘eating experiences and eating behaviours’ avoids potential confusion and more comprehensively encompasses the wide spectrum of eating-related outcomes. We have amended the full title and short title of this manuscript accordingly.

The following sections in the Title page have been amended accordingly (amendments highlighted in yellow):

| **Page number** | **Line number** | **Amended text highlighted in yellow** |
| --- | --- | --- |
| 1 | 1-3  (Full title) | The impacts of antipsychotic medications on eating-related outcomes: A mixed methods systematic review |
| 1 | 5, 6  (Short title) | Antipsychotic medications and eating-related outcomes: A mixed methods systematic review |

We wish to express our sincere gratitude to the Academic Editor and the Reviewers for their valuable guidance. We look forward to receiving your response regarding our revised submission and will gladly address any questions or comments you may have.

Thank you.
